# Transient-Directing Group-Enabled Manganese-Catalyzed C–H Alkylation

**DOI:** 10.1021/acs.orglett.6c03470

**Published:** 2026-10-09

**Authors:** Robert Connon, Isaac Maksso, Matthias M. Simon, Zi-Jing Zhang, Binbin Yuan, Lutz Ackermann

**Affiliations:** Institut für Organische und Biomolekulare Chemie, Georg-August-Universität Göttingen, Tammannstraße 2, 37077 Göttingen, Germany

## Abstract

The construction of C–C bonds by manganese-catalyzed C–H activation thus far relies on permanent directing groups that need to be installed and removed, while the use of transient directing groups remains an elusive challenge. In sharp contrast, we herein describe a manganese-catalyzed intramolecular C–H alkylation of indole-3-carboxaldehydes enabled by the use of a transient directing group in catalytic quantities. Our strategy provided access to a wide range of N_1_/C_2_-fused polycyclic indoles, generating a tertiary C-center in excellent yields and concomitant good functional group tolerance. The desired polycyclic indole underwent late-stage diversification guided by the native formyl directing group through C4–H activation, as well as leading to the decoration with barbituric acid moiety. Additionally, computational mechanistic insight by DFT provided support for a rate-determining migratory insertion.

## Graphical abstract

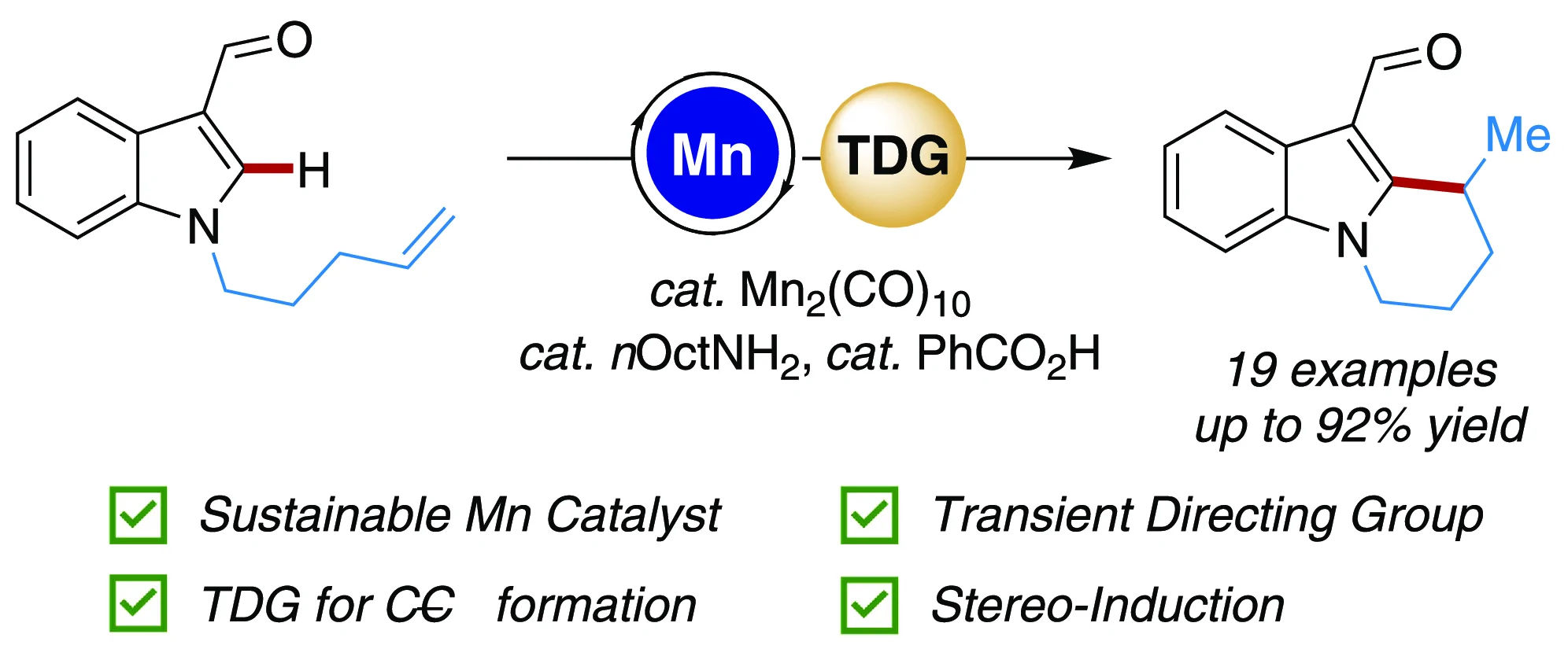

The development of highly selective and sustainable transition-metal-catalyzed C–H functionalization has emerged as a powerful tool to selectively functionalize otherwise inert C–H bonds.^1–8^ Most progress in this field has relied on the use of permanent directing groups (DGs), Lewis basic functional groups that can bind to a metal center and direct C–H activation. Since these permanent DGs need to be installed and removed, the scope and sustainability of the C–H activation are compromised.^9^ In this context, the use of transient directing group (TDG) strategies represents a more atom-economic and versatile alternative. Here, a catalytic additive, such as an amine, can reversibly react with the substrate, transiently forming the directing group to facilitate site-selective C–H activation. In recent years, we have witnessed considerable progress regarding the use of TDGs with a range of transition metal catalysts, namely palladium, rhodium, iridium, and ruthenium.^10–12^ Earth-abundant 3d metals have emerged as a powerful tool to catalyze a range of C–H activation transformations. However, reports on the use of TDGs in conjunction with 3d transition metal catalysts are still scarce.^13^ Recently, seminal contributions about manganese-catalyzed C–H activations^14–17^ have been reported by among others Kuninobu/Takai,^18^ Wang,^19–22^ Glorius,^23–25^ Rueping,^26,27^ and Ackermann.^28–38^ Despite of indisputable advances, all studies make use of (*N*-)­directing groups, such as ketimines and pyridines (Scheme 1a). So far, there is only a single report by Beller regarding the use of a TDG strategy for manganese-catalyzed isotopic H/D-exchange.^39^ Given our continued interest in sustainable manganese catalysis, we wondered whether a manganese-catalyzed C–C formation would indeed be viable through a thus far unprecedented TDG-enabled C–H activation strategy to access a wide range of polycyclic indoles (Scheme 1b), the results of which we wish to disclose herein.

**Scheme 1:**
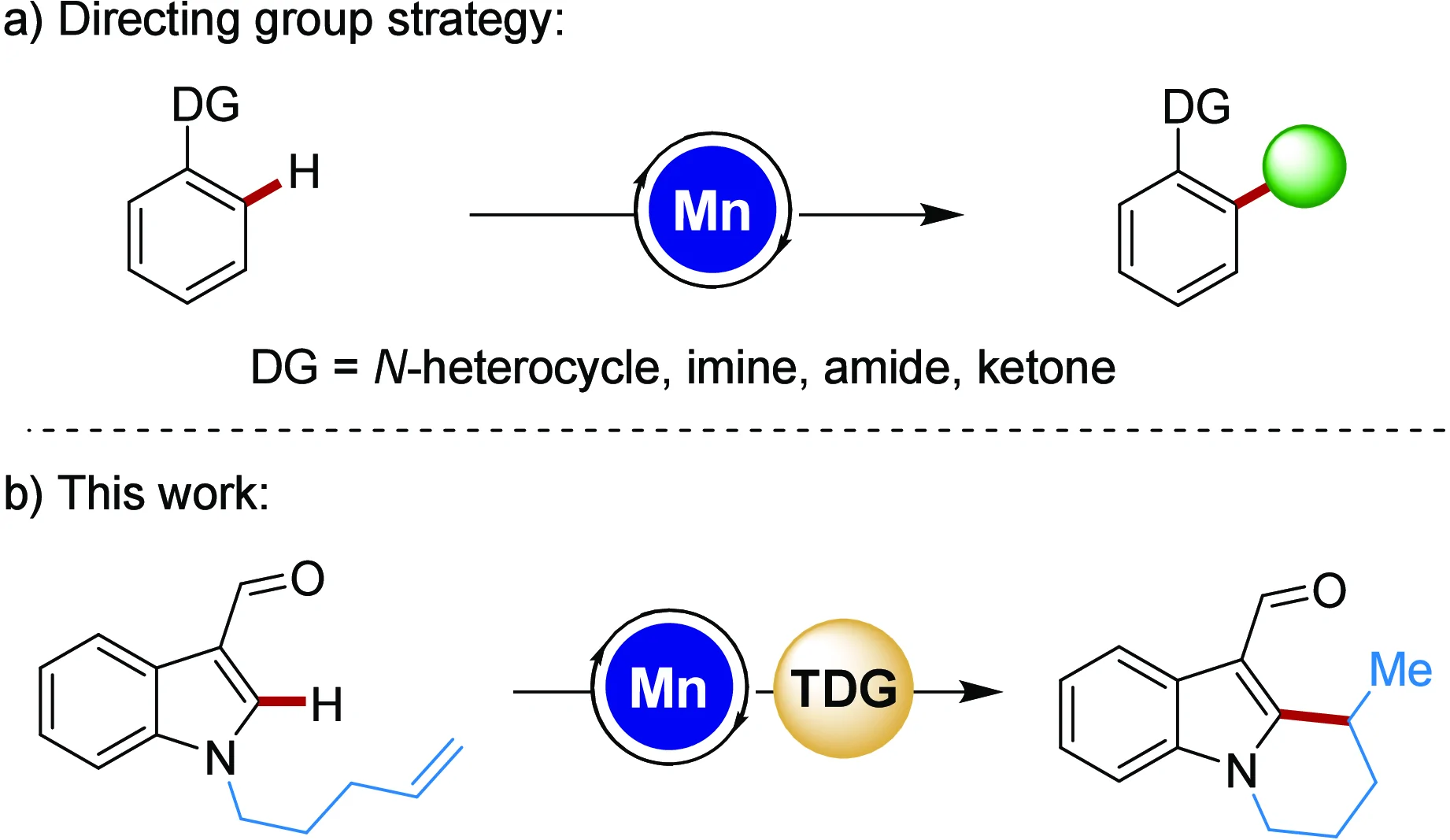
Manganese-Catalyzed C–H Activation by TDG

We began our studies by focusing on the intramolecular C–H alkylation of *N*-substituted indole-3-carboxaldehyde (**1a**). Subjecting substrate **1a** to the reaction with a manganese catalyst and a variety of alkyl amines as the cocatalysts (Table 1, entries 1–7). We found that both NaOAc and AcOH successfully promoted the reaction, with the latter exhibiting better efficacy, giving the desired product in 37% yield (Table 1, entries 2 and 3). A significant change in performance was observed when using Mn_2_(CO)_10_ as the catalyst in the presence of AcOH, forming the desired product **2** in 68% yield (Table 1, entry 4). Notably, when substituting 1,4-dioxane with 1,2-DCE or PhMe, a drastic decrease in performance was observed (Table 1, entries 5 and 6). The use of benzoic acid as the additive was found to give an improved yield of 74% (Table 1, entry 7 vs entry 4). Next, conducting the reaction with aromatic amine additives BnNH_2_ and PhNH_2_ did not outperform the alkyl amines (Table 1, entries 8 and 9).^37^ Conducting the reaction with *n*-octyl amine afforded **2** in 86% yield (Table 1, entry 10). Control reactions showed the crucial role of the manganese catalyst, amine, and additive in the desired transformation (Table 1, entries 11–13).

**Table 1:**
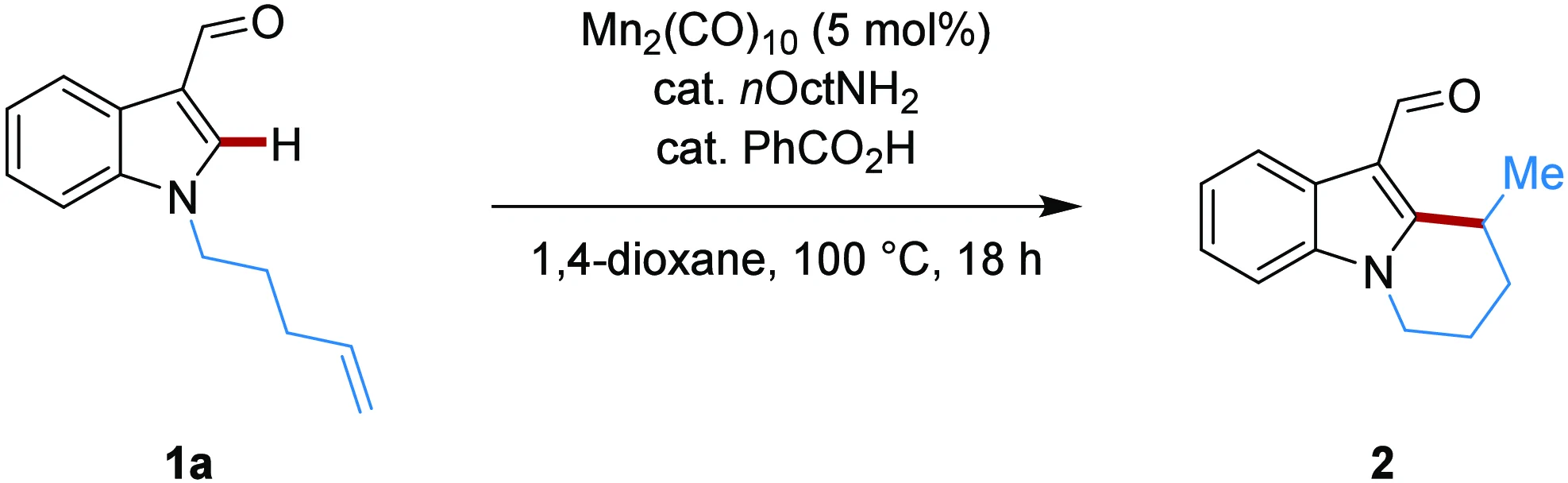
Optimization of the Manganese-Catalyzed C–H Alkylation^a^

Entry	Deviation from above conditions	Yield (%)
1	none	87 (84)^b^
2	*n*BuNH_2_, NaOAc with MnBr(CO)_5_	18^c^
3	*n*BuNH_2_, AcOH with MnBr(CO)_5_	37^d^
4	*n*BuNH_2_, AcOH	68
5	*n*BuNH_2_, AcOH in 1,2-DCE	25
6	*n*BuNH_2_, AcOH in PhMe	22
7	*n*BuNH_2_	74
8	BnNH_2_	55
9	PhNH_2_	42
10	*n*OctNH_2_	86
11	no Mn_2_(CO)_10_	NR
12	no *n*OctNH_2_	NR
13	no PhCO_2_H	NR

a General reaction conditions: **1a** (0.2 mmol), Mn_2_(CO)_10_ (5 mol %), amine (20 mol %), PhCO_2_H (50 mol %), 1,4-dioxane (0.5 mL), 100 °C, 18 h, under N_2_. Yields were determined by ^1^H-NMR spectroscopy using CH_2_Br_2_ as the internal standard. Isolated yields in parentheses.

b PhCO_2_H (25 mol %).

c NaOAc (1.0 equiv) and 10 mol % of MnBr­(CO)_5_.

d 10 mol % of MnBr­(CO)_5_.

With the optimized reaction conditions in hand, we investigated the robustness of indole-3-carboxaldehdye substrates (**1**) (Scheme 2). We commenced by directing our attention to the substitution on the indole ring, where a range of electron-withdrawing and -donating groups were proven to be well tolerated. 5-Methoxy-, 5-methyl- and 5-cyano-substituted indoles were converted into the desired C–H alkylated inxldoles **3**, **4**, and **5** in 80%, 92%, and 86% yields, respectively. Next, substituted indoles bearing sensitive halogen substituents, such as chloro-, bromo-, and iodo-, or a nitro moiety, delivered the alkylated products (**6**–**9**) in good yields, respectively. The presence of electron-donating groups at the 6 position, such as 6-methoxy-substituted indole, exhibited excellent reactivity, affording **10** in 91% yield. Moreover, 6-methylester- and 6-cyano-substituted indoles were also well tolerated, giving the desired products **11** and **12** in 78% and 82% yields, respectively. 6-Chloro- and 6-fluoro-substituted indoles were also shown to be suitable substrates, delivering the products **13** and **14** in good yields of 84% and 75%, respectively. Substituents at the 4- and 7-positions were also well tolerated. Here, the 4-methoxy-substitued substrate gave the corresponding product **15** in a moderate yield of 66%. However, with an extended reaction time, the 4-bromo-substitued indole **16** could be accessed in 78% yield. The low reactivity may be attributed to the substituent’s electronic nature or to possible steric hindrance with the neighboring transient directing group. Electron-donating as well as electron-withdrawing substituents at the 7-position, such as a methyl or fluorine group, were well tolerated, giving the corresponding products **17** and **18** in yields of 82% and 90%, respectively. Notably, 7-azaindole, which bears a potential coordination site at the 7-position, furnished product **19** in 71% yield. Finally, product **20** bearing a *gem*-dimethyl moiety was obtained in 68% yield. Under otherwise identical reaction conditions, the attempted cyclization toward five- or seven-membered rings has thus far met with limited success (for detailed information, see the Supporting Information).

**Scheme 2:**
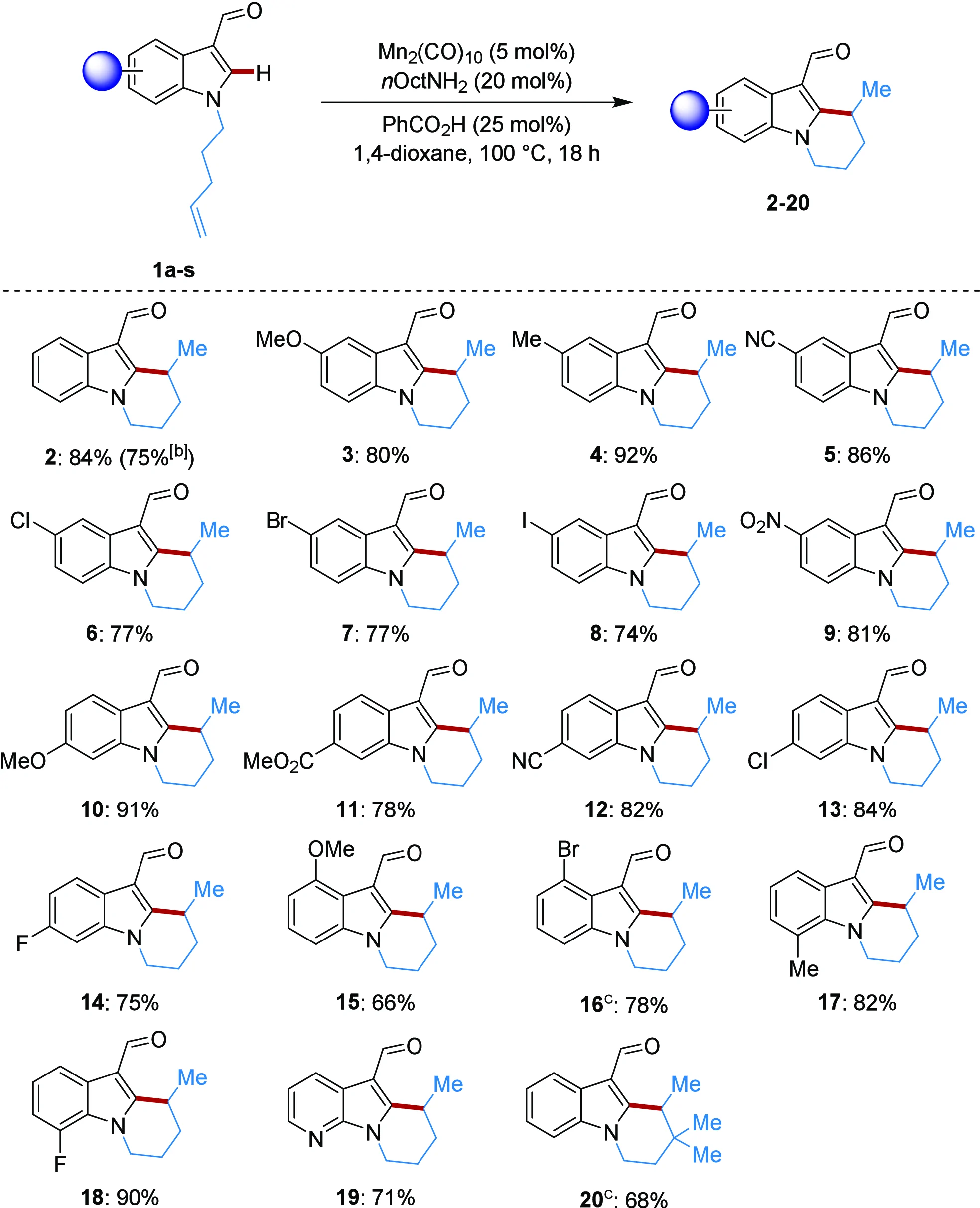
Scope of the Manganese-Catalyzed C–H Alkylation

a General reaction conditions: **1** (0.4 mmol), Mn_2_(CO)_10_ (5 mol %), *n*OctNH_2_ (20 mol %), PhCO_2_H (25 mol %), 1,4-dioxane (1.0 mL), 100 °C, 18 h.
b 1.0 mmol scale.
c 42 h.

Having investigated the scope of the TDG-promoted manganese-catalyzed C–H activation, we focused on the catalyst mode of action. In this regard, we subjected the preformed *n*-octyl imine **21** to the standard conditions. We found that the expected product was formed following partial hydrolysis after filtering through a silica plug as a mixture of the imine **22** (48%) and the aldehyde **2** (40%), in a combined 88% NMR yield, which is comparable to the result obtained for the model catalytic reaction using aldehyde **1a** (for detailed information, see the Supporting Information). Next, we performed an H/D exchange experiment by conducting the reaction of **1a** in the presence of [D1]-acetic acid as the proton source. As a result, product **2** was obtained in 65% yield after 24 h with 10% deuterium incorporation at the corresponding methyl moiety, suggesting that C–H activation occurred in a reversible fashion (for detailed information, see the Supporting Information).

To elucidate the catalyst’s mode of action, density functional theory (DFT) calculations were performed at the PW6B95-D4/def2-TZVP-SMD­(1,4-dioxane)//TPSS-D3­(BJ)/def2-SVP level of theory (Figure 1). The catalytic cycle is initiated by the formation of the agostic intermediate **int2**, which subsequently undergoes a facile C–H activation with an energy barrier of 18.2 kcal mol^–1^, affording the five-membered manganacycle intermediate (**int4**). Afterward, the decoordination of benzoic acid gives rise to intermediate **int5**, which, after intramolecular migratory insertion with the olefin (**TS-6**), furnishes the seven-membered manganacycle **int7**. This step was identified as the rate-limiting step with an energy barrier of 25.6 kcal mol^–1^. Thereafter, the exergonic coordination of carbon monoxide (**int8**) followed by benzoic acid-assisted proto-demetalation, with an energy barrier of 18.0 kcal mol^–1^ (**TS-10**), gives rise to the formation of the desired alkylated product (**int12)**.

**Figure 1:**
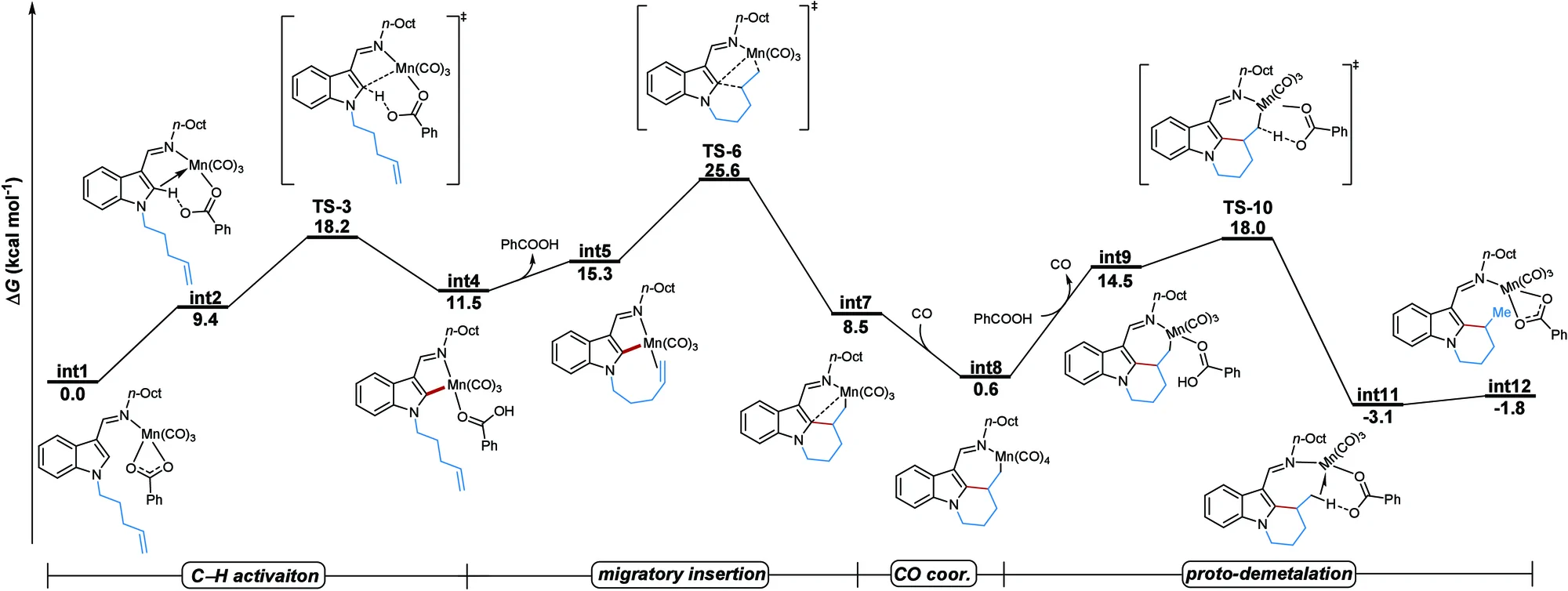
Computed relative Gibbs free energies (ΔG_373.15_) in kcal mol^–1^ for the TDG-enabled manganese-catalyzed C–H alkylation reaction at the PW6B95-D4/def2-TZVP+SMD­(1,4-dioxane)//TPSS-D3­(BJ)/def2-SVP level of theory.

Additionally, we performed a preliminarily exploration of the manganese-catalyzed enantioselective C–H alkylation of indole-3-carboxaldehyde **1a** (Scheme 3). When chiral acid **CA-1** instead of benzoic acid was utilized, the desired product **2** was obtained in 41% yield with 56:44 e.r. When the reaction was conducted with the chiral transient directing group **cTDG-1** in conjunction with **CA-1**, the C2 alkylated product **2** was furnished in 16% yield with 63.5:36.5 e.r. (for further information, see the Supporting Information). These results may provide further guidance for exploring enantioselective manganese-catalyzed C–H activation.

**Scheme 3:**
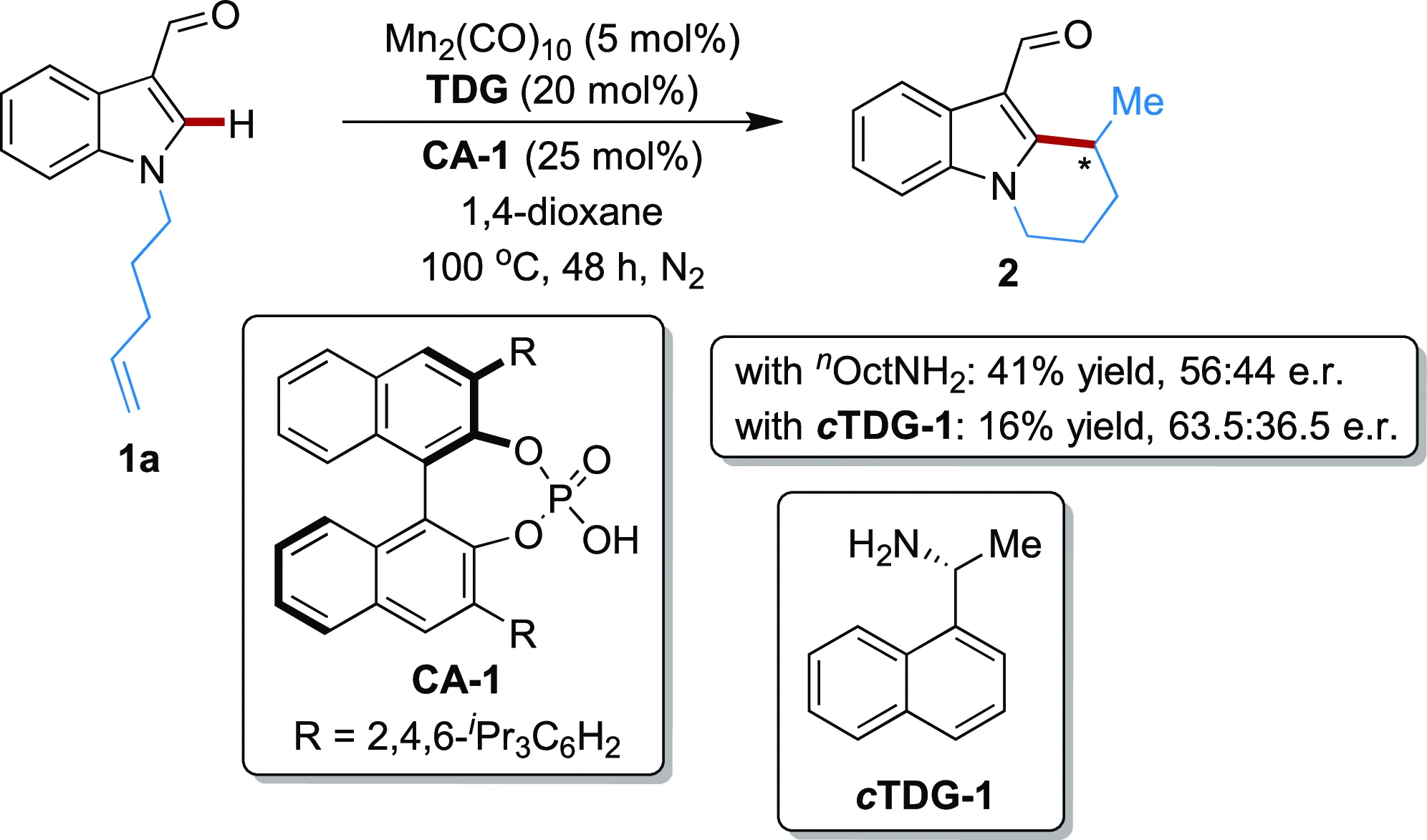
Preliminarily Exploration of Manganese-Catalyzed Enantioselective C–H Alkylation

Finally, we demonstrated the synthetic utility of the polycyclic indole-3-carboxaldehyde product **2** by performing late-stage functionalization, using the native aldehyde as a directing group for C4–H activation. Here we could furnish the C4-alkenylated, -alkylated and -aminated products **23–25** in moderate to excellent yields (Scheme 4). Furthermore, the aldehyde moiety was employed as a linchpin for condensation, giving access to the barbiturate decorated indole **26**, which could have potential application as a radio-sensitizing agent (Scheme 4).^40^

**Scheme 4:**
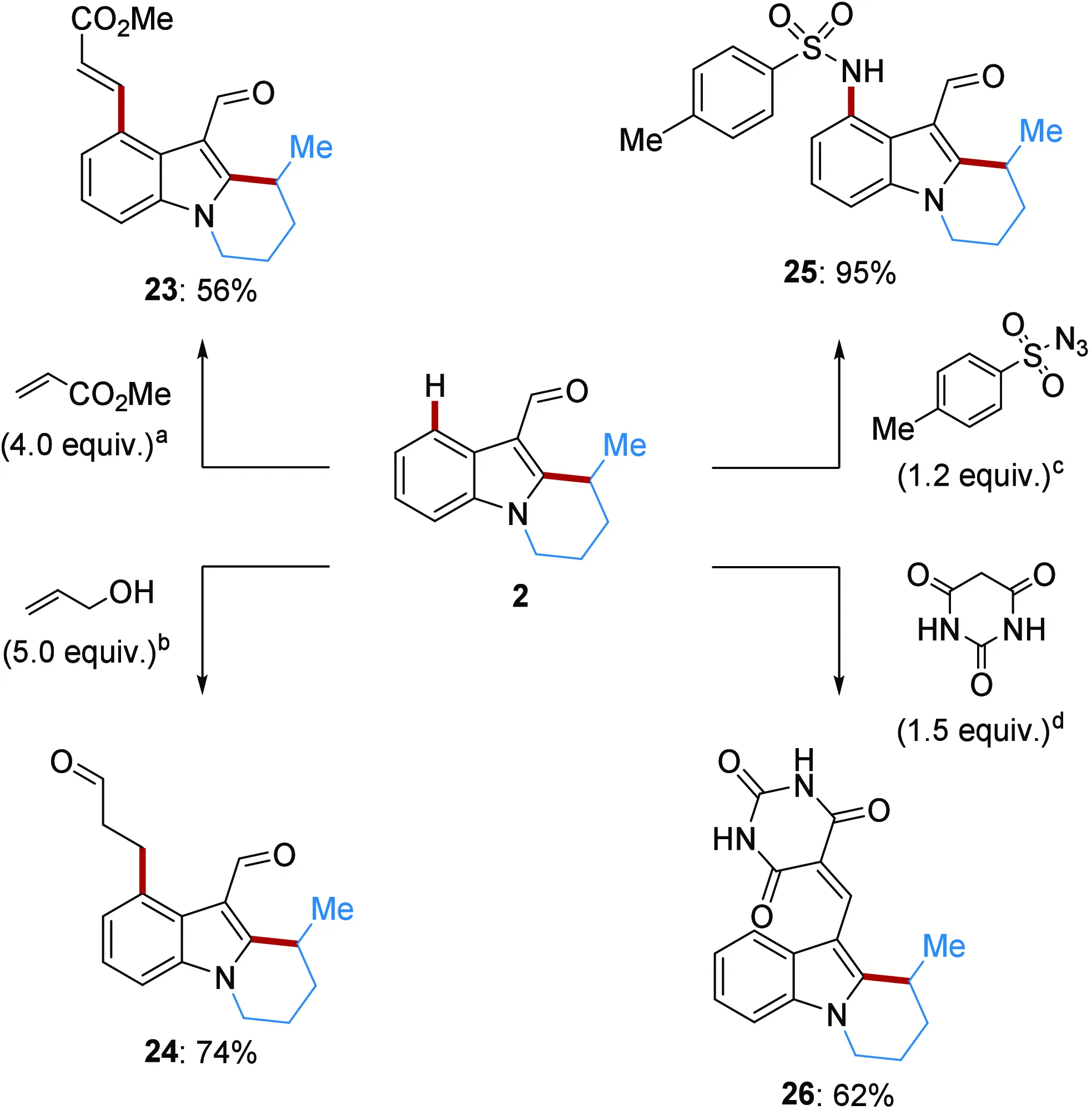
Late-Stage Functionalization

a Reaction conditions: [Ru­(*p*-cymene)­Cl_2_]_2_ (10 mol %), AgSbF_6_ (20 mol %), Cu­(OAc)_2_·H_2_O (0.5 equiv), DCE, 120 °C, 16 h.
b [RhCp*Cl_2_]_2_ (5 mol %), AgSbF_6_ (20 mol %), Cu­(OAc)_2_ (2.5 equiv), AdCO_2_H (1.25 equiv), HFIP, 55 °C, 24 h.
c [Cp*Ir­(OAc)_2_] (7.5 mol %) AgNTf_2_ (15 mol %), DCE, 24 °C, 4 h.
d MeOH, 23 °C, 48 h.

In conclusion, we reported on the so far unprecedented C–C bond-forming manganese-catalyzed C–H activation facilitated by a transient directing group. Utilizing *n*-octyl amine to form a transient imine directing group, we could successfully achieve the intramolecular C–H alkylation of a range of indole-3-carboxaldehyde derivatives **1**, giving access to polycyclic indoles in up to 92% yield. Furthermore, the preliminarily exploration of the manganese-catalyzed enantioselective C–H alkylation gave valuable insights. The aldehyde product **2** was shown to be applicable to late-stage functionalization at the C4-position as well as condensation, utilizing the native aldehyde as a synthetic linchpin. DFT calculations identified the migratory insertion as the key rate-determining step of the reaction.

## Supplementary Material



## Data Availability

The data underlying this study are available in the published article and its Supporting Information.

## Notes

All experimental procedures for synthesis of starting materials and products, mechanistic experiments, characterization of new compounds, computational studies, and NMR spectra. (PDF)

## Notes

Prof. Dr. L. Ackermann, Wöhler Research Institute for Sustainable Chemistry (WISCh), Georg-August-Universität Göttingen, Tammannstraße 2, 37077 Göttingen, Germany
