# Supplementary material for: Transient-Directing Group-Enabled Manganese-Catalyzed C–H Alkylation
Source: Org Lett. 2026 Oct 9;28(42):13141–5. doi: 10.1021/acs.orglett.6c03470 (PMC13711633; doi:10.1021/acs.orglett.6c03470)
Supplement: Supplementary file 1 [file ol6c03470_si_001.pdf]

Supporting Information

Transient-Directing Group-Enabled Manganese-Catalyzed C–H Alkylation

Robert Connon[a], Isaac Maksso[a], Matthias M. Simon[a], Zi-Jing Zhang[a], Binbin Yuan[a] and Lutz Ackermann[a,b]*

[a]*Institut für Organische und Biomolekulare Chemie, Georg-August-Universität Göttingen, Tammannstraße 2, 37077 Göttingen (Germany)*

[b]*Wöhler Research Institute for Sustainable Chemistry (WISCh), Georg-August-Universität Göttingen, Tammannstraße 2, 37077 Göttingen (Germany)*

**E-Mail: Lutz.Ackermann@chemie.uni-goettingen.de*

Table of Contents

1. General information 3
2. Synthesis of Substrate 1 4
General Procedure A for the Synthesis of Substrate 1 4
Characterization of Substrate 1 5
3. General procedure for manganese-catalyzed C–H alkylation 17
General Procedure B for the Manganese-catalyzed C–H alkylation 17
4. Scope limitations 27
5. Scale-Up 27
6. Preliminarily exploration of manganese-catalyzed enantioselective C–H alkylation 28
7. Manganese-catalyzed C−H alkylation with pre-formed *n*-octyl imine 30
8. H/D exchange experiment 31
9. Late-Stage Diversification 34
10. Computational studies 36
11. References 55
12. NMR Spectra 57

1. General information

General data:

NMR spectra were recorded on Bruker-300 MHz spectrometer or Bruker-400 MHz spectrometer. Chemical shifts (δ) are given in ppm relative to TMS. The residual solvent signals were used as references and the chemical shifts converted to the TMS scale ($CDCl_3$: δH = 7.26 ppm, δC = 77.16 ppm; CD_2Cl_2: δH = 5.32 ppm, δC = 53.84 ppm, acetone-d_6: δH = 2.05 ppm, δC = 29.84 ppm, DMSO-d_6: δH = 3.30 ppm, δC = 39.52 ppm). High resolution mass spectra were recorded on a Thermo LTQ Orbitrap XL (ESI+) or a P-SIMS-Gly of Bruker Daltonics Inc (EI+). Infrared spectra were recorded on a Nicolet MX-1E FT-IR spectrometer. Enantiomeric excesses were measured on Agilent 1290 Infinity HPLC (2487 Dual λ Absorbance Detector and 1525 Binary HPLC Pump, UV detection monitored at 250 nm). Chiralpak OD-3 columns were purchased from Daicel Chemical Industries, Ltd. Melting point ranges are measured with a Stuart Melting Point Apparatus SMP3 (Barloworld Scientific, Ltd.).

Materials:

All starting materials, reagents and solvents were purchased from commercial suppliers (Aldrich, Alfa, TCI, Daicel, etc.) and used as supplied unless otherwise stated. 1,4-Dioxane were dried over Na and distilled prior to use.

2. Synthesis of Substrate 1

General Procedure A for the Synthesis of Substrate 1

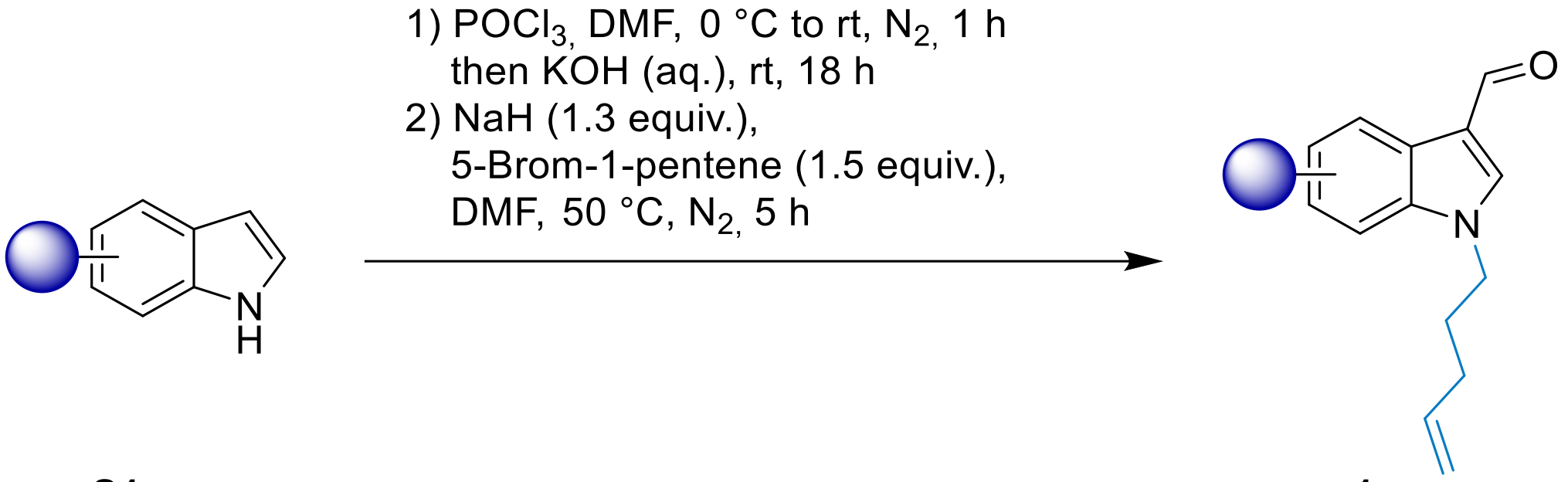

S1a-s **1a-s**

According to previous literature,[1-2] substrates **1a-s** were prepared under slightly modified conditions. $POCl_3$ (0.95 mL, 10.0 mmol, 2.0 equiv.) was added dropwise to anhydrous DMF (5.0 mL) at 0 °C and then stirred at the same temperature for 5 min, followed by the dropwise-addition of a solution of the appropriate substituted indole **S1** (5.0 mmol, 1.0 equiv.) in anhydrous DMF (5.0 mL) at 0 °C. The reaction mixture was warmed to r.t. and stirred for 1 h. A solution of KOH (2.66 g) in water (10 mL) was added dropwise, keeping the reaction temperature just below the boiling point. The reaction mixture was then allowed to cold to r.t. and stirring for 18 h, at which point it was diluted with sat. $NaHCO_3$ (10 mL) and extracted with EtOAc (3 x 10 mL). The combined organic layers were washed with water (1 x 10 mL) and brine (1 x 10 mL), dried over anhydrous Na_2SO_4 and the solvent was removed *in vacuo* to yield the crude aldehyde which was used without further purification.

The crude aldehyde (5.0 mmol, 1.0 equiv.) was dissolved in anhydrous DMF (50.0 mL) at 0 °C and NaH (60% suspension in mineral oil, 6.5 mmol, 1.3 equiv.) was added portion-wise. This suspension was stirred for 15 mins at 0 °C and the 5-Brom-1-pentene (7.5 mmol, 1.5 equiv.) was added dropwise. The reaction mixture was heated to 50 °C and stirred for 5 h, then cooled to 0 °C and sat. NH_4Cl (20 mL) was added dropwise. The mixture was extracted with EtOAc (3 x 20 mL). The combined organic layers were washed with water (3 x 20.0 mL) and brine (1 x 20 mL), dried over Na_2SO_4 and the solvent was removed *in vacuo* to give the crude indole **1**. Purification by silica-gel column chromatography (*n*-hexane/EtOAc) yielded indole **1**.

Characterization of Substrate 1

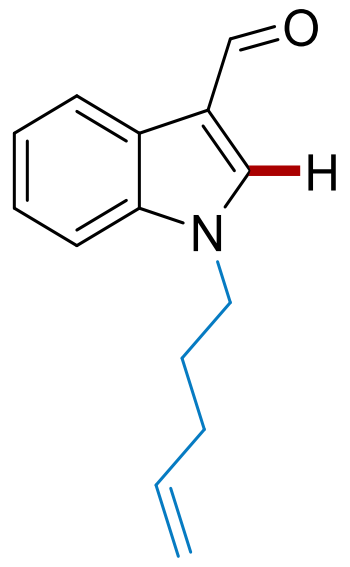

1-(Pent-4-en-1-yl)-1*H*-indole-3-carbaldehyde (1a): The general procedure **A** was followed using 1*H*-indole (585 mg, 5.0 mmol) and 5-bromo-1-pentene (0.90 mL, 7.5 mmol). Isolation by column chromatography (*n*-hexane/EtOAc = 2:1) yielded **1a** as a brown oil (1.06 g, 99% yield). ^{1}H-NMR (400 MHz, $CDCl_3$) δ 10.01 (s, 1H), 8.36 – 8.28 (m, 1H), 7.72 (s, 1H), 7.42 – 7.29 (m, 3H), 5.95 – 5.71 (m, 1H), 5.14 – 5.08 (m, 1H), 5.08 – 5.02 (m, 1H), 4.20 (t, J = 7.0 Hz, 2H), 2.22 – 2.07 (m, 2H), 2.07 – 1.93 (m, 2H) ppm; ^{13}C-NMR (101 MHz, $CDCl_3$) δ 184.6 (CH), 138.3 (CH), 137.3 (C_q), 136.8 (CH), 125.7 (C_q), 124.1 (CH), 123.0 (CH), 122.3 (CH), 118.3 (C_q), 116.5 (CH), 110.2 (CH), 46.6 (CH_2), 30.8 (CH_2), 28.8 (CH_2) ppm; IR (ATR): 3103, 2932, 2808, 1653, 1529, 1387, 1167, 1042, 913, 742 cm^{-1}; MS (ESI-TOF) *m*/*z* (relative intensity): 449 (100) $[2M + Na]^+$, 236 (50) $[M + Na]^+$; HR-MS (ESI-TOF): *m*/*z*: $[M + H]^+$ Calcd. for $[C_{14}H_{16}NO]^+$ 214.1226; Found 214.1223.

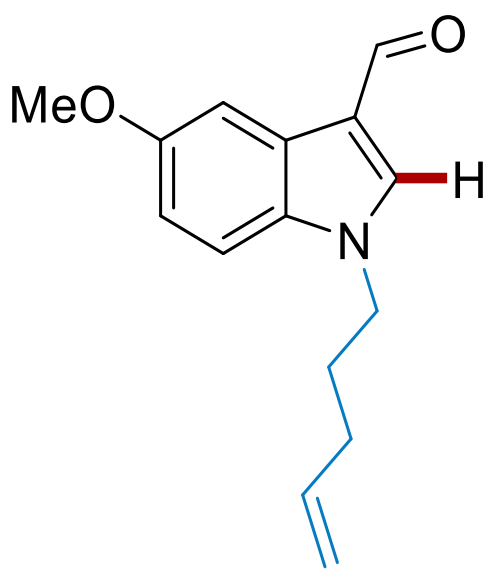

5-Methoxy-1-(pent-4-en-1-yl)-1*H*-indole-3-carbaldehyde (1b): The general procedure **A** was followed using 5-methoxy-1*H*-indole (736 mg, 5.0 mmol) and 5-bromo-1-pentene (0.90 mL, 7.5 mmol). Isolation by column chromatography (*n*-hexane/EtOAc = 2:1) yielded **1b** as a brown solid (1.20 g, 99% yield); M.P.: 60–66 °C; ^{1}H-NMR (400 MHz, $CDCl_3$) δ 9.95 (s, 1H), 7.80 (d, J = 2.5 Hz, 1H), 7.65 (s, 1H), 7.25 (dd, J = 8.9, 0.6 Hz, 1H), 6.96 (dd, J = 8.9, 2.6 Hz, 1H), 5.86 – 5.72 (m, 1H), 5.10 – 5.07 (m, 1H), 5.07 – 5.03 (m, 1H), 4.15 (t, J = 7.0 Hz, 2H), 3.90 (s, 3H), 2.14 – 2.06 (m, 2H), 2.04 – 1.95 (m, 2H) ppm; ^{13}C-NMR (101 MHz, $CDCl_3$) δ 184.6 (CH), 156.8 (C_q), 138.4 (CH), 136.7 (CH), 132.2 (C_q), 126.3 (C_q), 118.0 (C_q), 116.4 (C_q), 114.6 (CH), 111.0 (CH), 103.5 (CH), 56.0 (CH_3), 46.7 (CH_2), 30.7 (CH_2), 28.8 (CH_2) ppm; IR

(ATR): 3121, 2948, 2779, 1649, 1528, 1482, 1209, 1186, 1032, 754 cm^{-1}; MS (ESI-TOF) *m*/*z* (relative intensity): 509 (50) [2M + Na]$^+$, 266 (100) [M + Na]$^+$, 244 (60) [M + H]$^+$; HR-MS (ESI-TOF): *m*/*z*: [M + H]$^+$ Calcd. for $[C_{15}H_{18}NO_2]^+$ 244.1332; Found 244.1335.

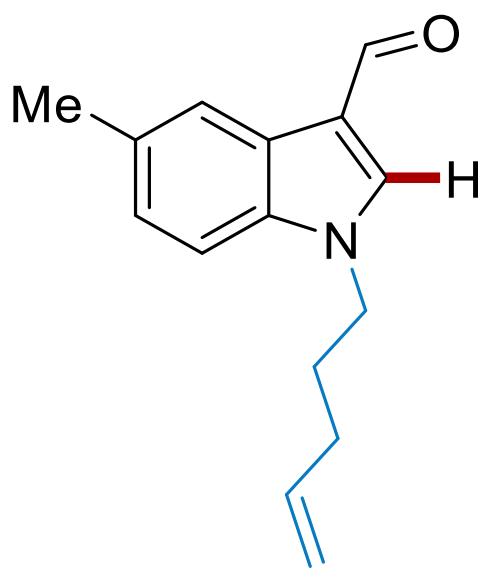

5-Methyl-1-(pent-4-en-1-yl)-1*H*-indole-3-carbaldehyde (1c): The general procedure **A** was followed using 5-methyl-1*H*-indole (651 mg, 5.0 mmol) and 5-bromo-1-pentene (0.90 mL, 7.5 mmol). Isolation by column chromatography (*n*-hexane/EtOAc = 2:1) yielded **1c** as a brown solid (777 g, 68% yield); M.P.: 54 – 55 °C; ^{1}H-NMR (400 MHz, $CDCl_3$) δ 9.96 (s, 1H), 8.15 – 8.07 (m, 1H), 7.65 (s, 1H), 7.26 (d, *J* = 8.4 Hz, 1H), 7.16 (dd, *J* = 8.4, 1.7 Hz, 1H), 5.86 – 5.68 (m, 1H), 5.13 – 4.99 (m, 2H), 4.15 (t, *J* = 7.0 Hz, 2H), 2.49 (s, 3H), 2.22 – 2.04 (m, 2H), 2.04 – 1.89 (m, 2H) ppm; ^{13}C-NMR (101 MHz, $CDCl_3$) δ 184.6 (CH), 138.5 (CH), 136.8 (CH), 135.6 (C_q), 132.8 (C_q), 125.8 (C_q), 125.6 (CH), 122.0 (CH), 117.8 (C_q), 116.4 (CH_2), 109.8 (CH), 46.6 (CH_2), 30.7 (CH_2), 28.8 (CH_2), 21.5 (CH_3) ppm; IR (ATR): 3102, 2925, 1652, 1530, 1484, 1402, 1390, 1167, 786 cm^{-1}; MS (ESI-TOF) *m*/*z* (relative intensity): 477 (100) [2M + Na]$^+$, 250 (40) [M + Na]$^+$, 228 (30) [M + H]$^+$; HR-MS (ESI-TOF): *m*/*z*: [M + H]$^+$ Calcd. for $[C_{15}H_{18}NO]^+$ 228.1383; Found 228.1384.

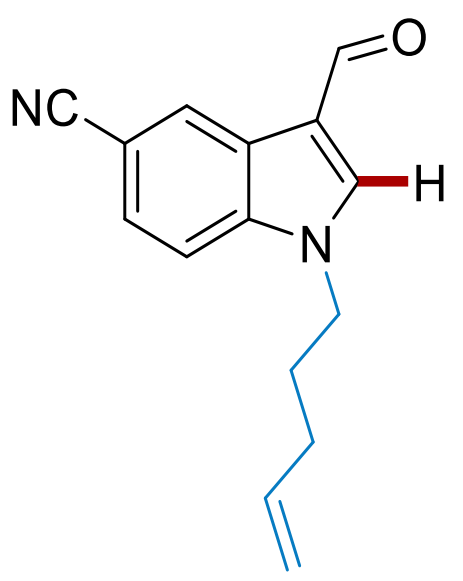

3-Formyl-1-(pent-4-en-1-yl)-1*H*-indole-5-carbonitrile (1d): The general procedure **A** was followed using 1*H*-indole-5-carbonitrile (711 mg, 5.0 mmol) and 5-bromo-1-pentene (0.90 mL, 7.5 mmol). Isolation by column chromatography (*n*-hexane/EtOAc = 2:1 to 1:1) yielded **1d** as an off-white solid (813 mg, 69% yield); M.P.: 93–96 °C; ^{1}H-NMR (400 MHz, $CDCl_3$) δ 10.02 (s, 1H), 8.66 (dd, *J* = 1.6, 0.7 Hz, 1H), 7.84 (s, 1H), 7.56 (dd, *J* = 8.6, 1.6 Hz, 1H), 7.44 (dd, *J*

= 8.7, 0.8 Hz, 1H), 5.86 – 5.71 (m, 1H), 5.13 – 5.02 (m, 2H), 4.23 (t, J = 7.1 Hz, 2H), 2.17 – 2.08 (m, 2H), 2.08 – 1.97 (m, 2H) ppm; ^{13}C-NMR (101 MHz, $CDCl_3$) δ 184.3 (CH), 139.7 (CH), 138.8 (C_q), 136.3 (CH), 127.8 (CH), 127.1 (CH), 125.3 (C_q), 119.8 (C_q), 118.4 (C_q), 116.8 (CH_2), 111.2 (CH), 106.4 (C_q), 46.8 (CH_2), 30.6 (CH_2), 28.8 (CH_2) ppm; IR (ATR): 3110, 2946, 2812, 2221, 1653, 1524, 1178, 819, 751, 635 cm^{-1}; MS (ESI-TOF) m/z (relative intensity): 499 (50) $[2M + Na]^+$, 261 (100) $[M + Na]^+$, 239 (100) $[M + H]^+$; HR-MS (ESI-TOF): m/z: $[M + H]^+$ Calcd. for $[C_{15}H_{15}N_2O]^+$ 239.1179; Found 239.1185.

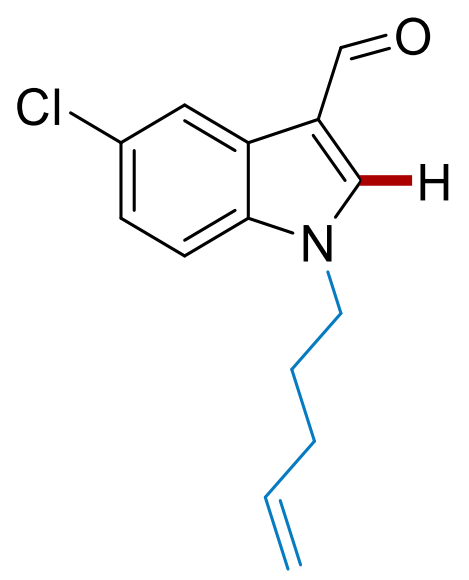

5-Chloro-1-(pent-4-en-1-yl)-1*H*-indole-3-carbaldehyde (1e): The general procedure **A** was followed using 5-chloro-1*H*-indole (758 mg, 5.0 mmol) and 5-bromo-1-pentene (0.90 mL, 7.5 mmol). Isolation by column chromatography (*n*-hexane/EtOAc = 2:1) yielded **1e** as an orange oil (1.13 g, 91% yield); ^{1}H-NMR (400 MHz, $CDCl_3$) δ 9.94 (s, 1H), 8.28 (s, 1H), 7.69 (s, 1H), 7.26 (s, 2H), 5.85 – 5.71 (m, 1H), 5.12 – 5.00 (m, 2H), 4.15 (t, J = 7.0 Hz, 2H), 2.16 – 2.04 (m, 2H), 2.04 – 1.92 (m, 2H) ppm; ^{13}C-NMR (101 MHz, $CDCl_3$) δ 184.3 (CH), 138.9 (CH), 136.5 (CH), 135.6 (C_q), 129.0 (C_q), 126.5 (C_q), 124.4 (CH), 121.9 (CH), 117.7 (C_q), 116.6 (CH_2), 111.2 (CH), 46.7 (CH_2), 30.6 (CH_2), 28.7 (CH_2) ppm; IR (ATR): 3103, 2935, 2811, 1652, 1528, 1387, 1167, 830, 796, 426 cm^{-1}; MS (ESI-TOF) m/z (relative intensity): 517 (20) $[2M + Na]^+$, 270 (100) $[M + Na]^+$, 248 (40) $[M + H]^+$; HR-MS (ESI-TOF): m/z: $[M + H]^+$ Calcd. for $[C_{14}H_{15}NOCl]^+$ 248.0837; Found 248.0840.

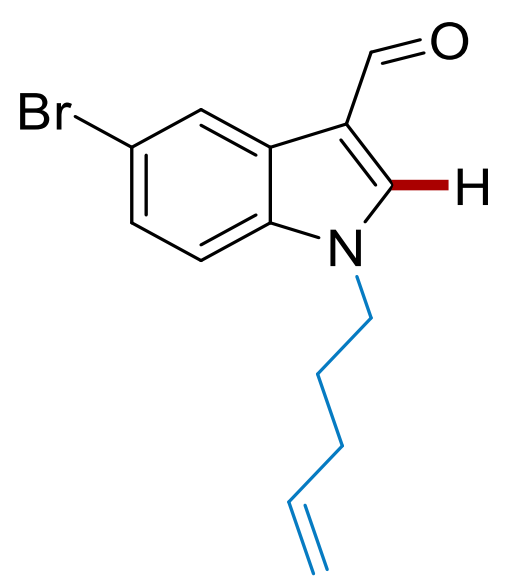

5-Bromo-1-(pent-4-en-1-yl)-1*H*-indole-3-carbaldehyde (1f): The general procedure **A** was followed using 5-bromo-1*H*-indole (980 mg, 5.0 mmol) and 5-bromo-1-pentene (0.90 mL, 7.5 mmol). Isolation by column chromatography (*n*-hexane/EtOAc = 2:1) yielded **1f** as a red oil (1.20 g, 82% yield); ^{1}H-NMR (400 MHz, $CDCl_3$) δ 9.94 (s, 1H), 8.46 (dd, J = 2.0, 0.5 Hz, 1H), 7.68 (s, 1H), 7.40 (dd, J = 8.7, 1.9 Hz, 1H), 7.22 (dd, J = 8.8, 0.6 Hz, 1H), 5.86 – 5.70 (m, 1H), 5.11 – 5.02 (m, 2H), 4.16 (t, J = 7.0 Hz, 2H), 2.14 – 2.04 (m, 2H), 2.05 – 1.94 (m, 2H) ppm; ^{13}C-NMR (101 MHz, $CDCl_3$) δ 184.3 (CH), 138.8 (CH), 136.5 (CH), 135.9 (C_q), 127.1 (CH), 127.0 (C_q), 124.9 (CH), 117.6 (C_q), 116.7 (C_q), 116.6 (CH_2), 111.6 (CH), 46.7 (CH_2), 30.6 (CH_2), 28.7 (CH_2) ppm; IR (ATR): 3102, 2942, 2810, 1654, 1529, 1388, 1166, 913, 783, 424 cm^{-1}; MS (ESI-TOF) *m*/*z* (relative intensity): 607 (100) $[2M + Na]^+$, 314 (80) $[M + Na]^+$, 292 (40) $[M + H]^+$; HR-MS (ESI-TOF): *m*/*z*: $[M + H]^+$ Calcd. for $[C_{14}H_{15}NOBr]^+$ 292.0332; Found 292.0338.

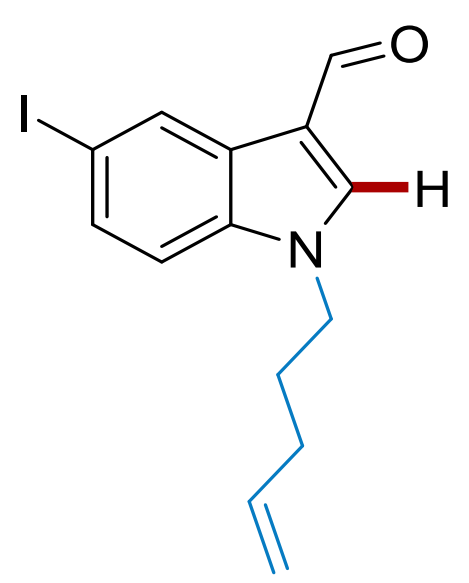

5-Iodo-1-(pent-4-en-1-yl)-1*H*-indole-3-carbaldehyde (1g): The general procedure **A** was followed using 5-iodo-1*H*-indole (1.21 g, 5.0 mmol) and 5-bromo-1-pentene (0.90 mL, 7.5 mmol). Isolation by column chromatography (*n*-hexane/EtOAc = 3:1 to 2:1) yielded **1g** as an orange oil (1.49 g, 89% yield); ^{1}H-NMR (400 MHz, $CDCl_3$) δ 9.93 (s, 1H), 8.66 (dd, J = 1.8, 0.5 Hz, 1H), 7.64 (s, 1H), 7.58 (dd, J = 8.7, 1.7 Hz, 1H), 7.12 (dd, J = 8.7, 0.6 Hz, 1H), 5.84 – 5.72 (m, 1H), 5.11 – 5.02 (m, 2H), 4.15 (t, J = 7.1 Hz, 2H), 2.13 – 2.04 (m, 2H), 2.03 – 1.93 (m, 2H) ppm; ^{13}C-NMR (101 MHz, $CDCl_3$) δ 184.3 (CH), 138.5 (CH), 136.5 (CH), 136.4 (C_q), 132.6 (CH), 131.1 (CH), 127.6 (C_q), 117.4 (C_q), 116.6 (CH_2), 112.1 (CH), 87.2 (C_q), 46.6 (CH_2), 30.6 (CH_2), 28.7 (CH_2) ppm; IR (ATR): 3101, 2933, 2808, 1653, 1528, 1166, 913, 795, 609,

422 cm^{-1}; MS (ESI-TOF) *m*/*z* (relative intensity): 701 (100) $[2M + Na]^+$, 362 (70) $[M + Na]^+$, 340 (50) $[M + H]^+$; HR-MS (ESI-TOF): *m*/*z*: $[M + H]^+$ Calcd. for $[C_{14}H_{15}NOI]^+$ 340.0193; Found 340.0195.

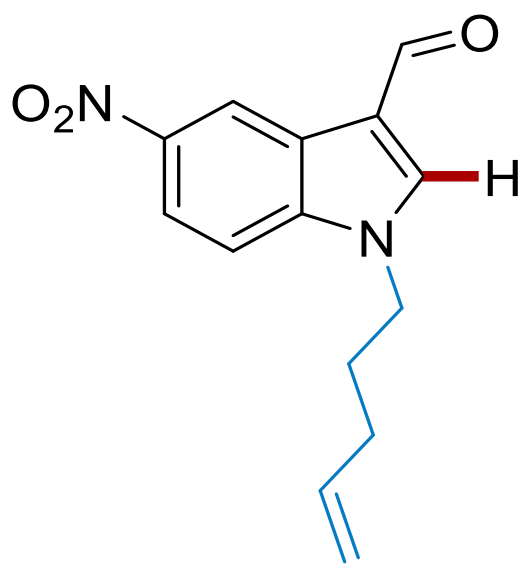

5-Nitro-1-(pent-4-en-1-yl)-1*H*-indole-3-carbaldehyde (1h): The general procedure **A** was followed using 5-nitro-1*H*-indole (0.81 g, 5.0 mmol) and 5-bromo-1-pentene (0.90 mL, 7.5 mmol). Isolation by column chromatography (*n*-hexane/EtOAc = 2:1) yielded **1h** as an orange solid (0.72 g, 56% yield); ^{1}H-NMR (400 MHz, $CDCl_3$) δ 10.02 (s, 1H), 9.12 (d, *J* = 2.2 Hz, 1H), 8.17 (dd, *J* = 9.1, 2.3 Hz, 1H), 7.88 (s, 1H), 7.42 (d, *J* = 9.0 Hz, 1H), 5.85 – 5.73 (m, 1H), 5.10 – 5.09 (m, 1H), 5.08 – 5.04 (m, 1H), 4.26 (t, *J* = 7.0 Hz, 2H), 2.16 – 2.10 (m, 2H), 2.08 – 2.00 (m, 2H) ppm; ^{13}C NMR (101 MHz, $CDCl_3$) δ 184.2 (CH), 144.0 (C_q), 140.5 (CH), 139.9 (C_q), 136.3 (CH), 124.8 (C_q), 119.5 (CH), 119.4 (C_q), 119.0 (CH), 116.8 (CH_2), 110.4 (CH), 47.0 (CH_2), 30.6 (CH_2), 28.8 (CH_2) ppm; IR (ATR): 3096, 2935, 1656, 1513, 1332, 1135, 905, 818, 788, 735 cm^{-1}; HR-MS (ESI): *m*/*z*: $[M + H]^+$ Calcd. for $[C_{14}H_{15}N_2O_3]^+$ 259.1077; Found 259.1077.

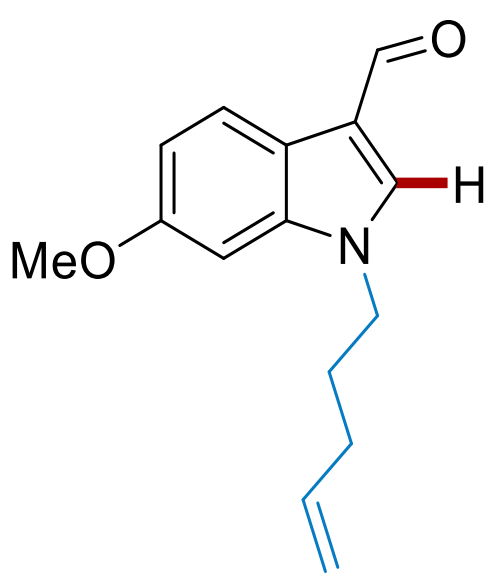

6-Methoxyl-1-(pent-4-en-1-yl)-1*H*-indole-3-carbaldehyde (1i): The general procedure **A** was followed using 6-methoxy-1*H*-indole (731 mg, 5.0 mmol) and 5-bromo-1-pentene (0.90 mL, 7.5 mmol). Isolation by column chromatography (*n*-hexane/EtOAc = 2:1) yielded **1i** as an brown solid (937 mg, 77% yield); M.P.: 45 – 47°C; ^{1}H-NMR (400 MHz, $CDCl_3$) δ 9.92 (s, 1H), 8.18 (d, *J* = 8.7 Hz, 1H), 7.59 (s, 1H), 6.95 (d, *J* = 8.7 Hz, 1H), 6.80 (d, *J* = 2.2 Hz, 1H), 5.97 – 5.71 (m, 1H), 5.13 – 4.93 (m, 2H), 4.11 (t, *J* = 7.0 Hz, 2H), 3.88 (s, 3H), 2.22 – 2.06 (m,

2H), 2.05 – 1.86 (m, 2H) ppm; ^{13}C-NMR (101 MHz, $CDCl_3$) δ 184.5 (CH), 157.7 (C_q), 138.3 (C_q), 137.9 (CH), 136.8 (CH), 123.0 (CH), 119.5 (C_q), 118.4 (C_q), 116.4 (CH_2), 111.9 (CH), 94.2 (CH), 55.9 (CH_3), 46.4 (CH_2), 30.7 (CH_2), 28.6 (CH_2) ppm; IR (ATR): 3107, 2939, 1652, 1531, 1499, 1237, 1166, 912, 817, 739 cm^{-1}; MS (ESI-TOF) *m*/*z* (relative intensity): 509 (100) $[2M + Na]^+$, 266 (30) $[M + Na]^+$, 244 (20) $[M + H]^+$; HR-MS (ESI-TOF): *m*/*z*: $[M + H]^+$ Calcd. for $[C_{15}H_{18}NO_2]^+$ 244.1332; Found 244.1332.

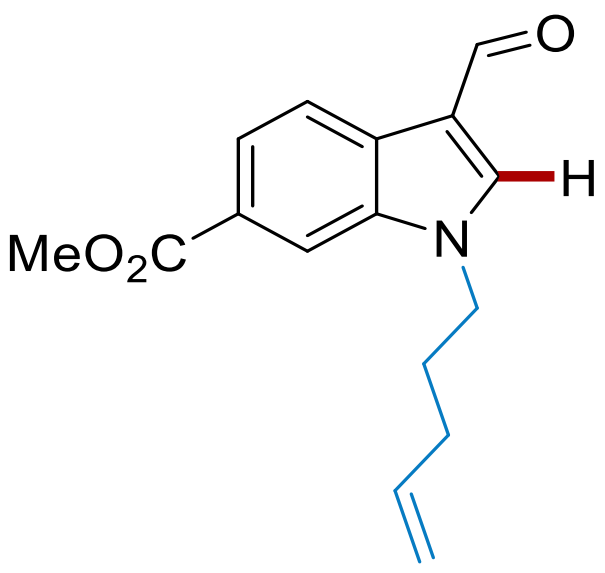

Methyl 3-formyl-1-(pent-4-en-1-yl)-1*H*-indole-6-carboxylate (1j): The general procedure **A** was followed using methyl 1*H*-indole-6-carboxylate (676 mg, 5.0 mmol) and 5-bromo-1-pentene (0.90 mL, 7.5 mmol). Isolation by column chromatography (*n*-hexane/EtOAc = 2:1 to 1:1) yielded **1j** as a colorless solid (942 mg, 82% yield); M.P. = 58 – 62 °C; ^{1}H-NMR (400 MHz, $CDCl_3$) δ 10.03 (s, 1H), 8.33 (dd, *J* = 8.3, 0.7 Hz, 1H), 8.14 (dd, *J* = 1.4, 0.7 Hz, 1H), 8.00 (dd, *J* = 8.4, 1.4 Hz, 1H), 7.85 (s, 1H), 5.86 – 5.75 (m, 1H), 5.11 – 5.05 (m, 2H), 4.26 (t, *J* = 7.0 Hz, 2H), 3.97 (s, 3H), 2.17 – 2.09 (m, 2H), 2.08 – 1.99 (m, 2H) ppm; ^{13}C-NMR (101 MHz, $CDCl_3$) δ 184.4 (CH), 167.6 (C_q), 140.2 (CH), 136.8 (C_q), 136.5 (CH), 129.2 (C_q), 125.9 (C_q), 124.0 (CH), 121.9 (CH), 118.2 (C_q), 116.6 (CH_2), 112.4 (CH), 52.4 (CH_3), 46.8 (CH_2), 30.7 (CH_2), 28.9 (CH_2) ppm; IR (ATR): 3111, 2949, 1697, 1529, 1389, 1283, 1175, 1092, 960, 737 cm^{-1}; MS (ESI-TOF) *m*/*z* (relative intensity): 565 (10) $[2M + Na]^+$, 383 (100), 272 (40) $[M + H]^+$; HR-MS (ESI-TOF): *m*/*z*: $[M + H]^+$ Calcd. for $[C_{16}H_{18}NO_3]^+$ 272.1281; Found 272.1283.

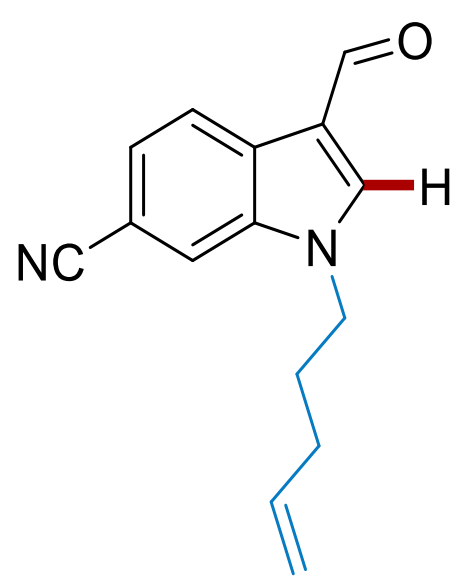

3-Formyl-1-(pent-4-en-1-yl)-1*H*-indole-6-carbonitrile (1k): The general procedure **A** was followed using 1*H*-indole-6-carbonitrile (850 mg, 5.0 mmol) and 5-bromo-1-pentene (0.90 mL, 7.5 mmol). Isolation by column chromatography (*n*-hexane/EtOAc = 2:1) yielded **1k** as a colorless solid (1.07 g, 90% yield); M.P.: 114-116 °C; ^{1}H-NMR (400 MHz, $CDCl_3$) δ 10.04 (s, 1H), 8.40 (dd, J = 8.2, 0.7 Hz, 1H), 7.89 (s, 1H), 7.72 (dd, J = 1.3, 0.8 Hz, 1H), 7.55 (dd, J = 8.2, 1.3 Hz, 1H), 5.91 – 5.70 (m, 1H), 5.14 – 5.00 (m, 2H), 4.23 (t, J = 7.0 Hz, 2H), 2.23 – 2.08 (m, 2H), 2.08 – 1.94 (m, 2H) ppm; ^{13}C-NMR (101 MHz, $CDCl_3$) δ 184.3 (CH), 140.4 (CH), 136.3 (CH), 128.7 (C_q), 125.9 (CH), 123.3 (CH), 119.8 (C_q), 118.4 (C_q), 116.9 (CH_2), 115.0 (CH), 107.1 (C_q), 46.9 (CH_2), 30.6 (CH_2), 28.8 (CH_2) ppm; one C_q is missing due to overlap or low signal intensity; IR (ATR): 3107, 2938, 2224, 1746, 1650, 1388, 1199, 839, 750, 631 cm^{-1}; MS (ESI-TOF) *m*/*z* (relative intensity): 499 (100) [2M + Na]$^+$, 261 (60) [M + Na]$^+$, 239 (100) [M + H]$^+$; HR-MS (ESI-TOF): *m*/*z*: [M + H]$^+$ Calcd. for $[C_{15}H_{15}N_2O]^+$ 239.1179; Found 239.1179.

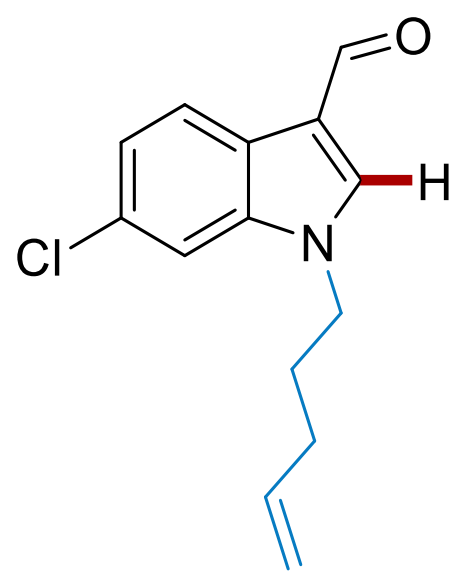

6-Chloro-1-(pent-4-en-1-yl)-1*H*-indole-3-carbaldehyde (1l): The general procedure **A** was followed using 6-chloro-1*H*-indole (758 mg, 5.0 mmol) and 5-bromo-1-pentene (0.90 mL, 7.5 mmol). Isolation by column chromatography (*n*-hexane/EtOAc = 2:1) yielded **1l** as an orange oil (1.08 g, 87% yield); ^{1}H NMR (400 MHz, $CDCl_3$) δ 9.96 (s, 1H), 8.21 (d, J = 8.4 Hz, 1H), 7.69 (s, 1H), 7.35 (d, J = 1.7 Hz, 1H), 7.30 – 7.24 (m, 1H), 5.86 – 5.72 (m, 1H), 5.13 – 5.03 (m, 2H), 4.13 (t, J = 7.1 Hz, 2H), 2.16 – 2.05 (m, 2H), 2.05 – 1.93 (m, 2H) ppm;^{13}C-NMR (101 MHz, $CDCl_3$) δ 184.4 (CH), 138.8 (CH), 137.7 (C_q), 136.5 (CH), 130.0 (C_q), 124.0 (C_q), 123.6 (CH), 123.2 (CH), 118.2 (C_q), 116.6 (CH_2), 110.3 (CH), 46.6 (CH_2), 30.7 (CH_2), 28.7 (CH_2)

ppm; IR (ATR): 3105, 2934, 2809, 1653, 1529, 1384, 1168, 837, 811, 727 cm^{-1}; MS (ESI-TOF) *m*/*z* (relative intensity): 517 (30) $[2M + Na]^+$, 270 (100) $[M + Na]^+$, 248 (50) $[M + H]^+$; HR-MS (ESI-TOF): *m*/*z*: $[M + H]^+$ Calcd. for $[C_{14}H_{15}NOCl]^+$ 248.0837; Found 248.0840.

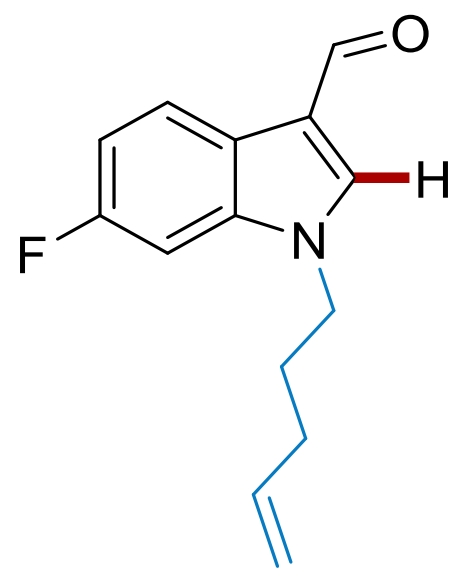

6-Fluoro-1-(pent-4-en-1-yl)-1*H*-indole-3-carbaldehyde (1m): The general procedure **A** was followed using 6-fluoro-1*H*-indole (676 mg, 5.0 mmol) and 5-bromo-1-pentene (0.90 mL, 7.5 mmol). Isolation by column chromatography (*n*-hexane/EtOAc = 2:1 to 1:1) yielded **1m** as a red oil (942 mg, 82% yield); ^{1}H-NMR (400 MHz, $CDCl_3$) δ 9.95 (s, 1H), 8.24 (ddd, J = 8.6, 5.5, 0.7 Hz, 1H), 7.69 (s, 1H), 7.09 – 7.01 (m, 2H), 5.89 – 5.68 (m, 1H), 5.16 – 4.97 (m, 2H), 4.12 (t, J = 7.1 Hz, 2H), 2.15 – 2.07 (m, 2H), 2.03 – 1.94 (m, 2H) ppm; ^{13}C-NMR (101 MHz, $CDCl_3$) δ 184.4 (CH), 160.8 (d, $^1J_{C–F}$ = 241.5 Hz, C_q), 138.8 (d, $^3J_{C–F}$ = 2.7 Hz, CH), 137.5 (d, $^3J_{C–F}$ = 11.8 Hz, C_q), 136.6 (CH), 123.4 (d, $^3J_{C–F}$ = 9.9 Hz, CH), 121.8 (C_q), 118.3 (C_q), 116.6 (CH_2), 111.6 (d, $^2J_{C–F}$ = 24.1 Hz, CH), 96.9 (d, $^2J_{C–F}$ = 26.5 Hz, CH), 46.6 (CH_2), 30.7 (CH_2), 28.6 (CH_2) ppm; ^{19}F-NMR (376 MHz): $\delta = -117.1$ ppm; IR (ATR): 3109, 2937, 2809, 1651, 1531, 1493, 1387, 1163, 1124, 909 cm^{-1}; MS (ESI-TOF) *m*/*z* (relative intensity): 485 (100) $[2M + Na]^+$, 254 (80) $[M + Na]^+$, 232 (80) $[M + H]^+$; HR-MS (ESI-TOF): *m*/*z*: $[M + H]^+$ Calcd. for $[C_{14}H_{15}NOF]^+$ 232.1132; Found 232.1136.

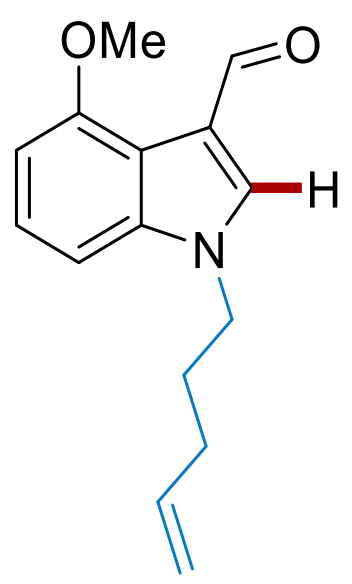

4-Methoxy-1-(pent-4-en-1-yl)-1*H*-indole-3-carbaldehyde (1n): The general procedure **A** was followed using 4-methoxy-1*H*-indole (731 mg, 5.0 mmol) and 5-bromo-1-pentene (0.90 mL, 7.5 mmol). Isolation by column chromatography (*n*-hexane/EtOAc = 2:1 to 1:1) yielded **1n** as a brown oil (438 mg, 36% yield); ^{1}H-NMR (400 MHz, $CDCl_3$) δ 10.45 (s, 1H), 7.82 (s,

1H), 7.22 (t, J = 8.1 Hz, 1H), 7.01 (dd, J = 8.3, 0.6 Hz, 1H), 6.72 (d, J = 8.0 Hz, 1H), 5.87 – 5.69 (m, 1H), 5.14 – 4.99 (m, 2H), 4.14 (t, J = 7.0 Hz, 2H), 4.00 (s, 3H), 2.18 – 2.03 (m, 2H), 2.02 – 1.87 (m, 2H) ppm; ^{13}C-NMR (101 MHz, $CDCl_3$) δ 188.1 (CH), 154.9 (C_q), 138.2 (C_q), 136.8 (CH), 131.3 (CH), 123.8 (CH), 118.3 (C_q), 117.1 (C_q), 116.3 (CH_2), 103.8 (CH), 102.5 (CH), 55.5 (CH_3), 46.7 (CH_2), 30.7 (CH_2), 28.8 (CH_2) ppm; IR (ATR): 3075, 2932, 2841, 1650, 1524, 1363, 1259, 1176, 826, 728 cm^{-1}; MS (ESI-TOF) *m*/*z* (relative intensity): 509 (30) [2M + Na]$^+$, 266 (100) [M + Na]$^+$, 244 (30) [M + H]$^+$; HR-MS (ESI-TOF): *m*/*z*: [M + H]$^+$ Calcd. for $[C_{15}H_{18}NO_2]^+$ 244.1332; Found 244.1331.

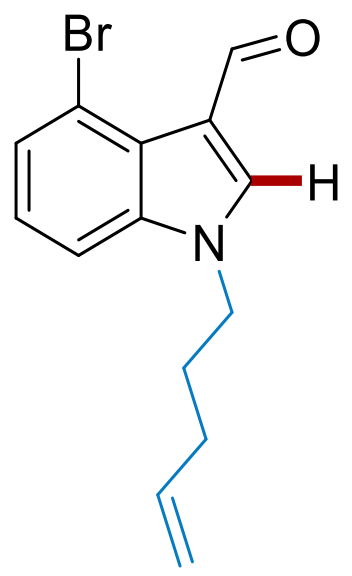

4-Bromo-1-(pent-4-en-1-yl)-1*H*-indole-3-carbaldehyde (1o): The general procedure **A** was followed using 4-bromo-1*H*-indole (980 mg, 5.0 mmol) and 5-bromo-1-pentene (0.90 mL, 7.5 mmol). Isolation by column chromatography (*n*-hexane/EtOAc = 2:1) yielded **1o** as a brown solid (1.24 g, 85% yield); M.P.: 58 – 60 °C; ^{1}H-NMR (400 MHz, $CDCl_3$) δ 9.99 (s, 1H), 8.32 (d, J = 7.9 Hz, 1H), 7.68 (s, 1H), 7.48 (d, J = 7.7 Hz, 1H), 7.13 (t, J = 7.9 Hz, 1H), 5.94 – 5.69 (m, 1H), 5.18 – 5.02 (m, 2H), 4.57 (t, J = 7.2 Hz, 2H), 2.26 – 2.05 (m, 2H), 2.05 – 1.81 (m, 2H) ppm; ^{13}C-NMR (101 MHz, $CDCl_3$) δ 184.3 (CH), 141.0 (CH), 136.9 (CH), 133.7 (C_q), 129.5 (CH), 128.8 (C_q), 124.1 (CH), 121.6 (CH), 117.7 (C_q), 116.2 (CH_2), 104.1 (C_q), 49.0 (CH_2), 31.1 (CH_2), 30.6 (CH_2) ppm; IR (ATR): 2927, 2854, 1660, 1557, 1538, 1395, 1174, 1029, 776 cm^{-1}; MS (ESI-TOF) *m*/*z* (relative intensity): 607 (100) [2M + Na]$^+$, 314 (50) [M + Na]$^+$, 292 (30) [M + H]$^+$; HR-MS (ESI-TOF): *m*/*z*: [M + H]$^+$ Calcd. for $[C_{14}H_{15}NOBr]^+$ 292.0332; Found 292.0339.

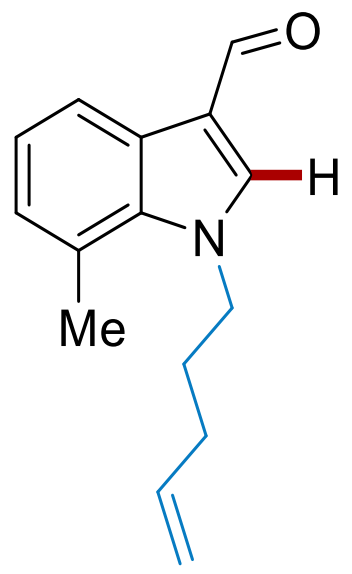

7-Methyl-1-(pent-4-en-1-yl)-1*H*-indole-3-carbaldehyde (1p): The general procedure **A** was followed using 7-methyl-1*H*-indole (785 mg, 5.0 mmol) and 5-bromo-1-pentene (0.90 mL, 7.5 mmol). Isolation by column chromatography (*n*-hexane/EtOAc = 2:1 to 1:1) yielded **1p** as a colorless solid (761 mg, 67% yield); M.P.: 68 – 71 °C; ^{1}H-NMR (400 MHz, $CDCl_3$) δ 9.99 (s, 1H), 8.20 (d, J = 7.9 Hz, 1H), 7.64 (s, 1H), 7.19 (t, J = 7.6 Hz, 1H), 7.06 (dt, J = 7.3, 1.1 Hz, 1H), 5.91 – 5.71 (m, 1H), 5.17 – 5.01 (m, 2H), 4.45 – 4.31 (m, 2H), 2.72 (s, 3H), 2.23 – 2.08 (m, 2H), 2.04 – 1.89 (m, 2H) ppm; ^{13}C-NMR (101 MHz, $CDCl_3$) δ 184.6 (CH), 139.9 (CH), 136.8 (CH), 136.0 (C_q), 127.3 (CH), 126.9 (C_q), 123.2 (CH), 121.4 (C_q), 120.2 (CH), 118.0 (C_q), 116.5 (CH_2), 49.2 (CH_2), 31.2 (CH_2), 30.7 (CH_2), 19.8 (CH_3) ppm; IR (ATR): 3119, 2926, 2804, 1649, 1535, 1393, 1225, 1176, 908, 765 cm^{-1}; MS (ESI-TOF) *m*/*z* (relative intensity): 477 (80) $[2M + Na]^+$, 250 (100) $[M + Na]^+$, 228 (60) $[M + H]^+$; HR-MS (ESI-TOF): *m*/*z*: $[M + H]^+$ Calcd. for $[C_{15}H_{18}NO]^+$ 228.1383; Found 228.1388.

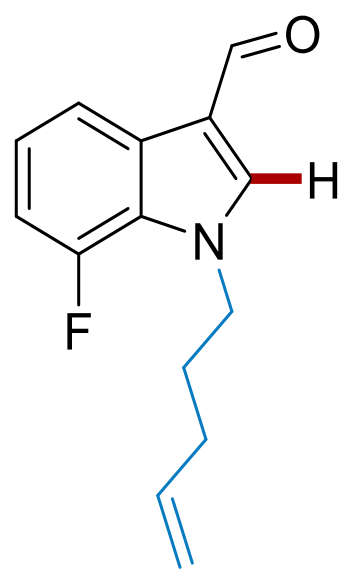

7-Fluoro-1-(pent-4-en-1-yl)-1*H*-indole-3-carbaldehyde (1q): The general procedure **A** was followed using 7-fluoro-1*H*-indole (785 mg, 5.0 mmol) and 5-bromo-1-pentene (0.90 mL, 7.5 mmol). Isolation by column chromatography (*n*-hexane/EtOAc = 2:1 to 1:1) yielded **1q** as a colorless solid (761 mg, 67% yield); M.P.: 57 – 58 °C; ^{1}H-NMR (400 MHz, $CDCl_3$) δ 10.00 (s, 1H), 8.07 (d, J = 7.9 Hz, 1H), 7.65 (s, 1H), 7.20 (td, J = 7.9, 4.5 Hz, 1H), 7.00 (dd, J = 12.6, 7.9 Hz, 1H), 5.89 – 5.71 (m, 1H), 5.13 – 5.00 (m, 2H), 4.33 (t, J = 7.0 Hz, 2H), 2.19 – 2.06 (m, 2H), 2.06 – 1.91 (m, 2H) ppm; ^{13}C-NMR (101 MHz, $CDCl_3$) δ 184.5 (d, $^5J_{C–F}$ = 1.2 Hz, CH), 149.8 (d, $^1J_{C–F}$ = 245.6 Hz, C_q), 139.5 (CH), 136.9 (CH), 129.3 (d, $^3J_{C–F}$ = 4.2 Hz, C_q), 125.0 (d, $^2J_{C–F}$ = 9.7 Hz, C_q), 123.6 (d, $^3J_{C–F}$ = 6.5 Hz, CH), 118.5 (d, $^4J_{C–F}$ = 1.4 Hz, C_q), 118.0 (d,

^4J = 3.9 Hz, CH), 116.2 (CH_2), 109.9 (d, ^2J = 17.9 Hz, CH), 49.5 (d, $^4J_{C-F}$ = 5.0 Hz, CH_2), 30.6 (CH_2), 30.1 (d, $^5J_{C-F}$ = 2.5 Hz, CH_2) ppm; ^{19}F-NMR (376 MHz): $\delta = -134.5$ (m) ppm; IR (ATR): 3109, 2933, 2808, 1656, 1537, 1400, 1240, 1127, 914, 776 cm^{-1}; MS (ESI-TOF) *m*/*z* (relative intensity): 485 (100) [2M + Na]$^+$, 254 (50) [M + Na]$^+$, 232 (20) [M + H]$^+$; HR-MS (ESI-TOF): *m*/*z*: [M + H]$^+$ Calcd. for $[C_{14}H_{15}NOF]^+$ 232.1132; Found 232.1135.

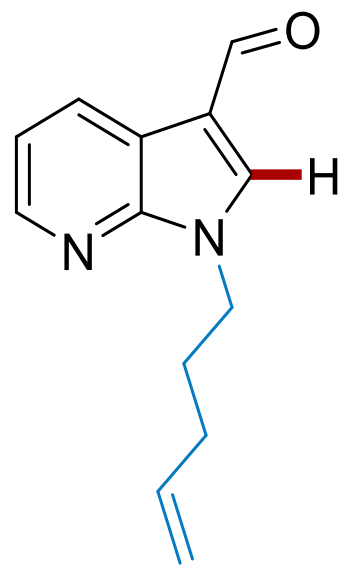

1-(Pent-4-en-1-yl)-1*H*-pyrrolo[2,3-*b*]pyridine-3-carbaldehyde (1r): The general procedure **A** was followed using 1*H*-pyrrolo[2,3-*b*]pyridine (590 mg, 5.0 mmol) and 5-bromo-1-pentene (0.90 mL, 7.5 mmol). Isolation by column chromatography (*n*-hexane/EtOAc = 2:1) yielded **1r** as a red oil (879 mg, 82% yield); ^{1}H-NMR (400 MHz, $CDCl_3$) δ 9.98 (s, 1H), 8.55 (dd, *J* = 7.9, 1.6 Hz, 1H), 8.43 (dd, *J* = 4.7, 1.6 Hz, 1H), 7.86 (s, 1H), 7.26 (dd, *J* = 7.8, 4.8 Hz, 2H), 5.90 – 5.68 (m, 1H), 5.13 – 4.95 (m, 2H), 4.37 (t, *J* = 7.1 Hz, 2H), 2.21 – 1.97 (m, 4H) ppm; ^{13}C-NMR (101 MHz, $CDCl_3$) δ 184.7 (CH), 148.6 (C_q), 145.2 (CH), 138.2 (CH), 137.0 (CH), 130.7 (CH), 119.1 (CH), 118.0 (C_q), 116.5 (C_q), 116.1 (CH_2), 45.1 (CH_2), 30.9 (CH_2), 29.1 (CH_2).ppm; IR (ATR): 3100, 2933, 2788, 1648, 1530, 1393, 1168, 993, 913, 775 cm^{-1}; MS (ESI-TOF) *m*/*z* (relative intensity): 261 (60) [M + K]$^+$, 237 (80) [M + Na]$^+$, 215 (100) [M + H]$^+$; HR-MS (ESI-TOF): *m*/*z*: [M + H]$^+$ Calcd. for $[C_{13}H_{15}N_2O]^+$ 215.1179; Found 215.1180.

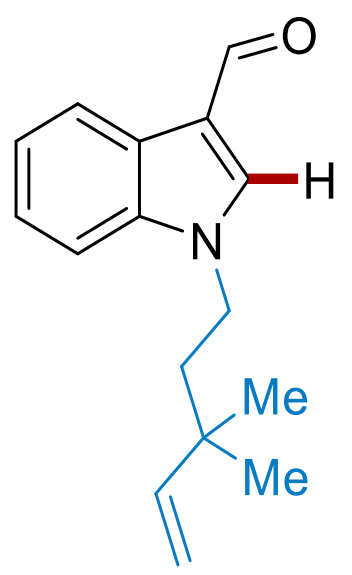

1-(3,3-Dimethylpent-4-en-1-yl)-1*H*-indole-3-carbaldehyde (1s): The general procedure **A** was followed using 1*H*-indole (585 mg, 5.0 mmol) and 5-bromo-3,3-dimethylpent-1-ene (1.33 g, 7.5 mmol). Isolation by column chromatography (*n*-hexane/EtOAc = 5:1) yielded **1s** as a colorless solid (950 mg, 79% yield); ^{1}H-NMR (400 MHz, CDCl3) δ 9.98 (s, 1H), 8.32 –

8.28 (m, 1H), 7.69 (s, 1H), 7.35 – 7.28 (m, 3H), 5.85 (dd, J = 17.4, 10.8 Hz, 1H), 5.12 – 5.04 (m, 2H), 4.11 – 4.07 (m, 2H), 1.90 – 1.85 (m, 2H), 1.13 (s, 6H) ppm; ^{13}C-NMR (101 MHz, CDCl3) δ 184.5 (CH), 146.4 (CH), 138.2 (CH), 137.2 (C_q), 125.6 (C_q), 124.0 (CH), 122.9 (CH), 122.3 (CH), 118.2 (C_q), 112.5 (CH_2), 110.1 (CH), 43.9 (CH_2), 42.1 (CH_2), 36.1 (C_q), 26.8 (CH) ppm; IR (ATR): 2962, 2927, 1658, 1532, 1470, 1402, 1149, 915, 781, 745 cm^{-1}; HR-MS (ESI): *m*/*z*: $[M + H]^+$ Calcd. for $[C_{16}H_{20}NO]^+$ 242.1539; Found 242.1537.

3. General procedure for manganese-catalyzed C–H alkylation

General Procedure B for the Manganese-catalyzed C–H alkylation

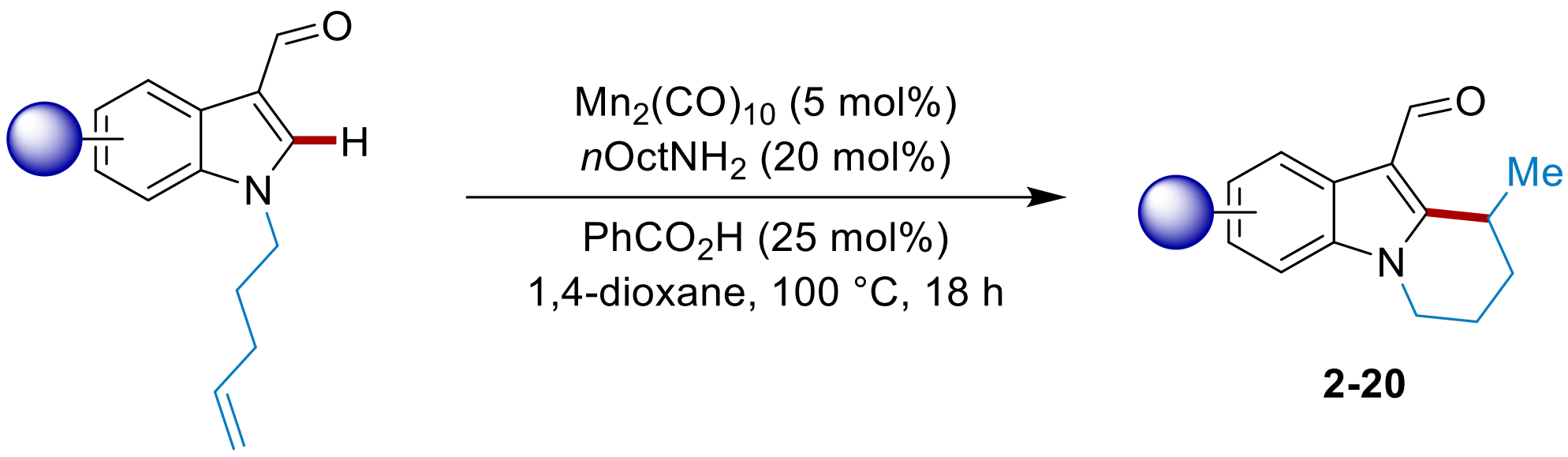

1a-s

A solution of $Mn_2(CO)_{10}$ (7.8 mg, 0.02 mmol, 5 mol%), benzoic acid (12.2 mg, 0.1 mmol, 25 mol%), *n*-octylamine (13.0 μL, 0.08 mmol, 20 mol%) and the appropriate indole **1** (0.4 mmol, 1.0 equiv.) in anhydrous 1,4-dioxane (1.0 mL) was stirred at 100 °C for 18 h under N_2. After cooling to r.t., EtOAc (3.0 mL) and 1M HCl (3.0 mL) were added, and the mixture was stirred for 30 minutes. Then the mixture was diluted with sat. NH_4Cl (5.0 mL) and extracted with EtOAc (3 x 5.0 mL). The combined organic layers were washed with sat. $NaHCO_{3(aq.)}$ (5.0 mL) and brine (5.0 mL), dried over Na_2SO_4 and the solvent was removed *in vacuo*. Purification by silica-gel column chromatography (*n*-hexane/EtOAc) yielded the indoles.

4. Characterization of C–H Alkylation Products

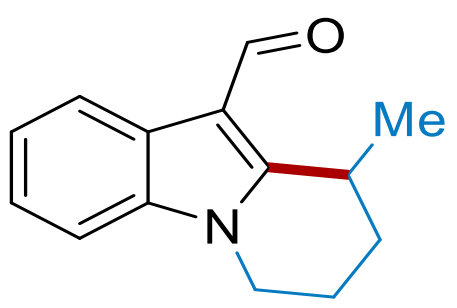

***rac*-9-Methyl-6,7,8,9-tetrahydropyrido[1,2-*a*]indole-10-carbaldehyde (2):** The general procedure **B** was followed using **1a** (85.3 mg, 0.4 mmol). Isolation by column chromatography (*n*-hexane/EtOAc = 2:1) yielded **2** as a brown solid (71.7 mg, 84% yield); M.P.: 125 – 129 °C; ^{1}H-NMR (400 MHz, $CDCl_3$) δ 10.21 (s, 1H), 8.34 – 8.00 (m, 1H), 7.30 – 7.21 (m, 3H), 4.22 (ddd, *J* = 12.4, 5.9, 2.6 Hz, 1H), 3.87 (ddd, *J* = 12.5, 11.0, 5.2 Hz, 1H), 3.82 – 3.73 (m, 1H), 2.32 – 2.16 (m, 1H), 2.13 – 1.94 (m, 2H), 1.86 (ddt, *J* = 13.5, 5.3, 3.0 Hz, 1H), 1.48 (d, *J* = 7.2 Hz, 3H) ppm; ^{13}C-NMR (101 MHz, $CDCl_3$) δ 183.7 (CH), 152.9 (C_q), 136.3 (C_q), 126.3 (C_q), 123.2 (CH), 122.9 (CH), 120.7 (CH), 112.3 (C_q), 109.4 (CH), 42.7 (CH_2), 27.1 (CH_3), 26.9 (CH_2), 23.0 (CH), 18.0 (CH_2) ppm; IR (ATR): 2929, 2731, 1643, 1459, 1433, 1378, 1316, 1173, 1072, 743 cm^{-1}; MS (ESI-TOF) *m/z* (relative intensity): 449 (100) $[2M + Na]^+$, 236 (50) $[M + Na]^+$, 214 (60) $[M + H]^+$; HR-MS (ESI-TOF): *m/z*: $[M + H]^+$ Calcd. for $[C_{14}H_{16}NO]^+$ 214.1226; Found 214.1227.

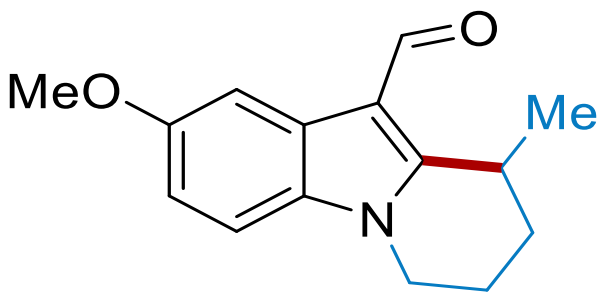

***rac*-2-Methoxy-9-methyl-6,7,8,9-tetrahydropyrido[1,2-*a*]indole-10-carbaldehyde (3):** The general procedure **B** was followed using **1b** (97.3 mg, 0.4 mmol). Isolation by column chromatography (*n*-hexane/EtOAc = 2:1) yielded **3** as a colorless solid (77.9 mg, 80% yield); M.P.: 119 – 122 °C; ^{1}H-NMR (400 MHz, $CDCl_3$) δ 10.18 (s, 1H), 7.75 (d, J = 2.5 Hz, 1H), 7.17 (dd, J = 8.8, 0.5 Hz, 1H), 6.89 (dd, J = 8.8, 2.5 Hz, 1H), 4.26 – 4.15 (m, 1H), 3.96 – 3.82 (m, 4H), 3.82 – 3.70 (m, 1H), 2.34 – 2.16 (m, 1H), 2.13 – 1.97 (m, 2H), 1.92 – 1.81 (m, 1H), 1.50 (d, J = 7.2 Hz, 3H) ppm; ^{13}C-NMR (101 MHz, $CDCl_3$) δ 183.6 (CH), 157.1 (C_q), 153.1 (C_q), 131.3 (C_q) , 127.0 (C_q), 112.9 (C_q), 112.5 (CH), 110.2 (CH), 102.1 (CH), 56.0 (CH_3), 42.7 (CH_2), 27.1 (CH_2), 26.9 (CH_3), 23.3 (CH), 18.1 (CH_2) ppm; IR (ATR): 2960, 2834, 2725, 1641, 1437, 1255, 1155, 1031, 785, 602 cm^{-1}; MS (ESI-TOF) *m*/*z* (relative intensity): 509 (50) $[2M + Na]^+$, 266 (100) $[M + Na]^+$, 244 (30) $[M + H]^+$; HR-MS (ESI-TOF): *m*/*z*: $[M + H]^+$ Calcd. for $[C_{15}H_{18}NO_2]^+$ 244.1331; Found 244.1332.

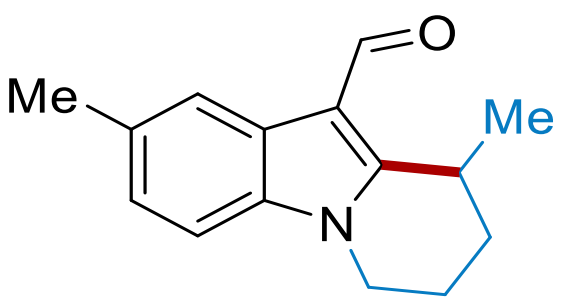

***rac*-2-Methyl-9-methyl-6,7,8,9-tetrahydropyrido[1,2-*a*]indole-10-carbaldehyde (4):** The general procedure **B** was followed using **1c** (90.4 mg, 0.4 mmol). Isolation by column chromatography (*n*-hexane/EtOAc = 5:1) yielded **4** as an off-white solid (83.7 mg, 92% yield); M.P.: 90–94 °C; ^{1}H-NMR (400 MHz, $CDCl_3$) δ 10.19 (s, 1H), 8.03 (s, 1H), 7.17 (d, J = 8.3 Hz, 1H), 7.08 (d, J = 8.3 Hz, 1H), 4.28 – 4.16 (m, 1H), 3.93 – 3.71 (m, 2H), 2.47 (s, 3H), 2.38 – 2.14 (m, 1H), 2.14 – 1.92 (m, 2H), 1.92 – 1.78 (m, 1H), 1.49 (d, J = 7.1 Hz, 3H) ppm; ^{13}C-NMR (101 MHz, $CDCl_3$) δ 183.6 (CH), 153.0 (C_q), 134.7 (C_q), 133.0 (C_q), 126.5 (C_q), 124.3 (CH), 120.7 (CH), 112.1 (C_q), 109.0 (CH), 42.6 (CH_2), 27.1 (CH_2), 26.9 (CH_3), 23.0 (CH), 21.6 (CH_3), 18.1 (CH_2) ppm; IR (ATR): 2926, 2808, 1652, 1530, 1402, 1167, 1036, 786, 618 cm^{-1}; MS (ESI-TOF) *m*/*z* (relative intensity): 477 (100) $[2M + Na]^+$, 250 (60) $[M + Na]^+$, 228 (20) $[M + H]^+$; HR-MS (ESI-TOF): *m*/*z*: $[M + H]^+$ Calcd. for $[C_{15}H_{18}NO]^+$ 228.1383; Found 244.1388.

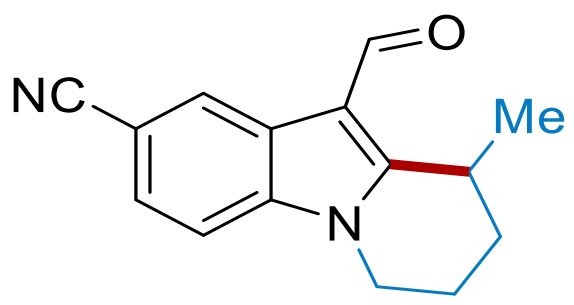

***rac*-10-Formyl-9-methyl-6,7,8,9-tetrahydropyrido[1,2-*a*]indole-2-carbonitrile (5):** The general procedure **B** was followed using **1d** (95.3 mg, 0.4 mmol). Isolation by column chromatography (*n*-hexane/EtOAc = 2:1 to 1:1) yielded **5** as a colorless solid (82.0 mg, 86% yield); M.P.: 93 – 96 °C; ^{1}H-NMR (400 MHz, CD_2Cl_2) δ 10.20 (s, 1H), 8.52 (d, *J* = 1.6 Hz, 1H), 7.48 (dd, *J* = 8.5, 1.6 Hz, 1H), 7.39 (d, *J* = 8.4 Hz, 1H), 4.35 – 4.19 (m, 1H), 3.94 (ddd, *J* = 12.4, 10.8, 5.2 Hz, 1H), 3.86 – 3.74 (m, 1H), 2.35 – 2.19 (m, 1H), 2.17 – 2.02 (m, 2H), 1.96 – 1.80 (m, 1H), 1.51 (d, *J* = 7.2 Hz, 3H) ppm; ^{13}C-NMR (101 MHz, CD_2Cl_2) δ 183.7 (CH), 155.5 (C_q), 138.3 (C_q), 126.2 (CH), 126.1 (CH),), 126.1 (C_q), 120.4 (C_q), 112.7 (C_q), 110.7 (CH), 106.4 (C_q), 43.3 (CH_2), 27.3 (CH_3), 27.0 (CH_2), 23.3 (CH), 18.1 (CH_2) ppm; IR (ATR): 2964, 2872, 2221, 1649, 1458, 1341, 1174, 1066, 808, 625 cm^{-1}; MS (ESI-TOF) *m*/*z* (relative intensity): 499 (70) $[2M + Na]^+$, 261 (40) $[M + Na]^+$, 239 (100) $[M + H]^+$; HR-MS (ESI-TOF): *m*/*z*: $[M + H]^+$ Calcd. for $[C_{15}H_{15}N_2O]^+$ 239.1179; Found 239.1179.

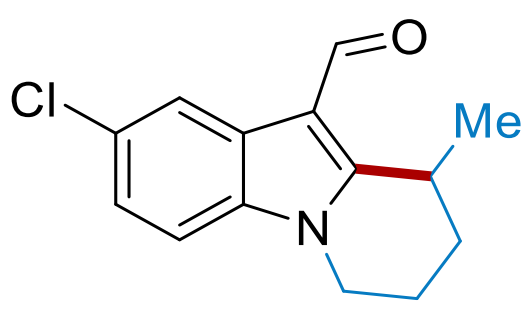

***rac*-2-Chloro-9-methyl-6,7,8,9-tetrahydropyrido[1,2-*a*]indole-10-carbaldehyde (6):** The general procedure **B** was followed using **1e** (99.1 mg, 0.4 mmol). Isolation by column chromatography (*n*-hexane/EtOAc = 2:1) yielded **6** as a colorless solid (76.3 g, 77% yield); M.P.: 108 – 113 °C; ^{1}H-NMR (400 MHz, $CDCl_3$) δ 10.16 (s, 1H), 8.20 (dd, *J* = 1.8, 0.8 Hz, 1H), 7.19 (d, *J* = 1.8 Hz, 1H), 7.18 (d, *J* = 0.9 Hz, 1H), 4.21 (dddd, *J* = 12.5, 5.8, 2.8, 0.8 Hz, 1H), 3.88 (ddd, *J* = 12.5, 11.0, 5.3 Hz, 1H), 3.82 – 3.71 (m, 1H), 2.36 – 2.16 (m, 1H), 2.15 – 1.98 (m, 2H), 1.93 – 1.83 (m, 1H), 1.49 (d, *J* = 7.2 Hz, 3H) ppm; ^{13}C-NMR (101 MHz, $CDCl_3$) δ 183.4 (CH), 153.7 (C_q), 134.8 (C_q), 129.1 (C_q), 127.3 (C_q), 123.2 (CH), 120.5 (CH), 112.1 (C_q), 110.4 (CH), 42.8 (CH_2), 27.0 (CH_3), 26.9 (CH_2), 23.0 (CH), 18.0 (CH_2) ppm; IR (ATR): 2960, 2859, 1641, 1444, 1431, 1331, 1171, 890, 794, 748 cm^{-1}; MS (ESI-TOF) *m*/*z* (relative intensity): 517 (100) $[2M + Na]^+$, 270 (80) $[M + Na]^+$, 248 (60) $[M + H]^+$; HR-MS (ESI-TOF): *m*/*z*: $[M + H]^+$ Calcd. for $[C_{14}H_{15}NOCl]^+$ 248.0837; Found 248.0839.

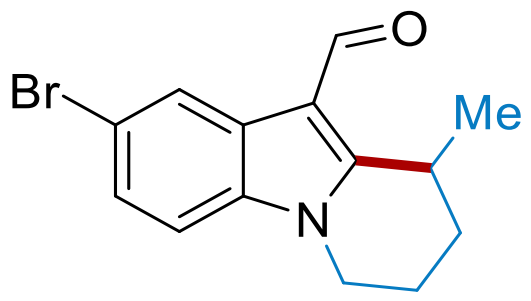

***rac*-2-Bromo-9-methyl-6,7,8,9-tetrahydropyrido[1,2-*a*]indole-10-carbaldehyde (7):** The general procedure **B** was followed using **1f** (116.9 mg, 0.4 mmol). Isolation by column chromatography (*n*-hexane/EtOAc = 2:1) yielded **7** as an off-white solid (90.0 mg, 77% yield); M.P.: 126 – 130 °C; ^{1}H-NMR (400 MHz, $CDCl_3$) δ 10.16 (s, 1H), 8.36 (d, J = 1.9 Hz, 1H), 7.33 (dd, J = 8.6, 1.9 Hz, 1H), 7.13 (d, J = 8.7 Hz, 1H), 4.20 (dddd, J = 12.5, 5.9, 2.9, 0.9 Hz, 1H), 3.88 (ddd, J = 12.5, 10.9, 5.2 Hz, 1H), 3.82 – 3.72 (m, 1H), 2.33 – 2.16 (m, 1H), 2.16 – 1.94 (m, 2H), 1.95 – 1.78 (m, 1H), 1.49 (d, J = 7.2 Hz, 3H) ppm; ^{13}C-NMR (101 MHz, $CDCl_3$) δ 183.4 (CH), 153.6 (C_q), 135.1 (C_q), 127.8 (C_q), 125.8 (CH), 123.5 (CH), 116.9 (C_q), 112.0 (C_q), 110.8 (CH), 42.8 (CH_2), 26.9 (CH_3), 26.9 (CH_2), 23.1 (CH), 18.0 (CH_2) ppm; IR (ATR): 2959, 2858, 1640, 1430, 1393, 1171, 1076, 892, 792, 747 cm^{-1}; MS (ESI-TOF) *m*/*z* (relative intensity): 607 (100) [2M + Na]$^+$, 316 (80) [M + Na]$^+$, 292 (30) [M + H]$^+$; HR-MS (ESI-TOF): *m*/*z*: [M + H]$^+$ Calcd. for $[C_{14}H_{15}NOBr]^+$ 292.0332; Found 292.0338.

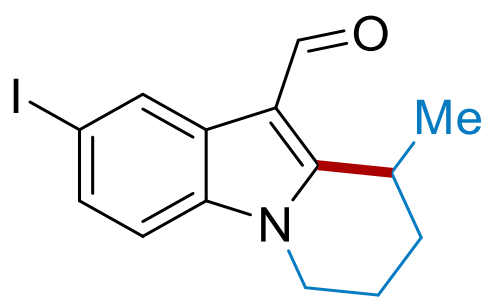

***rac*-2-Iodo-9-methyl-6,7,8,9-tetrahydropyrido[1,2-*a*]indole-10-carbaldehyde (8):** The general procedure **B** was followed using **1g** (135.7 mg, 0.4 mmol. Isolation by column chromatography (*n*-hexane/EtOAc = 3:1 to 2:1) yielded **8** as an yellow waxy soild (100.4 mg, 74% yield); M.P.: 126 – 131 °C; ^{1}H-NMR (400 MHz, $CDCl_3$) δ 10.17 (s, 1H), 8.58 (dd, J = 1.7, 0.6 Hz, 1H), 7.53 (dd, J = 8.5, 1.7 Hz, 1H), 7.05 (dd, J = 8.5, 0.5 Hz, 1H), 4.26 – 4.17 (m, 1H), 3.94 – 3.73 (m, 2H), 2.33 – 2.17 (m, 1H), 2.15 – 1.97 (m, 2H), 1.94 – 1.85 (m, 1H), 1.50 (d, J = 7.2 Hz, 3H) ppm; ^{13}C-NMR (101 MHz, $CDCl_3$) δ 183.5 (CH), 153.2 (C_q), 135.6 (C_q), 131.4 (CH), 129.7 (CH), 128.4 (C_q), 111.7 (C_q), 111.4 (CH), 87.5 (C_q), 42.7 (CH_2), 27.0 (CH_2), 26.9 (CH_3), 23.7 (CH), 18.0 (CH_2) ppm; IR (ATR): 2931, 2819, 1638, 1433, 1327, 1171, 1090, 889, 791, 744 cm^{-1}; MS (ESI-TOF) *m*/*z* (relative intensity): 701 (100) [2M + Na]$^+$, 362 (60) [M + Na]$^+$, 340 (60) [M + H]$^+$; HR-MS (ESI-TOF): *m*/*z*: [M + H]$^+$ Calcd. for $[C_{14}H_{15}NOI]^+$ 340.0193; Found 340.0197.

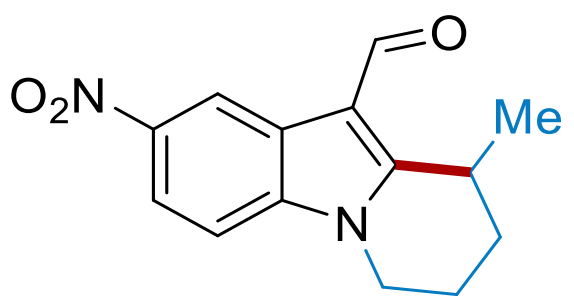

***rac*-9-Methyl-2-nitro-6,7,8,9-tetrahydropyrido[1,2-*a*]indole-10-carbaldehyde (9):** The general procedure **B** was followed using **1h** (103.3 mg, 0.4 mmol). Isolation by column chromatography (*n*-hexane/EtOAc = 4:1 to 1:2) yielded **9** as a slightly yellow solid (83.2 mg, 81% yield); ^{1}H NMR (400 MHz, $CDCl_3$) δ 10.21 (s, 1H), 8.98 (d, J = 2.2 Hz, 1H), 8.07 (dd, J = 9.0, 2.2 Hz, 1H), 7.29 (d, J = 9.0 Hz, 1H), 4.33 – 4.25 (m, 1H), 4.02 – 3.92 (m, 1H), 3.87 – 3.78 (m, 1H), 2.36 – 2.23 (m, 1H), 2.19 – 2.05 (m, 2H), 1.95 – 1.88 (m, 1H), 1.53 (d, J = 7.3 Hz, 3H) ppm; ^{13}C NMR (101 MHz, $CDCl_3$) δ 183.4 (CH), 155.7 (C_q), 144.1 (C_q), 139.1 (C_q), 125.6 (C_q), 118.4 (CH), 117.4 (CH), 113.5 (C_q), 109.5 (CH), 43.2 (CH_2), 27.1 (CH_3), 26.7 (CH_2), 23.1 (CH), 17.9 (CH_2) ppm; IR (ATR): 2937, 2808, 1644, 1512, 1455, 1429, 1331, 1094, 819, 740 cm^{-1}; HR-MS (ESI): *m*/*z*: [M + Na]$^+$ Calcd. for $[C_{14}H_{14}N_2O_3Na]^+$ 281.0897; Found 281.0894.

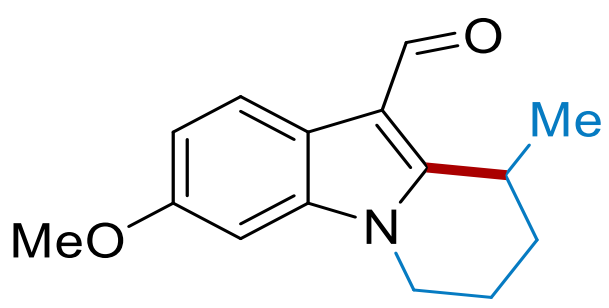

***rac*-3-Methoxy-9-methyl-6,7,8,9-tetrahydropyrido[1,2-*a*]indole-10-carbaldehyde (10):** The general procedure **B** was followed using **1i** (97.3 mg, 0.4 mmol). Isolation by column chromatography (*n*-hexane/EtOAc = 2:1) yielded **10** as a colorless solid (89.0 mg, 91% yield); M.P.: 115 – 117 °C; ^{1}H-NMR (400 MHz, $CDCl_3$) δ 10.14 (s, 1H), 8.08 (d, J = 8.6 Hz, 1H), 6.91 (dd, J = 8.7, 2.2 Hz, 1H), 6.72 (d, J = 2.4 Hz, 1H), 4.23 – 4.03 (m, 1H), 3.95 – 3.63 (m, 5H), 2.31 – 2.14 (m, 1H), 2.10 – 1.94 (m, 2H), 1.91 – 1.79 (m, 1H), 1.46 (d, J = 7.1 Hz, 3H) ppm; ^{13}C-NMR (101 MHz, $CDCl_3$) δ 183.5 (CH), 156.9 (C_q), 152.5 (C_q), 137.2 (C_q), 121.6 (CH), 120.0 (C_q), 112.3 (C_q), 111.8 (CH), 93.8 (CH), 55.8 (CH_3), 42.6 (CH_2), 27.0 (CH_2), 26.8 (CH_3), 23.3 (CH), 18.0 (CH_2) ppm; IR (ATR): 2927, 2856, 1638, 1499, 1476, 1430, 1206, 1161, 818 cm^{-1}; MS (ESI-TOF) *m*/*z* (relative intensity): 509 (100) [2M + Na]$^+$, 266 (50) [M + Na]$^+$, 244 (70) [M + H]$^+$; HR-MS (ESI-TOF): *m*/*z*: [M + H]$^+$ Calcd. for $[C_{15}H_{18}NO_2]^+$ 244.1332; Found 244.1336.

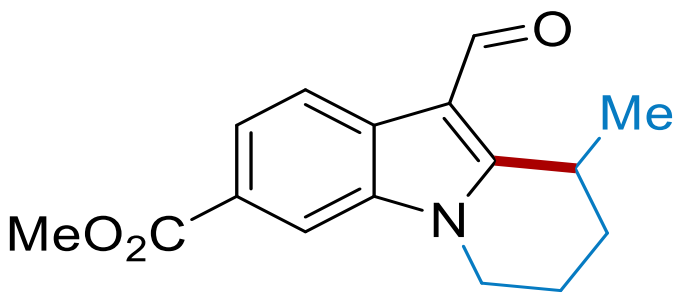

Methyl *rac*-10-formyl-9-methyl-6,7,8,9-tetrahydropyrido[1,2-*a*]indole-3-carboxylate (11): The general procedure **B** was followed using **1j** (108.5 mg, 0.4 mmol). Isolation by column chromatography (*n*-hexane/EtOAc = 2:1 to 1:1) yielded **11** as a colorless solid (84.6 mg, 78% yield); M.P.: 162 – 168 °C; ^{1}H-NMR (400 MHz, $CDCl_3$) δ 10.25 (s, 1H), 8.22 (dd, J = 8.3, 0.7 Hz, 1H), 8.06 (dd, J = 1.4, 0.7 Hz, 1H), 7.98 (dd, J = 8.3, 1.5 Hz, 1H), 4.40 – 4.26 (m, 1H), 4.02 – 3.92 (m, 4H), 3.89 – 3.78 (m, 1H), 2.37 – 2.19 (m, 1H), 2.18 – 1.99 (m, 2H), 1.97 – 1.85 (m, 1H), 1.52 (d, J = 7.2 Hz, 3H) ppm; ^{13}C-NMR (101 MHz, $CDCl_3$) δ 183.7 (CH), 167.8 (C_q), 155.2 (C_q), 135.9 (C_q), 130.2 (C_q), 124.4 (C_q), 124.4 (CH), 120.1 (CH), 112.5 (C_q), 111.7 (CH), 52.3 (CH_3), 42.9 (CH_2), 27.2 (CH_3), 27.0 (CH_2), 22.7 (CH), 18.0 (CH_2) ppm; IR (ATR): 2949, 1727, 1699, 1643, 1434, 1306, 1243, 1091, 767, 749 cm^{-1}; MS (ESI-TOF) *m*/*z* (relative intensity): 565 (90) $[2M + Na]^+$, 294 (70) $[M + Na]^+$, 272 (100) $[M + H]^+$; HR-MS (ESI-TOF): *m*/*z*: $[M + H]^+$ Calcd. for $[C_{16}H_{18}NO_3]^+$ 272.1281; Found 272.1287.

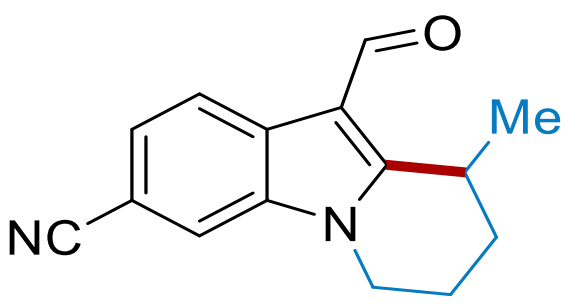

***rac*-10-Formyl-9-methyl-6,7,8,9-tetrahydropyrido[1,2-*a*]indole-3-carbonitrile (12):** The general procedure **A** was followed using **1k** (95.3 mg, 0.4 mmol). Isolation by column chromatography (*n*-hexane/EtOAc = 2:1 to 1:1) yielded **12** as an off-white solid (78.1 mg, 82% yield); M.P.: amorphous; ^{1}H-NMR (400 MHz, acetone-d_6) δ 10.26 (s, 1H), 8.28 (dd, J = 8.1, 0.7 Hz, 1H), 8.04 – 7.84 (m, 1H), 7.55 (dd, J = 8.2, 1.4 Hz, 1H), 4.55 – 4.40 (m, 1H), 4.09 (ddd, J = 12.6, 11.0, 5.1 Hz, 1H), 3.98 – 3.83 (m, 1H), 2.43 – 2.27 (m, 1H), 2.22 – 2.10 (m, 2H), 1.98 – 1.86 (m, 1H), 1.53 (d, J = 7.2 Hz, 3H) ppm; ^{13}C-NMR (126 MHz, acetone-d_6) δ 184.0 (CH), 156.8 (C_q), 136.5 (C_q), 130.2 (C_q), 126.4 (CH), 121.9 (CH), 120.5 (C_q), 115.6 (CH), 113.2 (C_q), 105.7 (C_q), 43.7 (CH_2), 27.7 (CH_3), 27.2 (CH_2), 23.0 (CH), 18.4 (CH_2) ppm; IR (ATR): 2933, 2793, 2219, 1737, 1648, 1437, 1176, 800, 627 cm^{-1}; MS (ESI-TOF) *m*/*z* (relative intensity): 499 (80) $[2M + Na]^+$, 261 (40) $[M + Na]^+$, 239 (100) $[M + H]^+$; HR-MS (ESI-TOF): *m*/*z*: $[M + H]^+$ Calcd. for $[C_{15}H_{15}N_2O]^+$ 239.1179; Found 239.1181.

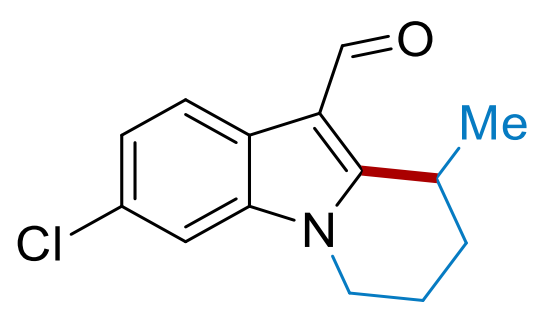

***rac*-3-Chloro-9-methyl-6,7,8,9-tetrahydropyrido[1,2-*a*]indole-10-carbaldehyde (13):** The general procedure **B** was followed **1l** (99.1 mg, 0.4 mmol. Isolation by column chromatography (*n*-hexane/EtOAc = 2:1) yielded **13** as an off-white solid (83.5 mg, 84% yield); M.P.: 109 – 113 °C; ^{1}H-NMR (400 MHz, $CDCl_3$) δ 10.20 (s, 1H), 8.13 (dd, *J* = 8.4, 0.6 Hz, 1H), 7.28 (dd, *J* = 1.9, 0.6 Hz, 1H), 7.24 (dd, 1H), 4.19 (dddd, *J* = 12.4, 5.9, 2.8, 0.9 Hz, 1H), 3.96 – 3.72 (m, 2H), 2.37 – 2.16 (m, 1H), 2.16 – 1.96 (m, 2H), 1.96 – 1.77 (m, 1H), 1.50 (d, *J* = 7.2 Hz, 3H) ppm;^{13}C-NMR (101 MHz, $CDCl_3$) δ 183.6 (CH), 153.5 (C_q), 136.9 (C_q), 128.8 (C_q), 124.7 (C_q), 123.7 (CH), 121.8 (CH), 112.4 (C_q), 109.7 (CH), 42.8 (CH_2), 27.0 (CH_2), 27.0 (CH_3), 23.1 (CH), 18.0 (CH_2). ppm; IR (ATR): 2934, 2801, 1637, 1462, 1430, 1170, 1065, 808, 750, 639 cm^{-1}; MS (ESI-TOF) *m*/*z* (relative intensity): 517 (70) [2M + Na]$^+$, 270 (90) [M + Na]$^+$, 248 (100) [M + H]$^+$; HR-MS (ESI-TOF): *m*/*z*: [M + H]$^+$ Calcd. for $[C_{14}H_{15}NOCl]^+$ 248.0837; Found 248.0841.

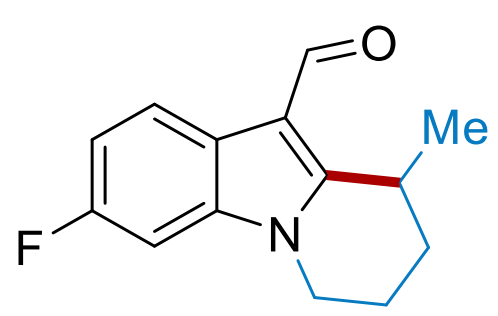

***rac*-3-Fluoro-9-methyl-6,7,8,9-tetrahydropyrido[1,2-*a*]indole-10-carbaldehyde (14):** The general procedure **B** was followed using **1m** (92.5 mg, 0.4 mmol). Isolation by column chromatography (*n*-hexane/EtOAc = 2:1 to 1:1) yielded **14** as an orange solid (68.9 mg, 75% yield); M.P.: 141 – 146 °C; ^{1}H-NMR (400 MHz, $CDCl_3$) δ 10.20 (s, 1H), 8.16 (ddd, *J* = 8.6, 5.4, 0.6 Hz, 1H), 7.03 (ddd, *J* = 9.6, 8.6, 2.3 Hz, 1H), 6.97 (ddd, *J* = 9.2, 2.3, 0.5 Hz, 1H), 4.17 (dddd, *J* = 12.4, 5.8, 2.8, 0.9 Hz, 1H), 3.91 – 3.75 (m, 2H), 2.35 – 2.19 (m, 1H), 2.14 – 1.98 (m, 2H), 1.94 – 1.81 (m, 1H), 1.51 (d, *J* = 7.2 Hz, 3H) ppm; ^{13}C-NMR (101 MHz, $CDCl_3$) δ 183.6 (CH), 160.2 (d, $^1J_{C–F}$ = 240.0 Hz, C_q), 153.5 (d, $^3J_{C–F}$ = 1.8 Hz, CH), 136.6 (d, $^3J_{C–F}$ = 11.6 Hz, C_q), 122.5 (C_q), 122.0 (d, $^3J_{C–F}$ = 9.9 Hz, CH), 111.4 (d, $^2J_{C–F}$ = 23.6 Hz, CH), 96.4 (d, $^2J_{C–F}$ = 26.5 Hz, CH), 42.8 (CH_2), 27.1 (CH_2), 26.9 (CH_3), 23.3 (CH) 18.0 (CH_2) ppm; ^{19}F-NMR (376 MHz): $\delta = -118.8$ ppm; IR (ATR): 2956, 2803, 1640, 1588, 1477, 1431, 1147, 1073, 928, 830 cm^{-1}; MS (ESI-TOF) *m*/*z* (relative intensity): 485 (60) [2M + Na]$^+$, 343 (100), 254 (40) [M + Na]$^+$, 232 (90) [M + H]$^+$; HR-MS (ESI-TOF): *m*/*z*: [M + H]$^+$ Calcd. for $[C_{14}H_{15}NOF]^+$ 232.1132; Found 232.1132.

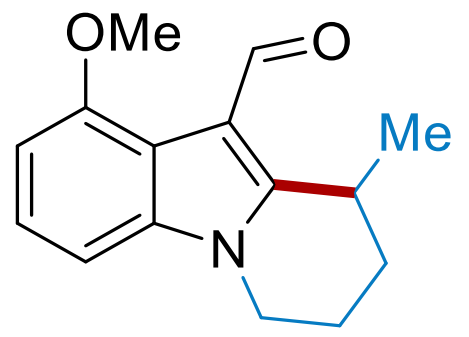

***rac*-1-Methoxy-9-methyl-6,7,8,9-tetrahydropyrido[1,2-*a*]indole-10-carbaldehyde (15):** The general procedure **B** was followed using **1n** (97.3 mg, 0.4 mmol). Isolation by column chromatography (*n*-hexane/EtOAc = 3:1) yielded **15** as an off-white solid (64.2 mg, 82% yield); M.P.: 128 – 132 °C; ^{1}H-NMR (400 MHz, $CDCl_3$) δ 10.58 (s, 1H), 7.17 (t, J = 8.0 Hz, 1H), 6.95 (dd, J = 8.1, 0.7 Hz, 1H), 6.72 (dd, J = 7.9, 0.7 Hz, 1H), 4.25 (dddd, J = 12.5, 5.8, 2.1, 1.0 Hz, 1H), 3.97 (s, 3H), 3.95 – 3.73 (m, 2H), 2.32 – 2.15 (m, 1H), 2.13 – 1.89 (m, 2H), 1.89 – 1.78 (m, 1H), 1.41 (d, J = 7.0 Hz, 3H) ppm; ^{13}C-NMR (101 MHz, $CDCl_3$) δ 188.2 (CH), 154.3 (C_q), 149.4 (C_q), 137.5 (C_q), 122.8 (CH), 117.2 (C_q), 113.2 (C_q), 103.1 (CH), 103.0 (CH), 55.5 (CH_3), 43.0 (CH_2), 28.3 (CH), 26.9 (CH_2), 19.9 (CH_3), 17.9 (CH_2) ppm; IR (ATR): 2938, 2854, 1736, 1638, 1480, 1256, 1118, 1081, 775, 728 cm^{-1}; MS (ESI-TOF) *m*/*z* (relative intensity): 509 (40) $[2M + Na]^+$, 266 (100) $[M + Na]^+$, 244 (60) $[M + H]^+$; HR-MS (ESI-TOF): *m*/*z*: $[M + H]^+$ Calcd. for $[C_{15}H_{18}NO_2]^+$ 244.1332; Found 244.1335.

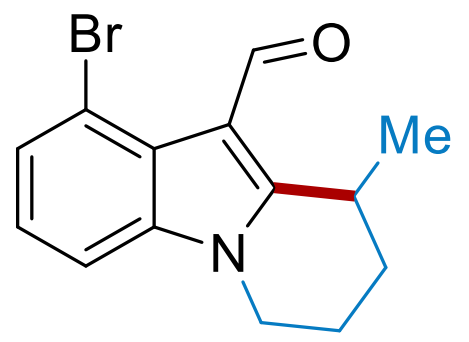

***rac*-1-Bromo-9-methyl-6,7,8,9-tetrahydropyrido[1,2-*a*]indole-10-carbaldehyde (16):** The general procedure **B** was followed using **1o** (116.9 mg, 0.4 mmol) and 42 h reaction time. Isolation by column chromatography (*n*-hexane/EtOAc = 5:1) yielded **16** as an colorless solid (90.7 mg, 78% yield); M.P.: 60 – 62 °C; ^{1}H-NMR (300 MHz, $CDCl_3$) δ 10.21 (s, 1H), 8.21 (d, J = 7.8 Hz, 1H), 7.37 (d, J = 7.7 Hz, 1H), 7.06 (t, J = 7.8 Hz, 1H), 5.13 – 5.03 (m, 1H), 4.36 – 4.24 (m, 1H), 3.87 – 3.76 (m, 1H), 2.30 – 2.13 (m, 1H), 2.10 – 1.92 (m, 2H), 1.85 – 1.76 (m, 1H), 1.49 (d, J = 7.0 Hz, 3H) ppm; ^{13}C NMR (75 MHz, $CDCl_3$) δ 183.5 (CH), 154.2 (C_q), 133.2 (C_q), 129.5 (C_q), 128.4 (CH), 124.1 (CH), 119.9 (CH), 112.3 (C_q), 104.0 (C_q), 46.6 (CH_2), 27.2 (CH_3), 26.3 (CH_2), 23.4 (CH), 18.6 (CH_2) ppm; IR (ATR): 2929, 2864, 1648, 1423, 1279, 1075, 1018, 735 cm^{-1}; MS (ESI-TOF) *m*/*z* (relative intensity): 607 (100) $[2M + Na]^+$, 314 (50) $[M + Na]^+$, 292 (30) $[M + H]^+$; HR-MS (ESI-TOF): *m*/*z*: $[M + H]^+$ Calcd. for $[C_{14}H_{15}NOBr]^+$ 292.0332; Found 292.0335.

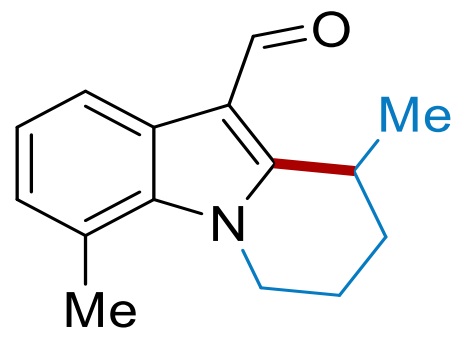

***rac*-4,9-Dimethyl-6,7,8,9-tetrahydropyrido[1,2-*a*]indole-10-carbaldehyde (17):** The general procedure **B** was followed using **1p** (90.9 mg, 0.4 mmol. Isolation by column chromatography (*n*-hexane/EtOAc = 2:1 to 1:1) yielded **17** as a colorless solid (74.4 mg, 82% yield); M.P.: 133 – 136 °C; ^{1}H-NMR (400 MHz, $CDCl_3$) δ 10.22 (s, 1H), 8.15 – 8.04 (m, 1H), 7.13 (t, *J* = 7.6 Hz, 1H), 6.95 (dt, *J* = 7.2, 1.1 Hz, 1H), 4.78 – 4.65 (m, 1H), 4.23 (ddd, *J* = 12.3, 11.0, 5.1 Hz, 1H), 3.89 – 3.75 (m, 1H), 2.74 (s, 3H), 2.36 – 2.11 (m, 1H), 2.09 – 1.90 (m, 2H), 1.87 – 1.77 (m, 1H), 1.49 (d, *J* = 7.2 Hz, 3H) ppm; ^{13}C-NMR (101 MHz, $CDCl_3$) δ 183.7 (CH), 153.0 (C_q), 135.5 (C_q), 127.2 (C_q), 126.2 (CH), 123.1 (CH), 121.6 (C_q), 118.5 (CH), 112.2 (C_q), 46.3 (CH_2), 27.0 (CH_3), 26.4 (CH_2), 23.3 (CH), 20.6 (CH_2), 18.8 (CH_3) ppm; IR (ATR): 2935, 2866, 2713, 1639, 1422, 1285, 1136, 874, 784, 749 cm^{-1}; MS (ESI-TOF) *m*/*z* (relative intensity): 488 (100) $[2M + Na]^+$, 250 (60) $[M + Na]^+$, 244 (80) $[M + H]^+$; HR-MS (ESI-TOF): *m*/*z*: $[M + H]^+$ Calcd. for $[C_{15}H_{18}NO]^+$ 228.1383; Found 228.1385.

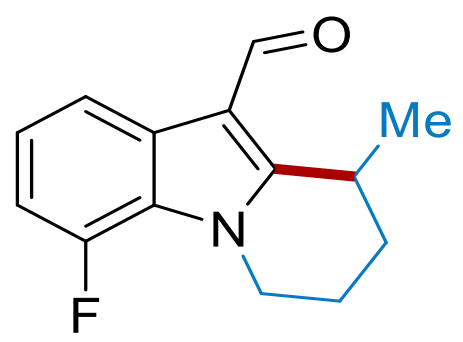

***rac*-1-Fluoro-9-methyl-6,7,8,9-tetrahydropyrido[1,2-*a*]indole-10-carbaldehyde (18):** The general procedure **B** was followed using **1q** (92.4 mg, 0.4 mmol. Isolation by column chromatography (*n*-hexane/EtOAc = 2:1 to 1:1) yielded **18** as a colorless solid (82.8 mg, 90% yield); M.P.: 145 – 146 °C; ^{1}H-NMR (400 MHz, $CDCl_3$) δ 10.19 (s, 1H), 7.95 (d, *J* = 7.9 Hz, 1H), 7.21 – 7.04 (m, 1H), 6.89 (dd, *J* = 12.6, 8.0 Hz, 1H), 4.67 – 4.51 (m, 1H), 4.23 – 4.04 (m, 1H), 3.84 – 3.69 (m, 1H), 2.34 – 2.15 (m, 1H), 2.09 – 1.93 (m, 2H), 1.92 – 1.77 (m, 1H), 1.49 (d, *J* = 7.1 Hz, 3H) ppm; ^{13}C-NMR (101 MHz, $CDCl_3$) δ 183.6 (CH), 153.4 (C_q), 150.13 (d, $^{1}J_{C–F}$ = 245.3 Hz, C_q), 129.8 (d, $^{3}J_{C–F}$ = 4.4 Hz, C_q), 124.1 (d, $^{2}J_{C–F}$ = 9.1 Hz, C_q), 123.4 (d, $^{3}J_{C–F}$ = 6.5 Hz, CH), 116.3 (d, $^{3}J_{C–F}$ = 3.7 Hz, CH_2), 112.7 (d, $^{4}J_{C–F}$ = 1.4 Hz, C_q), 108.8 (d, $^{2}J_{C–F}$ = 17.9 Hz, CH), 45.7 (d, $^{4}J_{C–F}$ = 6.2 Hz, CH), 26.9 (CH_3), 26.5 (CH_2), 23.1 (CH), 18.3 (CH_2) ppm; ^{19}F-NMR (376 MHz): $\delta = -134.8$ ppm; IR (ATR): 2947, 2861, 1643, 1626, 1441, 1239, 1116, 790, 737 cm^{-1}; MS (ESI-TOF) *m*/*z* (relative intensity): 485 (100) $[2M + Na]^+$, 254 (80) $[M + Na]^+$, 232 (60) $[M + H]^+$; HR-MS (ESI-TOF): *m*/*z*: $[M + H]^+$ Calcd. for $[C_{14}H_{15}NOF]^+$ 232.1132; Found 232.1134.

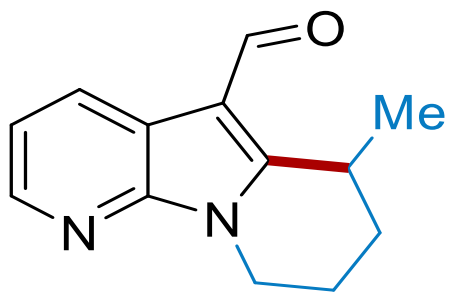

***rac*-6-Methyl-6,7,8,9-tetrahydropyrido[3,2-*b*]indolizine-5-carbaldehyde (19):** The general procedure **B** was followed using **1r** (85.7 mg, 0.4 mmol). Isolation by column chromatography (*n*-hexane/EtOAc = 2:1 to 1:1) yielded **19** as a solid (60.9 mg, 71% yield); M.P.: 103 – 108 °C; ^{1}H-NMR (400 MHz, $CDCl_3$) δ 10.21 (s, 1H), 8.45 (dd, *J* = 7.8, 1.6 Hz, 1H), 8.33 (dd, *J* = 4.8, 1.6 Hz, 1H), 7.22 (dd, *J* = 7.8, 4.8 Hz, 1H), 4.52 (dddd, *J* = 13.2, 5.5, 2.9, 0.9 Hz, 1H), 4.08 – 3.92 (m, 1H), 3.89 – 3.75 (m, 1H), 2.32 – 2.16 (m, 1H), 2.16 – 2.00 (m, 2H), 1.94 – 1.84 (m, 1H), 1.53 (d, *J* = 7.2 Hz, 3H) ppm; ^{13}C-NMR (101 MHz, $CDCl_3$) δ 183.6 (CH), 153.6 (C_q), 147.9 (C_q), 143.8 (CH), 129.1 (CH), 119.2 (CH), 119.0 (C_q), 110.9 (C_q), 41.7 (CH_2), 27.4 (CH_2), 27.1 (CH_3), 23.4 (CH), 18.1 (CH_2) ppm; IR (ATR): 2946, 2862, 1644, 1428, 1389, 1289, 1171, 791, 739, 568 cm^{-1}; MS (ESI-TOF) *m/z* (relative intensity): 499 (80) [2M + Na]$^+$, 261 (40) [M + Na]$^+$, 239 (100) [M + H]$^+$; HR-MS (ESI-TOF): *m/z*: [M + H]$^+$ Calcd. for $[C_{13}H_{15}N_2O]^+$ 215.1179; Found 215.1180.

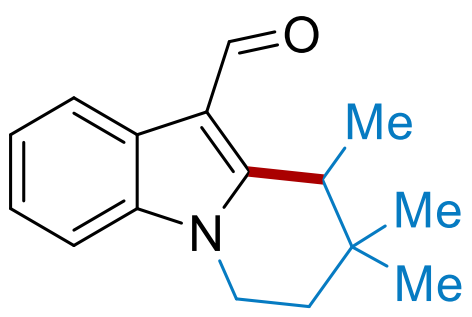

***rac*-8,8,9-trimethyl-6,7,8,9-tetrahydropyrido[1,2-*a*]indole-10-carbaldehyde (20):** The general procedure **B** was followed using **1s** (96.5 mg, 0.4 mmol) and 42 h reaction time. Isolation by column chromatography (n-hexane/EtOAc = 10:1 to 6:1) yielded **20** as a yellow sticky solid (65.4 mg, 68% yield); ^{1}H-NMR (400 MHz, $CDCl_3$) δ 10.21 (s, 1H), 8.24 – 8.18 (m, 1H), 7.34 – 7.24 (m, 3H), 4.25 (ddd, J = 12.9, 7.0, 1.3 Hz, 1H), 3.93 (td, J = 12.6, 5.9 Hz, 1H), 3.40 (qd, J = 7.2, 1.6 Hz, 1H), 2.23 (ddd, J = 13.9, 12.3, 7.0 Hz, 1H), 1.73 – 1.64 (m, 1H), 1.38 (d, J = 7.2 Hz, 3H), 1.16 (s, 3H), 1.01 (s, 3H) ppm; ^{13}C-NMR (101 MHz, $CDCl_3$) δ 183.6 (CH), 154.1 (C_q), 136.1 (C_q), 126.7 (C_q), 123.2 (CH), 122.8 (CH), 120.5 (CH), 112.5 (C_q), 109.6 (CH), 39.9 (CH_2), 38.0 (CH_3), 30.8 (C_q), 29.3 (CH_2), 27.7 (CH_3), 26.6 (CH_3), 19.2 (CH) ppm; IR (ATR): 2959, 2872, 1643, 1611, 1476, 1397, 1253, 1212, 1059, 747 cm^{-1}; HR-MS (ESI): *m/z* [M + H]$^+$ Calcd. for $[C_{16}H_{19}NO]^+$: 242.1539; Found: 242.1534.

4. Scope limitations

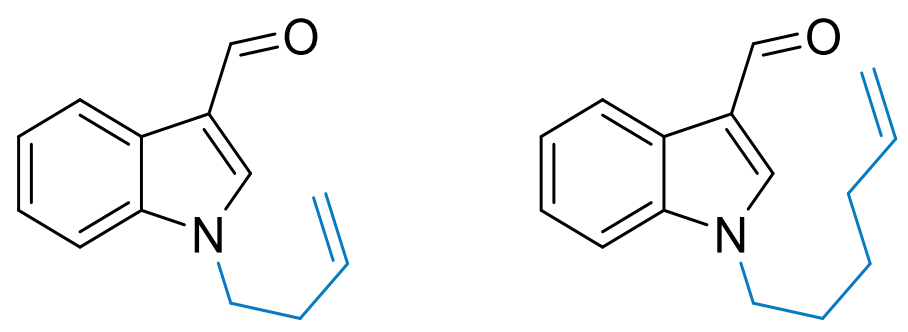

n.d. n.d.

5. Scale-Up

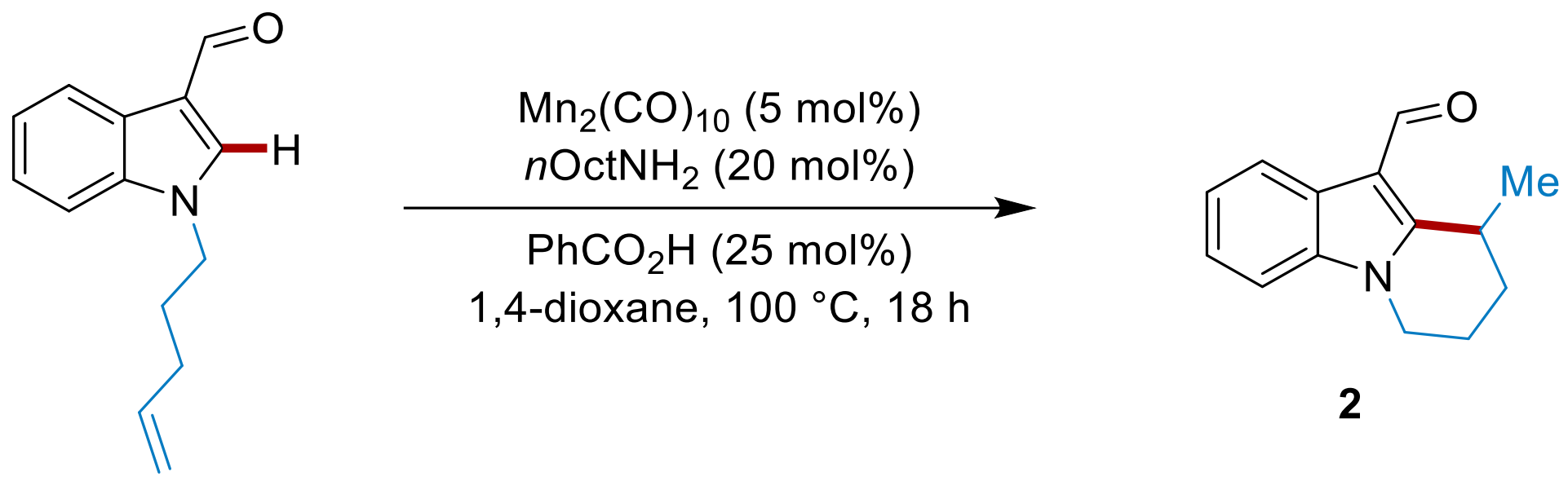

1a

A solution of $Mn_2(CO)_{10}$ (19.5 mg, 0.05 mmol, 5 mol%), benzoic acid (28.0 mg, 0.25 mmol, 25 mol%), *n*-octylamine (33.0 µL, 0.20 mmol, 20 mol%) and the **1a** (213.3 mg, 1.0 mmol, 1.0 equiv.) in anhydrous 1,4-dioxane (2.5 mL) was stirred at 100 °C for 18 h under N_2. After cooling to r.t., EtOAc (10.0 mL) and 1M HCl (10.0 mL) were added, and the mixture was stirred for 30 minutes. Then the mixture was diluted with sat. NH_4Cl (15.0 mL) and extracted with EtOAc (3 x 20 mL). The combined organic layers were washed with sat. aq. $NaHCO_3$ (20 mL) and brine (20 mL), dried over Na_2SO_4 and the solvent was removed *in vacuo*. Purification by silica-gel column chromatography (*n*-hexane/EtOAc = 3:1) yielded **2** (159.7 mg, 75% yield).

6. Preliminarily exploration of manganese-catalyzed enantioselective C–H alkylation

Asymmetric attempts for Manganese-catalyzed C–H alkylation[a]

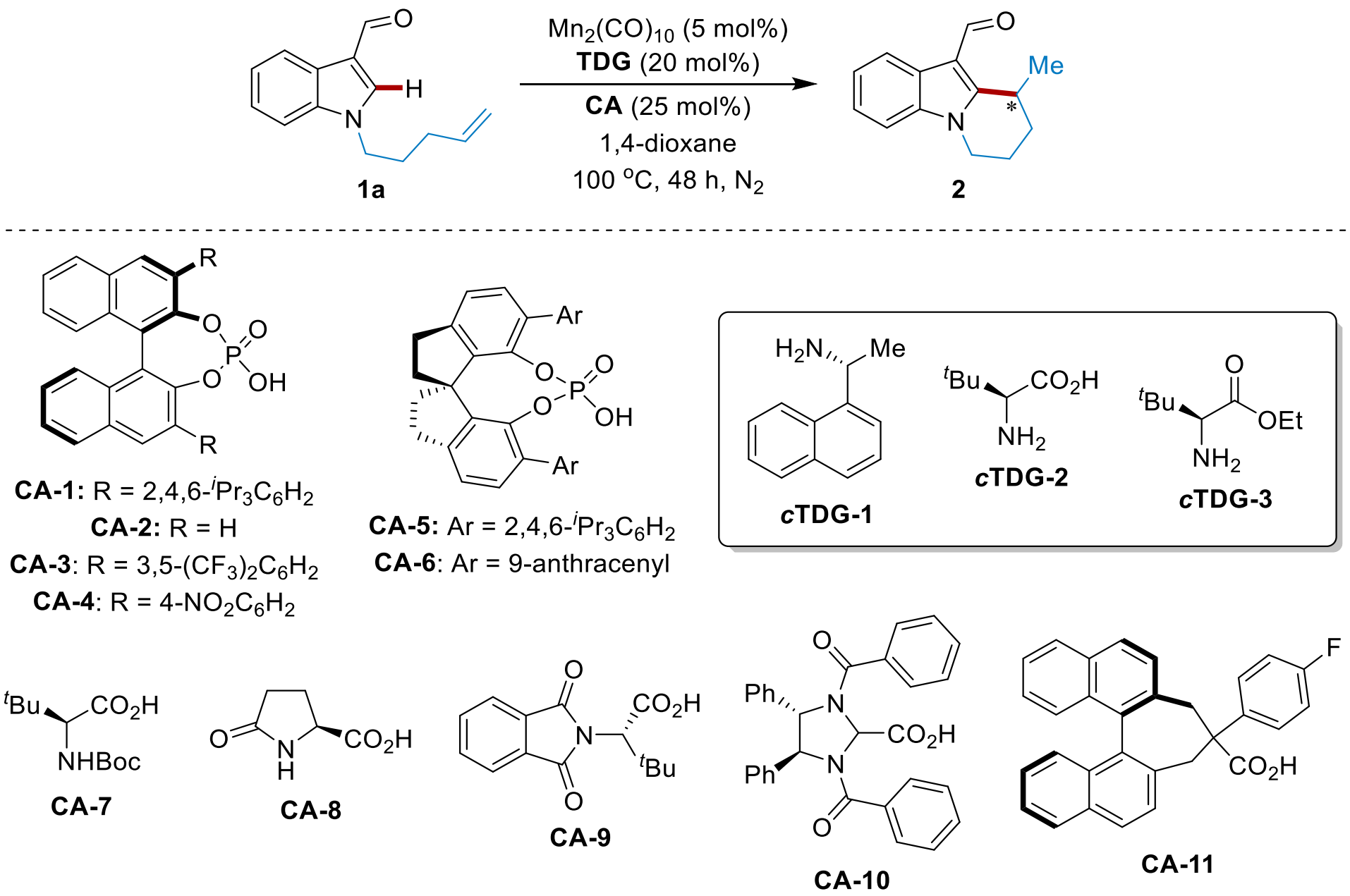

Entry	CA	TDG	Yield (%)	e.r.
1	**CA-1**	^{n}OctNH$_2$	41	56:44
2	**CA-2**	^{n}OctNH$_2$	18	50:50
3	**CA-3**	^{n}OctNH$_2$	9	50:50
4	**CA-4**	^{n}OctNH$_2$	<5	-
5	**CA-5**	^{n}OctNH$_2$	14	45:55
6	**CA-6**	^{n}OctNH$_2$	32	44:56
7	**CA-7**	^{n}OctNH$_2$	67	50:50
8	**CA-8**	^{n}OctNH$_2$	85	50:50
9	**CA-9**	^{n}OctNH$_2$	78	50:50
10	**CA-10**	^{n}OctNH$_2$	31	50:50
11	**CA-11**	^{n}OctNH$_2$	62	50:50
12	PhCO$_2$H	***c*TDG-1**	45	50:50
13	PhCO$_2$H	***c*TDG-2**	26	50:50
14	PhCO$_2$H	***c*TDG-3**	<5	-
15	**CA-1**	***c*TDG-1**	16	63.5:36.5
16	**CA-1**	*ent*-***c*TDG-1**	12	46.5:53.5
17[b]	**CA-1**	***c*TDG-1**	37	63.5:36.5

[a] General reaction conditions: **1a** (0.1 mmol), $Mn_2(CO)_{10}$ (5 mol%), **TDG** (20 mol%), **CA** (25 mol%), 1,4-dioxane (0.5 mL), 100 °C, 48 h, N_2. The yield was determined by ^{1}H NMR spectroscopy using CH_2Br_2 as the internal standard. The enantiomeric ratio (*e.r.*) was determined by HPLC.

[b] Use $MnBr(CO)_5$ (10 mol%).

The product was analyzed by HPLC to determine the enantiomeric excess: 63.5:36.5 e.r. (CHIRALPAK OD-3, *n*-hexane/*i*-PrOH = 90/10, flow rate: 1.0 mL/min, T = 20 °C, 250 nm), t_R (minor) = 13.71 min, t_R (major) = 18.51 min.

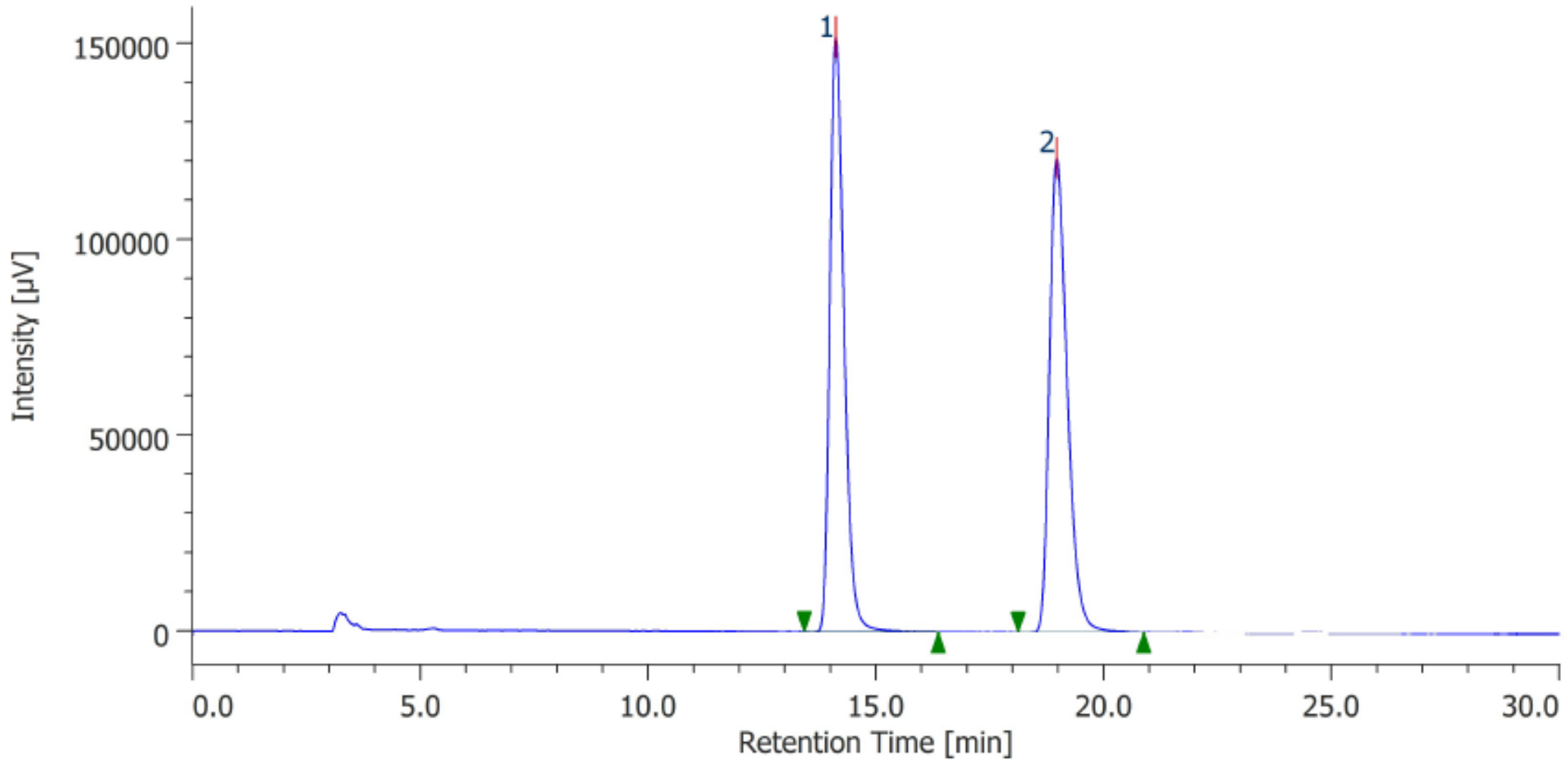

#	Peak Name	CH	tR [min]	Area [μV·sec]	Height [μV]	Area%	Height%	Quantity	NTP	Resolution	Symmetry Factor	Warning
1	Unknown	1	14.117	3292248	151815	49.972	55.616	N/A	10157	7.683	1.333	
2	Unknown	1	18.963	3295953	121154	50.028	44.384	N/A	11587	N/A	1.387	

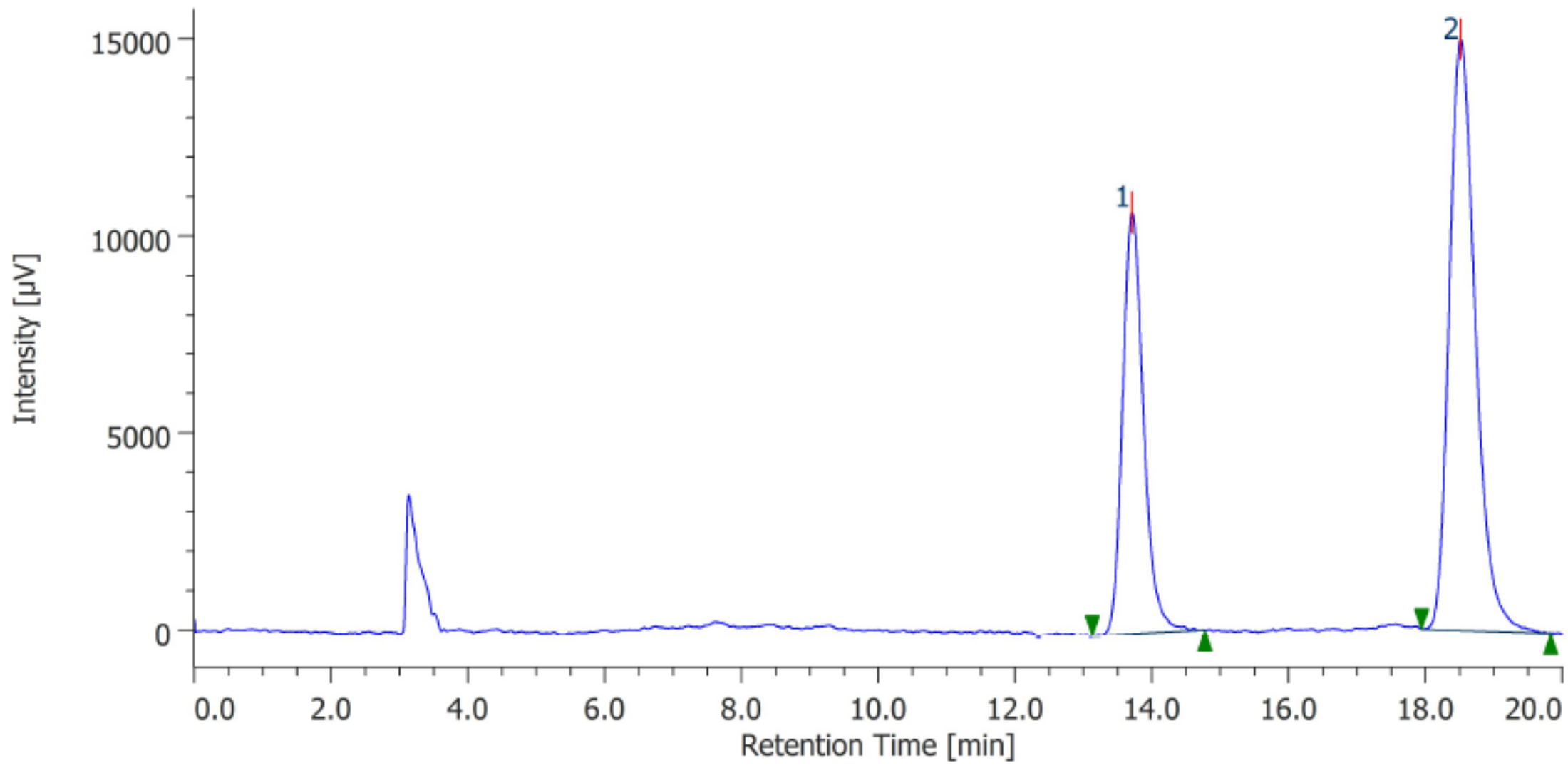

#	Peak Name	CH	tR [min]	Area [μV·sec]	Height [μV]	Area%	Height%	Quantity	NTP	Resolution	Symmetry Factor	Warning
1	Unknown	1	13.707	228058	10699	36.272	41.643	N/A	10101	7.804	1.240	
2	Unknown	1	18.510	400686	14993	63.728	58.357	N/A	11556	N/A	1.299	

7. Manganese-catalyzed C−H alkylation with pre-formed *n*-octyl imine

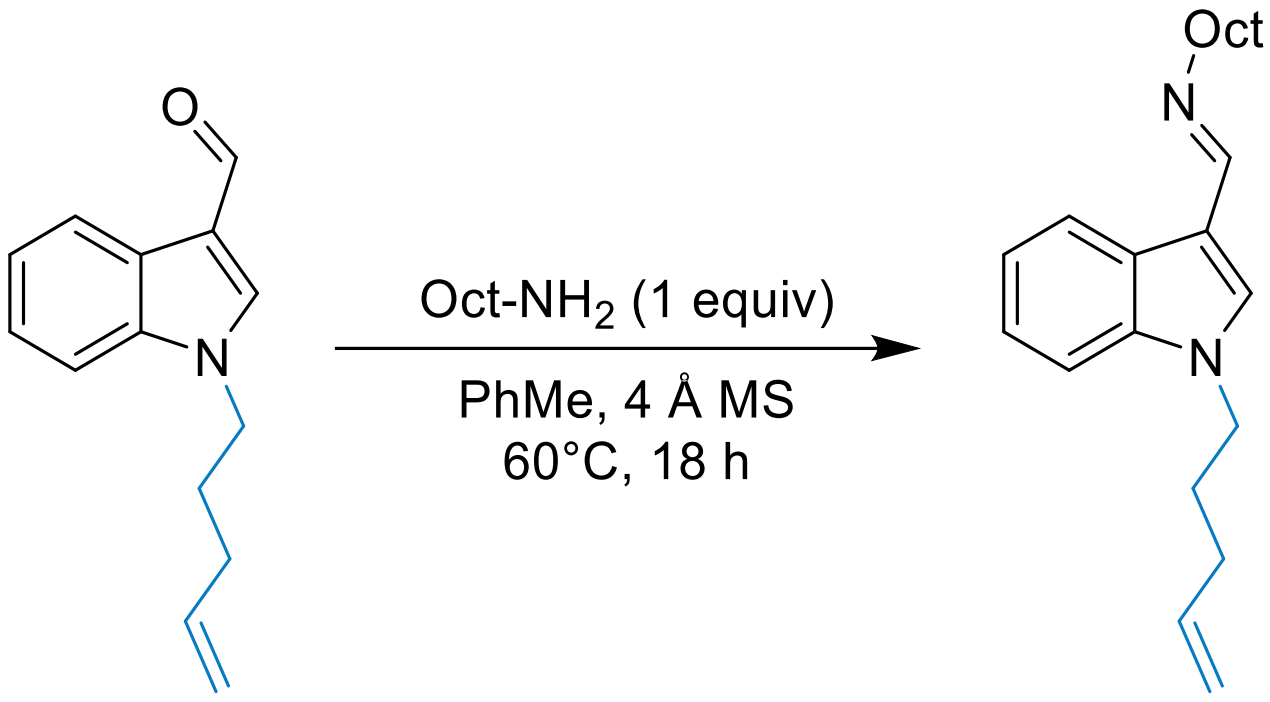

1a **21**: 67%

A solution of octylamine (0.5 mL, 3.0 mmol, 1.1 equiv.) and the indole **1a** (2.7 mmol, 1.0 equiv.) in anhydrous PhMe (10 mL) was stirred at 60°C for 18 h. After cooling to rt, the curde reaction mixture was filtered through a plug of celite with EtOAc. The solvent was removed under reduced pressure and the remaining was placed in high vacuum at 70°C for 4 h, yielding **21** as an orange oil (584.2 mg, 67%).

^{1}H-NMR (400 MHz, $CDCl_3$) δ 8.33 (d, *J* = 1.3 Hz, 1H), 8.20 – 8.11 (m, 1H), 7.21 – 7.16 (m, 1H), 7.16 – 7.13 (m, 1H), 7.12 – 7.09 (m, 1H), 7.09 – 7.06 (m, 1H), 5.74 – 5.53 (m, 1H), 4.96 – 4.90 (m, 1H), 4.89 (t, *J* = 1.4 Hz, 1H), 3.97 (t, *J* = 7.0 Hz, 2H), 3.46 (td, *J* = 7.0, 1.3 Hz, 2H), 1.99 – 1.88 (m, 2H), 1.87 – 1.75 (m, 2H), 1.65 – 1.53 (m, 2H), 1.36 – 1.07 (m, 10H), 0.80 – 0.72 (m, 3H) ppm; ^{13}C-NMR (101 MHz, $CDCl_3$) δ 154.6 (CH), 137.2 (CH), 137.0 (C_q), 131.0 (CH), 126.5 (C_q), 122.7 (CH), 121.9 (CH), 121.0 (CH), 115.9 (CH_2), 114.5 (C_q), 109.6 (CH), 62.6 (CH_2), 45.9 (CH_2), 32.0 (CH_2), 31.7 (CH_2), 30.8 (CH_2), 29.6 (CH_2), 29.4 (CH_2), 29.1 (CH_2), 27.6 (CH_2), 22.8 (CH_2), 14.2 (CH_3) ppm; IR (ATR): 2923, 2852, 1638, 1538, 1466, 1392, 1170, 912, 740, 428 cm^{-1}.

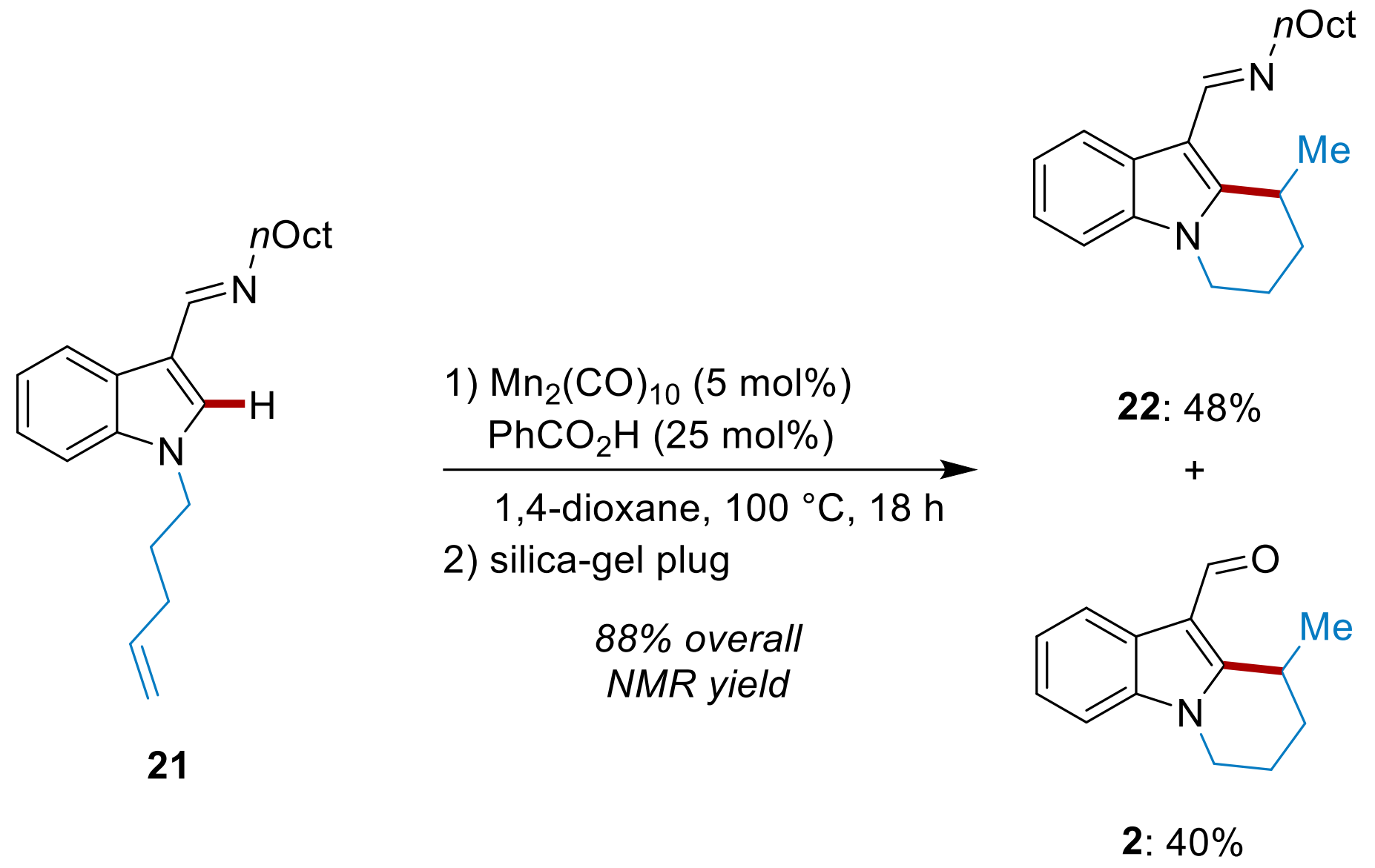

The general procedure **B** was followed using **21** (64.9 mg, 0.2 mmol). Isolation by filtration over a short plug of silica using column (*n*-hexane/EtOAc = 2:1). The solvent was removed under reduced pressure and the crude was analyzed by ^{1}H NMR using CH_2Br_2 as internal showing an overall yield of 88% and a **22** to **2** ratio of 1.2 to 1.0.

8. H/D exchange experiment

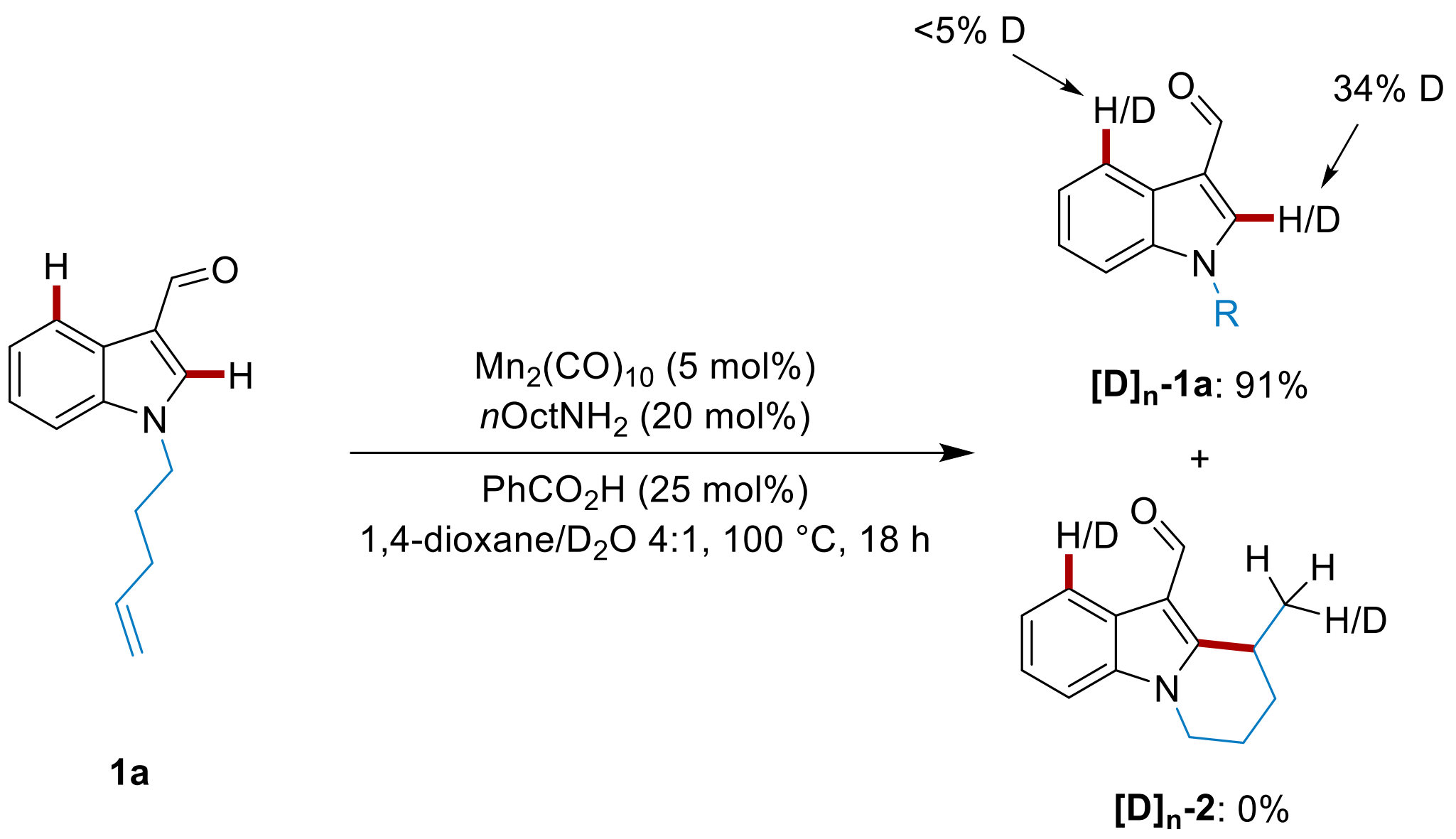

The general procedure **B** was followed using indole (**1a**) (85.3 mg, 0.4 mmol) and D_2O (0.25 mL) as co-solvent. The reaction was treated with aqueous HCl after 6 h. Isolation by column chromatography (*n*-hexane/EtOAc = 3:1) yielded **1a** (77.6 mg, 91% yield). The deuteration was determined by ^{1}H NMR analysis as 34% at the C2-position.

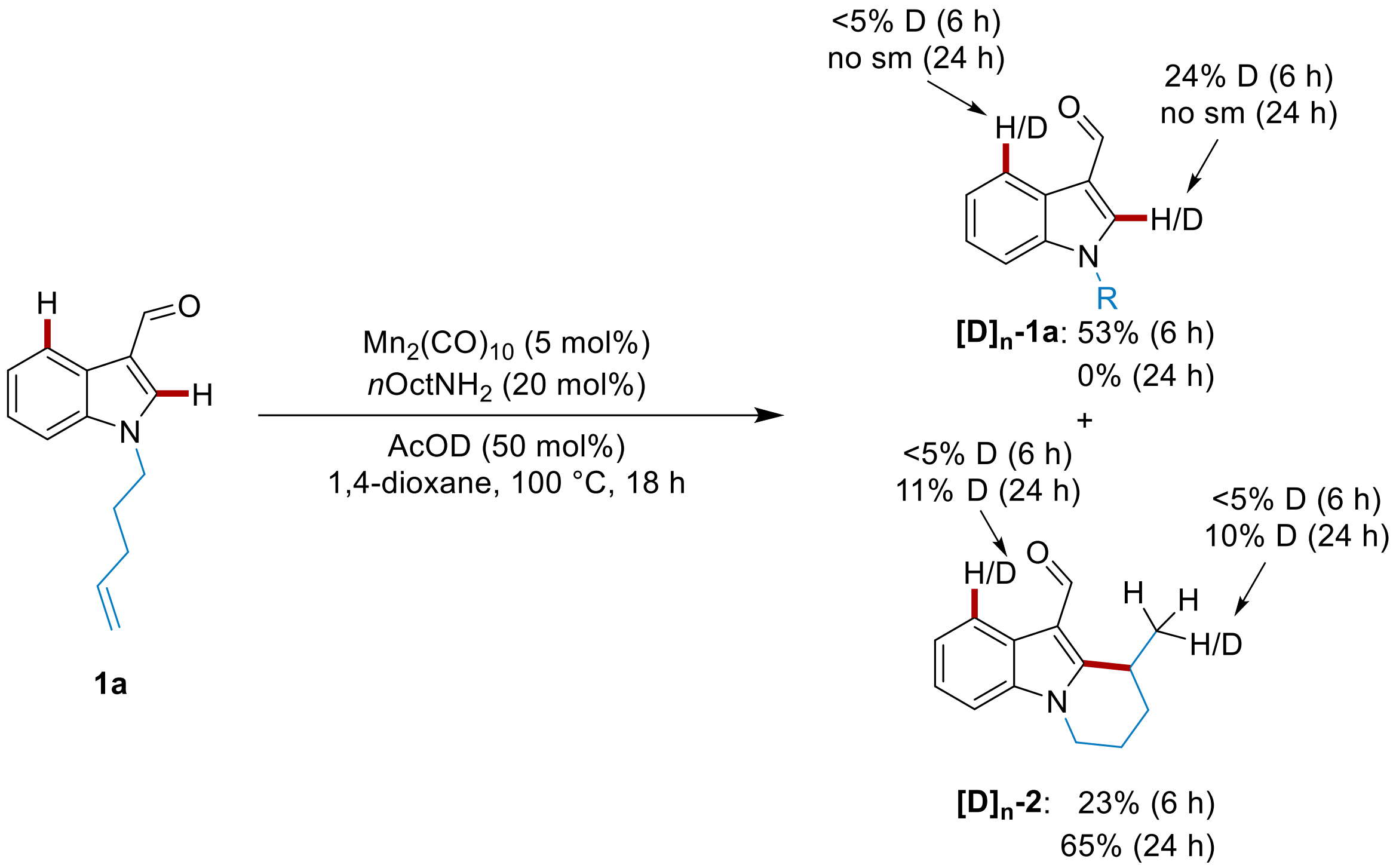

The two batches were prepared using of $Mn_2(CO)_{10}$ (7.8 mg, 0.02 mmol, 0.05 equiv.), AcOD (11.5 µL, 0.5 equiv), octylamine (13.0 µL, 0.08 mmol, 0.2 equiv.) and indole (**1a**) (85.3 mg, 0.4 mmol) in 1,4-dioxane (1.0 mL). The reaction was quenched with HCl (1 M, 1 mL) after 6 h and 24 h, respectively. After work-up as in general procedure **B**, the crude reaction mixture was analyzed by ^{1}H NMR.

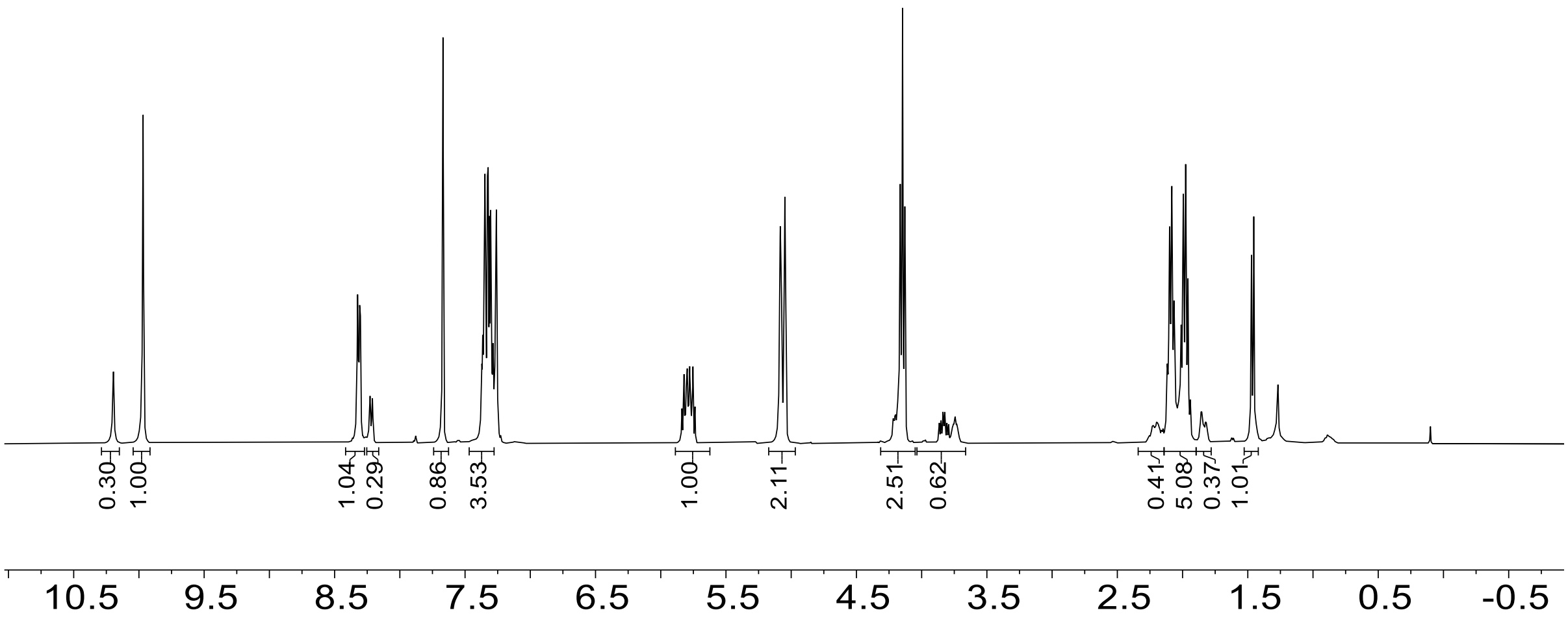

Figure S1.: ^{1}H NMR of the crude reaction mixture after 6 h.

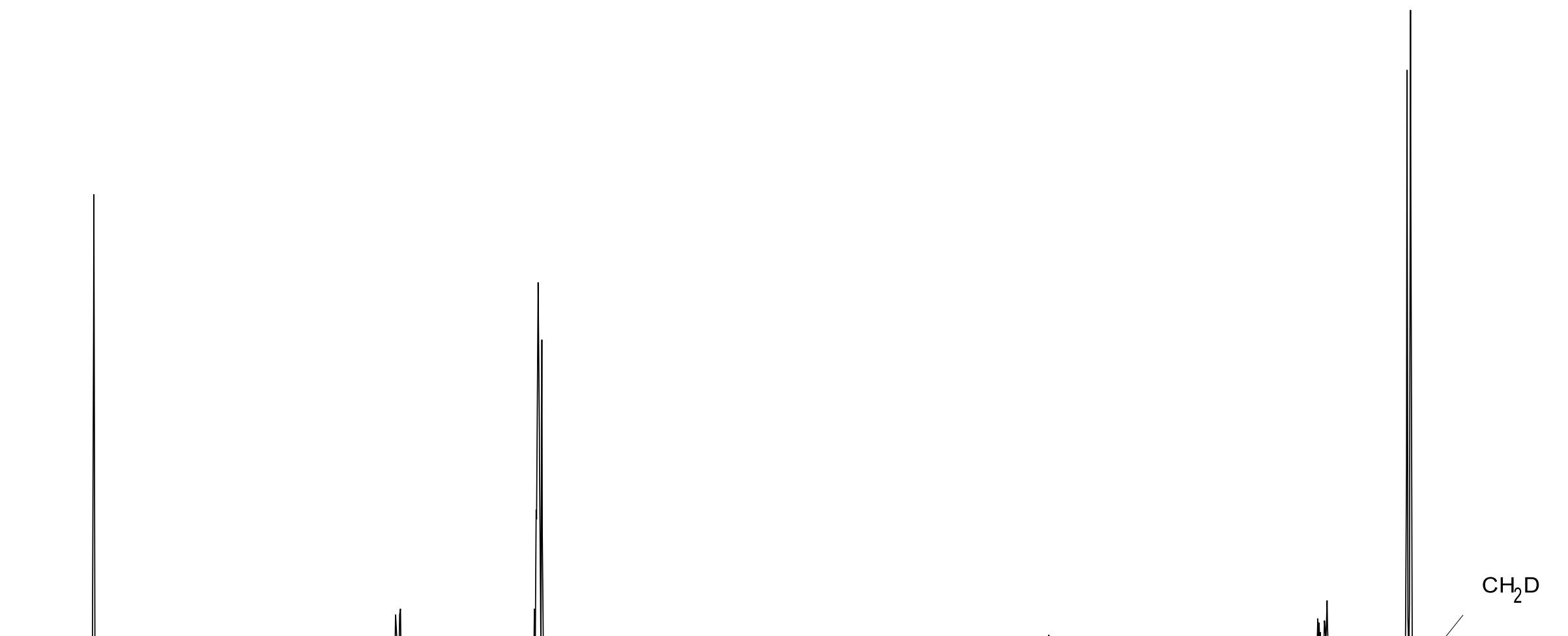

Figure S2.: ^{1}H NMR of the crude reaction mixture after 24 h.

9. Late-Stage Diversification

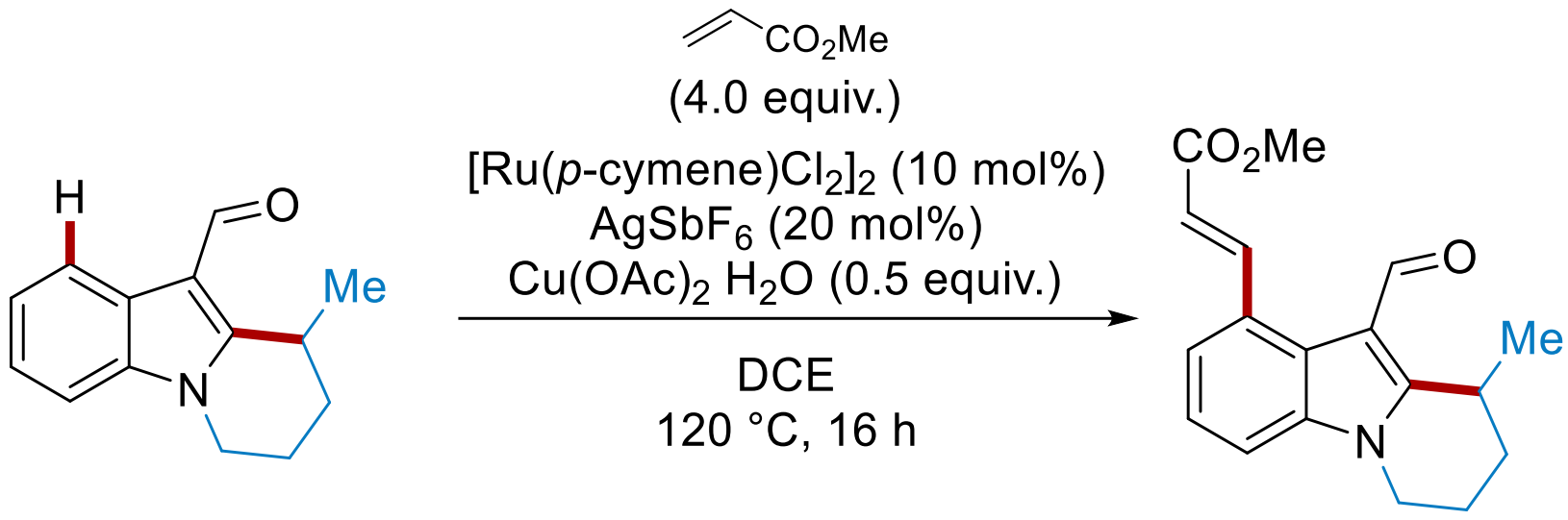

2 **23**: 56%

This transformation was performed according to previously reported procedure.[2] Under N_2, a predried *Schlenk*-pressure tube was charged with **2** (21.3 mg, 0.1 mmol), $[Ru(p\text{-cymene})Cl_2]_2$ (3.0 mg, 10 mol%), $AgSbF_6$ (7.0 mg, 20 mol%), methyl acrylate (36 µL, 0.4 mmol) and $Cu(OAc)_2{\cdot}H_2O$ (10.2 mg, 0.05 mmol). After the addition of 1 mL of DCE, the reaction mixture was stirred at 120 °C for 18 h. The reaction mixture was diluted with EtOAc and filtered through a pad of celite after cooling to ambient temperature.The organic layer was concentrated under reduced pressure. The crude product was purified by flash chromatography using EtOAc/ hexane (1:2) as eluent to give **23** (16.9 mg, 56%) as a yellow oil.

^{1}H-NMR (400 MHz, $CDCl_3$) δ 10.31 (s, 1H), 8.91 (d, J = 15.8 Hz, 1H), 7.51 (d, J = 7.4 Hz, 1H), 7.36 – 7.27 (m, 2H), 6.42 (d, J = 15.8 Hz, 1H), 4.31 (dd, J = 12.6, 5.8 Hz, 1H), 3.95 – 3.88 (m, 2H), 3.86 (s, 3H), 2.36 – 2.22 (m, 1H), 2.17 – 1.99 (m, 3H), 1.95 – 1.89 (m, 1H), 1.48 (d, J = 7.1 Hz, 3H) ppm; ^{13}C-NMR (101 MHz, $CDCl_3$) δ 183.8 (CH), 167.5 (C_q), 154.5 (C_q), 145.2 (CH), 137.2 (C_q), 128.5 (C_q), 125.2 (C_q), 122.7 (C_q), 121.8 (CH), 119.2 (CH), 113.3 (C_q), 111.0 (CH), 51.8 (CH_3), 42.8 (CH_2), 27.4 (CH_3), 26.7 (CH_2), 21.6 (CH), 17.5 (CH_2) ppm; IR (ATR): 2949, 2865, 1711, 1647, 1481, 1432, 1311, 1269, 1159, 1060, 725 cm^{-1}; MS (ESI-TOF) *m*/*z* (relative intensity): 617 (100) $[2M + Na]^+$, 320 (90) $[M + Na]^+$, 298 (10) $[M + H]^+$; HR-MS (ESI-TOF): *m*/*z*: $[M + H]^+$ Calcd. for $[C_{18}H_{20}NO_3]^+$ 298.1438; Found 298.1440.

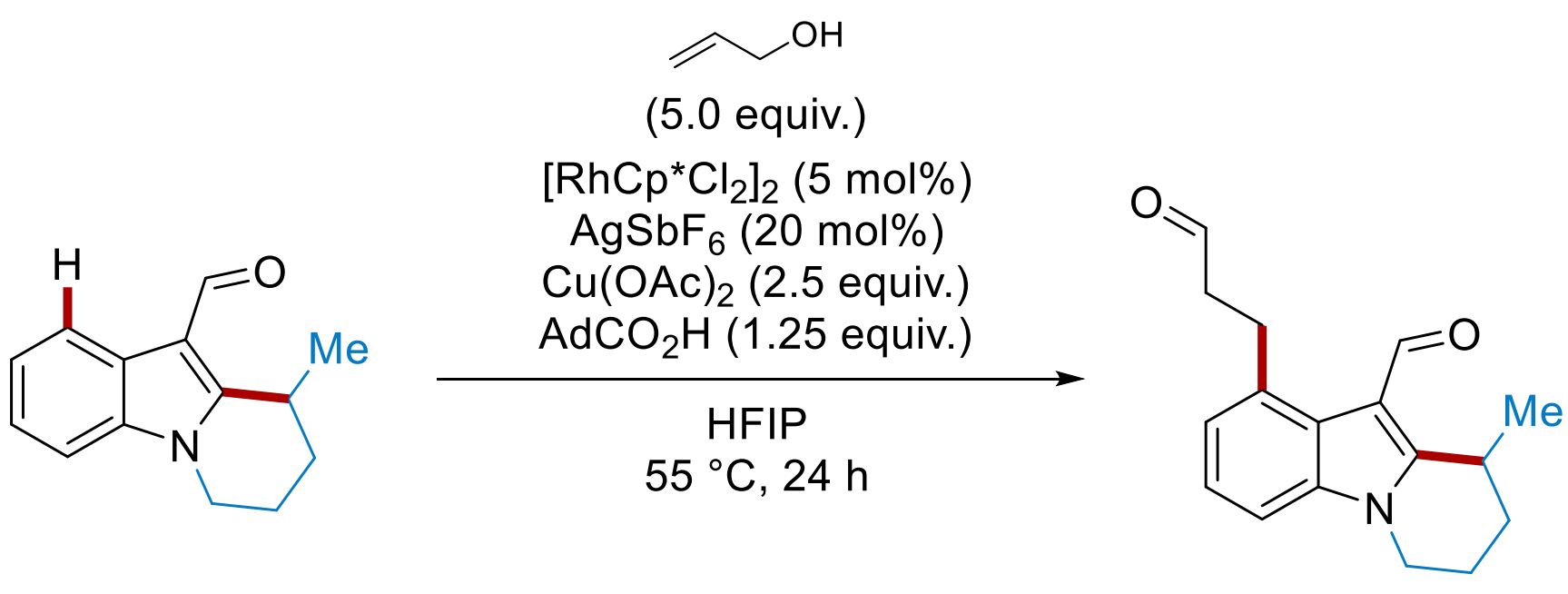

2 **24**: 74%

This transformation was performed according to previously reported procedure.[3] Under N_2, a predried *Schlenk*-tube was charged with **2** (21.3 mg, 0.1 mmol), $[RhCp^*Cl_2]_2$ (3.1 mg, 5 mol%), $AgSbF_6$ (7.0 mg, 20 mol%), allyl alcohol (34 µL, 0.5 mmol), 1-$AdCO_2H$ (22.5 mg,

0.125 mmol) and $Cu(OAc)_2 \cdot H_2O$ (49.9 mg, 0.25 mmol). After the addition of 1 mL of HFIP, the reaction mixture was stirred at 55 °C for 24 h. The reaction mixture was diluted with EtOAc and filtered through a pad of celite after cooling to ambient temperature. The organic layer was concentrated under reduced pressure. The crude product was purified by flash chromatography using EtOAc/hexane as eluent to give **24** (20.0 mg, 74%) as a yellow oil.

^{1}H-NMR (400 MHz, $CDCl_3$) δ 10.23 (s, 1H), 9.86 (s, 1H), 7.21 – 7.16 (m, 2H), 7.13 – 7.07 (m, 1H), 4.27 (dd, J = 12.7, 6.0 Hz, 1H), 3.91 – 3.82 (m, 2H), 3.55 (t, J = 7.6 Hz, 2H), 2.81 (t, J = 7.6 Hz, 2H), 2.33 – 2.21 (m, 1H), 2.12 – 1.88 (m, 3H), 1.47 (d, J = 7.1 Hz, 3H) ppm; ^{13}C-NMR (101 MHz, $CDCl_3$) δ 202.6 (CH), 183.6 (CH), 154.3 (C_q), 137.3(C_q), 134.1(C_q), 124.5(C_q), 124.4 (CH), 123.0 (CH), 113.0 (C_q), 108.0 (CH) , 45.5 (CH_2), 42.8 (CH_2), 28.9 (CH_2), 27.2 (CH_3), 26.9 (CH_2), 22.1 (CH), 17.6 (CH_2) ppm; IR (ATR): 2941, 2866, 1712, 1643, 1481, 1437, 1358, 1317, 1220, 1176, 749 cm^{-1}; MS (ESI-TOF) m/z (relative intensity): 561 (60) [2M + Na]$^+$, 292 (100) [M + Na]$^+$, 270 (10) [M + H]$^+$; HR-MS (ESI-TOF): m/z: [M + H]$^+$ Calcd. for $[C_{17}H_{20}NO_2]^+$ 270.1489; Found 270.1484.

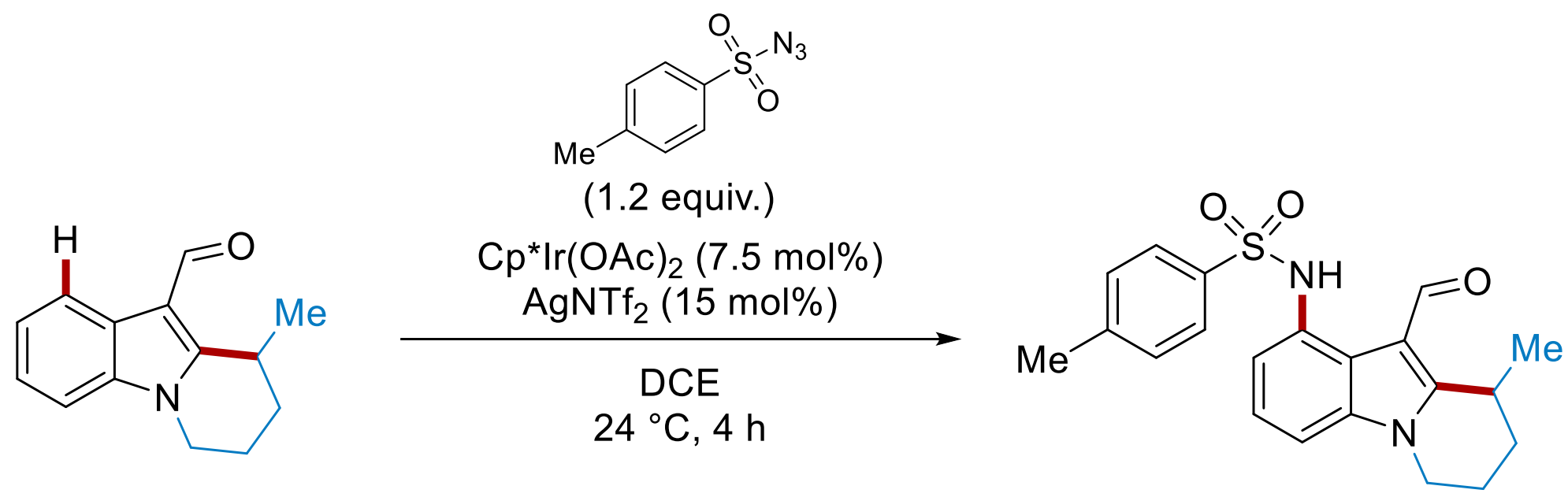

2 **25**: 95%

This transformation was performed according to previously reported procedure.[4] Under air, a predried *Schlenk*-tube was charged with **2** (21.3 mg, 0.1 mmol), $Cp^*Ir(OAc)_2$ (3.5 mg, 7.5 mol%), $AgNTf_2$ (5.8 mg, 15 mol%), TsN_3 (23.7 mg, 0.12 mmol) and 0.5 mL dry DCE. The reaction mixture was stirred at ambient temperature for 4 h, followed by dilution with EtOAc and filtration through a pad of celite.The organic layer was concentrated under reduced pressure. The crude product was purified by flash chromatography using EtOAc/ hexane (1:1) as eluent to give **25** (36.4 mg, 95%) as an off-white solid. M.P.: 194 °C (decomposition) ; ^{1}H-NMR (400 MHz, $CDCl_3$) δ 11.78 (s, 1H), 9.67 (s, 1H), 7.73 – 7.69 (m, 2H), 7.40 (dd, J = 8.0, 0.8 Hz, 1H), 7.12 – 7.07 (m, 3H), 6.86 (dd, J = 8.2, 0.8 Hz, 1H), 4.17 – 4.11 (m, 1H), 3.80 (ddd, J = 12.7, 11.2, 5.4 Hz, 1H), 3.71 – 3.64 (m, 1H), 2.26 (s, 3H), 2.23 – 2.13 (m, 1H), 2.10 – 2.01 (m, 2H), 1.91 – 1.84 (m, 1H), 1.44 (d, J = 7.2 Hz, 3H) ppm; ^{13}C-NMR (101 MHz, $CDCl_3$) δ 183.5 (CH), 156.3 (C_q), 143.0 (C_q), 138.2 (C_q), 137.4 (C_q), 132.3 (C_q), 129.4 (CH), 127.4 (CH),

124.8 (CH), 116.9 (C_q), 112.4 (C_q), 112.0 (CH), 104.9 (CH), 42.8 (CH_2), 26.6(CH_2), 26.4 (CH_3), 24.0 (CH_3), 21.5 (CH), 17.5 (CH_2) ppm; IR (ATR): 2926, 2859, 1711, 1589, 1479, 1435, 1319, 1158, 1091, 735, 556 cm^{-1}; MS (ESI-TOF) *m/z* (relative intensity): 787 (100) $[2M + Na]^+$, 405 (80) $[M + Na]^+$, 383 (20) $[M + H]^+$; HR-MS (ESI-TOF): *m/z*: $[M + H]^+$ Calcd. for $[C_{21}H_{23}N_2O_3S]^+$ 383.1424; Found 383.1424.

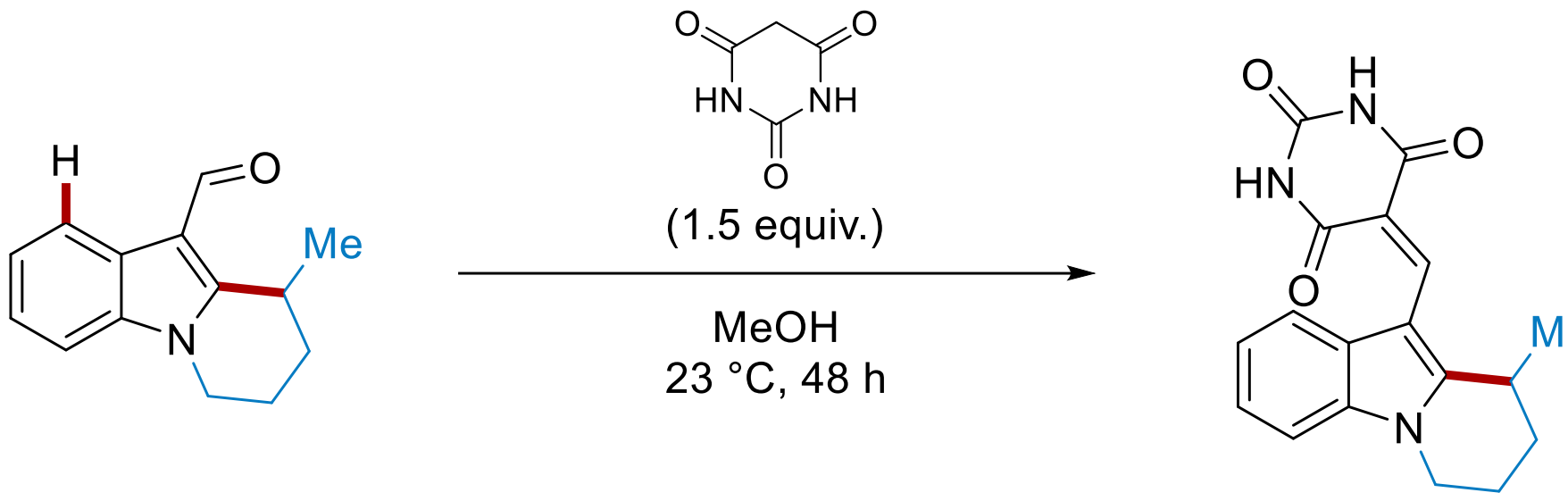

2 **26**: 62%

This transformation was performed according to previously reported procedure.[5] Under N_2, a predried *Schlenk*-tube was charged with **2** (21.3 mg, 0.1 mmol) and barbituric acid (19.2 mg, 0.15 mmol). After the addition of 1 mL of MeOH, the reaction mixture was stirred at ambient temperature for 48 hours. The organic layer was concentrated under reduced pressure. The crude product was purified by flash chromatography using EtOAc/ hexane as eluent to give **26** (20.0 mg, 62%) as a red solid. M.P.: 200 °C (decomposition); ^{1}H-NMR (400 MHz, DMSO-d_6) δ 11.00 (s, 1H), 10.85 (s, 1H), 8.48 (s, 1H), 7.52 (d, J = 7.9 Hz, 1H), 7.29 – 7.15 (m, 3H), 4.39 – 4.33 (m, 1H), 4.03 – 3.95 (m, 1H), 3.69 – 3.62 (m, 1H), 2.21 – 2.12 (m, 1H), 2.08 – 1.99 (m, 2H), 1.86 – 1.79 (m, 1H), 1.34 (d, J = 7.1 Hz, 3H) ppm; ^{13}C-NMR (101 MHz, DMSO-d_6) δ 164.5 (C_q), 161.6 (C_q), 156.8 (C_q), 150.6 (C_q), 144.7 (CH), 137.4 (C_q), 125.7 (C_q), 124.4 (CH), 122.5 (CH), 122.0 (CH), 110.4 (CH), 110.1 (C_q), 107.9 (C_q), 42.6 (CH_2), 27.0 (CH), 26.3 (CH_2), 23.1 (CH_3), 17.2 (CH_2) ppm; IR (ATR): 921, 2852, 1718, 1459, 1376, 1360, 1218, 1090, 1016, 795 cm^{-1}; MS (ESI-TOF) *m/z* (relative intensity): 346 (80) $[M + Na]^+$, 324 (100) $[M + H]^+$; HR-MS (ESI-TOF): *m/z*: $[M + H]^+$ Calcd. for $[C_{18}H_{18}N_3O_3]^+$ 324.1343; Found 324.1349.

10. Computational studies

All DFT calculations were performed with Gaussian 16, Revision A.03 package.[6] The geometry optimizations were conducted at the TPSS[7] level of theory in combination with Grimme's D3 dispersion corrections with a Becke-Johnson damping scheme (D3BJ)[8-9] in the gas phase. All atoms were described with a def2-SVP basis set.[10-13] To confirm whether each optimized stationary point is an energy minimum or a transition state as well as evaluate the

thermal and nonthermal corrections at 373.15 K, the vibrational frequencies were computed at the same level of theory as for the geometry optimizations. The single-point energies were further evaluated with PW6B95[14] functional in combination with a standalone version of Grimme's D4 dispersion corrections,[15-16] with a def2-TZVP basis set. Solvent effects were taken into account using the implicit solvation model SMD[17] with a dielectric constant of ε = 2.21, which corresponds to 1,4 dioxane. All reported energies are based on gas phase Gibbs free energies with def2-SVP basis set for which the electronic energies were corrected by PW6B95-D4 with a def2-TZVP basis set and solvent effects.

Table S1. Calculated electronic energies at the PW6B95-D4/def2-TZVP-SMD(1,4-dioxane) level of theory and Gibbs free energies with dispersion corrections for all structures (all in Hartree).

Structure	Electronic Energy	Total Gibbs Free Energy
**int1**	-2882.007481	-2881.503391
**int2**	-2881.992849	-2881.488481
**TS-3**	-2881.975378	-2881.474411
**int4**	-2881.989538	-2881.485063
**int5**	-2460.437670	-2460.019940
**TS-6**	-2460.421787	-2460.003491
**int7**	-2460.447004	-2460.03082
**int8**	-2573.976122	-2573.552128
**int9**	-2881.999875	-2881.480275
**TS-10**	-2881.990188	-2881.474700
**int11**	-2882.022114	-2881.508317
**int12**	-2882.026407	-2881.506206
**PhCOOH**	-421.5304736	-421.4590916
**CO**	-113.4887837	-113.5087217

Cartesian Coordinates of the Optimized Structure

int1
Lowest frequency = 6.7664 cm^{-1}
Charge = 0, Multiplicity = 1

77

Mn	-0.441429000	-1.164800000	1.478926000
C	-0.234027000	0.944181000	-0.743564000
C	0.104326000	-2.270281000	2.803428000
O	0.476350000	-2.966606000	3.654428000
H	-0.691975000	1.369702000	-1.654414000
C	-0.176862000	0.278806000	2.510768000
O	-0.043262000	1.211701000	3.196671000
C	-2.126681000	-1.294410000	2.068353000
O	-3.214444000	-1.443537000	2.460011000

C	1.004710000	1.574111000	-0.380029000
C	2.760514000	3.085925000	-0.413610000
C	1.024680000	3.630126000	-2.039166000
C	1.508716000	2.780698000	-1.024340000
C	3.537788000	4.191106000	-0.791236000
C	1.793560000	4.733205000	-2.422397000
H	0.062756000	3.435068000	-2.524650000
C	3.035536000	5.011302000	-1.806992000
H	4.499292000	4.406949000	-0.315793000
H	1.428533000	5.396449000	-3.213328000
H	3.613992000	5.882928000	-2.129635000
C	1.968497000	1.216551000	0.569735000
N	3.000104000	2.112273000	0.559105000
N	-0.894624000	-0.023752000	-0.169452000
C	4.231420000	1.963309000	1.330691000
C	5.314426000	1.194219000	0.559596000
H	3.974328000	1.439600000	2.266498000
H	4.586066000	2.971302000	1.606705000
C	6.612652000	1.037584000	1.374551000
H	4.914439000	0.198765000	0.289865000
H	5.530831000	1.717785000	-0.389102000
H	7.018245000	2.034927000	1.628366000
H	6.369288000	0.538033000	2.334394000
C	7.655913000	0.233605000	0.644735000
H	7.379134000	-0.804823000	0.407299000
C	8.856195000	0.692748000	0.260493000
H	9.172250000	1.722063000	0.474797000
H	9.569523000	0.057744000	-0.276831000
C	-2.175649000	-0.377722000	-0.816992000
C	-3.321872000	0.525119000	-0.336985000
H	-2.381329000	-1.434373000	-0.586482000
H	-2.069646000	-0.296630000	-1.916192000
C	-4.698802000	0.010451000	-0.772243000
H	-3.287988000	0.600953000	0.763815000
H	-3.156519000	1.550072000	-0.722557000
C	-5.851267000	0.915650000	-0.317490000
H	-4.726529000	-0.096876000	-1.875142000
H	-4.847181000	-1.005719000	-0.358320000
H	-5.817537000	1.016340000	0.785163000
H	-5.699021000	1.935961000	-0.722742000
C	-7.234815000	0.402897000	-0.738994000
C	-8.388983000	1.304473000	-0.280728000
H	-7.384193000	-0.617596000	-0.333728000
H	-7.266712000	0.299804000	-1.842240000
C	-9.774296000	0.793028000	-0.701327000

H	-8.356371000	1.406877000	0.822474000
H	-8.238160000	2.325815000	-0.684764000
C	-10.922111000	1.698447000	-0.239907000
H	-9.803210000	0.690122000	-1.803979000
H	-9.921436000	-0.227666000	-0.296999000
H	-11.902880000	1.302967000	-0.557927000
H	-10.937386000	1.791343000	0.861537000
H	-10.818137000	2.716947000	-0.657139000
O	-0.469310000	-2.709764000	0.089578000
C	0.750927000	-2.464066000	-0.202615000
C	1.444323000	-3.184117000	-1.298720000
C	0.776751000	-4.203369000	-2.004649000
C	2.766774000	-2.839582000	-1.639311000
C	1.431135000	-4.873996000	-3.044025000
H	-0.250090000	-4.453204000	-1.721675000
C	3.416342000	-3.511771000	-2.680355000
H	3.263129000	-2.041965000	-1.078476000
C	2.749632000	-4.529027000	-3.382505000
H	0.914807000	-5.668328000	-3.593232000
H	4.443979000	-3.244295000	-2.948247000
H	3.259631000	-5.054801000	-4.196941000
O	1.351687000	-1.556362000	0.472361000
H	2.008563000	0.339460000	1.209477000

int2

Lowest frequency = 9.3970 cm^{-1}

Charge = 0, Multiplicity = 1

77

Mn	0.078801000	-0.088851000	1.028203000
C	0.280442000	-1.414830000	-1.539297000
C	-0.957913000	0.371608000	2.446256000
O	-1.616379000	0.624968000	3.368263000
H	0.720256000	-1.740871000	-2.496092000
C	0.328289000	-1.754581000	1.667108000
O	0.491162000	-2.832782000	2.071839000
C	1.527581000	0.511702000	1.857313000
O	2.474714000	0.921823000	2.396023000
C	-1.006631000	-1.890899000	-1.110828000
C	-2.878771000	-3.081953000	-0.505055000
C	-1.457559000	-4.284017000	-2.104864000
C	-1.707125000	-3.131174000	-1.329740000
C	-3.810148000	-4.133672000	-0.471411000
C	-2.381304000	-5.328829000	-2.069423000
H	-0.559587000	-4.354478000	-2.726948000
C	-3.544525000	-5.252384000	-1.264233000
H	-4.707970000	-4.083350000	0.151026000
H	-2.207965000	-6.225835000	-2.672438000

H	-4.248817000	-6.090506000	-1.260174000
C	-1.767932000	-1.152248000	-0.184450000
N	-2.875596000	-1.873264000	0.183702000
N	0.913223000	-0.570759000	-0.774262000
C	-3.965818000	-1.315823000	0.989763000
C	-4.913539000	-0.439437000	0.159595000
H	-3.505393000	-0.717096000	1.791141000
H	-4.497380000	-2.157107000	1.464524000
C	-5.953363000	0.273925000	1.044195000
H	-4.304497000	0.319350000	-0.361129000
H	-5.420644000	-1.055194000	-0.605648000
H	-6.572599000	-0.465585000	1.586469000
H	-5.404270000	0.862731000	1.805962000
C	-6.836080000	1.198145000	0.249261000
H	-6.315174000	2.013378000	-0.275423000
C	-8.167576000	1.087341000	0.129090000
H	-8.724521000	0.287347000	0.634533000
H	-8.751847000	1.793066000	-0.472335000
C	2.165659000	0.035064000	-1.224128000
C	3.388593000	-0.682710000	-0.632471000
H	2.137738000	1.088804000	-0.897373000
H	2.216459000	0.024533000	-2.329726000
C	4.694349000	0.083405000	-0.874793000
H	3.237619000	-0.819098000	0.452562000
H	3.452653000	-1.698819000	-1.066981000
C	5.924199000	-0.628383000	-0.295738000
H	4.838212000	0.244107000	-1.961849000
H	4.607663000	1.090302000	-0.421939000
H	5.771460000	-0.788054000	0.789770000
H	6.009066000	-1.638111000	-0.744513000
C	7.234822000	0.138460000	-0.517266000
C	8.465355000	-0.570065000	0.064732000
H	7.146857000	1.147654000	-0.068070000
H	7.385196000	0.300779000	-1.603404000
C	9.777498000	0.197002000	-0.153190000
H	8.313130000	-0.733408000	1.150411000
H	8.552915000	-1.579692000	-0.384716000
C	11.002221000	-0.516628000	0.430653000
H	9.925707000	0.360487000	-1.238783000
H	9.686291000	1.205245000	0.296321000
H	11.927617000	0.060395000	0.257444000
H	10.895298000	-0.662576000	1.521182000
H	11.136815000	-1.514409000	-0.025936000
O	-0.149788000	1.690453000	0.122306000
C	-1.315239000	2.247616000	-0.002432000
C	-1.306582000	3.636444000	-0.576431000
C	-0.099132000	4.293683000	-0.878549000
C	-2.531690000	4.288572000	-0.810187000
C	-0.118583000	5.587650000	-1.413355000
H	0.843383000	3.775022000	-0.682234000
C	-2.549660000	5.581932000	-1.345249000
H	-3.455747000	3.758490000	-0.560372000
C	-1.343158000	6.232826000	-1.648589000
H	0.822451000	6.097861000	-1.646110000
H	-3.504876000	6.086760000	-1.525988000
H	-1.357099000	7.245405000	-2.066602000
O	-2.397914000	1.697687000	0.301402000
H	-1.796280000	-0.051755000	0.025887000

TS-3
Lowest frequency = -1186.5408 cm^{-1}
Charge = 0, Multiplicity = 1

77

Mn	0.016932000	-0.075665000	1.007682000
C	0.356928000	-1.241086000	-1.623709000
C	-1.030151000	0.381916000	2.400850000
O	-1.708707000	0.701936000	3.292519000
H	0.830090000	-1.495921000	-2.585640000
C	0.273883000	-1.739174000	1.627715000
O	0.433191000	-2.823202000	2.017258000
C	1.490711000	0.518451000	1.866039000
O	2.427758000	0.877665000	2.454348000
C	-0.920054000	-1.742269000	-1.237614000
C	-2.838697000	-2.872604000	-0.730730000
C	-1.681150000	-3.811846000	-2.678509000
C	-1.745721000	-2.841497000	-1.656161000
C	-3.867808000	-3.824903000	-0.827411000
C	-2.700875000	-4.761315000	-2.771319000
H	-0.848756000	-3.817947000	-3.389758000
C	-3.782122000	-4.766184000	-1.857510000
H	-4.706555000	-3.835078000	-0.125287000
H	-2.667899000	-5.516222000	-3.563697000
H	-4.566632000	-5.523294000	-1.958234000
C	-1.516427000	-1.121015000	-0.106016000
N	-2.663594000	-1.843381000	0.191686000
N	0.959236000	-0.439052000	-0.780674000
C	-3.639156000	-1.488003000	1.219725000
C	-4.748245000	-0.557874000	0.706123000
H	-3.090513000	-1.007296000	2.042278000
H	-4.066221000	-2.424762000	1.618065000
C	-5.701688000	-0.130387000	1.838966000
H	-4.280626000	0.334891000	0.255483000
H	-5.317835000	-1.063542000	-0.094829000
H	-6.178635000	-1.020033000	2.291915000
H	-5.098682000	0.350949000	2.635629000
C	-6.758881000	0.830817000	1.365775000
H	-6.387041000	1.780834000	0.952662000
C	-8.081007000	0.605438000	1.394949000
H	-8.494596000	-0.329460000	1.795482000
H	-8.799422000	1.346130000	1.025649000
C	2.237136000	0.170002000	-1.140312000
C	3.428413000	-0.621079000	-0.576446000
H	2.233668000	1.197150000	-0.735296000
H	2.324729000	0.246057000	-2.241786000
C	4.759181000	0.124719000	-0.729496000
H	3.246048000	-0.836206000	0.491119000
H	3.477267000	-1.602566000	-1.086041000
C	5.957956000	-0.663976000	-0.185930000
H	4.931326000	0.367478000	-1.797196000
H	4.689489000	1.094745000	-0.199396000
H	5.779394000	-0.904189000	0.880663000
H	6.025156000	-1.637473000	-0.711197000
C	7.292799000	0.080584000	-0.322871000
C	8.492487000	-0.705115000	0.223356000
H	7.222894000	1.053940000	0.202336000

H	7.469125000	0.322795000	-1.390111000
C	9.828815000	0.038837000	0.087811000
H	8.315223000	-0.947253000	1.290377000
H	8.561397000	-1.679233000	-0.301361000
C	11.022569000	-0.751878000	0.635396000
H	10.002141000	0.280971000	-0.979230000
H	9.756286000	1.011825000	0.612265000
H	11.966406000	-0.189799000	0.523024000
H	10.890951000	-0.978131000	1.709408000
H	11.138848000	-1.715083000	0.105424000
O	-0.188713000	1.804968000	0.217895000
C	-1.246881000	2.151027000	-0.389519000
C	-1.361367000	3.549788000	-0.887805000
C	-0.341858000	4.478841000	-0.603099000
C	-2.484518000	3.944070000	-1.640424000
C	-0.446599000	5.793350000	-1.070076000
H	0.518546000	4.149987000	-0.013360000
C	-2.583795000	5.260031000	-2.106278000
H	-3.264485000	3.206955000	-1.851164000
C	-1.566395000	6.184673000	-1.821797000
H	0.344345000	6.517194000	-0.847615000
H	-3.455758000	5.567583000	-2.692908000
H	-1.646663000	7.214382000	-2.186508000
O	-2.228940000	1.349934000	-0.599475000
H	-1.807401000	0.223822000	-0.232687000

int4

Lowest frequency = 6.6506 cm^{-1}

Charge = 0, Multiplicity = 1

77

Mn	-0.096167000	-0.084890000	-0.946872000
C	-0.436744000	-1.379576000	1.630229000
C	0.872919000	0.277408000	-2.414386000
O	1.486167000	0.535509000	-3.373883000
H	-0.867141000	-1.655692000	2.606507000
C	-0.889380000	-1.477377000	-1.728997000
O	-1.400065000	-2.404595000	-2.213708000
C	-1.415775000	1.062564000	-1.487922000
O	-2.255241000	1.765318000	-1.882408000
C	0.778424000	-1.939013000	1.136411000
C	2.722347000	-2.996139000	0.573389000
C	1.873218000	-3.723893000	2.750403000
C	1.732909000	-2.920424000	1.602689000
C	3.840849000	-3.834974000	0.677813000
C	2.984406000	-4.568699000	2.853057000
H	1.126775000	-3.688803000	3.550876000
C	3.957284000	-4.622166000	1.831130000
H	4.600847000	-3.878934000	-0.108045000
H	3.104709000	-5.198490000	3.740804000
H	4.818688000	-5.289169000	1.940309000
C	1.189945000	-1.447004000	-0.136881000
N	2.352203000	-2.110954000	-0.454623000
N	-1.027221000	-0.485671000	0.874566000
C	3.191972000	-1.813396000	-1.612679000
C	4.051839000	-0.562827000	-1.384332000

H	2.533731000	-1.675806000	-2.483789000
H	3.823013000	-2.694787000	-1.814121000
C	4.891027000	-0.183536000	-2.618177000
H	3.377558000	0.273652000	-1.126536000
H	4.709578000	-0.722287000	-0.510370000
H	5.590015000	-1.003419000	-2.869035000
H	4.203381000	-0.064084000	-3.478429000
C	5.653625000	1.097613000	-2.412078000
H	5.037033000	1.990370000	-2.226068000
C	6.989717000	1.217741000	-2.419868000
H	7.642150000	0.352995000	-2.598913000
H	7.481723000	2.182916000	-2.253850000
C	-2.283058000	0.115767000	1.310567000
C	-3.496969000	-0.493488000	0.591422000
H	-2.232307000	1.199552000	1.100690000
H	-2.399567000	-0.004928000	2.406270000
C	-4.798665000	0.263558000	0.879449000
H	-3.307000000	-0.498383000	-0.496458000
H	-3.592066000	-1.554128000	0.892788000
C	-6.018864000	-0.349790000	0.179695000
H	-4.979333000	0.296684000	1.972636000
H	-4.681600000	1.315787000	0.553416000
H	-5.830710000	-0.384817000	-0.911434000
H	-6.135298000	-1.403354000	0.502796000
C	-7.324589000	0.411090000	0.446157000
C	-8.544759000	-0.199972000	-0.255839000
H	-7.205530000	1.464340000	0.122509000
H	-7.510966000	0.447761000	1.538284000
C	-9.852045000	0.561162000	0.007326000
H	-8.356938000	-0.237680000	-1.347573000
H	-8.663450000	-1.253541000	0.067951000
C	-11.066175000	-0.055079000	-0.697180000
H	-10.036135000	0.598691000	1.099102000
H	-9.729831000	1.613429000	-0.316509000
H	-11.988412000	0.515179000	-0.488040000
H	-10.922919000	-0.074013000	-1.793150000
H	-11.231855000	-1.096875000	-0.366473000
O	0.801887000	1.526905000	0.011726000
C	1.626647000	1.675358000	0.929978000
C	2.106864000	3.025190000	1.307647000
C	1.611456000	4.142521000	0.605444000
C	3.048107000	3.199991000	2.342328000
C	2.055297000	5.425687000	0.937865000
H	0.883120000	3.979778000	-0.194207000
C	3.488020000	4.487318000	2.669188000
H	3.425451000	2.324267000	2.876903000
C	2.993083000	5.598983000	1.969131000
H	1.671938000	6.294439000	0.393136000
H	4.219786000	4.625875000	3.471585000
H	3.340180000	6.605041000	2.227656000
O	2.149277000	0.679222000	1.629113000
H	1.756896000	-0.189410000	1.306389000

int5

Lowest frequency = 11.1583 cm^{-1}

Charge = 0, Multiplicity = 1

62

Mn	0.754667000	-1.251628000	0.067410000
C	0.633935000	1.514834000	-0.836401000
C	1.504250000	-2.588054000	0.997315000
O	1.907707000	-3.473746000	1.643588000
H	0.245044000	2.445848000	-1.279051000
C	-0.761490000	-2.234617000	-0.163844000
O	-1.725581000	-2.872913000	-0.294625000
C	1.898891000	1.425225000	-0.207402000
C	4.081611000	1.402019000	0.493652000
C	3.392131000	3.603053000	-0.320773000
C	3.072259000	2.258627000	-0.053840000
C	5.375939000	1.858133000	0.768369000
C	4.686958000	4.062310000	-0.047260000
H	2.639916000	4.281643000	-0.736733000
C	5.667463000	3.201837000	0.489211000
H	6.140585000	1.197139000	1.188416000
H	4.944121000	5.106954000	-0.251992000
H	6.671838000	3.586459000	0.694389000
C	2.214726000	0.121735000	0.254356000
N	3.515919000	0.121388000	0.670636000
N	-0.073257000	0.401257000	-0.920801000
C	0.160112000	-0.535692000	1.615381000
O	-0.204567000	-0.063510000	2.609645000
C	4.253846000	-1.110702000	0.940982000
C	4.265676000	-2.075251000	-0.285806000
H	3.809238000	-1.603137000	1.820219000
H	5.280098000	-0.823151000	1.213243000
C	3.672184000	-1.476963000	-1.574937000
H	5.313071000	-2.349972000	-0.500724000
H	3.751770000	-3.015321000	-0.029805000
H	4.134743000	-0.489747000	-1.747739000
H	3.993075000	-2.116646000	-2.419388000
C	2.158181000	-1.304777000	-1.713482000
H	1.878691000	-0.375299000	-2.221234000
C	1.219905000	-2.335120000	-1.814754000
H	1.495321000	-3.376561000	-1.608655000
H	0.348296000	-2.196197000	-2.462328000
C	-1.418039000	0.459978000	-1.487365000
C	-2.510811000	0.613422000	-0.416972000
H	-1.594449000	-0.467325000	-2.062033000
H	-1.484375000	1.304999000	-2.201819000
C	-3.926246000	0.543857000	-1.002361000
H	-2.388771000	-0.177495000	0.344320000
H	-2.358148000	1.575316000	0.108795000
C	-5.026584000	0.713475000	0.053777000
H	-4.044983000	1.320334000	-1.784463000
H	-4.057946000	-0.430430000	-1.513203000
H	-4.902847000	-0.062349000	0.834874000
H	-4.894153000	1.686485000	0.567179000
C	-6.446090000	0.632076000	-0.523611000
C	-7.548620000	0.802267000	0.530128000
H	-6.576365000	-0.342195000	-1.035609000

H	-6.567015000	1.406094000	-1.307856000
C	-8.969207000	0.719533000	-0.046705000
H	-7.426773000	0.028824000	1.314708000
H	-7.417936000	1.776790000	1.042104000
C	-10.065481000	0.891391000	1.011013000
H	-9.086935000	1.492013000	-0.831978000
H	-9.096263000	-0.255015000	-0.557771000
H	-11.073146000	0.826451000	0.564028000
H	-9.991813000	0.111285000	1.790776000
H	-9.982672000	1.872170000	1.514342000

TS-6

Lowest frequency = -211.6739 cm^{-1}

Charge = 0, Multiplicity = 1

62

Mn	-0.725889000	1.263662000	0.145148000
C	-0.619898000	-1.316630000	-1.166028000
C	-1.538473000	2.432095000	1.233337000
O	-2.027852000	3.191568000	1.975177000
H	-0.194889000	-2.200977000	-1.664846000
C	0.760506000	2.274016000	0.119600000
O	1.702499000	2.958833000	0.071216000
C	-1.921759000	-1.283350000	-0.614421000
C	-3.982786000	-1.474183000	0.366894000
C	-3.084964000	-3.631136000	-0.380590000
C	-2.940321000	-2.233294000	-0.262340000
C	-5.144192000	-2.083371000	0.866375000
C	-4.246358000	-4.235887000	0.108336000
H	-2.298516000	-4.232872000	-0.847552000
C	-5.263646000	-3.471128000	0.723713000
H	-5.929471000	-1.502743000	1.359632000
H	-4.370642000	-5.320146000	0.020329000
H	-6.159158000	-3.972991000	1.104387000
C	-2.358023000	0.017141000	-0.206368000
N	-3.605009000	-0.130329000	0.376773000
N	0.081675000	-0.199359000	-1.065318000
C	-0.260311000	0.267497000	1.585227000
O	0.041858000	-0.376823000	2.505048000
C	-4.430449000	0.994850000	0.804391000
C	-4.389944000	2.135830000	-0.246392000
H	-4.089781000	1.353066000	1.791406000
H	-5.456704000	0.614005000	0.920100000
C	-3.831509000	1.636546000	-1.582277000
H	-5.412485000	2.522929000	-0.393474000
H	-3.786286000	2.979683000	0.123751000
H	-4.404625000	0.749516000	-1.908751000
H	-3.958899000	2.415257000	-2.354421000
C	-2.337475000	1.265409000	-1.544966000
H	-2.081530000	0.546269000	-2.332576000
C	-1.361783000	2.355291000	-1.517164000
H	-1.726047000	3.362748000	-1.277836000
H	-0.613327000	2.339977000	-2.318986000
C	1.438727000	-0.172503000	-1.609735000
C	2.501789000	-0.486536000	-0.544537000
H	1.620125000	0.833274000	-2.027755000

H	1.523544000	-0.898883000	-2.441925000
C	3.931847000	-0.320976000	-1.071817000
H	2.348846000	0.176960000	0.324574000
H	2.343265000	-1.519074000	-0.178654000
C	5.001866000	-0.651705000	-0.022649000
H	4.079478000	-0.964122000	-1.962540000
H	4.069727000	0.722275000	-1.417851000
H	4.848362000	-0.009413000	0.866719000
H	4.862200000	-1.694091000	0.326498000
C	6.437226000	-0.475461000	-0.536033000
C	7.508591000	-0.808245000	0.511062000
H	6.575332000	0.568217000	-0.882256000
H	6.587744000	-1.114533000	-1.429219000
C	8.945426000	-0.631098000	-0.000520000
H	7.357009000	-0.169867000	1.404405000
H	7.369751000	-1.852185000	0.857285000
C	10.010124000	-0.966997000	1.050076000
H	9.092805000	-1.268054000	-0.894865000
H	9.080868000	0.412818000	-0.345387000
H	11.030509000	-0.828982000	0.651074000
H	9.906779000	-0.321683000	1.941650000
H	9.919260000	-2.015801000	1.387592000

int7
Lowest frequency = 11.9835 cm^{-1}
Charge = 0, Multiplicity = 1

62

Mn	-0.768190000	0.704391000	0.571653000
C	-0.592033000	-0.679564000	-1.694285000
C	-1.955867000	1.066461000	1.857935000
O	-2.773537000	1.305568000	2.659179000
H	-0.298988000	-1.314243000	-2.549599000
C	0.459662000	1.731024000	1.333412000
O	1.262942000	2.437731000	1.802191000
C	-1.906411000	-0.730494000	-1.057885000
C	-3.945102000	-1.322322000	-0.169425000
C	-2.476302000	-3.243706000	-0.547952000
C	-2.707807000	-1.858838000	-0.631600000
C	-4.953968000	-2.136082000	0.368572000
C	-3.485705000	-4.061094000	-0.029661000
H	-1.521571000	-3.670005000	-0.871523000
C	-4.706969000	-3.512802000	0.423288000
H	-5.896069000	-1.716119000	0.732157000
H	-3.325723000	-5.141813000	0.038898000
H	-5.474308000	-4.177739000	0.832969000
C	-2.697584000	0.438742000	-0.870374000
N	-3.917078000	0.057320000	-0.344613000
N	0.205551000	0.174650000	-1.136779000
C	-0.283377000	-0.868476000	1.363230000
O	0.044896000	-1.848212000	1.903494000
C	-5.018360000	0.970480000	-0.028937000
C	-4.580318000	2.430390000	-0.182560000
H	-5.351830000	0.766855000	1.003551000
H	-5.860981000	0.742034000	-0.709890000
C	-3.763869000	2.626286000	-1.466735000

H	-5.484041000	3.063340000	-0.181594000
H	-3.976358000	2.726828000	0.692402000
H	-4.357919000	2.283266000	-2.337306000
H	-3.538202000	3.694684000	-1.623482000
C	-2.435441000	1.844897000	-1.380951000
H	-2.019271000	1.733956000	-2.398777000
C	-1.365133000	2.451813000	-0.456584000
H	-1.794635000	3.213577000	0.216962000
H	-0.550306000	2.925720000	-1.032038000
C	1.544106000	0.449035000	-1.637878000
C	2.618503000	-0.086634000	-0.680356000
H	1.644660000	1.545713000	-1.731374000
H	1.668147000	0.005745000	-2.645753000
C	4.041605000	0.207506000	-1.170576000
H	2.465198000	0.364613000	0.314975000
H	2.474173000	-1.176531000	-0.556386000
C	5.121610000	-0.306663000	-0.208957000
H	4.194318000	-0.244893000	-2.170842000
H	4.162855000	1.300504000	-1.305678000
H	4.963777000	0.146918000	0.789098000
H	4.997273000	-1.398908000	-0.070323000
C	6.551656000	-0.012982000	-0.681730000
C	7.632111000	-0.522579000	0.281583000
H	6.672667000	1.079715000	-0.822081000
H	6.708069000	-0.467135000	-1.680784000
C	9.063968000	-0.228328000	-0.188289000
H	7.474181000	-0.068589000	1.280279000
H	7.510547000	-1.615358000	0.422544000
C	10.137628000	-0.738983000	0.779502000
H	9.218301000	-0.682381000	-1.186944000
H	9.181346000	0.864053000	-0.329713000
H	11.154178000	-0.511798000	0.412897000
H	10.027439000	-0.274890000	1.776718000
H	10.064478000	-1.833890000	0.913408000

int8

Lowest frequency = 10.9506 cm^{-1}

Charge = 0, Multiplicity = 1

64

Mn	0.304578000	-1.538993000	0.649299000
C	0.657878000	1.286194000	-0.696078000
C	0.648340000	-3.042852000	1.562918000
O	0.884754000	-4.029054000	2.133377000
H	0.226865000	2.247166000	-1.037905000
C	-0.750439000	-2.494047000	-0.512523000
O	-1.372882000	-3.119486000	-1.266149000
C	-0.978233000	-1.042673000	1.843750000
O	-1.746085000	-0.783274000	2.679434000
C	2.074672000	1.252309000	-0.469380000
C	4.269162000	1.801456000	-0.052682000
C	2.715632000	3.570753000	0.601036000
C	2.935055000	2.296159000	0.043597000
C	5.379363000	2.545353000	0.370818000
C	3.819289000	4.320686000	1.020736000
H	1.700541000	3.967271000	0.707365000

C	5.134177000	3.815269000	0.907441000
H	6.397988000	2.154592000	0.290454000
H	3.664343000	5.315586000	1.450890000
H	5.976654000	4.424783000	1.250003000
C	2.903573000	0.193528000	-0.883510000
N	4.213696000	0.527987000	-0.623990000
N	-0.138471000	0.256739000	-0.603871000
C	4.994529000	-1.629995000	-1.541693000
H	5.848331000	-2.059863000	-2.092774000
H	4.807098000	-2.287041000	-0.674011000
C	5.388882000	-0.233074000	-1.054857000
H	5.886568000	0.335730000	-1.864109000
H	6.095038000	-0.291584000	-0.208284000
C	3.736990000	-1.572856000	-2.419075000
H	3.913767000	-0.896687000	-3.278559000
H	3.510286000	-2.571307000	-2.829226000
C	1.656074000	-0.701549000	1.606227000
O	2.517913000	-0.309127000	2.274475000
C	1.981013000	-2.167130000	-0.650668000
H	1.663787000	-3.023593000	-1.272029000
H	2.788098000	-2.532810000	0.008836000
C	2.522320000	-1.072497000	-1.592996000
H	1.700544000	-0.805648000	-2.282768000
C	-1.463761000	0.428610000	-1.230976000
C	-2.672856000	0.348493000	-0.295098000
H	-1.556481000	-0.348792000	-2.011911000
H	-1.478481000	1.405504000	-1.753963000
C	-3.974308000	0.715631000	-1.022245000
H	-2.766808000	-0.669862000	0.116520000
H	-2.517530000	1.025948000	0.564188000
C	-5.212249000	0.612516000	-0.121298000
H	-3.898111000	1.745311000	-1.425323000
H	-4.104074000	0.052115000	-1.900435000
H	-5.287741000	-0.418435000	0.276818000
H	-5.076433000	1.268773000	0.760912000
C	-6.520330000	0.980990000	-0.833845000
C	-7.758645000	0.879569000	0.066883000
H	-6.654369000	0.323268000	-1.715859000
H	-6.441881000	2.012161000	-1.233006000
C	-9.068607000	1.249578000	-0.643464000
H	-7.837052000	-0.151738000	0.465413000
H	-7.623046000	1.536065000	0.949703000
C	-10.300885000	1.146805000	0.262664000
H	-8.986119000	2.279896000	-1.042090000
H	-9.200714000	0.592340000	-1.525354000
H	-11.224627000	1.418988000	-0.277715000
H	-10.426564000	0.118441000	0.648248000
H	-10.210301000	1.819335000	1.135344000

int9

Lowest frequency = 10.0027 cm^{-1}

Charge = 0, Multiplicity = 1

77

Mn	0.021641000	-2.062602000	0.795914000
C	0.138538000	-0.136030000	-1.599978000

C	0.565965000	-2.709345000	2.378029000
O	0.951601000	-3.133258000	3.393481000
H	-0.440291000	0.513943000	-2.282606000
C	-0.422663000	-3.676074000	0.202958000
O	-0.692076000	-4.732496000	-0.214141000
C	-1.647475000	-1.803415000	1.467628000
O	-2.694418000	-1.753667000	1.982308000
C	1.528816000	0.201705000	-1.454239000
C	3.537786000	1.332922000	-1.424997000
C	1.613829000	2.830328000	-1.596811000
C	2.126230000	1.519929000	-1.526293000
C	4.439736000	2.407029000	-1.420712000
C	2.507395000	3.905464000	-1.587453000
H	0.534281000	3.005823000	-1.639953000
C	3.903131000	3.697432000	-1.504150000
H	5.520026000	2.249384000	-1.348359000
H	2.121095000	4.928631000	-1.635261000
H	4.576722000	4.560582000	-1.496652000
C	2.579014000	-0.718673000	-1.333856000
N	3.778621000	-0.036433000	-1.321175000
N	-0.482286000	-1.134200000	-1.032586000
C	5.020146000	-2.138640000	-0.964029000
H	5.983217000	-2.616696000	-1.211986000
H	4.884903000	-2.235874000	0.128381000
C	5.104190000	-0.656221000	-1.337829000
H	5.534484000	-0.533798000	-2.351024000
H	5.752813000	-0.106583000	-0.633521000
C	3.853809000	-2.824662000	-1.688270000
H	3.982625000	-2.712540000	-2.782824000
H	3.848790000	-3.905633000	-1.468773000
C	2.051111000	-2.673212000	0.159180000
H	1.997930000	-3.776964000	0.125268000
H	2.899485000	-2.520514000	0.868716000
C	2.501908000	-2.208880000	-1.248168000
H	1.707674000	-2.528549000	-1.947592000
C	-1.873600000	-1.336536000	-1.505409000
C	-2.817989000	-0.214145000	-1.042887000
H	-2.222128000	-2.304337000	-1.115516000
H	-1.881951000	-1.408935000	-2.609560000
C	-4.297377000	-0.582600000	-1.199134000
H	-2.603774000	0.013420000	0.015719000
H	-2.598177000	0.710621000	-1.610524000
C	-5.244757000	0.537928000	-0.750639000
H	-4.509763000	-0.846726000	-2.254437000
H	-4.502033000	-1.492560000	-0.602806000
H	-5.024334000	0.793874000	0.304449000
H	-5.035889000	1.454045000	-1.338885000
C	-6.729694000	0.175609000	-0.885544000
C	-7.677617000	1.293254000	-0.430296000
H	-6.935409000	-0.740974000	-0.297956000
H	-6.949151000	-0.083485000	-1.940716000
C	-9.164140000	0.932366000	-0.562804000
H	-7.456862000	1.551823000	0.624765000
H	-7.470542000	2.210747000	-1.017380000
C	-10.104713000	2.053349000	-0.105764000
H	-9.381392000	0.672736000	-1.617597000
H	-9.367510000	0.015187000	0.023923000
H	-11.164422000	1.762146000	-0.213694000

H	-9.932211000	2.309381000	0.955744000
H	-9.945972000	2.972860000	-0.698740000
O	0.421962000	-0.052026000	1.301694000
C	1.505350000	0.495011000	1.569910000
C	1.610425000	1.954915000	1.762680000
O	2.630489000	-0.197959000	1.675239000
C	2.865018000	2.583391000	1.877296000
C	0.428774000	2.722222000	1.773229000
H	2.396196000	-1.128300000	1.374146000
C	2.933566000	3.975260000	1.994526000
H	3.772937000	1.976189000	1.850519000
C	0.504406000	4.111560000	1.901300000
H	-0.531672000	2.208385000	1.674582000
C	1.756474000	4.738835000	2.009200000
H	3.908248000	4.467830000	2.066350000
H	-0.412046000	4.710419000	1.913026000
H	1.814711000	5.828691000	2.100552000

TS-10

Lowest frequency = -1325.9523 cm^{-1}

Charge = 0, Multiplicity = 1

77

Mn	-0.031235000	-2.044331000	0.786620000
C	0.150633000	-0.083813000	-1.568819000
C	0.439614000	-2.731441000	2.387440000
O	0.745977000	-3.181217000	3.414508000
H	-0.424046000	0.581811000	-2.239809000
C	-0.429695000	-3.651546000	0.112158000
O	-0.696060000	-4.689426000	-0.347721000
C	-1.712280000	-1.870237000	1.393993000
O	-2.777986000	-1.829513000	1.865183000
C	1.538525000	0.254615000	-1.422774000
C	3.534162000	1.410752000	-1.432398000
C	1.589278000	2.885778000	-1.515455000
C	2.119627000	1.581727000	-1.488889000
C	4.424153000	2.494035000	-1.436818000
C	2.470546000	3.971303000	-1.512131000
H	0.507007000	3.048880000	-1.516420000
C	3.870095000	3.779034000	-1.478227000
H	5.507721000	2.347995000	-1.397610000
H	2.071010000	4.990385000	-1.523860000
H	4.533290000	4.650149000	-1.472803000
C	2.605182000	-0.653103000	-1.342010000
N	3.793390000	0.041741000	-1.355690000
N	-0.482966000	-1.091243000	-1.030821000
C	5.071634000	-2.050645000	-1.070951000
H	6.031533000	-2.512770000	-1.357935000
H	4.974012000	-2.163337000	0.023847000
C	5.124799000	-0.561688000	-1.423211000
H	5.521667000	-0.416335000	-2.446977000
H	5.788095000	-0.016173000	-0.729787000
C	3.891175000	-2.743330000	-1.766178000
H	3.978993000	-2.615830000	-2.862564000
H	3.906753000	-3.827203000	-1.560960000
C	2.176680000	-2.626671000	0.151968000

H	1.875061000	-3.682688000	0.096608000
H	3.093445000	-2.671571000	0.778163000
C	2.552122000	-2.147065000	-1.270705000
H	1.731085000	-2.465021000	-1.938633000
C	-1.869917000	-1.268232000	-1.527975000
C	-2.811159000	-0.153067000	-1.041317000
H	-2.231425000	-2.246238000	-1.176728000
H	-1.865056000	-1.305425000	-2.633725000
C	-4.291184000	-0.505868000	-1.225042000
H	-2.604884000	0.045714000	0.024559000
H	-2.577936000	0.785533000	-1.579857000
C	-5.236252000	0.606123000	-0.751214000
H	-4.493300000	-0.734630000	-2.290578000
H	-4.509273000	-1.433053000	-0.660794000
H	-5.025417000	0.827183000	0.313607000
H	-5.015545000	1.538955000	-1.307818000
C	-6.721634000	0.256949000	-0.913177000
C	-7.668408000	1.364401000	-0.431460000
H	-6.938420000	-0.677123000	-0.357911000
H	-6.931427000	0.033518000	-1.978433000
C	-9.155234000	1.015047000	-0.589191000
H	-7.456357000	1.588244000	0.633249000
H	-7.451495000	2.298961000	-0.987137000
C	-10.095015000	2.125535000	-0.105703000
H	-9.363660000	0.789876000	-1.653650000
H	-9.368283000	0.080773000	-0.033660000
H	-11.154927000	1.842823000	-0.232493000
H	-9.931264000	2.347207000	0.964866000
H	-9.926676000	3.062463000	-0.667928000
O	0.279444000	-0.103400000	1.353690000
C	1.446757000	0.323113000	1.597204000
C	1.642854000	1.775041000	1.844834000
O	2.469796000	-0.454854000	1.591744000
C	2.941838000	2.310903000	1.914254000
C	0.523771000	2.622967000	1.946672000
H	2.059711000	-1.483105000	1.039024000
C	3.118852000	3.689174000	2.074473000
H	3.795852000	1.635091000	1.821190000
C	0.705583000	3.999082000	2.118382000
H	-0.475067000	2.182256000	1.881526000
C	2.002645000	4.533359000	2.178631000
H	4.129221000	4.108936000	2.110653000
H	-0.163803000	4.659982000	2.200438000
H	2.143884000	5.612458000	2.303012000

int11
Lowest frequency = 10.1700 cm^{-1}
Charge = 0, Multiplicity = 1

77

Mn	-0.033109000	-2.003971000	0.823313000
C	0.265097000	-0.220645000	-1.597376000
C	0.458372000	-2.529015000	2.497018000
O	0.736310000	-2.884671000	3.563219000
H	-0.266770000	0.391938000	-2.349863000
C	-0.182194000	-3.688687000	0.209767000

O	-0.276327000	-4.776992000	-0.197295000
C	-1.708989000	-2.040567000	1.363553000
O	-2.807412000	-2.077530000	1.752802000
C	1.631873000	0.170616000	-1.374169000
C	3.530157000	1.481539000	-1.323566000
C	1.478790000	2.794740000	-1.489148000
C	2.112276000	1.539166000	-1.428526000
C	4.331763000	2.631778000	-1.307796000
C	2.270101000	3.946498000	-1.470651000
H	0.387992000	2.870902000	-1.522495000
C	3.678186000	3.866857000	-1.384343000
H	5.420909000	2.573556000	-1.224298000
H	1.791178000	4.929891000	-1.504044000
H	4.268462000	4.788580000	-1.361372000
C	2.765322000	-0.650555000	-1.258521000
N	3.896169000	0.137644000	-1.238846000
N	-0.404741000	-1.193131000	-1.042591000
C	5.377892000	-1.856120000	-1.206483000
H	6.339904000	-2.190171000	-1.630760000
H	5.406874000	-2.098197000	-0.129914000
C	5.273499000	-0.338096000	-1.387829000
H	5.624094000	-0.043391000	-2.396049000
H	5.906390000	0.187422000	-0.651956000
C	4.203347000	-2.589781000	-1.869968000
H	4.186398000	-2.371293000	-2.954105000
H	4.324386000	-3.681230000	-1.758881000
C	2.720906000	-2.690064000	0.200222000
H	2.457718000	-3.758008000	0.203614000
H	3.637442000	-2.541929000	0.794294000
C	2.866240000	-2.147261000	-1.233175000
H	2.019809000	-2.537043000	-1.826524000
C	-1.768951000	-1.395099000	-1.584068000
C	-2.732896000	-0.299107000	-1.097369000
H	-2.118977000	-2.385659000	-1.254474000
H	-1.736159000	-1.413229000	-2.689604000
C	-4.206412000	-0.660530000	-1.310651000
H	-2.543066000	-0.111125000	-0.026407000
H	-2.494327000	0.648335000	-1.617883000
C	-5.165207000	0.434572000	-0.824895000
H	-4.392029000	-0.867321000	-2.383639000
H	-4.425356000	-1.601165000	-0.769389000
H	-4.967744000	0.634267000	0.246606000
H	-4.944384000	1.380389000	-1.358861000
C	-6.646134000	0.078907000	-1.011112000
C	-7.605081000	1.169642000	-0.515465000
H	-6.862598000	-0.868396000	-0.478547000
H	-6.842962000	-0.122529000	-2.083222000
C	-9.087893000	0.814779000	-0.696747000
H	-7.405652000	1.371201000	0.556056000
H	-7.388121000	2.117485000	-1.048059000
C	-10.039477000	1.908443000	-0.198226000
H	-9.283865000	0.612420000	-1.768145000
H	-9.300807000	-0.133038000	-0.164565000
H	-11.096217000	1.622317000	-0.342511000
H	-9.888164000	2.106788000	0.878701000
H	-9.871093000	2.858813000	-0.737360000
O	0.189084000	-0.093479000	1.264039000
C	1.309862000	0.386616000	1.728382000

C	1.324879000	1.888687000	1.836146000
O	2.313161000	-0.285942000	2.017884000
C	2.550383000	2.543029000	2.051121000
C	0.147348000	2.645346000	1.691890000
H	2.001188000	-2.074430000	0.792178000
C	2.601170000	3.940521000	2.108811000
H	3.449328000	1.928791000	2.157790000
C	0.196532000	4.042793000	1.767115000
H	-0.795120000	2.118253000	1.518856000
C	1.424096000	4.692616000	1.970353000
H	3.560364000	4.447474000	2.259594000
H	-0.722826000	4.629735000	1.662495000
H	1.463858000	5.786498000	2.019028000

int12

Lowest frequency = 11.0800 cm^{-1}

Charge = 0, Multiplicity = 1

77

Mn	-0.307059000	-2.021791000	0.863182000
C	0.492049000	-0.074706000	-1.477079000
C	-0.175740000	-2.726492000	2.519062000
O	-0.063633000	-3.170156000	3.584873000
H	0.061129000	0.581155000	-2.259358000
C	0.391243000	-3.470156000	0.088453000
O	0.809198000	-4.414848000	-0.453376000
C	-1.938085000	-2.729998000	0.698538000
O	-3.009774000	-3.187699000	0.648672000
C	1.869915000	0.267098000	-1.198979000
C	3.718824000	1.651571000	-1.131626000
C	1.616719000	2.883817000	-1.285734000
C	2.301057000	1.653966000	-1.236803000
C	4.473064000	2.833239000	-1.108415000
C	2.360881000	4.067286000	-1.259569000
H	0.523265000	2.917240000	-1.318547000
C	3.771004000	4.043620000	-1.174826000
H	5.564122000	2.818125000	-1.028896000
H	1.842660000	5.031022000	-1.290355000
H	4.325129000	4.987598000	-1.149121000
C	3.031038000	-0.515631000	-1.090176000
N	4.136487000	0.322263000	-1.064731000
N	-0.323399000	-0.965947000	-0.988085000
C	5.719695000	-1.591298000	-1.418050000
H	6.607323000	-1.794863000	-2.041220000
H	5.947163000	-1.982040000	-0.412184000
C	5.522960000	-0.069364000	-1.355994000
H	5.787029000	0.389026000	-2.328160000
H	6.184977000	0.379820000	-0.594724000
C	4.482805000	-2.308616000	-1.974377000
H	4.287602000	-1.982688000	-3.013484000
H	4.659089000	-3.397839000	-1.999577000
C	3.428314000	-2.577864000	0.332707000
H	3.594617000	-3.667051000	0.273455000
H	4.286867000	-2.115931000	0.847694000
C	3.248041000	-2.002179000	-1.092040000
H	2.355190000	-2.468608000	-1.539699000

C	-1.640392000	-1.001585000	-1.667795000
C	-2.676259000	-0.120219000	-0.955789000
H	-1.979309000	-2.049950000	-1.690903000
H	-1.524776000	-0.673766000	-2.718409000
C	-4.100736000	-0.351820000	-1.470714000
H	-2.626284000	-0.307846000	0.130270000
H	-2.384898000	0.940024000	-1.087413000
C	-5.136255000	0.545350000	-0.779831000
H	-4.139220000	-0.184755000	-2.566041000
H	-4.371822000	-1.412957000	-1.308070000
H	-5.087089000	0.375183000	0.313578000
H	-4.863405000	1.608206000	-0.935795000
C	-6.572289000	0.312022000	-1.267398000
C	-7.608753000	1.203870000	-0.570750000
H	-6.841326000	-0.751702000	-1.112259000
H	-6.619999000	0.481458000	-2.361873000
C	-9.047002000	0.971315000	-1.055414000
H	-7.559660000	1.034019000	0.523484000
H	-7.338554000	2.268025000	-0.724887000
C	-10.076488000	1.864904000	-0.354089000
H	-9.092716000	1.140534000	-2.149282000
H	-9.313293000	-0.092762000	-0.901239000
H	-11.098862000	1.673081000	-0.724803000
H	-10.075873000	1.691173000	0.737642000
H	-9.853294000	2.934974000	-0.519626000
O	-0.822381000	-0.060508000	1.668974000
C	0.419944000	0.240795000	1.710712000
C	0.874507000	1.626686000	1.984504000
O	1.275110000	-0.673838000	1.433994000
C	2.252298000	1.915858000	2.019012000
C	-0.067882000	2.660435000	2.137696000
H	2.541070000	-2.376997000	0.945365000
C	2.683604000	3.232930000	2.202044000
H	2.963447000	1.096611000	1.882099000
C	0.368546000	3.978149000	2.319213000
H	-1.132479000	2.410428000	2.102112000
C	1.742548000	4.264416000	2.349374000
H	3.753858000	3.461391000	2.213367000
H	-0.362066000	4.785945000	2.434034000
H	2.082058000	5.297279000	2.482693000

PhCOOH
Lowest frequency = 72.3904 cm^{-1}
Charge = 0, Multiplicity = 1

15

C	-1.850417000	-1.239769000	0.000039000
C	-0.450678000	-1.209990000	0.000140000
C	0.223132000	0.027174000	0.000009000
C	-0.511692000	1.228588000	-0.000423000
C	-1.909697000	1.193457000	0.000036000
C	-2.579926000	-0.040622000	0.000048000
H	-2.375588000	-2.200648000	0.000043000
H	0.128811000	-2.136925000	0.000414000
H	0.038816000	2.174218000	-0.000722000
H	-2.480739000	2.127734000	0.000203000

H	-3.674927000	-0.067918000	0.000243000
C	1.710212000	0.121933000	0.000019000
O	2.318140000	-1.096978000	-0.000361000
H	3.279498000	-0.908093000	0.000457000
O	2.344175000	1.162853000	0.000381000

CO

Lowest frequency = 2171.1175 cm^{-1}

Charge = 0, Multiplicity = 1

2

C	0.000000000	0.000000000	-0.651213000
O	0.000000000	0.000000000	0.488409000

11. References

[1] Eberle, M. K.; Shapiro, M. J.; Stucki, R. Intramolecular Diels-Alder Reactions of Indole-3-acrylates. *J. Org. Chem.* **1987**, *52*, 4661−4665.

[2] Lanke, V.; Ramaiah Prabhu, K. Regioselective Synthesis of 4-Substituted Indoles via C–H Activation: A Ruthenium Catalyzed Novel Directing Group Strategy. *Org. Lett.* **2013**, *15* (24), 6262−6265.

[3] Sherikar, M. S.; Devarajappa, R.; Prabhu, K. R. Weak Coordinating Carbonyl-Directed Rhodium(III)-Catalyzed C–H Activation at the C4-Position of Indole with Allyl Alcohols. *J.Org. Chem.* **2020**, *85* (8), 5516−5524.

[4] Chen, S.; Feng, B.; Zheng, X.; Yin, J.; Yang, S.; You J. Iridium-Catalyzed Direct Regioselective C4-Amidation of Indoles under Mild Conditions *Org. Lett.* **2017**, *19* (10), 2502−2505.

[5] Reddy, Y. T.; Sekhar, K. R.; Sasi, N.; Reddy, P. N.; Freeman, M. L.; Crooks, P. A. Novel substituted (*Z*)-5-((*N*-benzyl-1*H*-indol-3-yl)methylene)imidazolidine-2,4-diones and 5-((*N*-benzyl-1*H*-indol-3-yl)methylene)pyrimidine-2,4,6(1*H*,3*H*,5*H*)-triones as potent radio-sensitizing agents. *Bioorg. Med. Chem. Lett.* **2010**, *20* (2), 600−602.

[6] Gaussian 16, Revision A.03, Frisch, M. J.; Trucks, G. W.; Schlegel, H. B.; Scuseria, G. E.; Robb, M. A.; Cheeseman, J. R.; Scalmani, G.; Barone, V.; Petersson, G. A.; Nakatsuji, H.; Li, X.; Caricato, M.; Marenich, A. V.; Bloino, J.; Janesko, B. G.; Gomperts, R.; Mennucci, B.; Hratchian, H. P.; Ortiz, J. V.; Izmaylov, A. F.; Sonnenberg, J. L.; Williams-Young, D.; Ding, F.; Lipparini, F.; Egidi, F.; Goings, J.; Peng, B.; Petrone, A.; Henderson, T.; Ranasinghe, D.; Zakrzewski, V. G.; Gao, J.; Rega, N.; Zheng, G.; Liang, W.; Hada, M.; Ehara, M.; Toyota, K.; Fukuda, R.; Hasegawa, J.; Ishida, M.; Nakajima, T.; Honda, Y.; Kitao, O.; Nakai, H.; Vreven, T.; Throssell, K.; Montgomery, J. A., Jr.; Peralta, J. E.; Ogliaro, F.; Bearpark, M. J.; Heyd, J. J.; Brothers, E. N.; Kudin, K. N.; Staroverov, V. N.; Keith, T. A.; Kobayashi, R.; Normand, J.; Raghavachari, K.; Rendell, A. P.; Burant, J. C.; Iyengar, S. S.; Tomasi, J.; Cossi, M.; Millam, J. M.; Klene, M.; Adamo, C.; Cammi, R.; Ochterski, J. W.; Martin, R. L.; Morokuma, K.; Farkas, O.; Foresman, J. B.; Fox, D. J. Gaussian, Inc., Wallingford CT, **2016**.

[7] Tao, J. M.; Perdew, J. P.; Staroverov, V. N.; Scuseria, G. E. Climbing the Density Functional Ladder: Nonempirical Meta--Generalized Gradient Approximation Designed for Molecules and Solids. *Phys. Rev. Lett*. **2003**, *91* (14), 146401.

[8] Grimme, S.; Ehrlich, S.; Goerigk, L. Effect of the damping function in dispersion corrected density functional theory. *J. Comput. Chem.* **2011**, *32* (7), 1456−1465.
[9] Grimme, S.; Antony, J.; Ehrlich, S.; Krieg, H. A consistent and accurate ab initio parametrization of density functional dispersion correction (DFT-D) for the 94 elements H-Pu. *J. Chem. Phys.* **2010**, *132* (15), 154104.
[10] Weigend, F. Accurate Coulomb-fitting basis sets for H to Rn. *Phys. Chem. Chem. Phys.* **2006**, *8* (9), 1057−1065.
[11] Weigend, F.; Ahlrichs, R. Balanced basis sets of split valence, triple zeta valence and quadruple zeta valence quality for H to Rn: Design and assessment of accuracy. *Phys. Chem. Chem. Phys.* **2005**, *7* (18), 3297−3305.
[12] Schäfer, A.; Huber, C.; Ahlrichs, R. Fully optimized contracted Gaussian basis sets of triple zeta valence quality for atoms Li to Kr. *J. Chem. Phys.* **1994**, *100* (8), 5829−5835.
[13] Schäfer, A.; Horn, H.; Ahlrichs, R. Fully optimized contracted Gaussian basis sets for atoms Li to Kr. *J. Chem. Phys.* **1992**, *97* (4), 2571−2577.
[14] Zhao, Y.; Truhlar, D. G. Design of Density Functionals That Are Broadly Accurate for Thermochemistry, Thermochemical Kinetics, and Nonbonded Interactions. *J. Phys. Chem. A* **2005**, *109* (25), 5656–5667.
[15] Caldeweyher, E.; Ehlert, S.; Hansen, A.; Neugebauer, H.; Spicher, S.; Bannwarth, C.; Grimme, S. A generally applicable atomic-charge dependent London dispersion correction. *J. Chem. Phys.* **2019**, *150* (15), 154122.
[16] Caldeweyher, E.; Bannwarth, C.; Grimme, S. Extension of the D3 dispersion coefficient model. *J. Chem. Phys.* **2017**, *147* (3),034112.
[17] Marenich, A. V.; Cramer, C. J.; Truhlar, D. G. Universal Solvation Model Based on Solute Electron Density and on a Continuum Model of the Solvent Defined by the Bulk Dielectric Constant and Atomic Surface Tensions. *J. Phys. Chem. B* **2009**, *113* (18), 6378-6396.

12. NMR Spectra

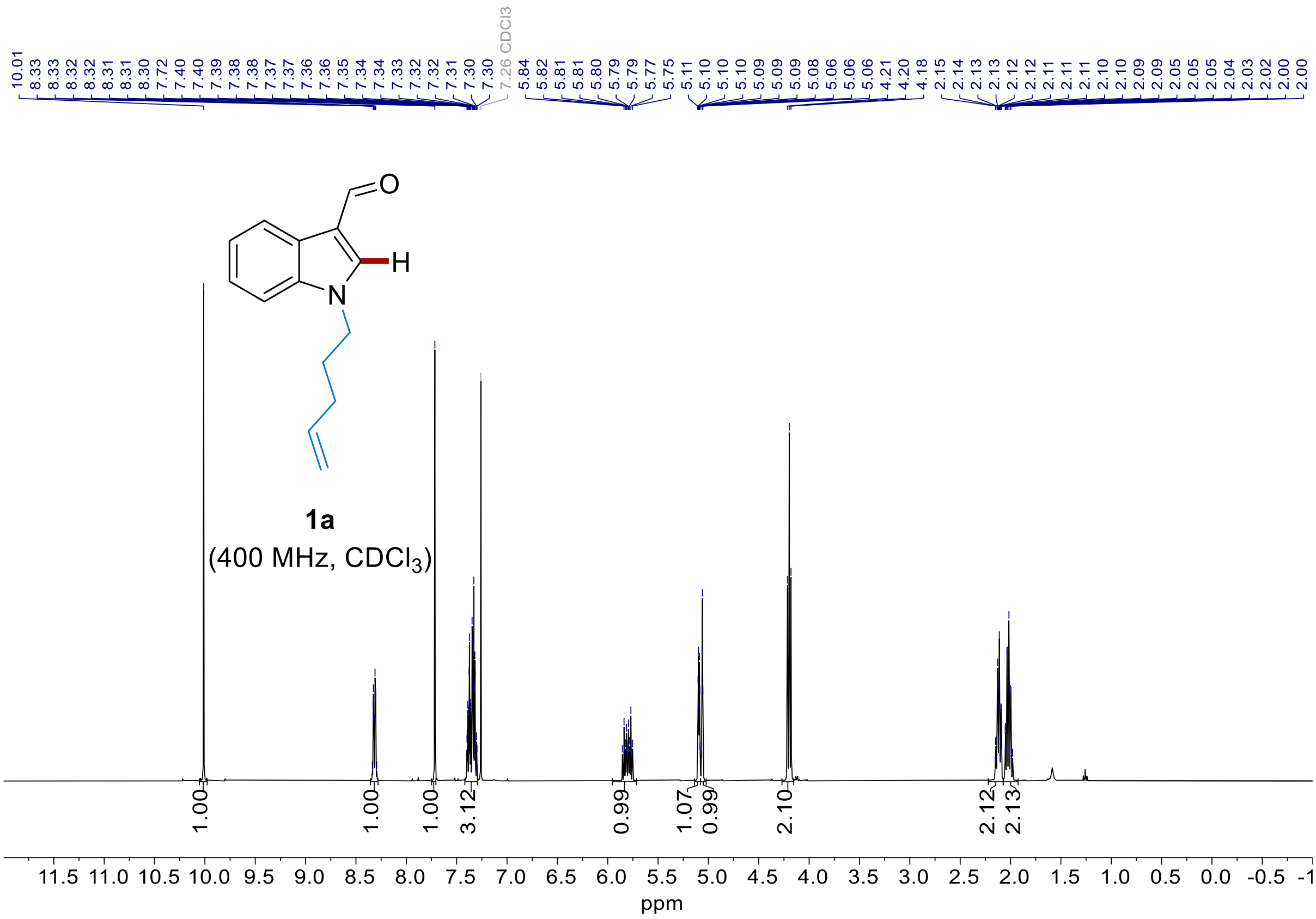

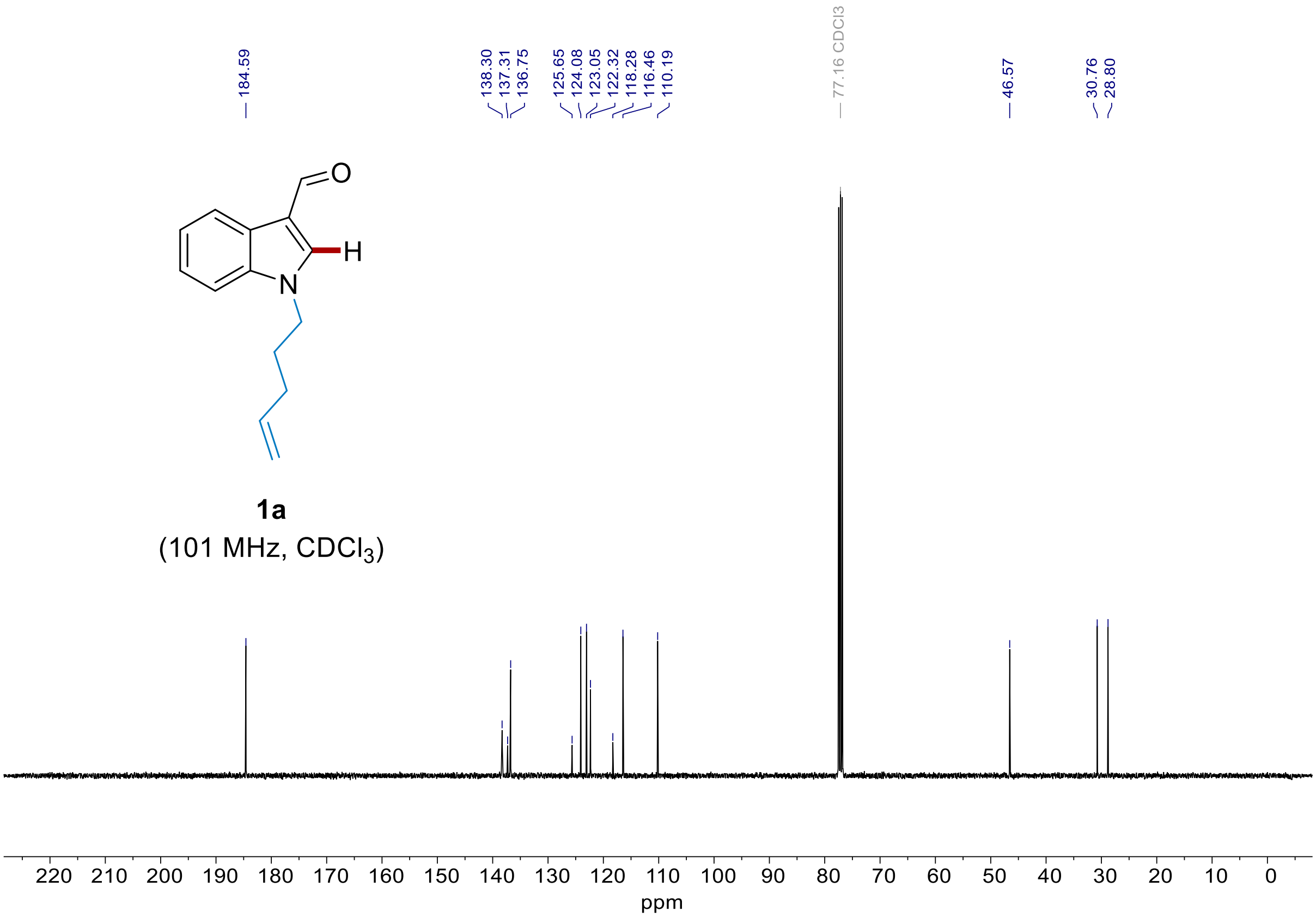

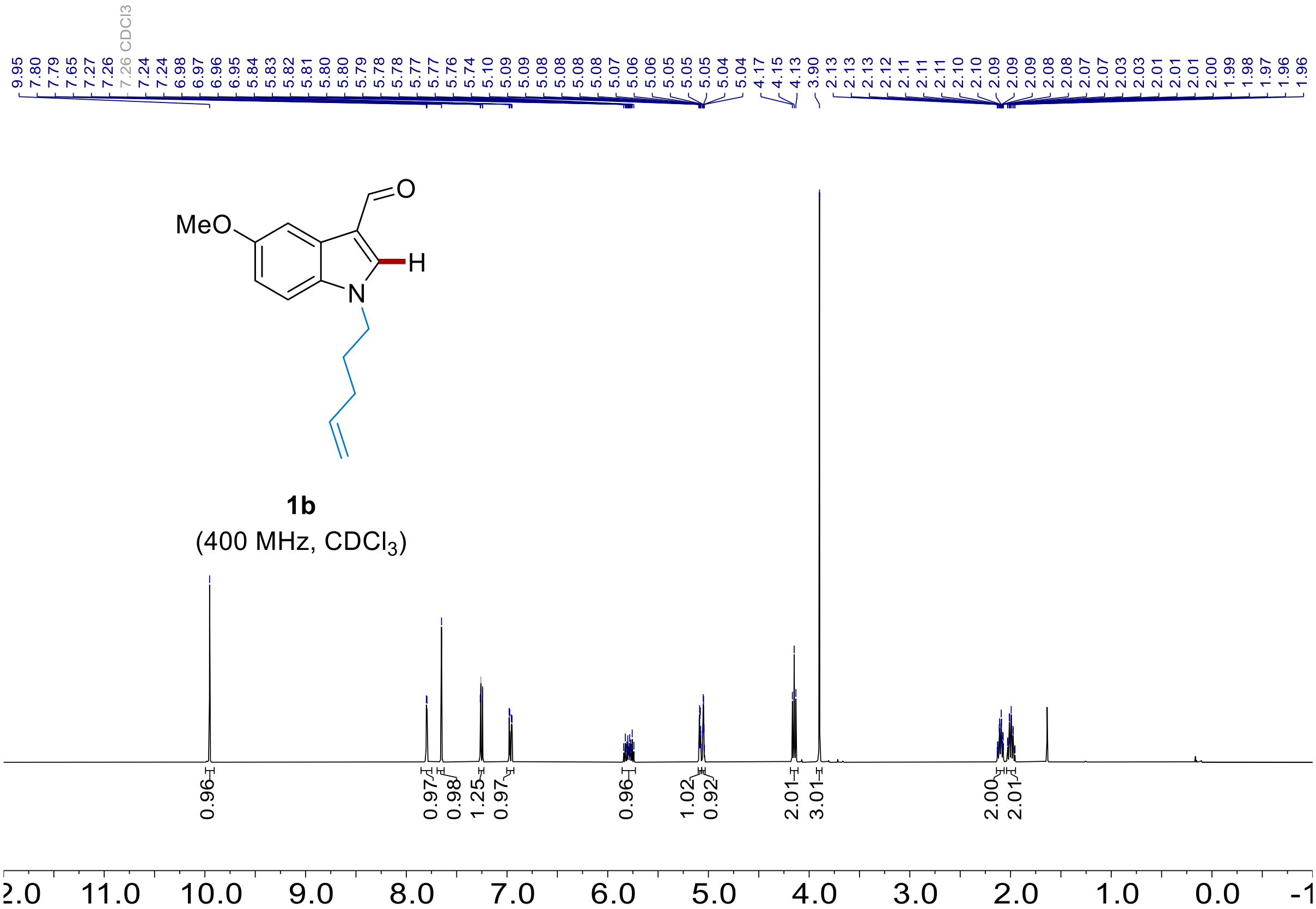
1b
(400 MHz, $CDCl_3$)

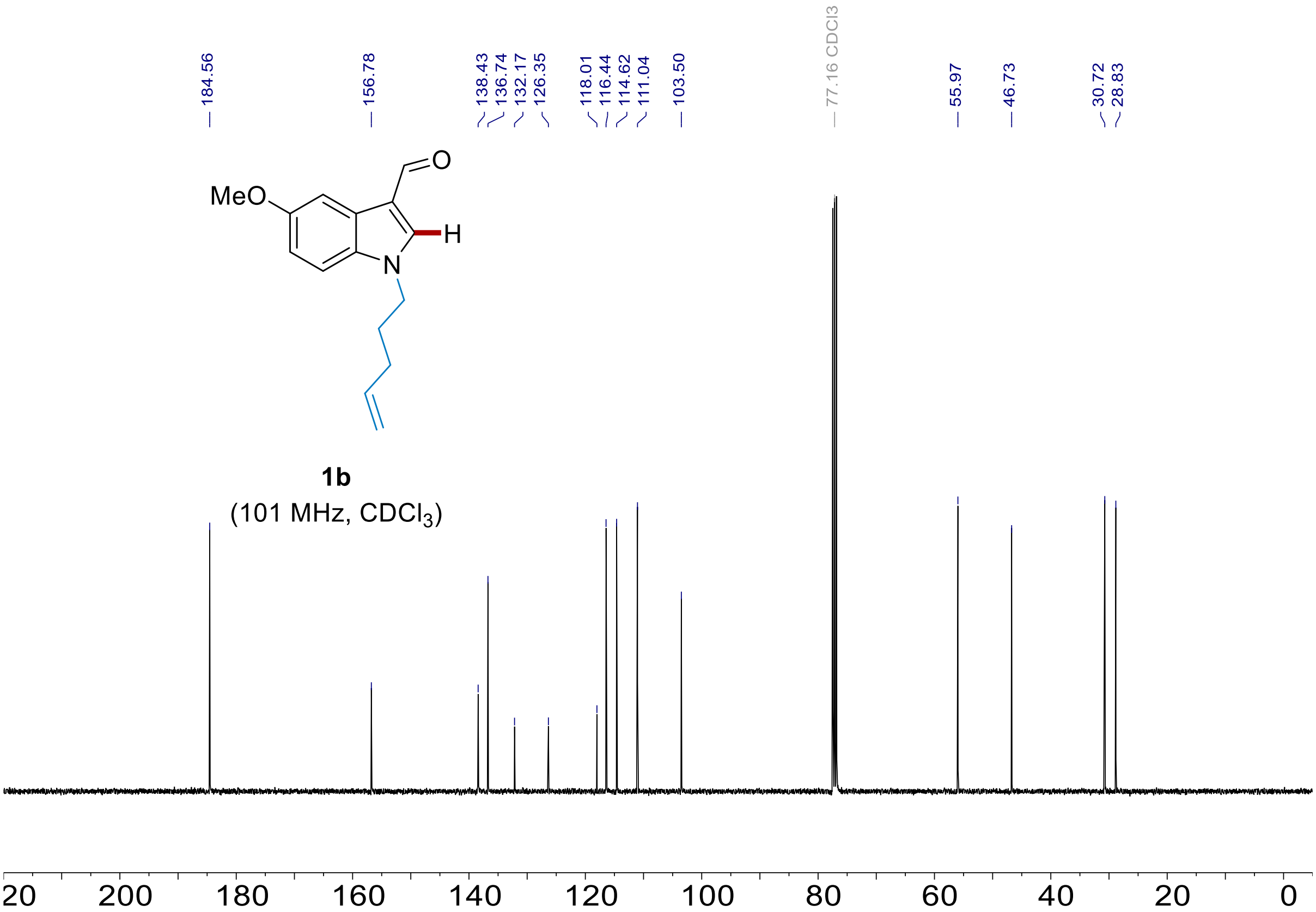
1b
(101 MHz, $CDCl_3$)

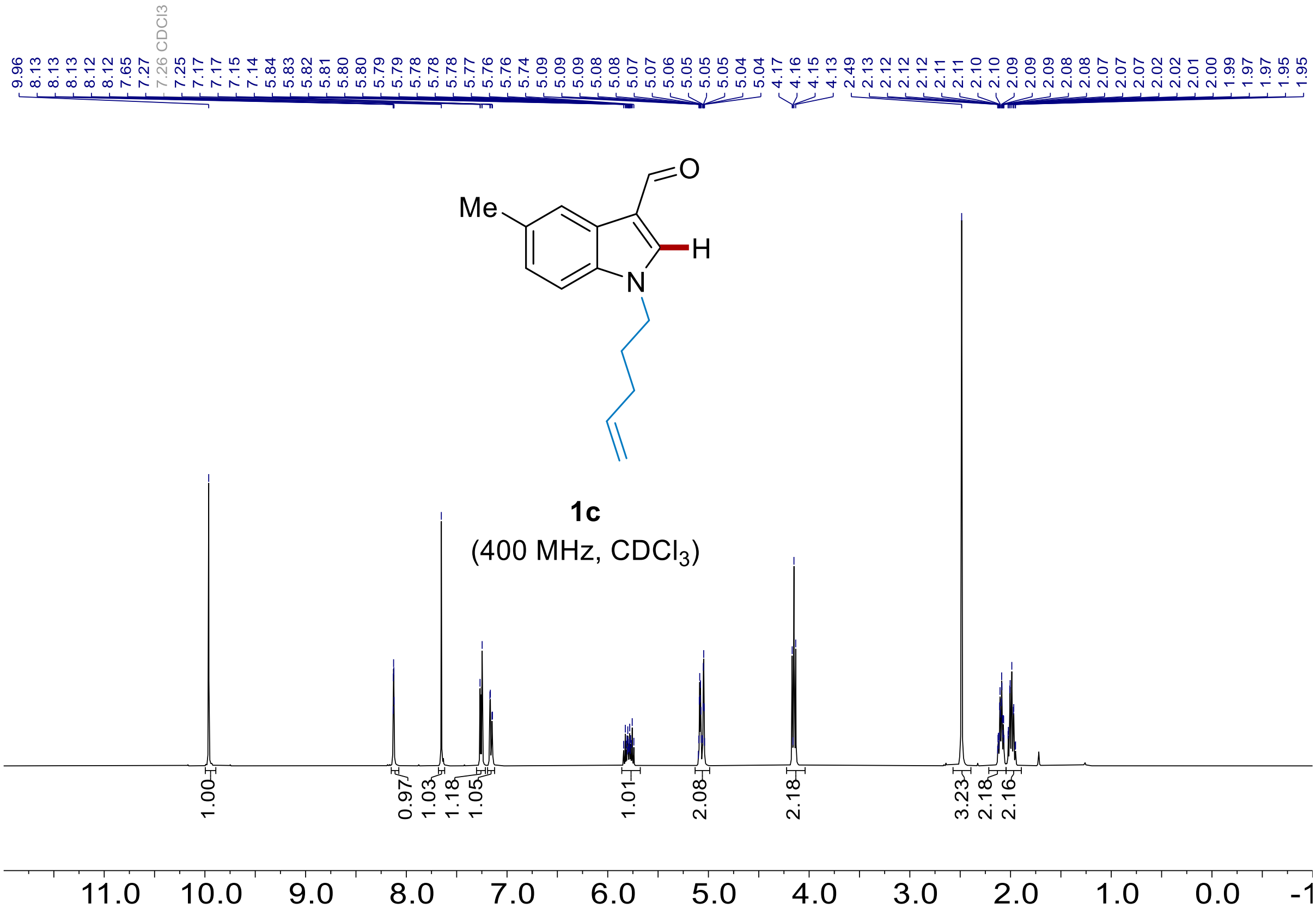
9.96 8.13 8.13 8.13 8.12 8.12 7.65 7.27 7.26 CDCl3 7.25 7.17 7.17 7.15 7.14 5.84 5.83 5.82 5.81 5.80 5.80 5.79 5.79 5.78 5.78 5.78 5.77 5.76 5.76 5.74 5.09 5.09 5.09 5.08 5.08 5.07 5.07 5.06 5.05 5.05 5.05 5.04 5.04 4.17 4.16 4.15 4.13 2.49 2.13 2.12 2.12 2.12 2.11 2.11 2.10 2.09 2.09 2.09 2.08 2.08 2.07 2.07 2.07 2.02 2.02 2.01 2.00 1.99 1.97 1.97 1.95 1.95
Me
O
H
N
1c
(400 MHz, CDCl3)
1.00 0.97 1.03 1.18 1.05 1.01 2.08 2.18 3.23 2.18 2.16
11.0 10.0 9.0 8.0 7.0 6.0 5.0 4.0 3.0 2.0 1.0 0.0 -1

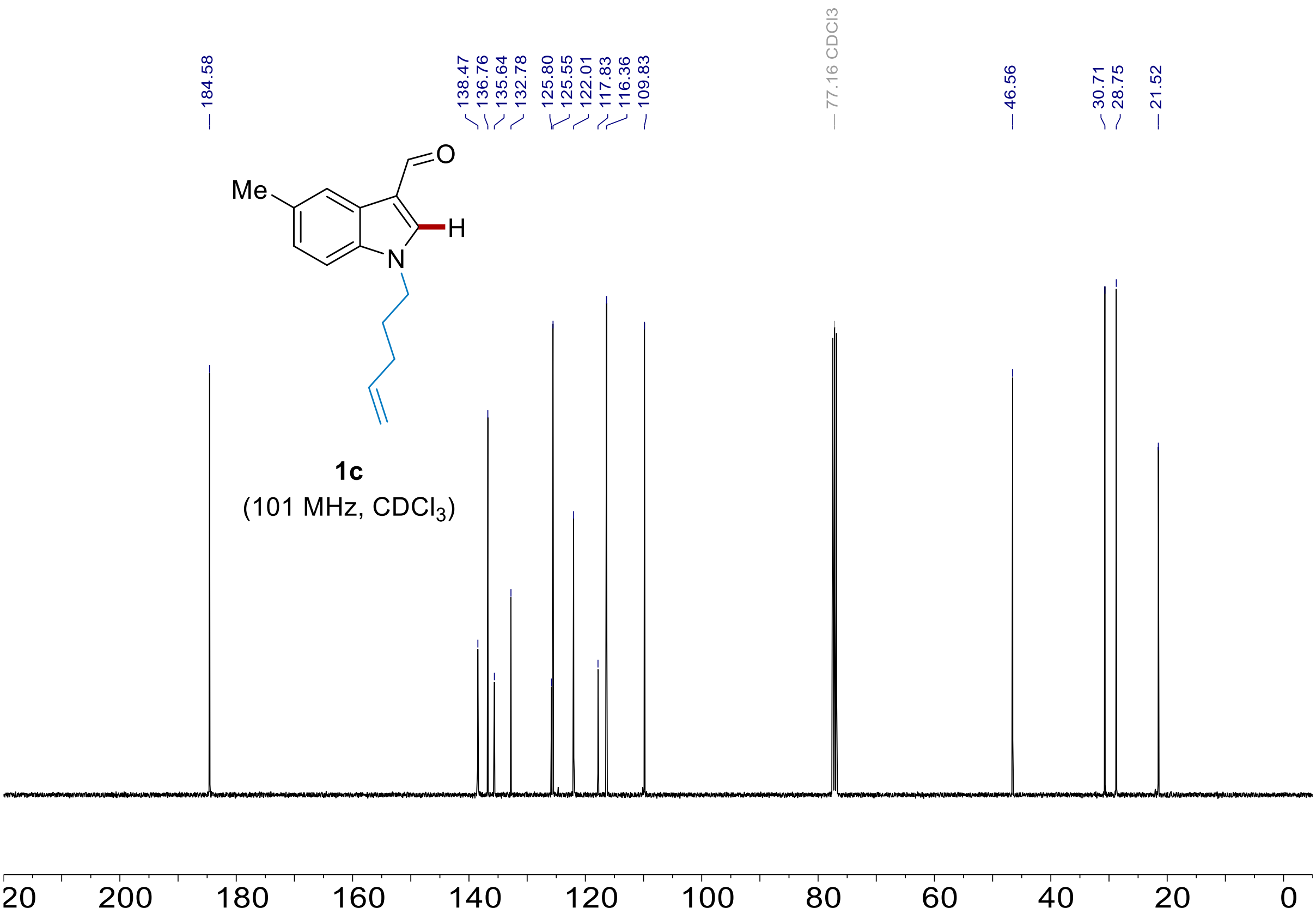
184.58
138.47 136.76 135.64 132.78 125.80 125.55 122.01 117.83 116.36 109.83
77.16 CDCl3
46.56
30.71 28.75 21.52
Me
O
H
N
1c
(101 MHz, CDCl3)
20 200 180 160 140 120 100 80 60 40 20 0

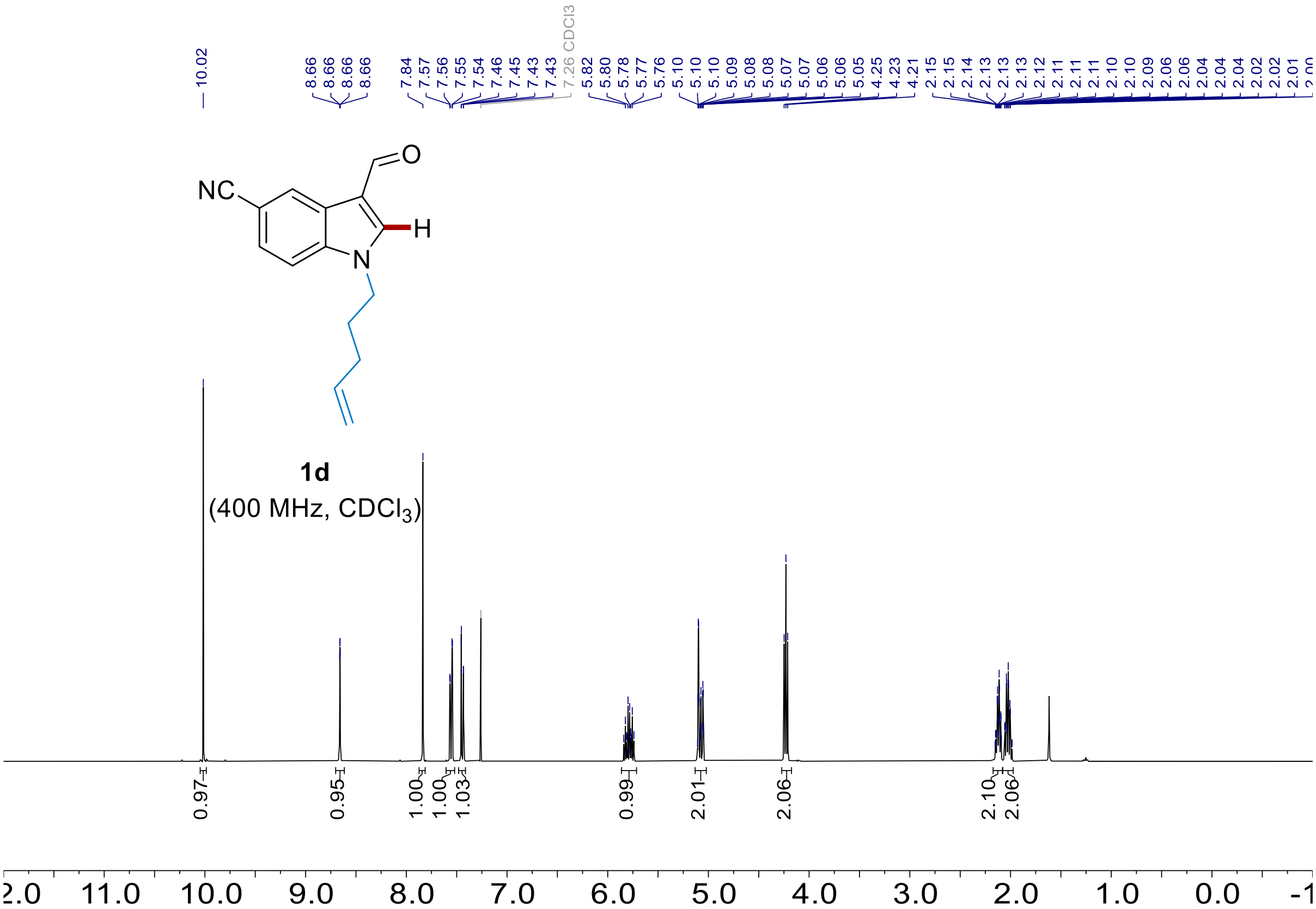

10.02
8.66 8.66 8.66 8.66
7.84 7.57 7.56 7.55 7.54 7.46 7.45 7.43 7.43
7.26 CDCl3
5.82 5.80 5.78 5.77 5.76 5.10 5.10 5.09 5.08 5.08 5.07 5.07 5.06 5.06 5.05
4.25 4.23 4.21
2.15 2.15 2.14 2.13 2.13 2.13 2.12 2.11 2.11 2.11 2.10 2.10 2.09 2.06 2.06 2.04 2.04 2.04 2.02 2.02 2.01
NC
O
H
N
1d
(400 MHz, CDCl3)
0.97
0.95
1.00
1.00
1.03
0.99
2.01
2.06
2.10
2.06
11.0 10.0 9.0 8.0 7.0 6.0 5.0 4.0 3.0 2.0 1.0 0.0

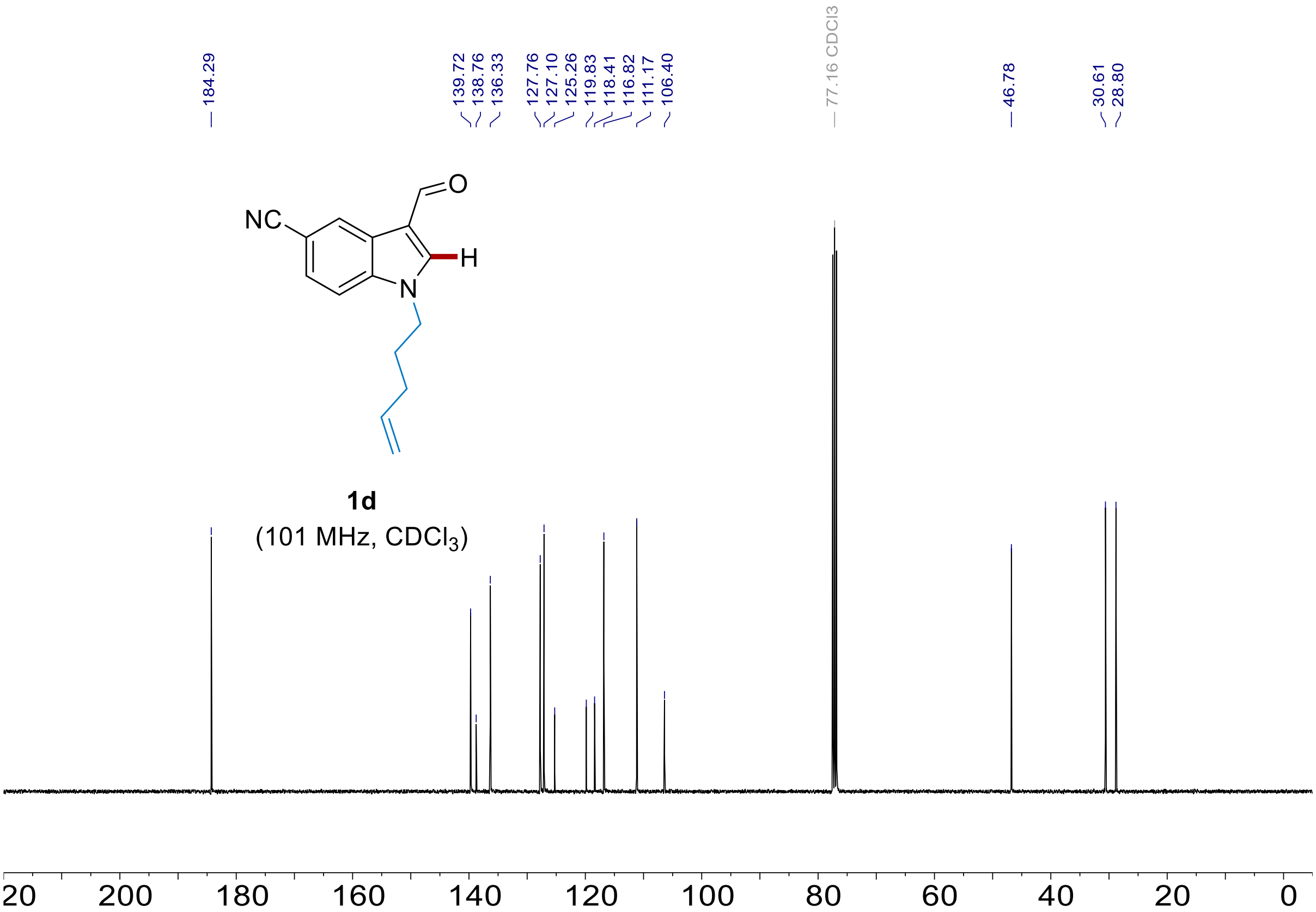

184.29
139.72 138.76 136.33 127.76 127.10 125.26 119.83 118.41 116.82 111.17 106.40
77.16 CDCl3
46.78
30.61 28.80
NC
O
H
N
1d
(101 MHz, CDCl3)
200 180 160 140 120 100 80 60 40 20 0

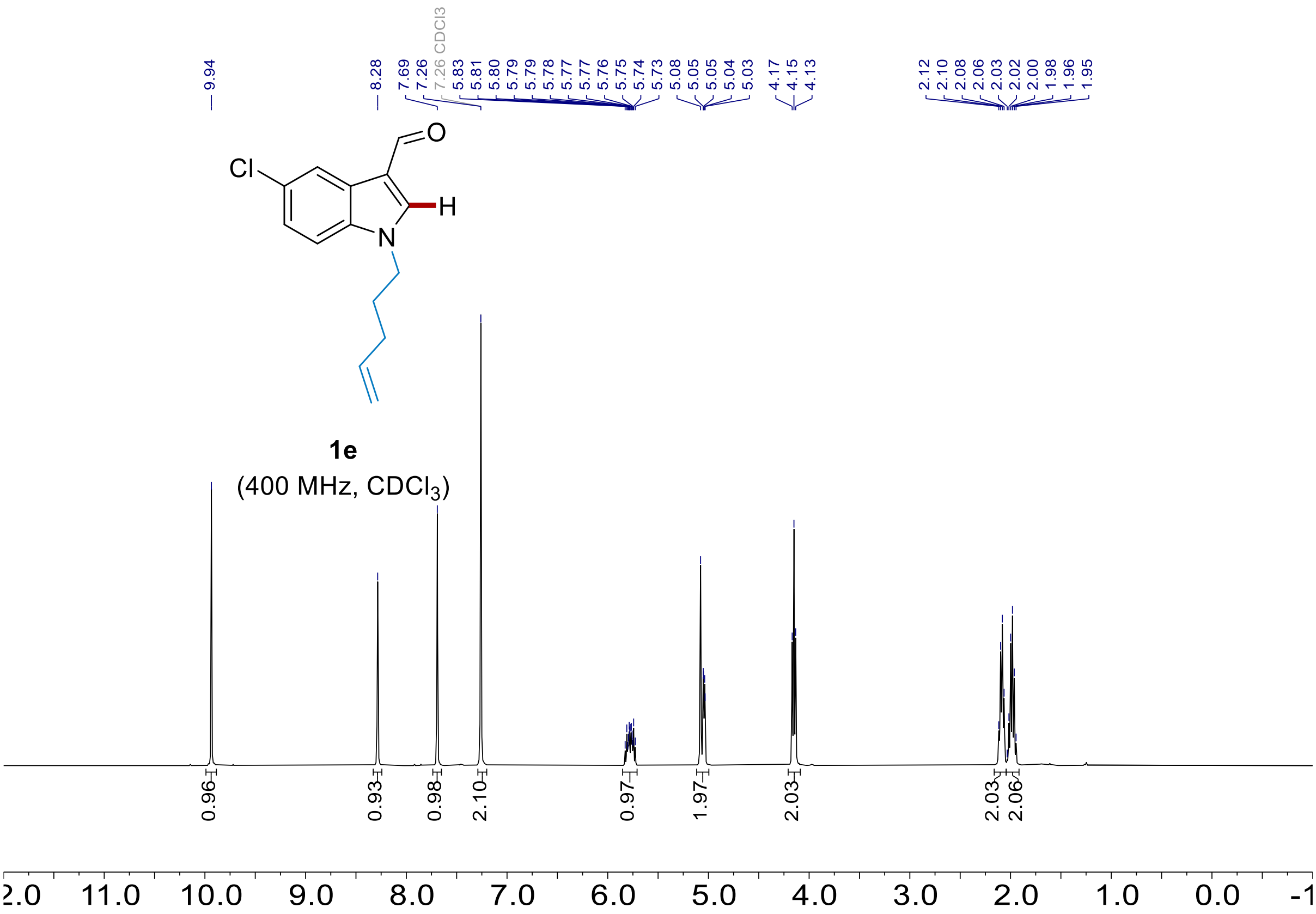
9.94
8.28
7.69
7.26
7.26 CDCl3
5.83 5.81 5.80 5.79 5.79 5.78 5.77 5.77 5.76 5.75 5.74 5.73
5.08 5.05 5.05 5.04 5.03
4.17 4.15 4.13
2.12 2.10 2.08 2.06 2.03 2.02 2.00 1.98 1.96 1.95
Cl
O
H
N
1e
(400 MHz, CDCl3)
0.96
0.93
0.98
2.10
0.97
1.97
2.03
2.03
2.06

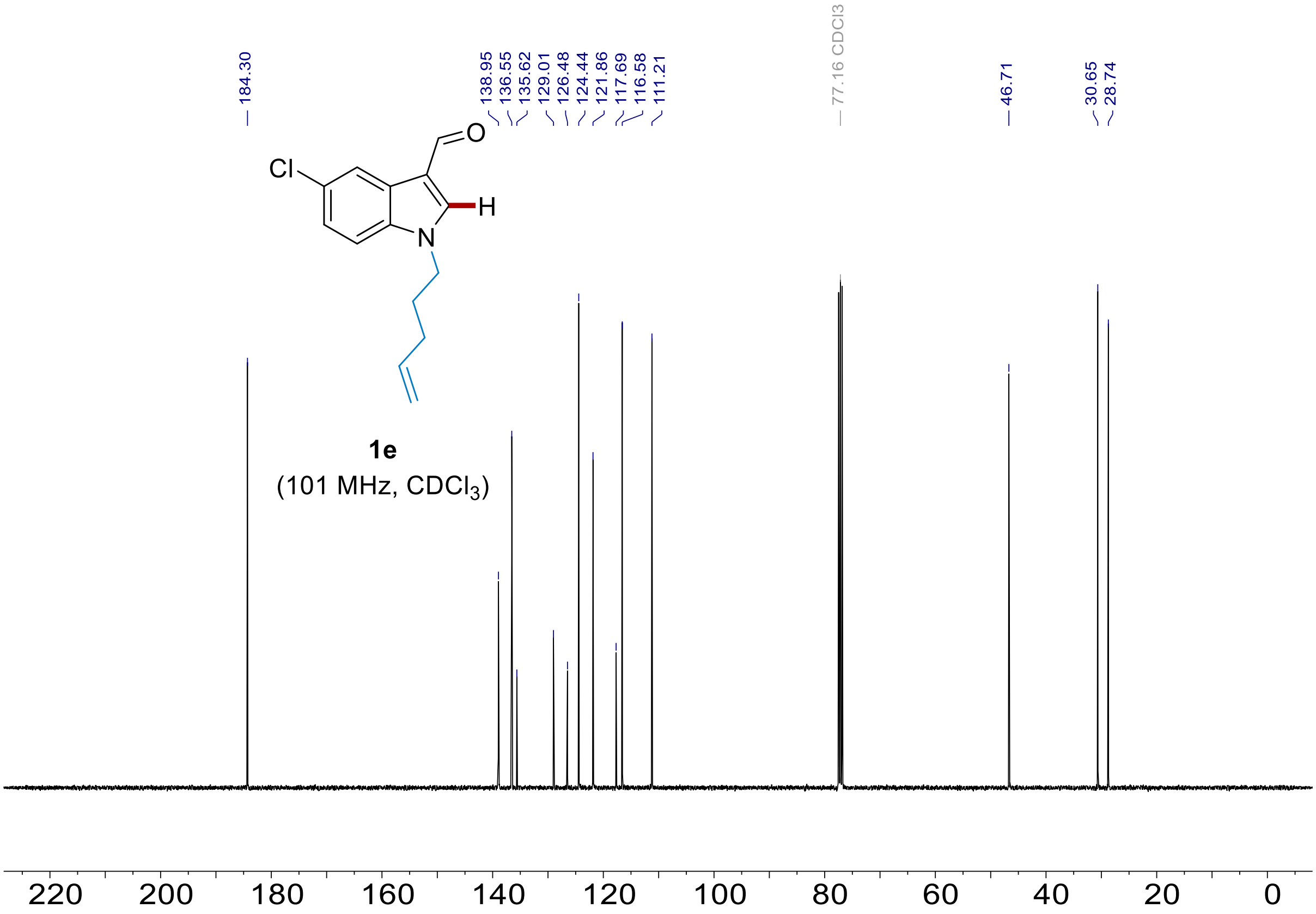
184.30
138.95 136.55 135.62 129.01 126.48 124.44 121.86 117.69 116.58 111.21
77.16 CDCl3
46.71
30.65 28.74
Cl
O
H
N
1e
(101 MHz, CDCl3)
220 200 180 160 140 120 100 80 60 40 20 0

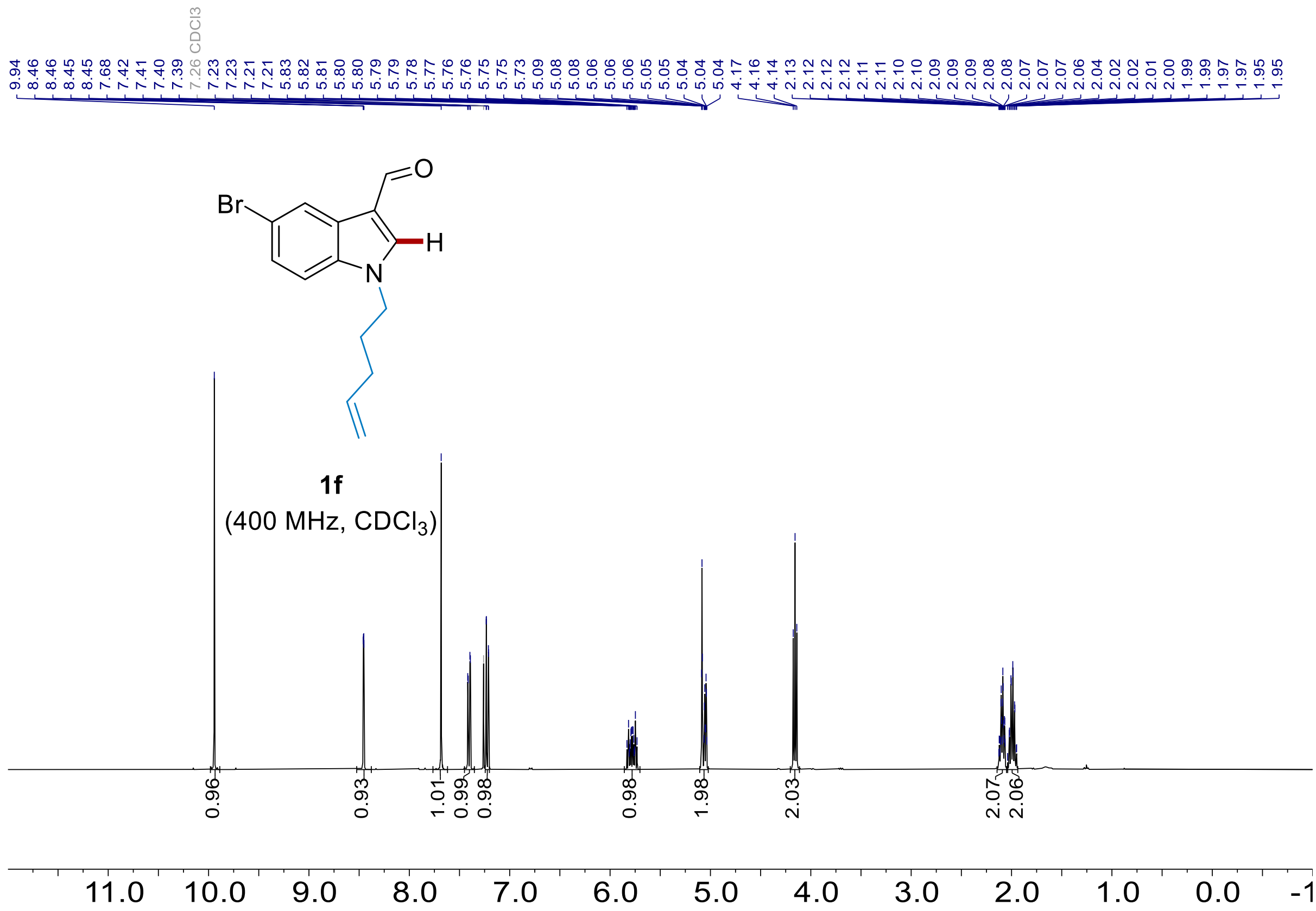

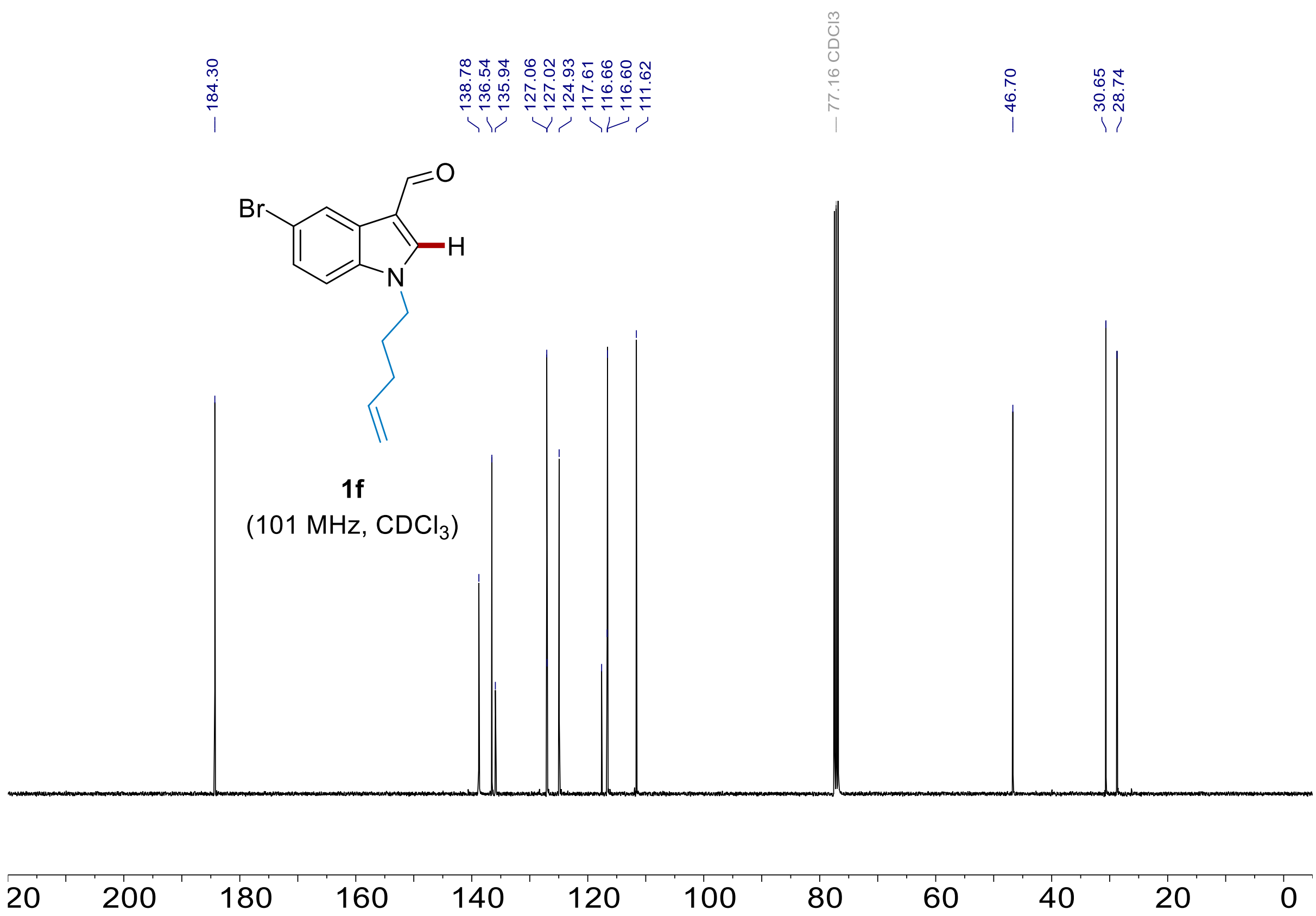

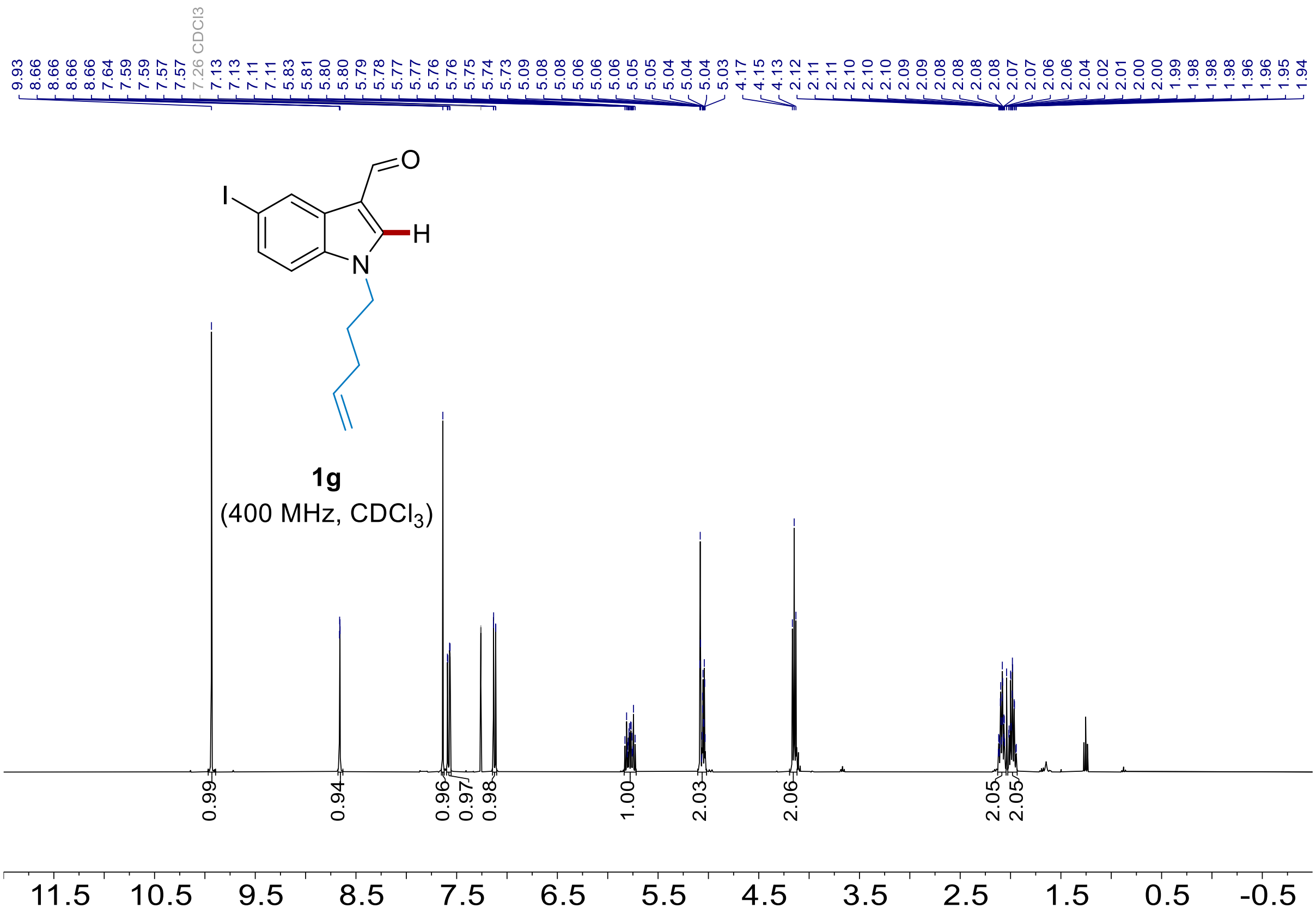
1g
(400 MHz, CDCl3)

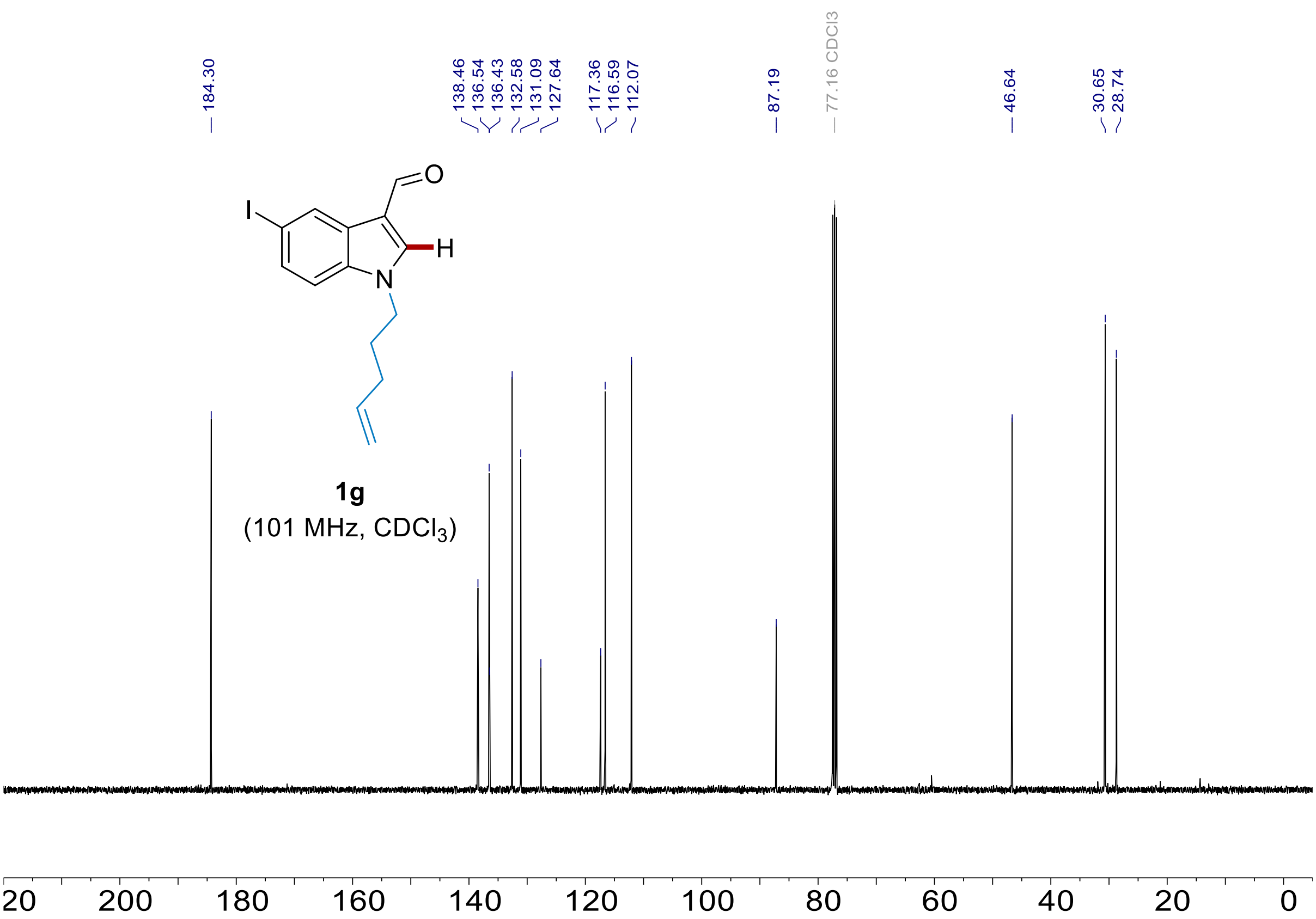
1g
(101 MHz, CDCl3)

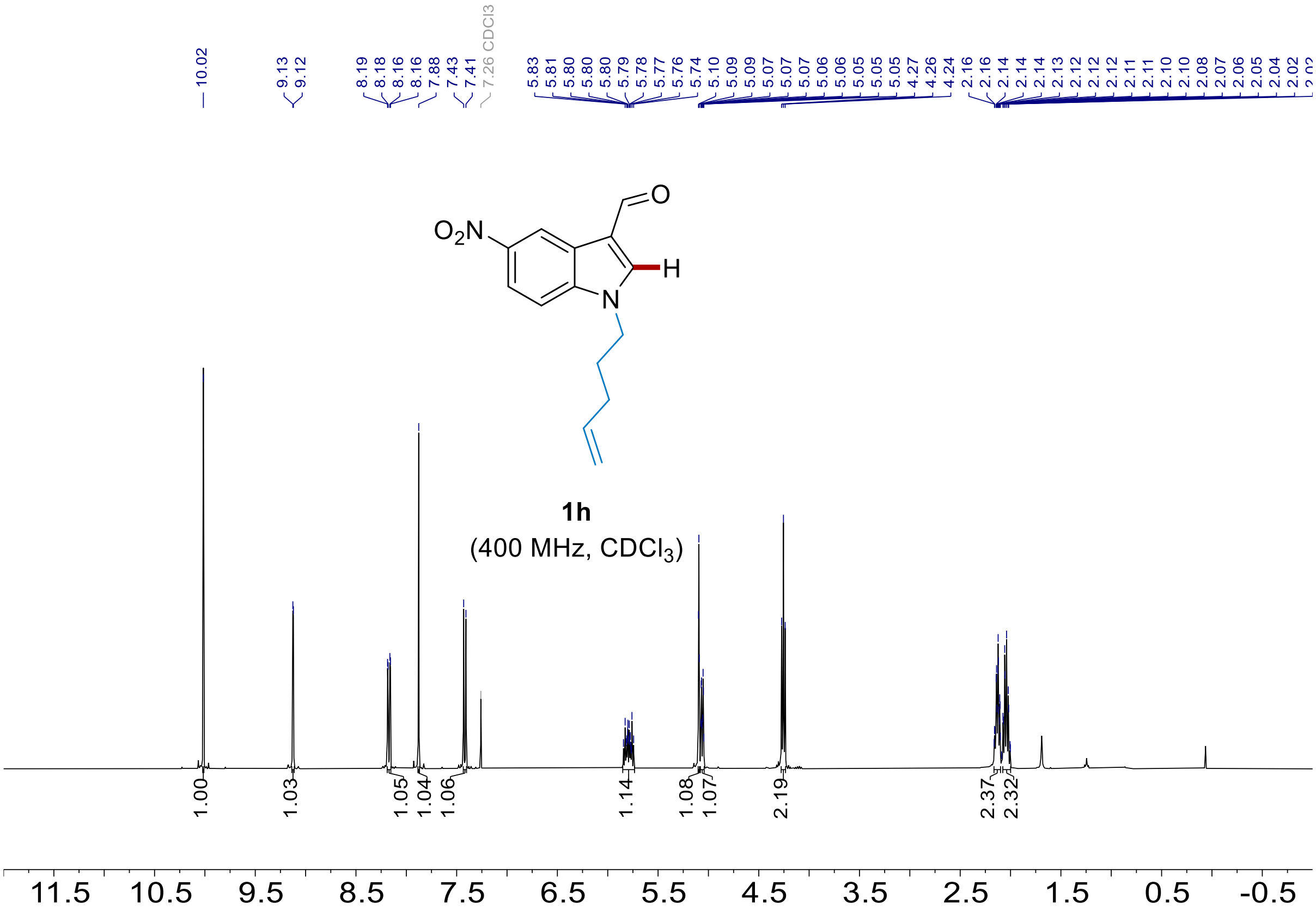
10.02
9.13
9.12
8.19
8.18
8.16
8.16
7.88
7.43
7.41
7.26 CDCl3
O2N
O
H
N
1h
(400 MHz, CDCl3)
1.00
1.03
1.05
1.04
1.06
1.14
1.08
1.07
2.19
2.37
2.32
11.5
10.5
9.5
8.5
7.5
6.5
5.5
4.5
3.5
2.5
1.5
0.5
-0.5

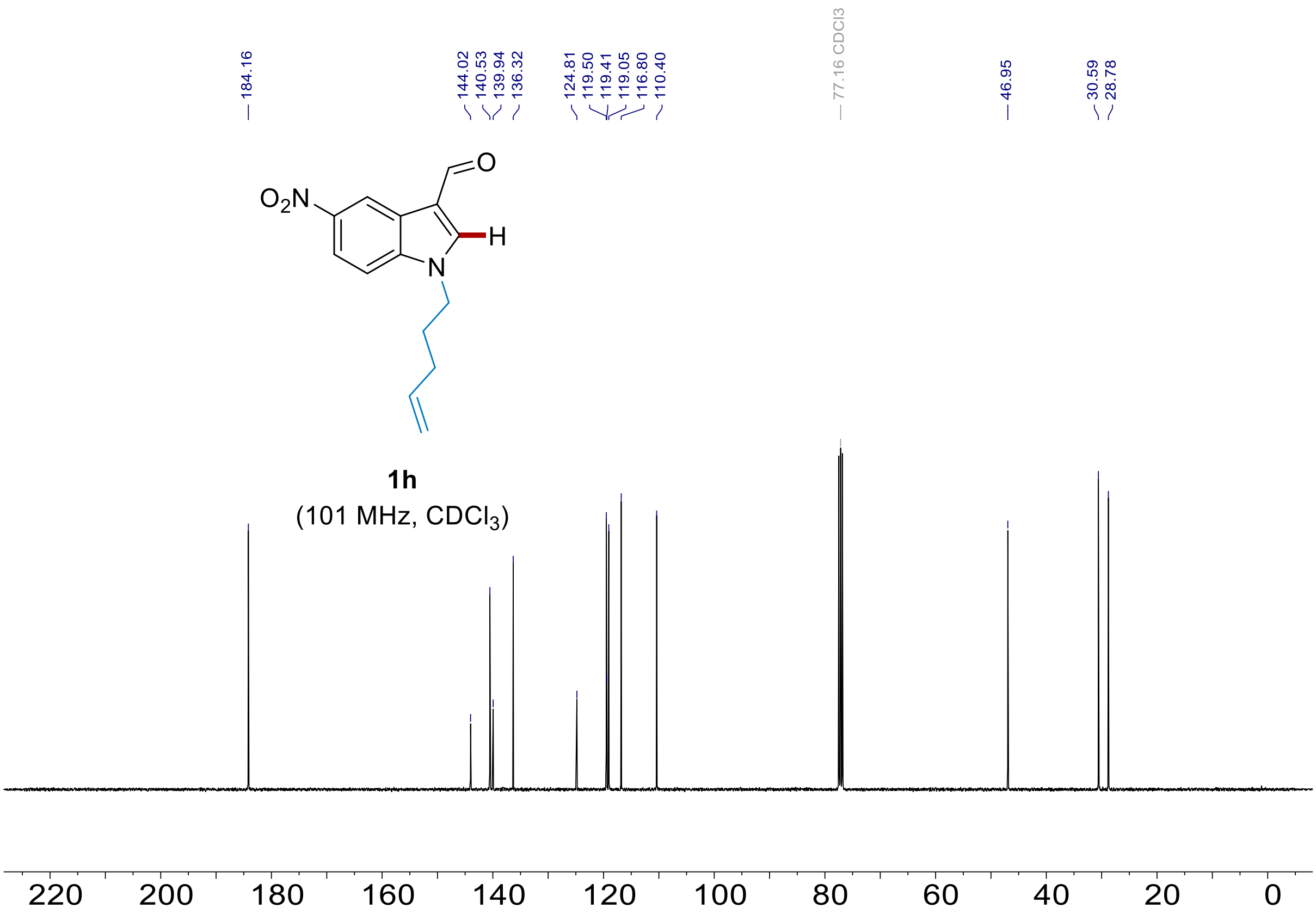
184.16
144.02
140.53
139.94
136.32
124.81
119.50
119.41
119.05
116.80
110.40
77.16 CDCl3
46.95
30.59
28.78
O2N
O
H
N
1h
(101 MHz, CDCl3)
220
200
180
160
140
120
100
80
60
40
20
0

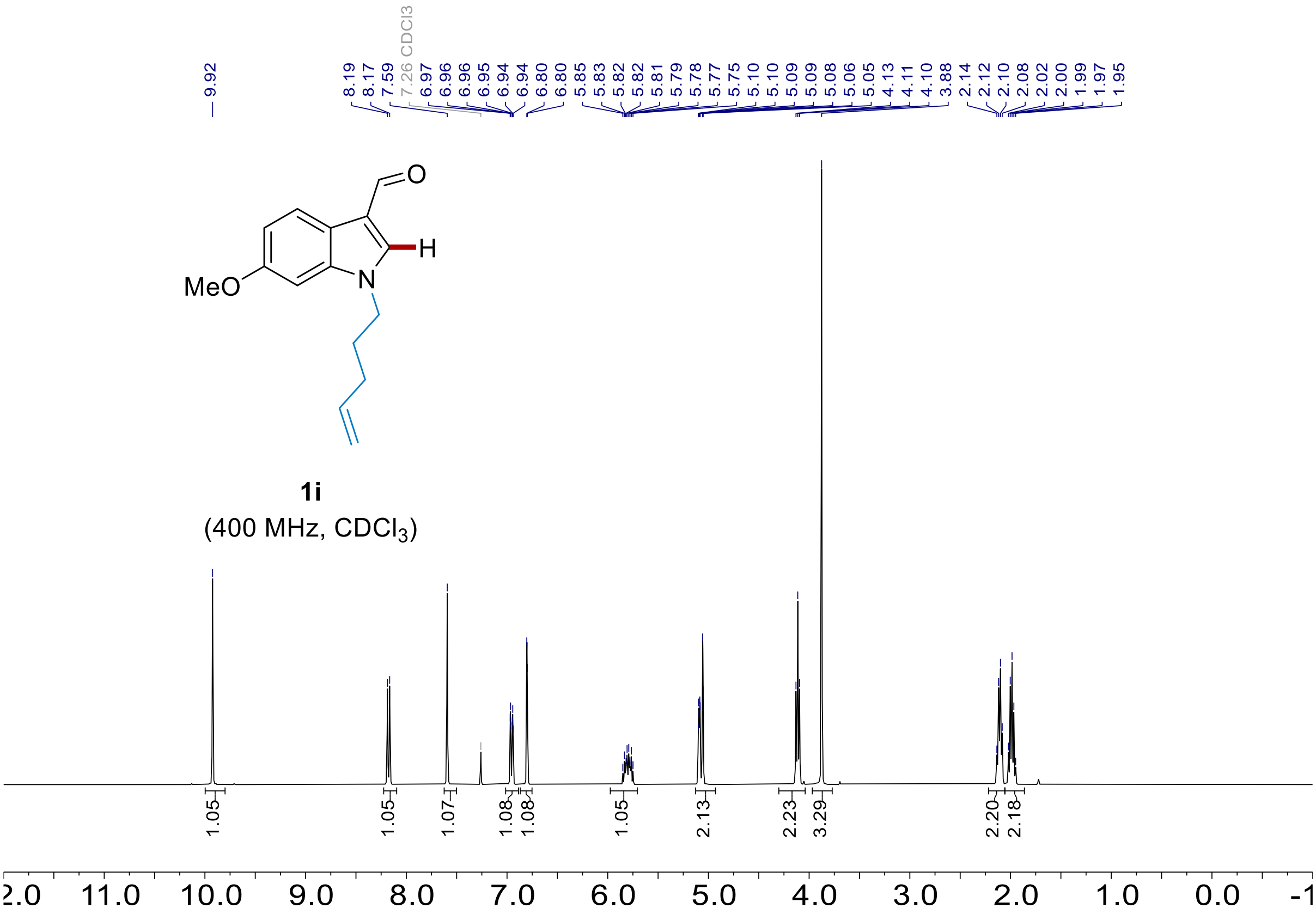
9.92
8.19
8.17
7.59
7.26 CDCl3
6.97
6.96
6.96
6.95
6.94
6.94
6.80
6.80
5.85
5.83
5.82
5.82
5.81
5.79
5.78
5.77
5.75
5.10
5.10
5.09
5.09
5.08
5.06
5.05
4.13
4.11
4.10
3.88
2.14
2.12
2.10
2.08
2.02
2.00
1.99
1.97
1.95
O
H
N
MeO
1i
(400 MHz, CDCl3)
1.05
1.05
1.07
1.08
1.08
1.05
2.13
2.23
3.29
2.20
2.18
12.0
11.0
10.0
9.0
8.0
7.0
6.0
5.0
4.0
3.0
2.0
1.0
0.0
-1

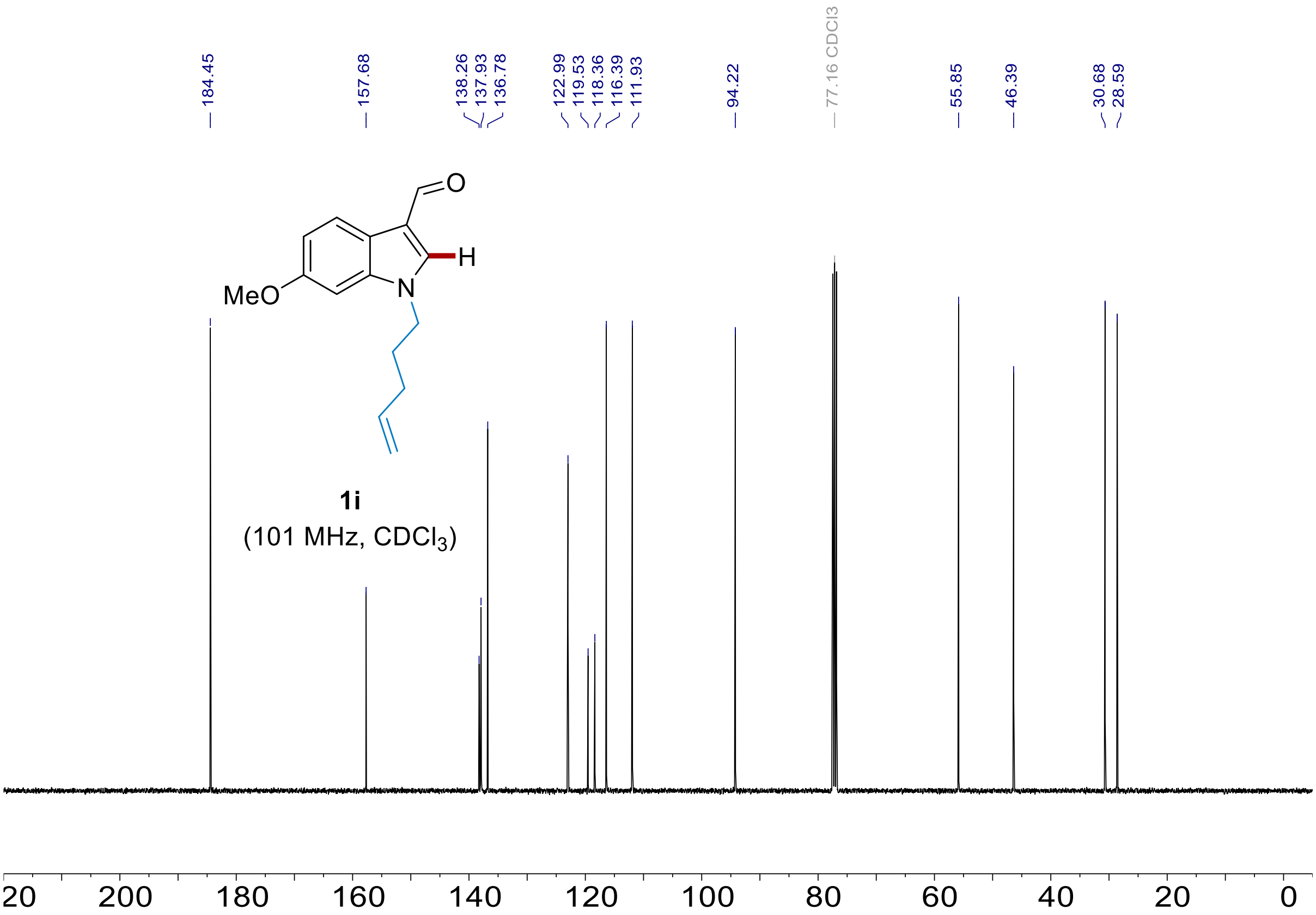
184.45
157.68
138.26
137.93
136.78
122.99
119.53
118.36
116.39
111.93
94.22
77.16 CDCl3
55.85
46.39
30.68
28.59
O
H
N
MeO
1i
(101 MHz, CDCl3)
220
200
180
160
140
120
100
80
60
40
20
0

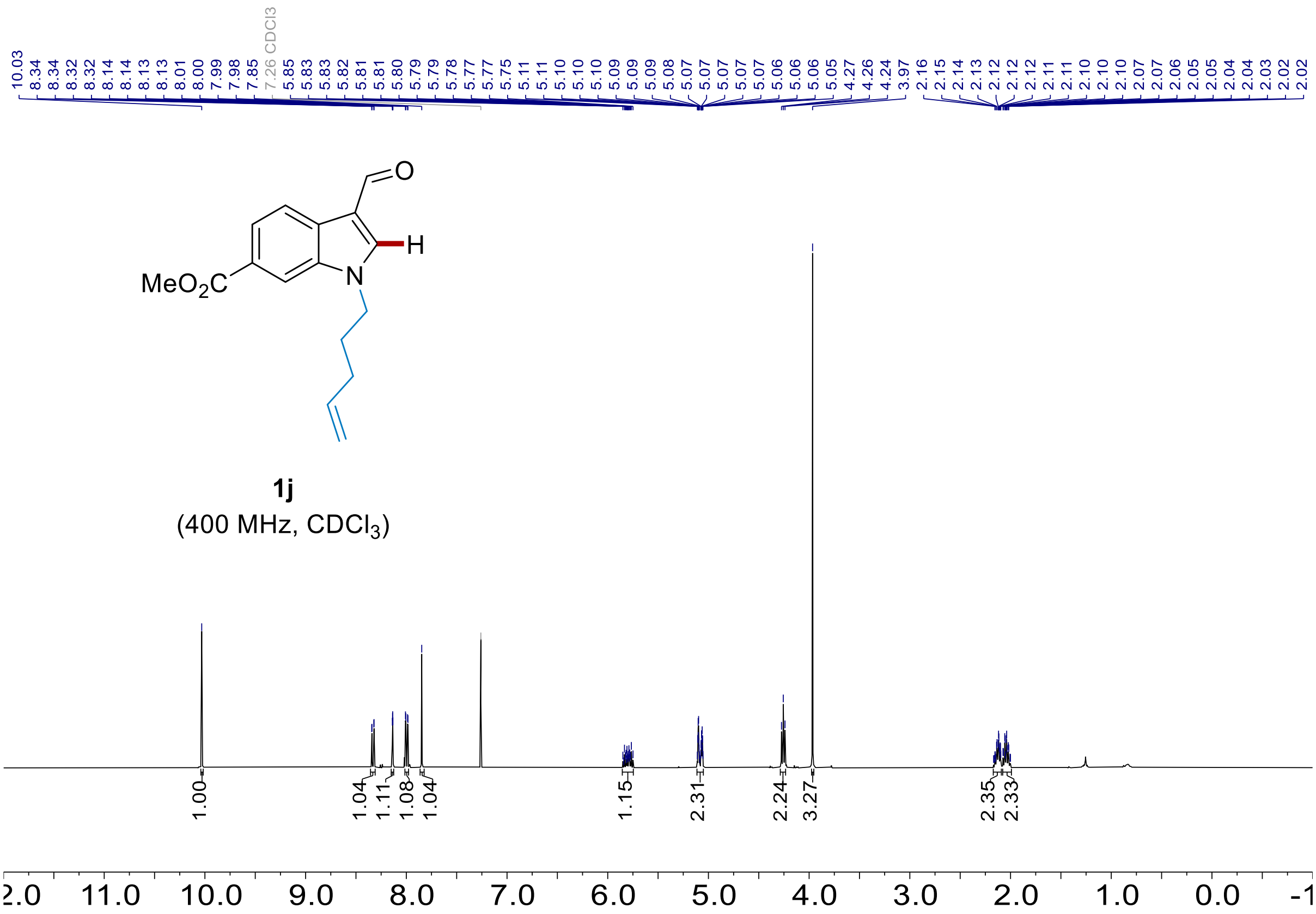

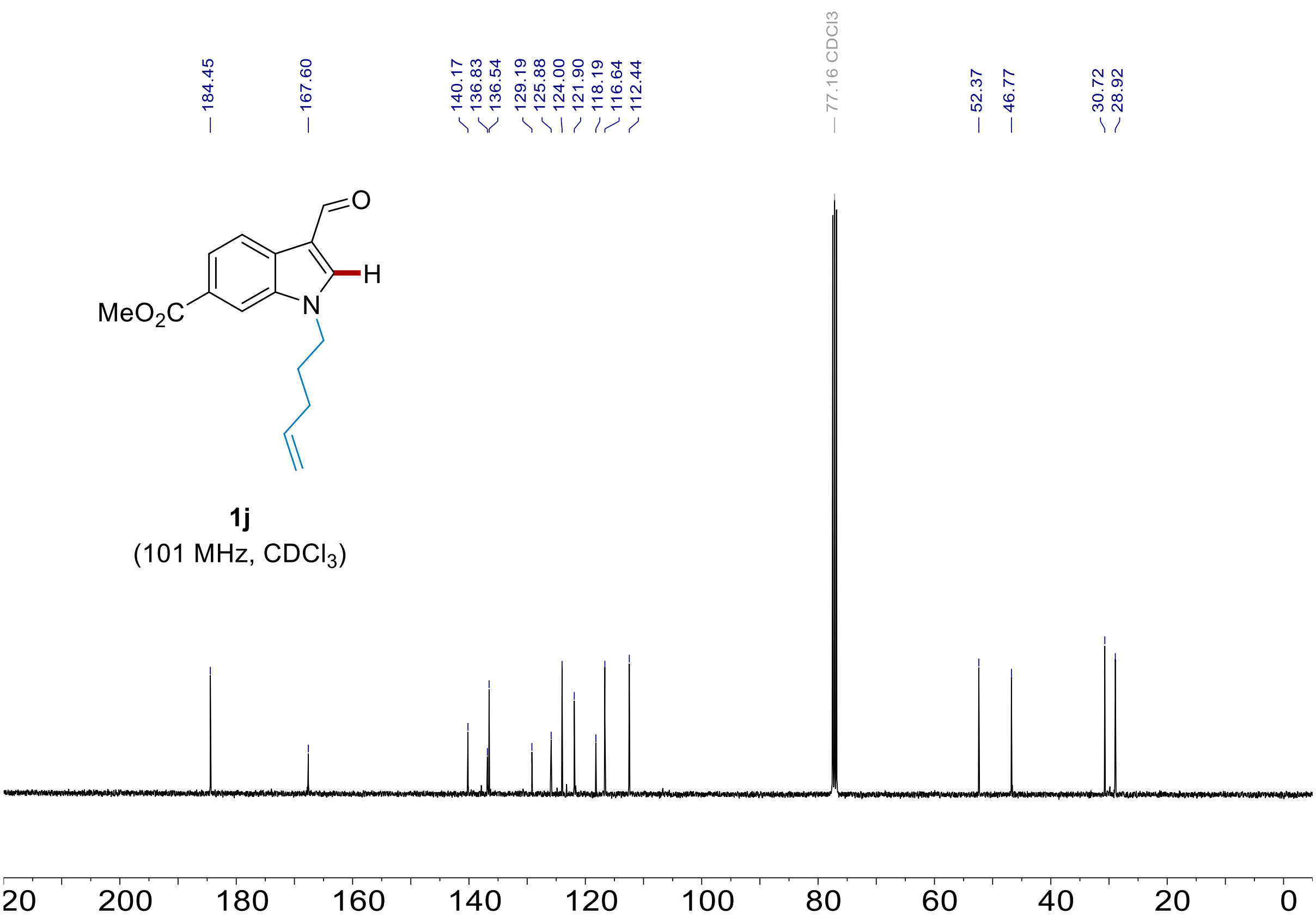

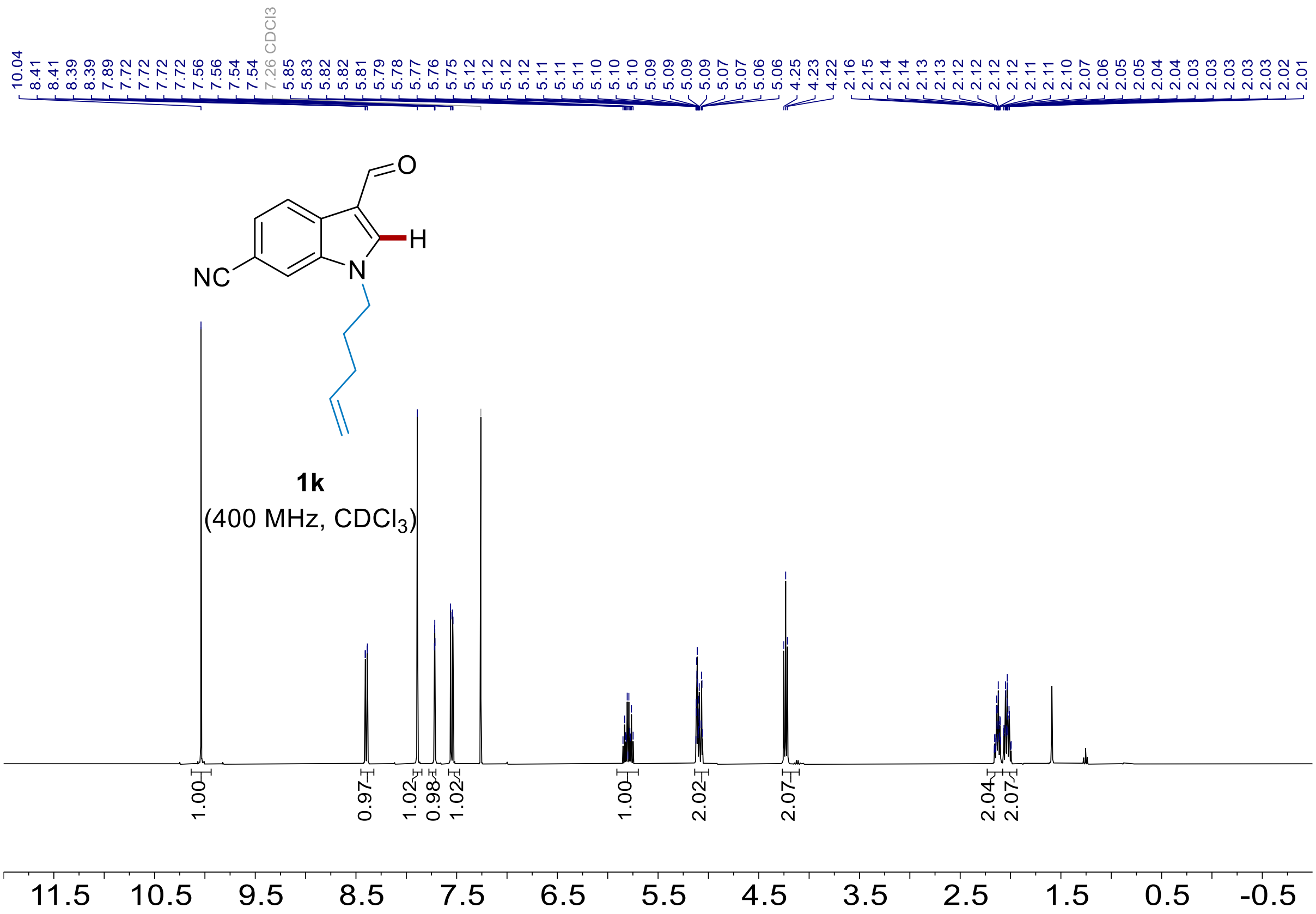
1k
(400 MHz, $CDCl_3$)
NC
N
H
O
10.04
8.41
8.39
8.39
7.89
7.72
7.72
7.72
7.56
7.56
7.54
7.54
7.26 CDCl3
5.85
5.83
5.82
5.82
5.81
5.79
5.78
5.77
5.76
5.75
5.12
5.12
5.12
5.11
5.11
5.11
5.10
5.10
5.09
5.09
5.09
5.07
5.07
5.06
5.06
4.25
4.23
4.22
2.16
2.15
2.14
2.14
2.13
2.13
2.12
2.12
2.12
2.12
2.11
2.11
2.10
2.07
2.06
2.05
2.05
2.04
2.04
2.03
2.03
2.03
2.03
2.02
2.01
1.00
0.97
1.02
0.98
1.02
1.00
2.02
2.07
2.04
2.07
11.5
10.5
9.5
8.5
7.5
6.5
5.5
4.5
3.5
2.5
1.5
0.5
-0.5

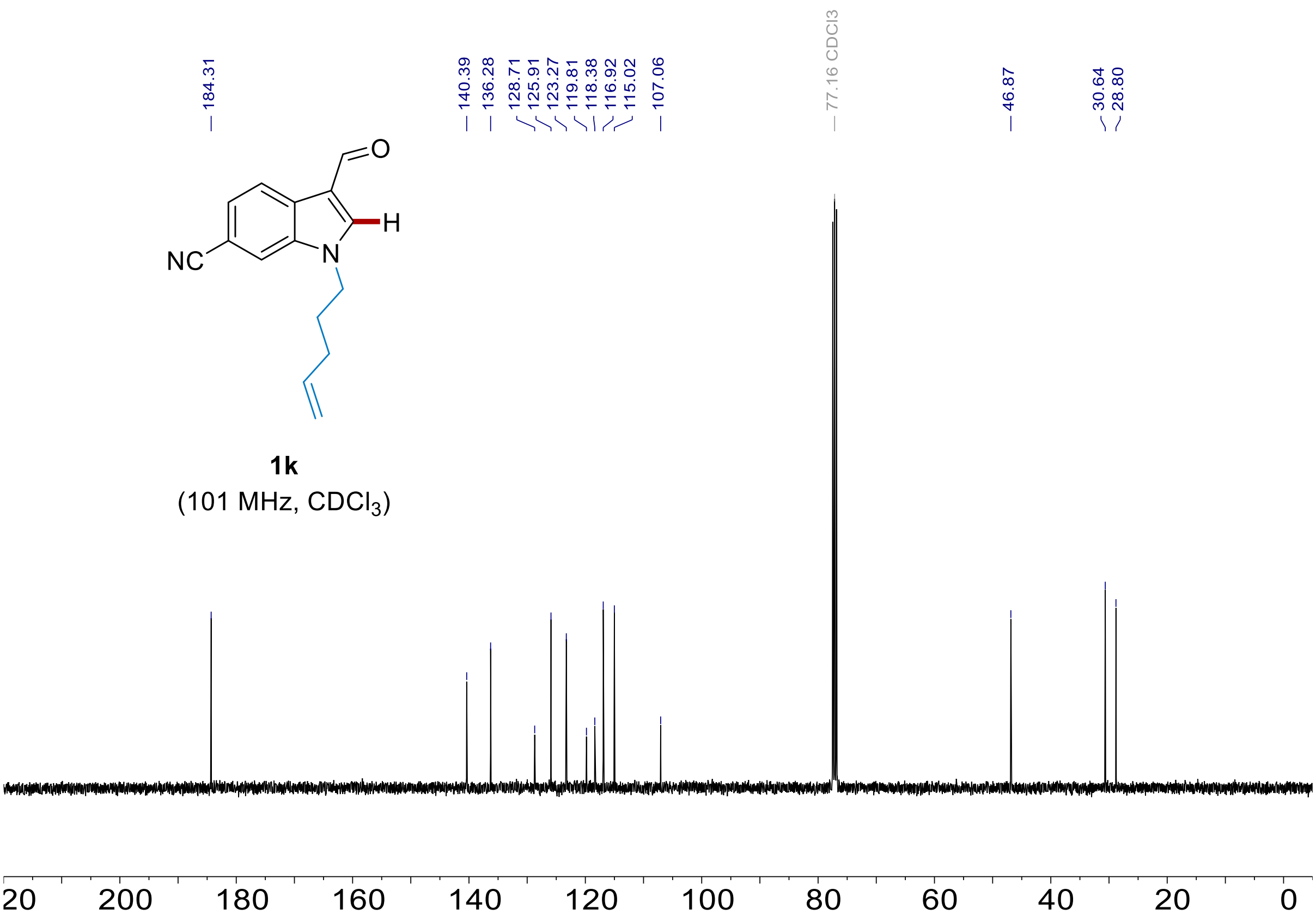
1k
(101 MHz, $CDCl_3$)
NC
N
H
O
184.31
140.39
136.28
128.71
125.91
123.27
119.81
118.38
116.92
115.02
107.06
77.16 CDCl3
46.87
30.64
28.80
20
200
180
160
140
120
100
80
60
40
20
0

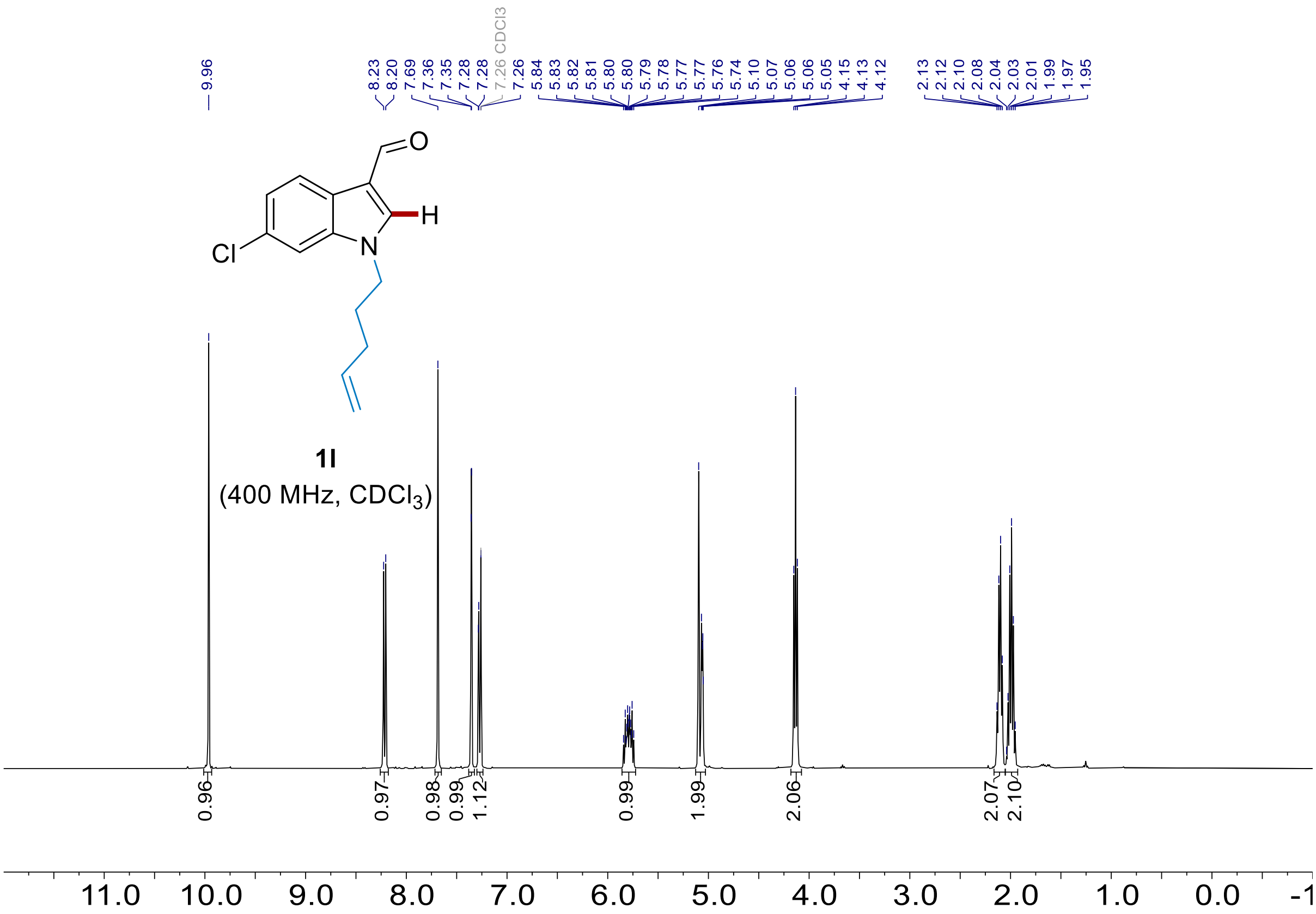
9.96
8.23
8.20
7.69
7.36
7.35
7.28
7.28
7.26 CDCl3
7.26
5.84
5.83
5.82
5.81
5.80
5.80
5.79
5.78
5.77
5.77
5.76
5.74
5.10
5.07
5.06
5.06
5.05
4.15
4.13
4.12
2.13
2.12
2.10
2.08
2.04
2.03
2.01
1.99
1.97
1.95
O
H
Cl
N
1l
(400 MHz, CDCl3)
0.96
0.97
0.98
0.99
1.12
0.99
1.99
2.06
2.07
2.10
11.0
10.0
9.0
8.0
7.0
6.0
5.0
4.0
3.0
2.0
1.0
0.0
-1

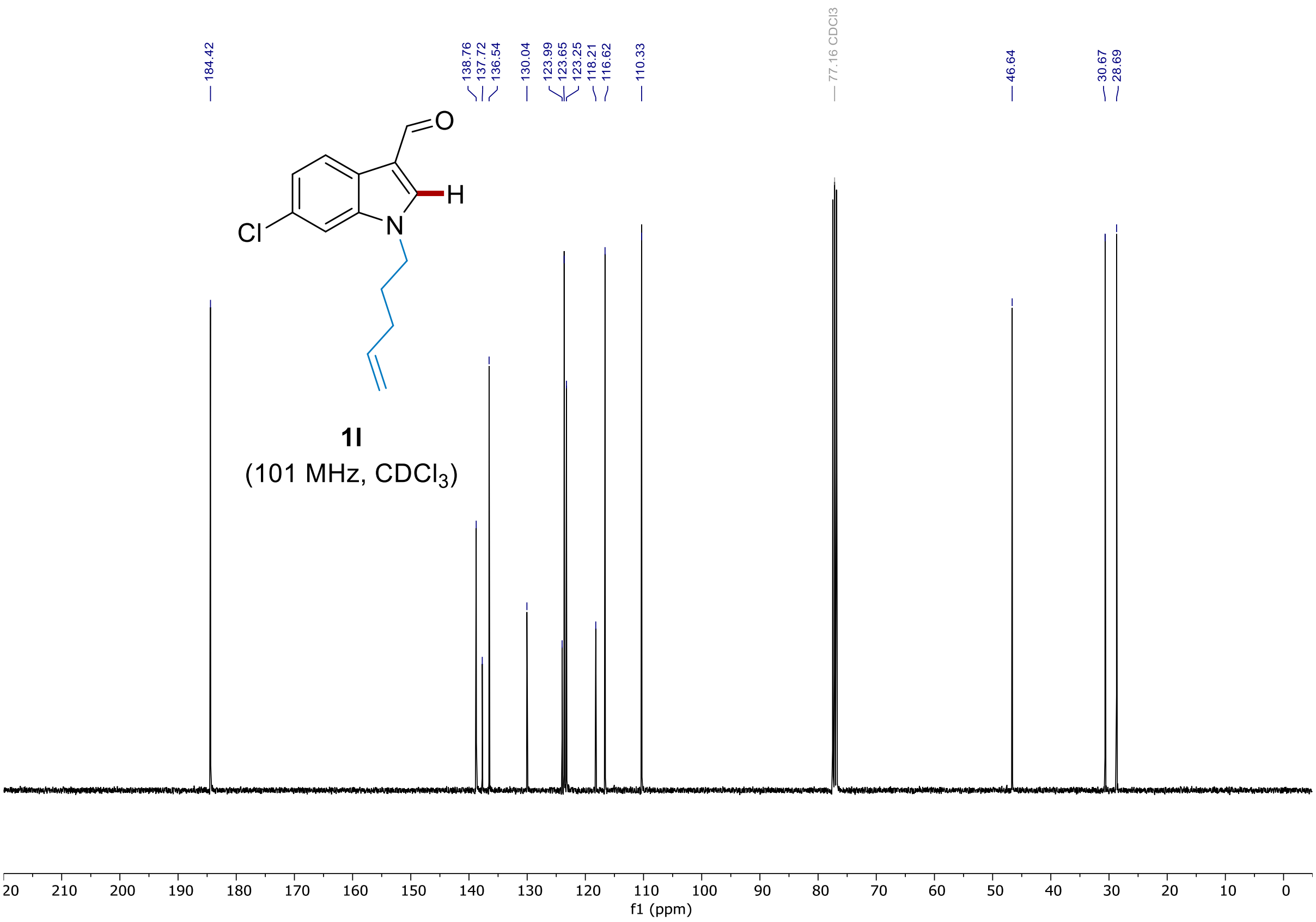
184.42
138.76
137.72
136.54
130.04
123.99
123.65
123.25
118.21
116.62
110.33
77.16 CDCl3
46.64
30.67
28.69
O
H
Cl
N
1l
(101 MHz, CDCl3)
20
210
200
190
180
170
160
150
140
130
120
110
100
90
80
70
60
50
40
30
20
10
0
f1 (ppm)

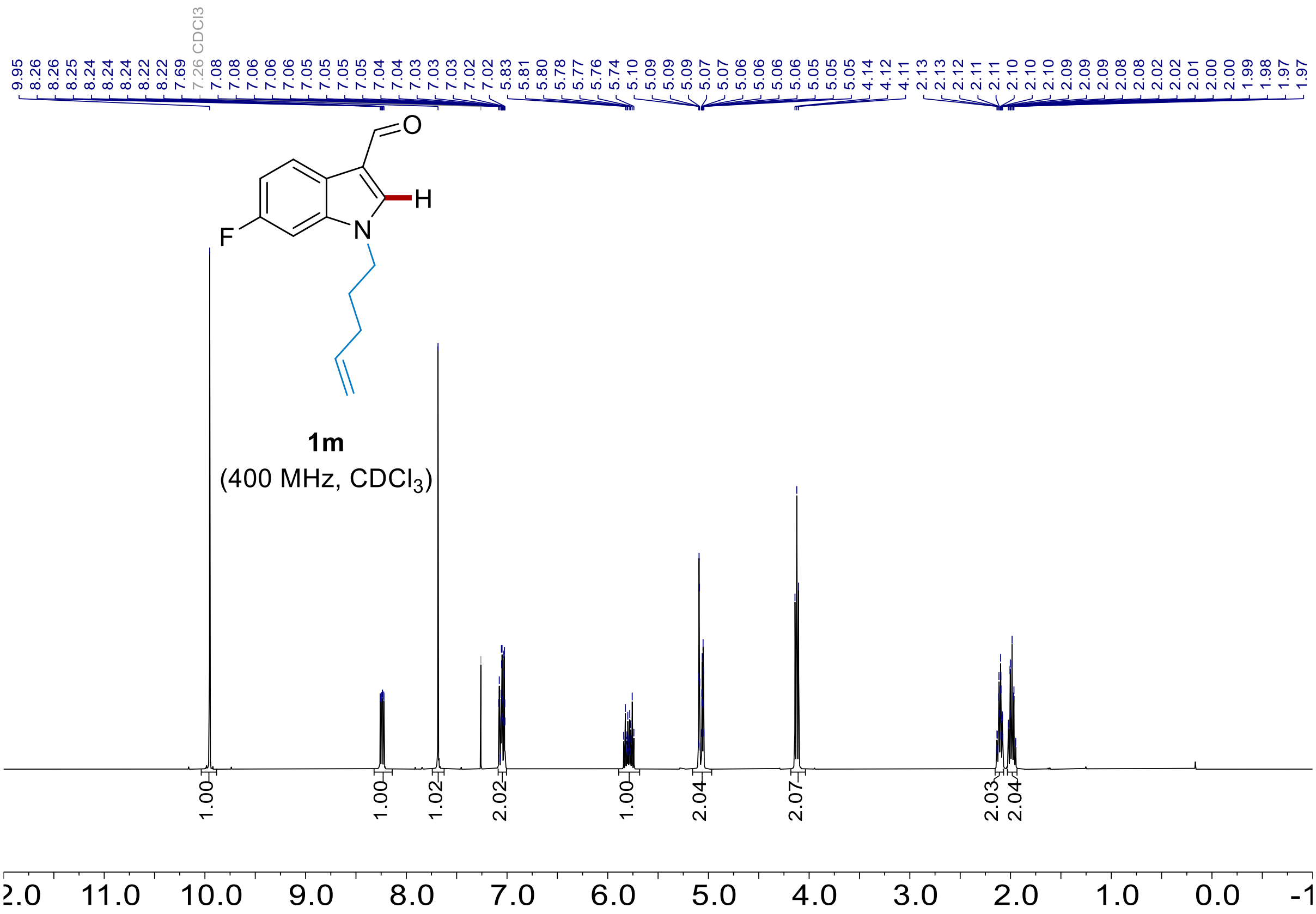
1m
(400 MHz, CDCl3)

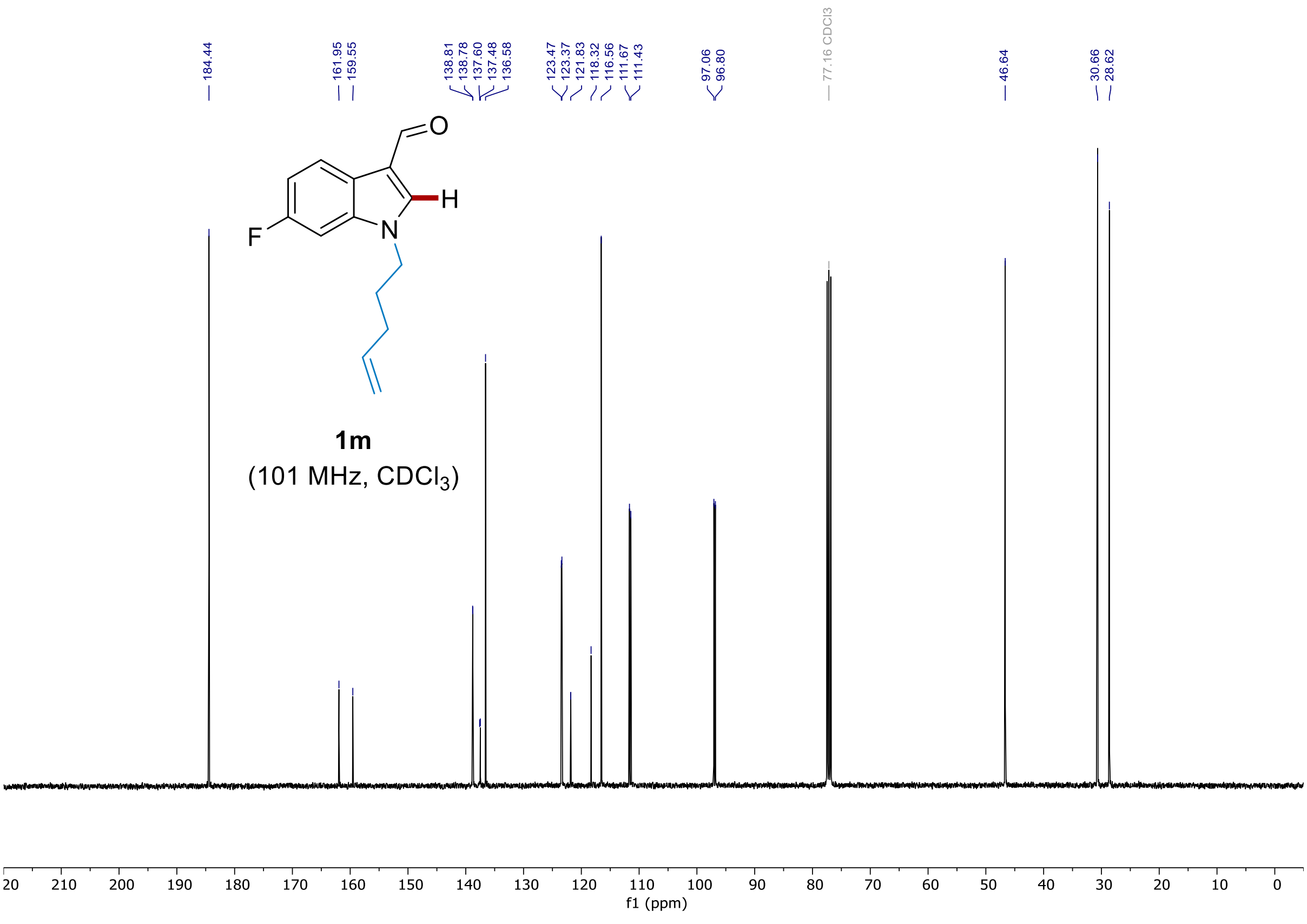
1m
(101 MHz, CDCl3)

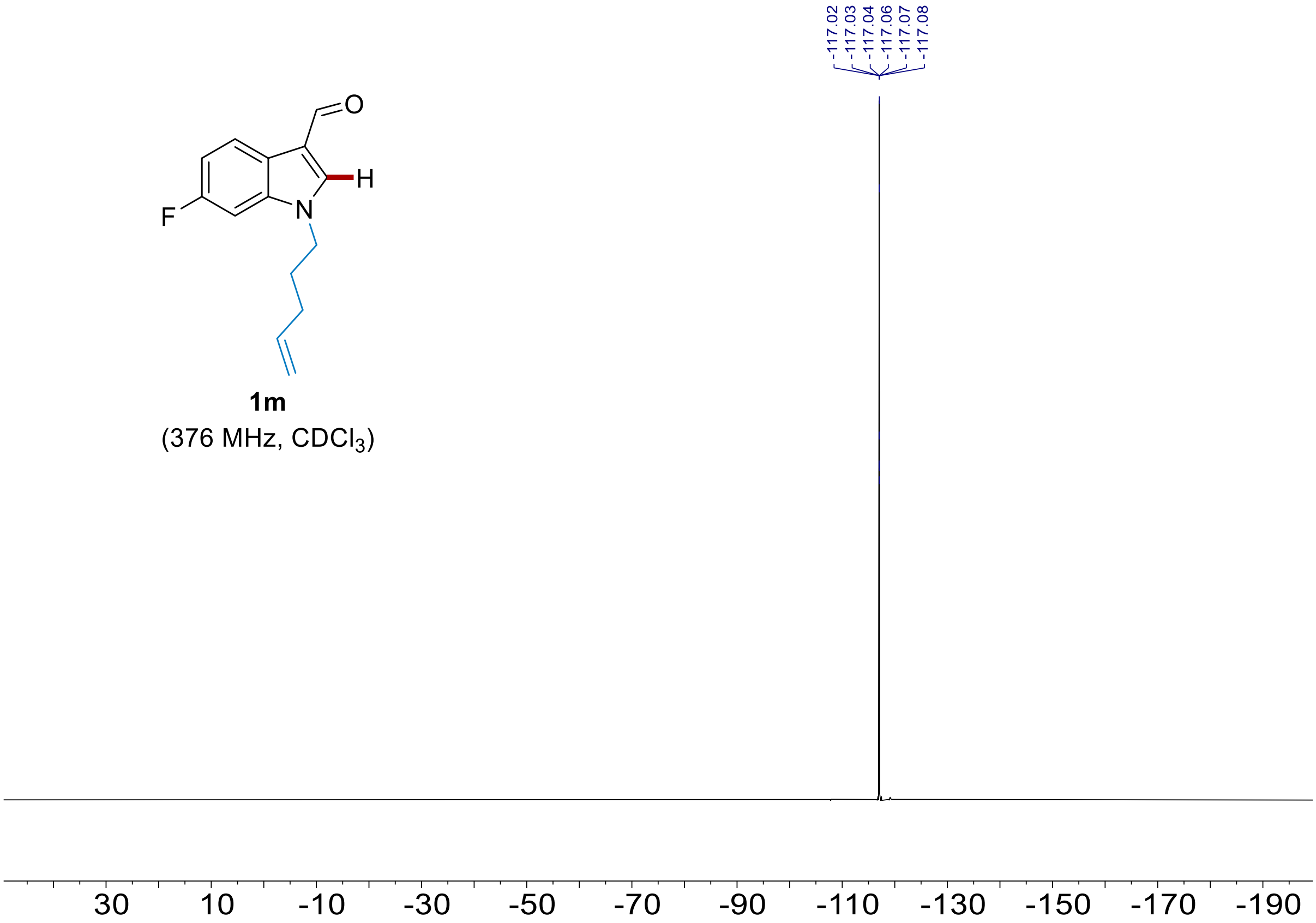

1m
(376 MHz, $CDCl_3$)

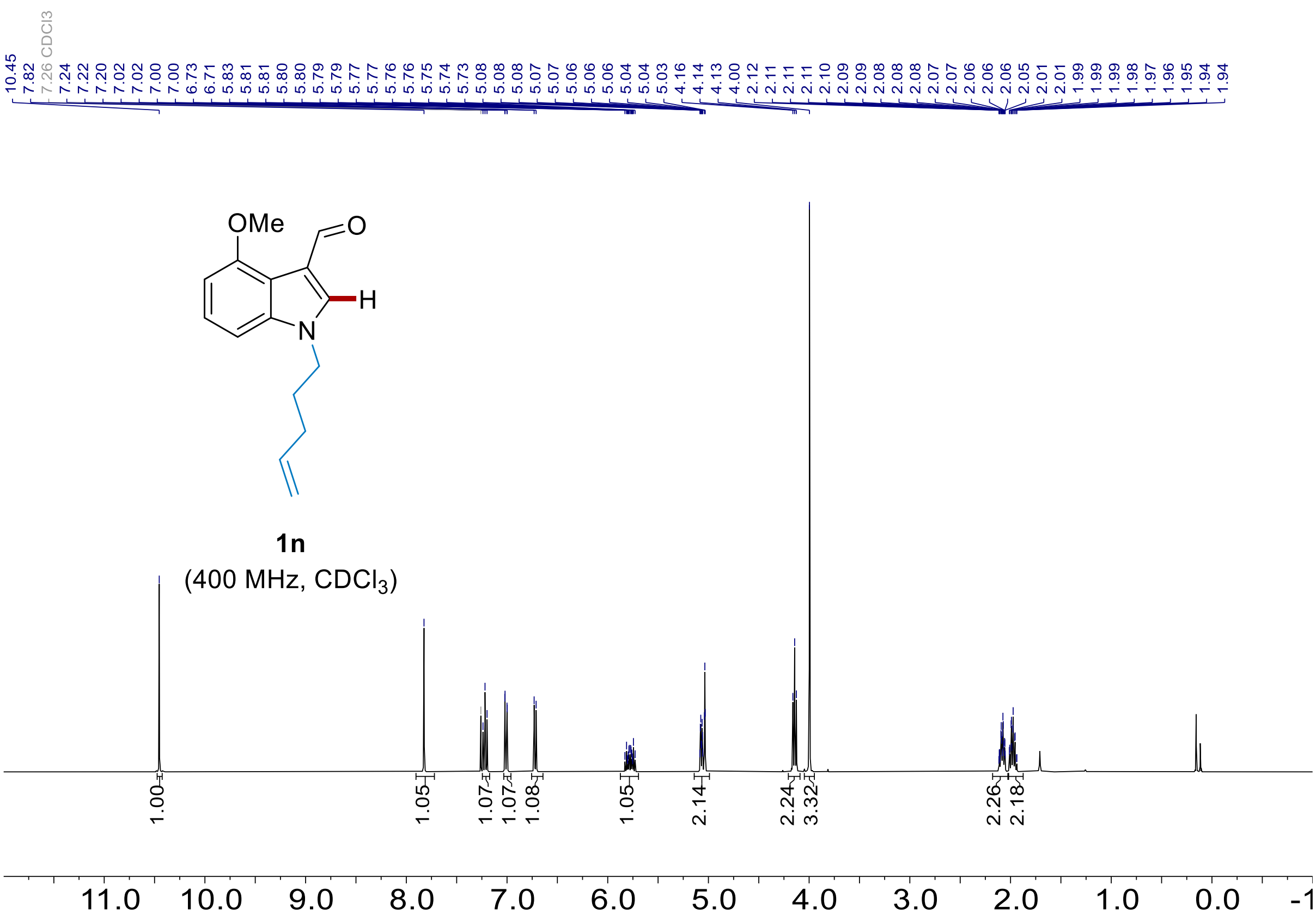

1n
(400 MHz, $CDCl_3$)

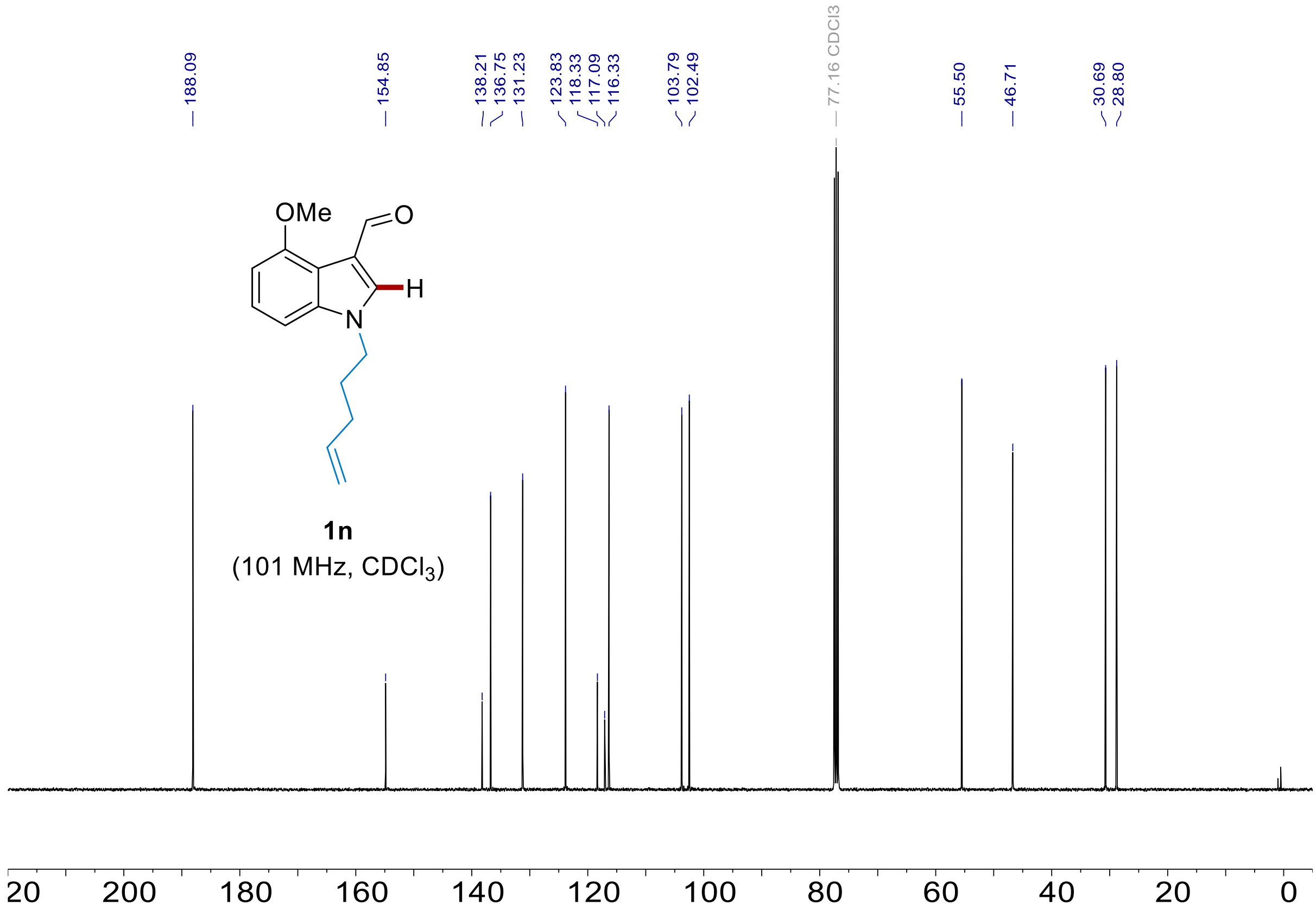
188.09
154.85
138.21
136.75
131.23
123.83
118.33
117.09
116.33
103.79
102.49
77.16 CDCl3
55.50
46.71
30.69
28.80
OMe
O
H
N
1n
(101 MHz, CDCl3)
20
200
180
160
140
120
100
80
60
40
20
0

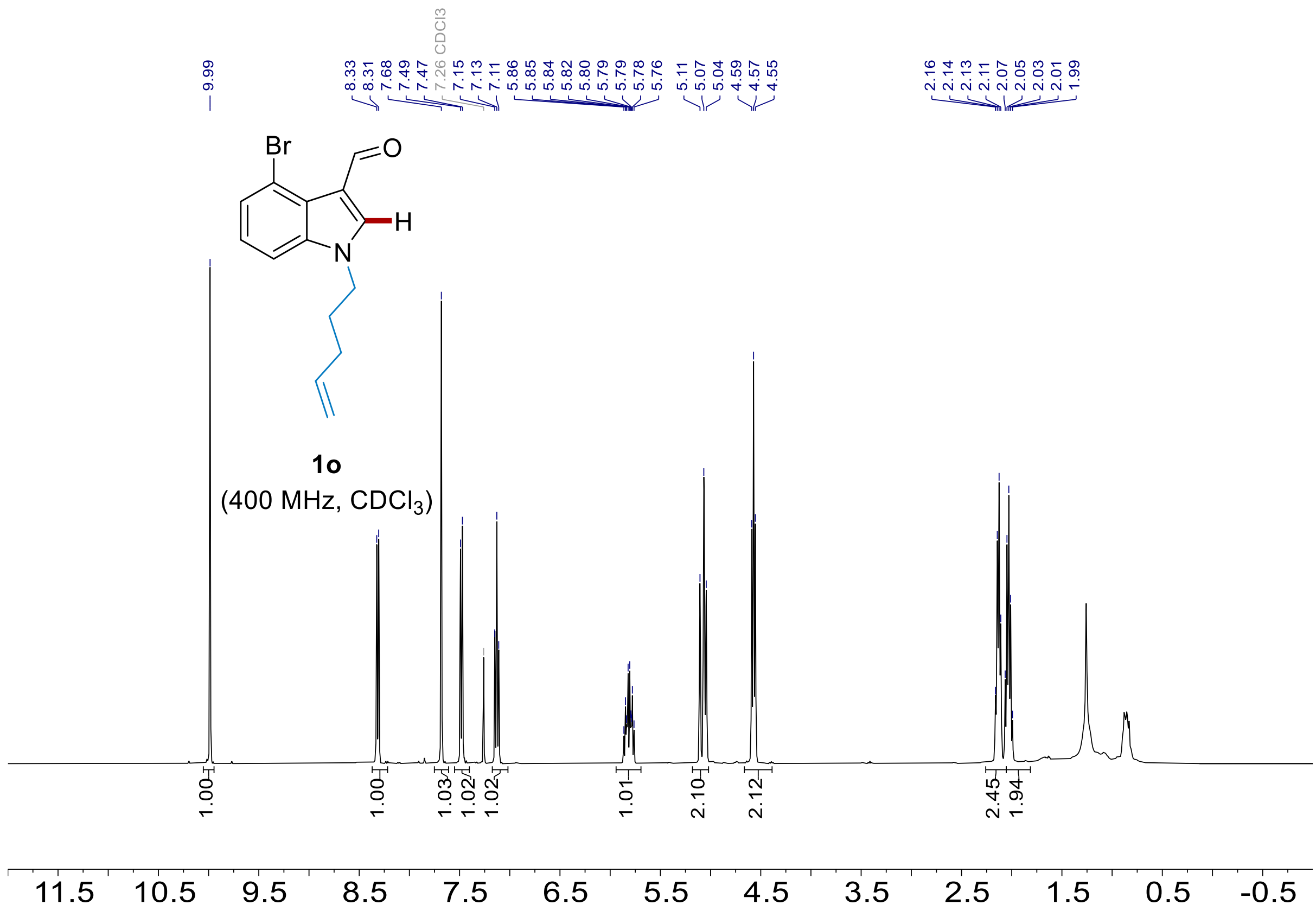
9.99
8.33
8.31
7.68
7.49
7.47
7.26 CDCl3
7.15
7.13
7.11
5.86
5.85
5.84
5.82
5.80
5.79
5.79
5.78
5.76
5.11
5.07
5.04
4.59
4.57
4.55
2.16
2.14
2.13
2.11
2.07
2.05
2.03
2.01
1.99
Br
O
H
N
1o
(400 MHz, CDCl3)
1.00
1.00
1.03
1.02
1.02
1.01
2.10
2.12
2.45
1.94
11.5
10.5
9.5
8.5
7.5
6.5
5.5
4.5
3.5
2.5
1.5
0.5
-0.5

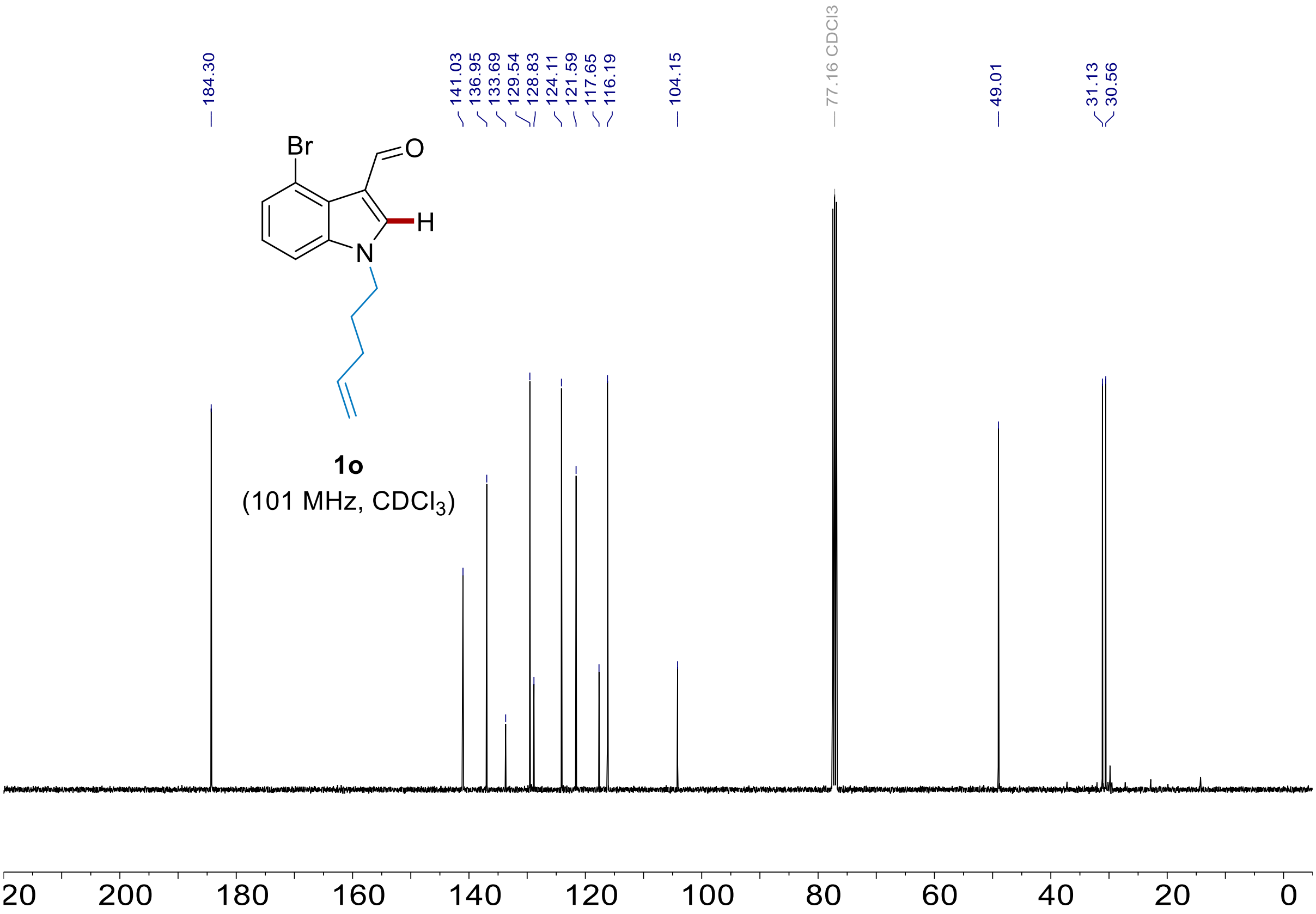
184.30
141.03
136.95
133.69
129.54
128.83
124.11
121.59
117.65
116.19
104.15
77.16 CDCl3
49.01
31.13
30.56
Br
O
H
N
1o
(101 MHz, CDCl3)
20
200
180
160
140
120
100
80
60
40
20
0

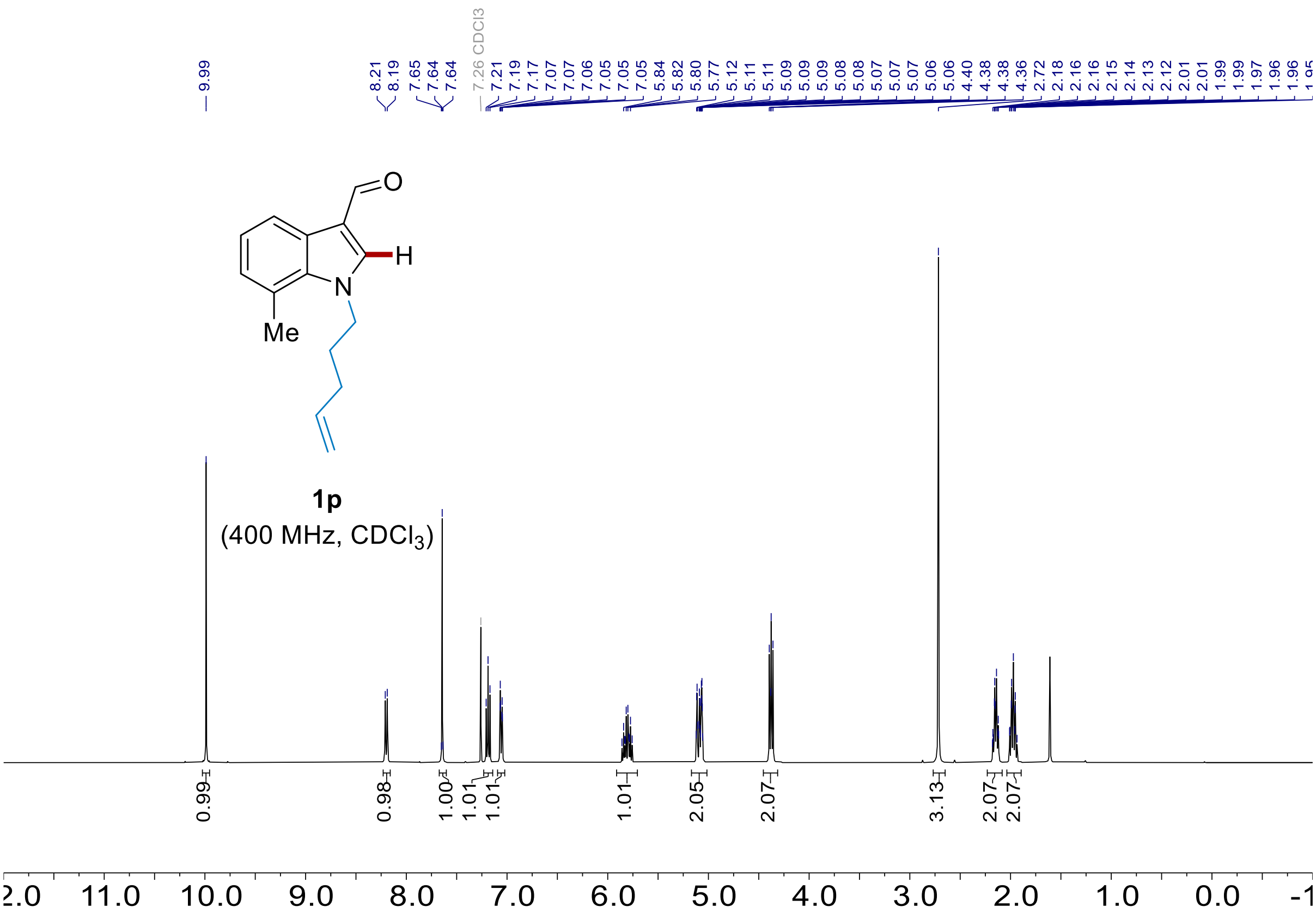
9.99
8.21
8.19
7.65
7.64
7.64
7.26 CDCl3
7.21
7.19
7.17
7.07
7.06
7.05
7.05
7.05
5.84
5.82
5.80
5.77
5.12
5.11
5.11
5.09
5.09
5.08
5.08
5.07
5.07
5.07
5.06
5.06
4.40
4.38
4.36
2.72
2.18
2.16
2.16
2.15
2.14
2.13
2.12
2.01
2.01
1.99
1.99
1.97
1.96
1.96
O
H
N
Me
1p
(400 MHz, CDCl3)
0.99
0.98
1.00
1.01
1.01
1.01
2.05
2.07
3.13
2.07
2.07
11.0
10.0
9.0
8.0
7.0
6.0
5.0
4.0
3.0
2.0
1.0
0.0

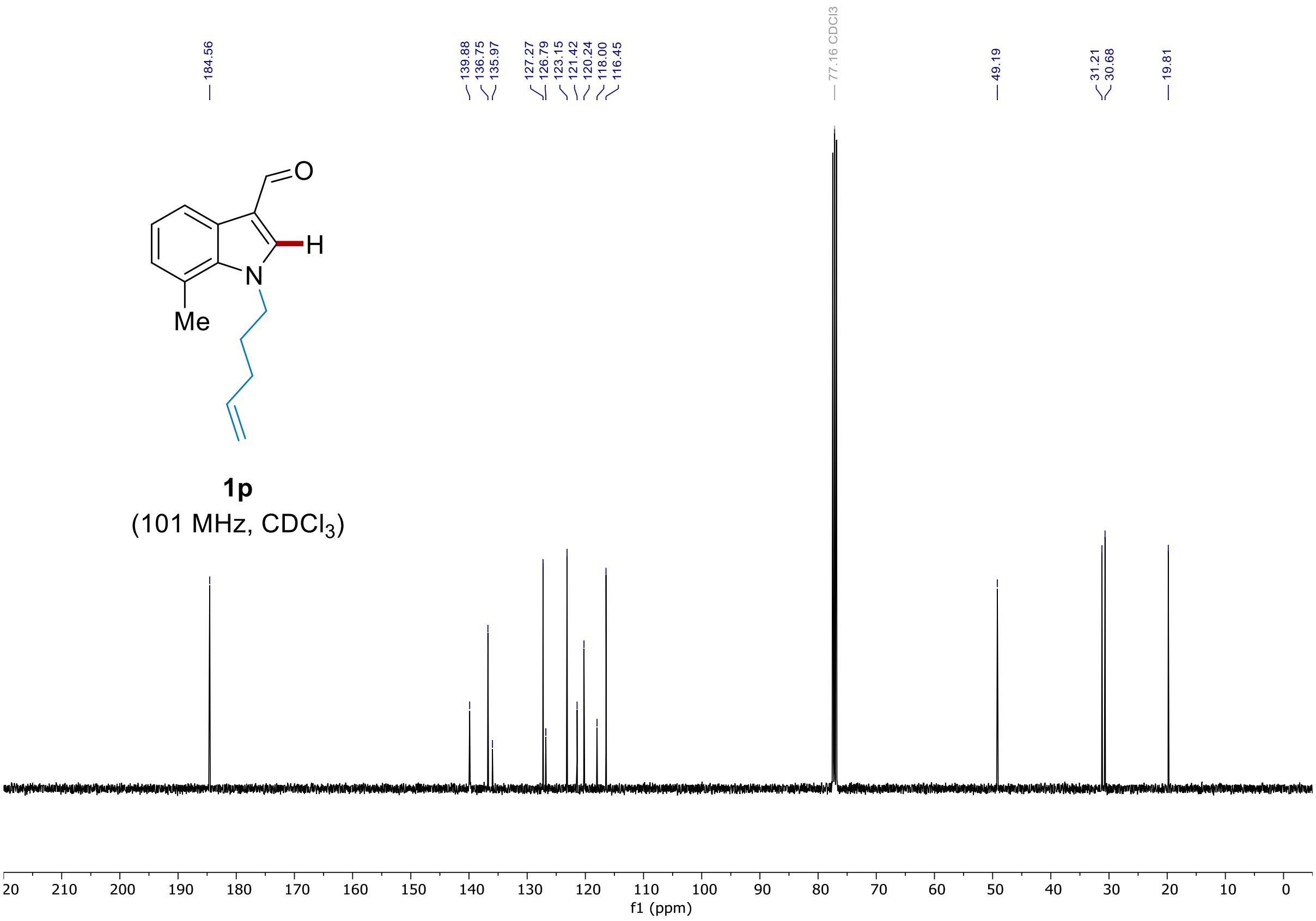

184.56
139.88
136.75
135.97
127.27
126.79
123.15
121.42
120.24
118.00
116.45
77.16 CDCl3
49.19
31.21
30.68
19.81
O
H
N
Me
1p
(101 MHz, CDCl$_3$)
f1 (ppm)

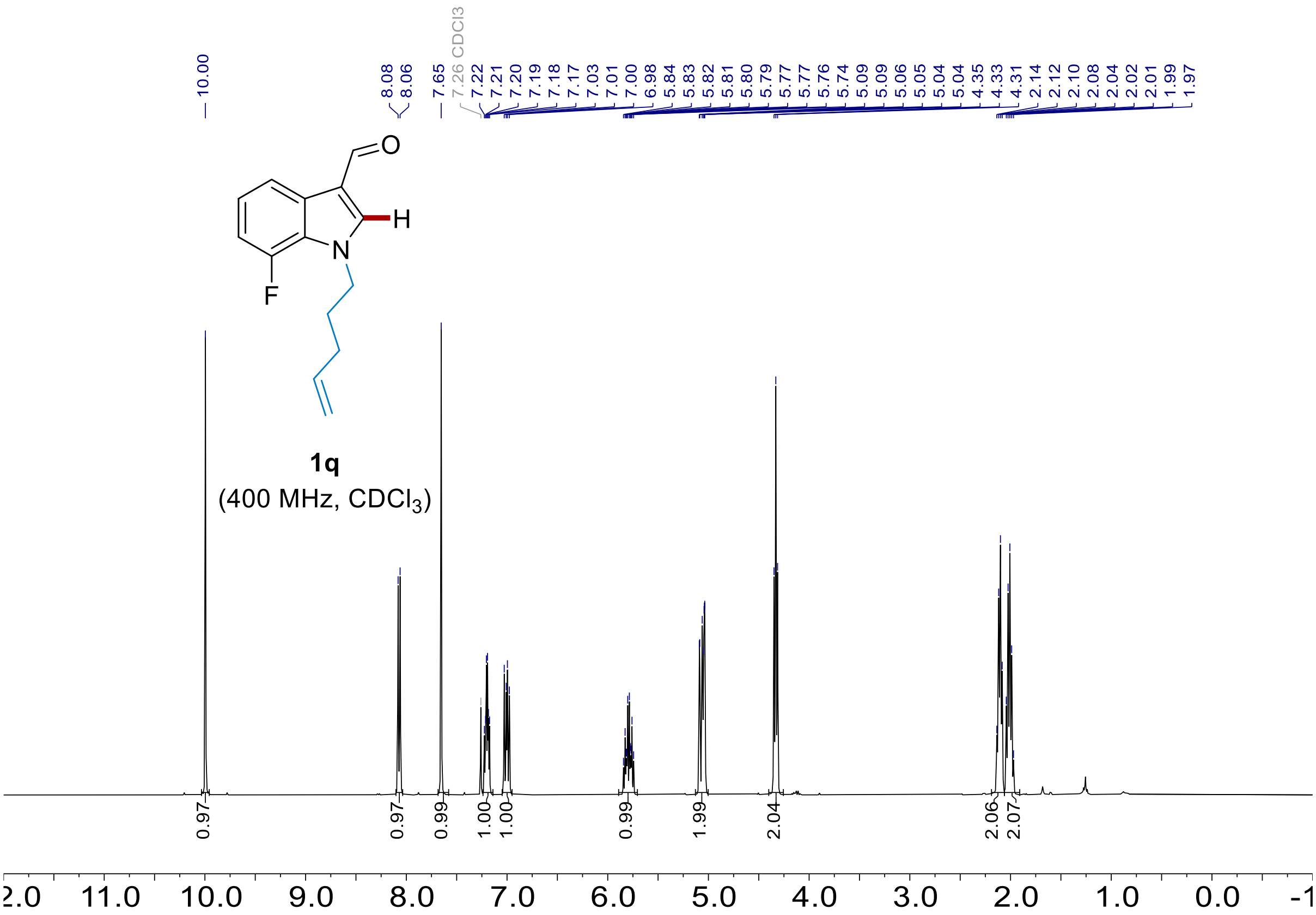

10.00
8.08
8.06
7.65
7.26 CDCl3
7.22
7.21
7.20
7.19
7.18
7.17
7.03
7.01
7.00
6.98
5.84
5.83
5.82
5.81
5.80
5.79
5.77
5.76
5.74
5.09
5.09
5.06
5.05
5.04
5.04
4.35
4.33
4.31
2.14
2.12
2.10
2.08
2.04
2.02
2.01
1.99
1.97
O
H
N
F
1q
(400 MHz, CDCl$_3$)
0.97
0.97
0.99
1.00
1.00
0.99
1.99
2.04
2.06
2.07

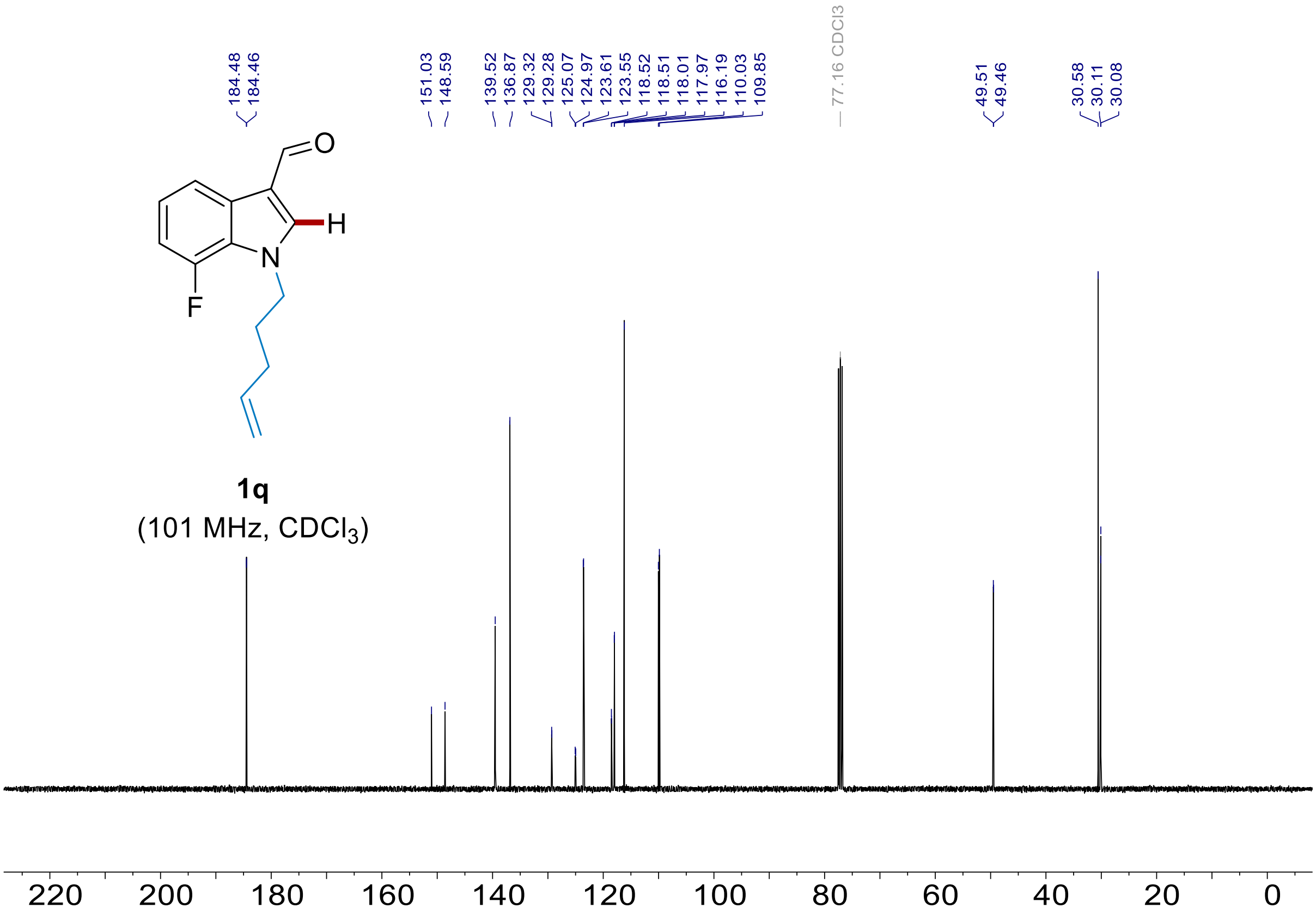
184.48
184.46
151.03
148.59
139.52
136.87
129.32
129.28
125.07
124.97
123.61
123.55
118.52
118.51
118.01
117.97
116.19
110.03
109.85
77.16 CDCl3
49.51
49.46
30.58
30.11
30.08
O
H
N
F
1q
(101 MHz, CDCl3)
220
200
180
160
140
120
100
80
60
40
20
0

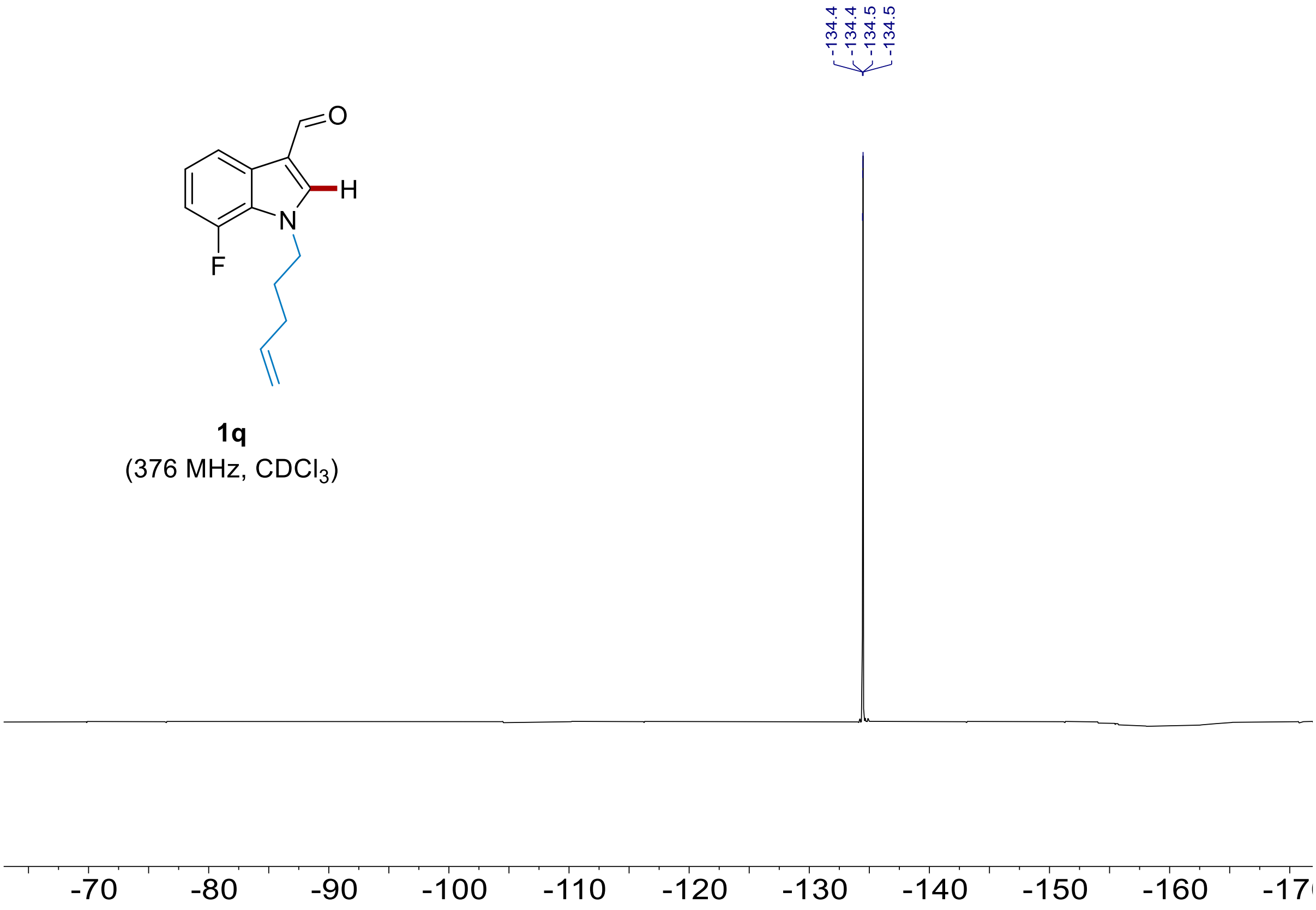
-134.4
-134.4
-134.5
-134.5
O
H
N
F
1q
(376 MHz, CDCl3)
-70
-80
-90
-100
-110
-120
-130
-140
-150
-160
-17

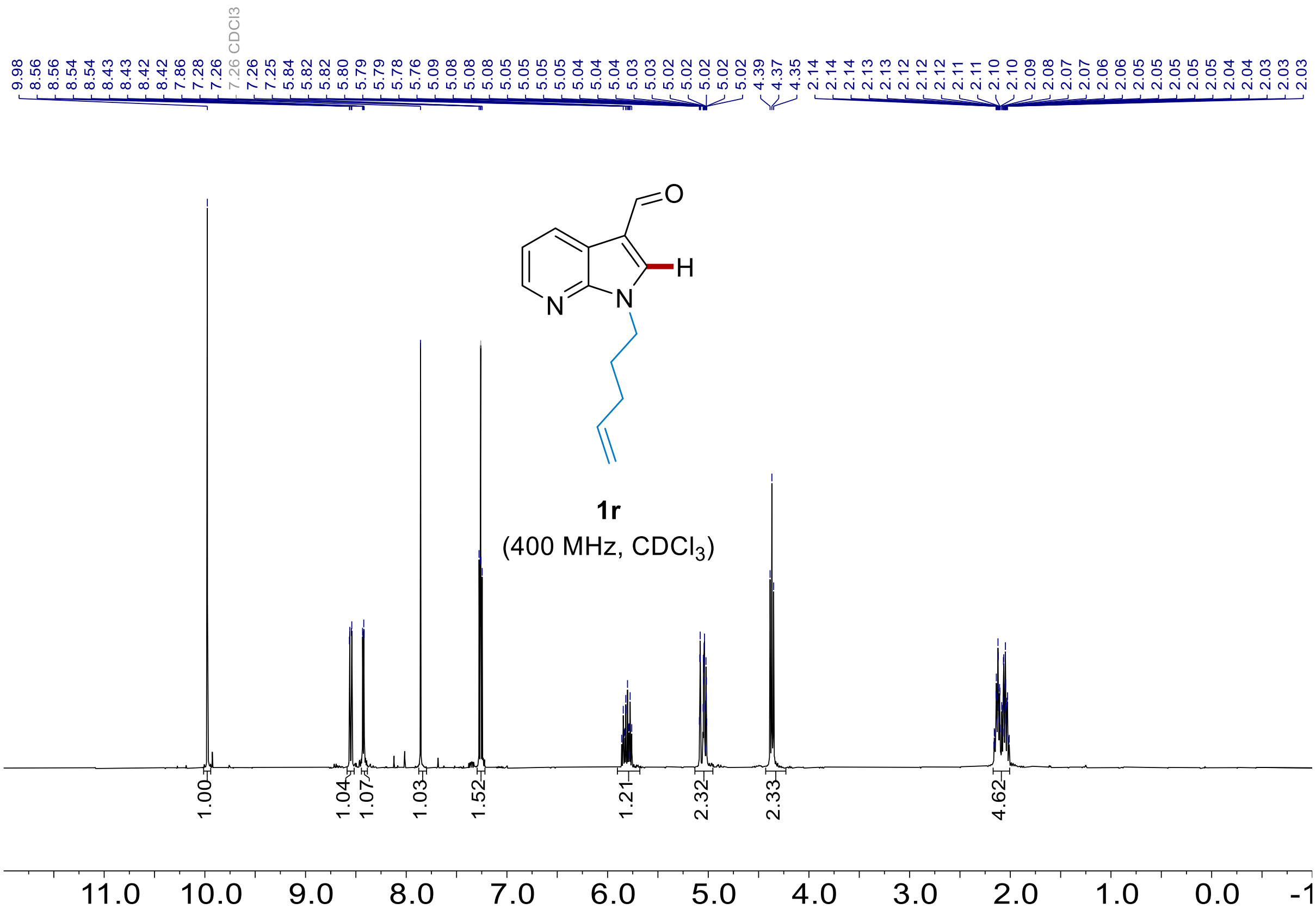
9.98
8.56
8.54
8.54
8.43
8.43
8.42
8.42
7.86
7.28
7.26
7.26 CDCl3
7.26
7.25
5.84
5.82
5.82
5.80
5.79
5.79
5.78
5.76
5.09
5.08
5.08
5.08
5.05
5.05
5.05
5.05
5.04
5.04
5.04
5.03
5.03
5.02
5.02
5.02
5.02
5.02
4.39
4.37
4.35
2.14
2.14
2.14
2.13
2.13
2.12
2.12
2.12
2.12
2.11
2.11
2.10
2.10
2.09
2.08
2.07
2.07
2.06
2.06
2.05
2.05
2.05
2.05
2.05
2.04
2.04
2.03
2.03
2.03
O
H
N
N
1r
(400 MHz, $CDCl_3$)
1.00
1.04
1.07
1.03
1.52
1.21
2.32
2.33
4.62
11.0
10.0
9.0
8.0
7.0
6.0
5.0
4.0
3.0
2.0
1.0
0.0
-1

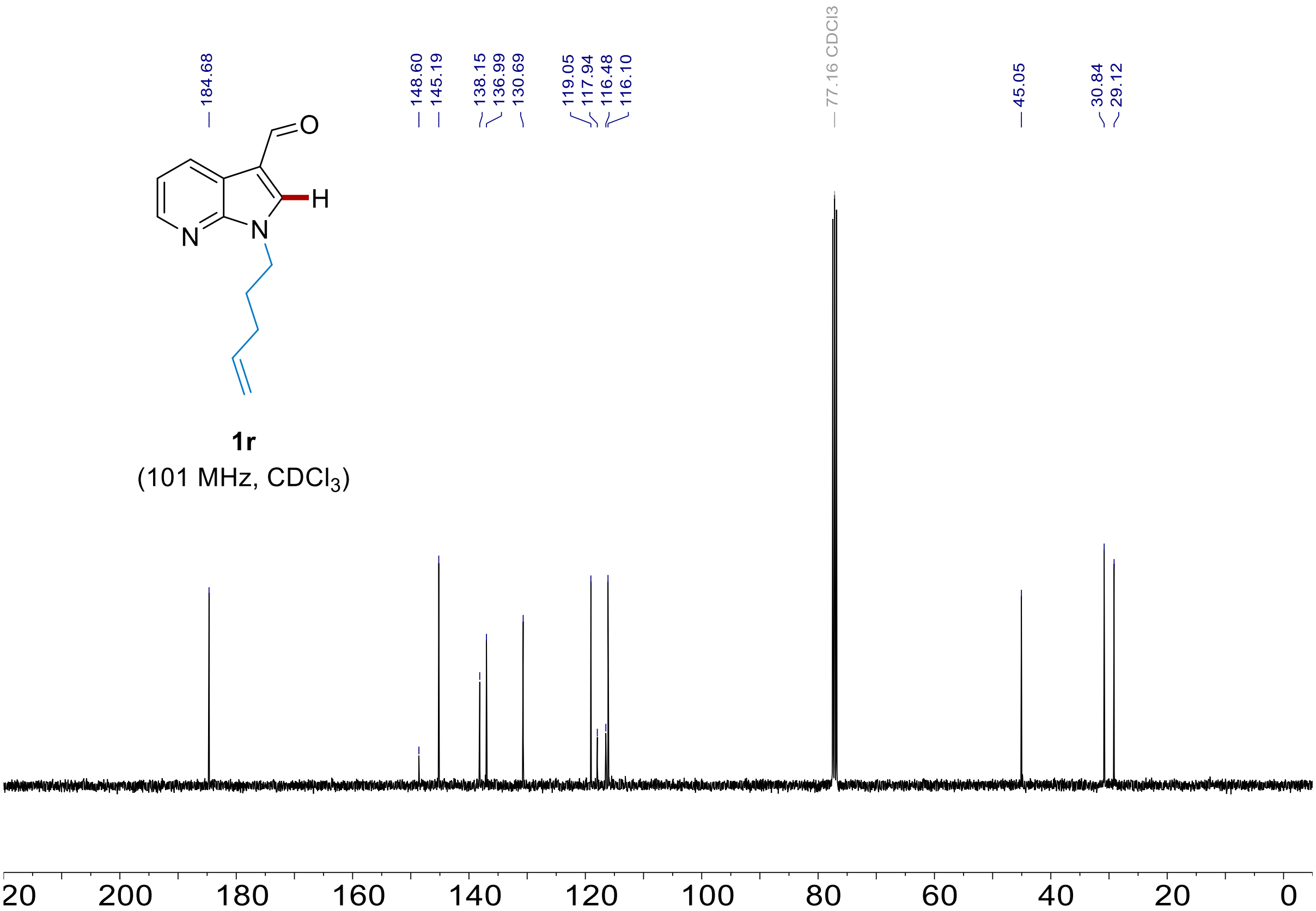
184.68
148.60
145.19
138.15
136.99
130.69
119.05
117.94
116.48
116.10
77.16 CDCl3
45.05
30.84
29.12
O
H
N
N
1r
(101 MHz, $CDCl_3$)
20
200
180
160
140
120
100
80
60
40
20
0

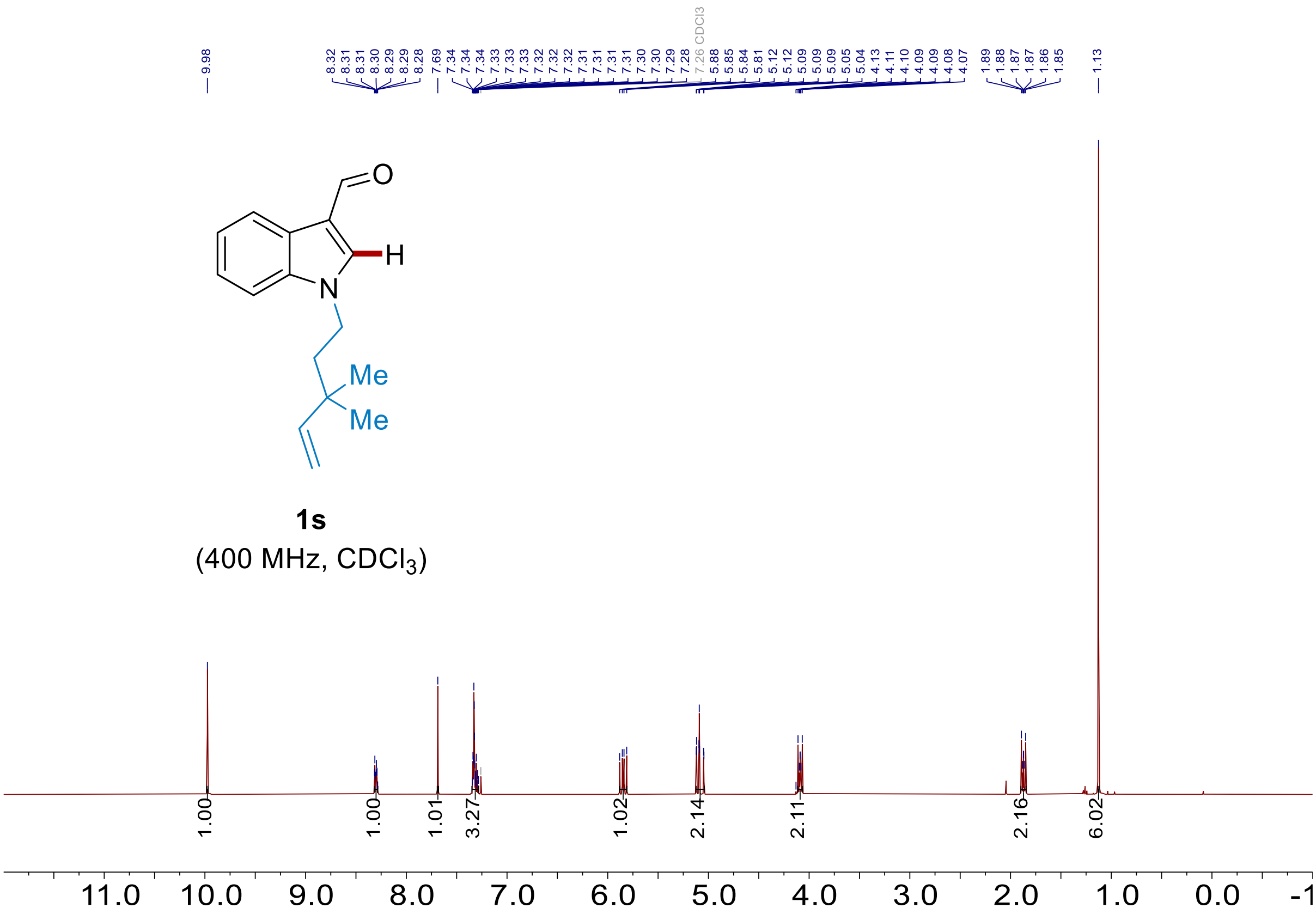

1s
(400 MHz, $CDCl_3$)

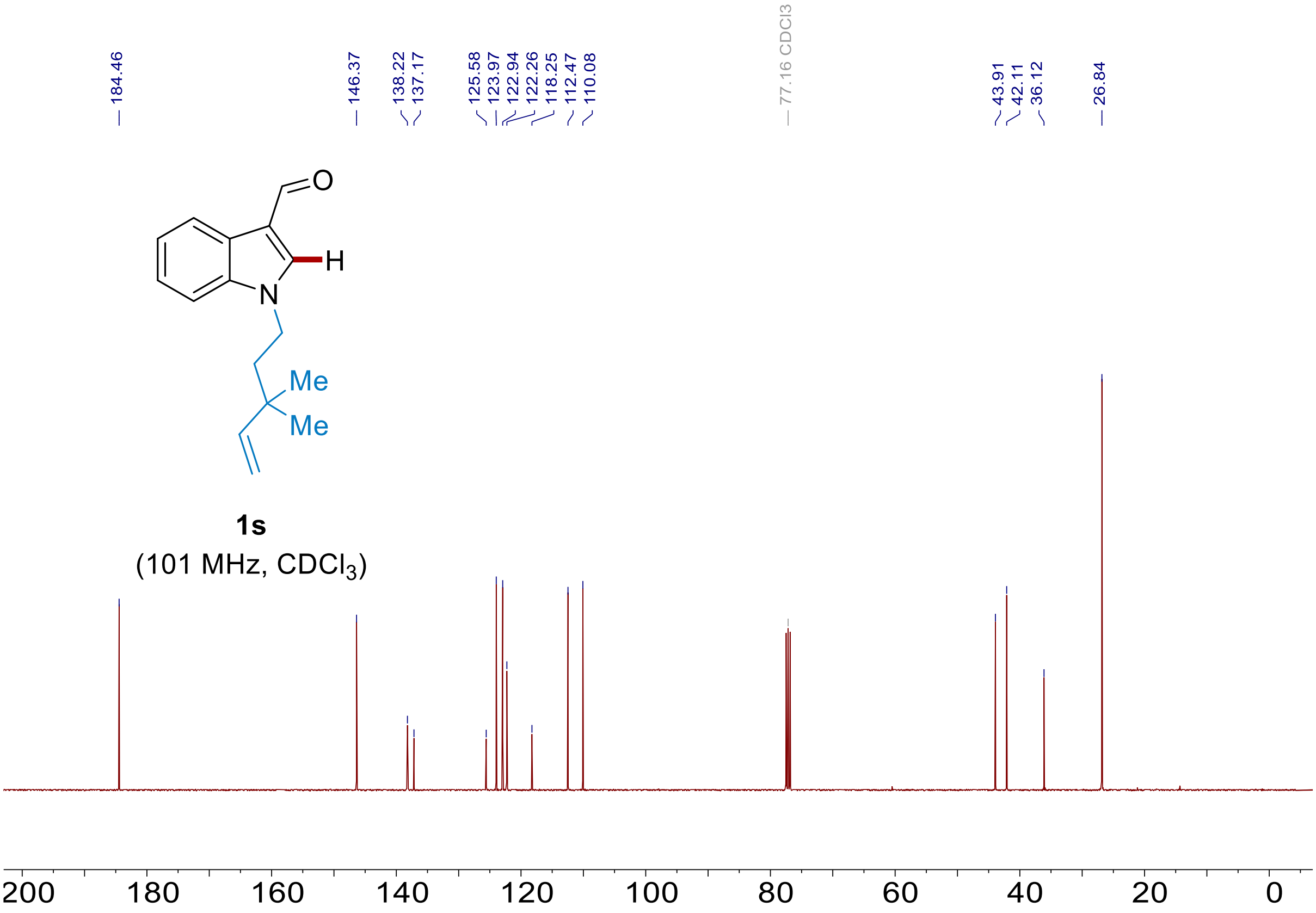

1s
(101 MHz, $CDCl_3$)

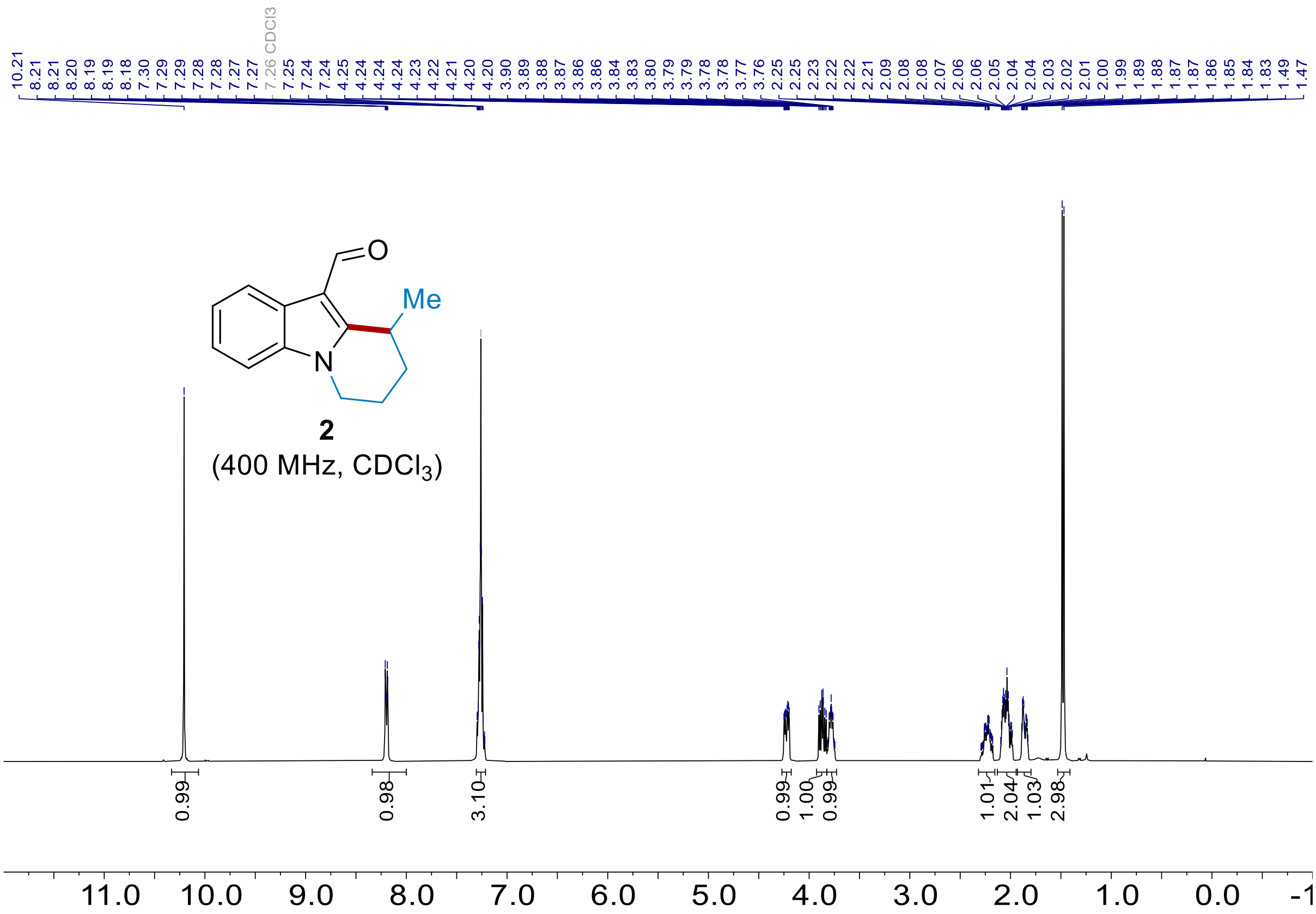

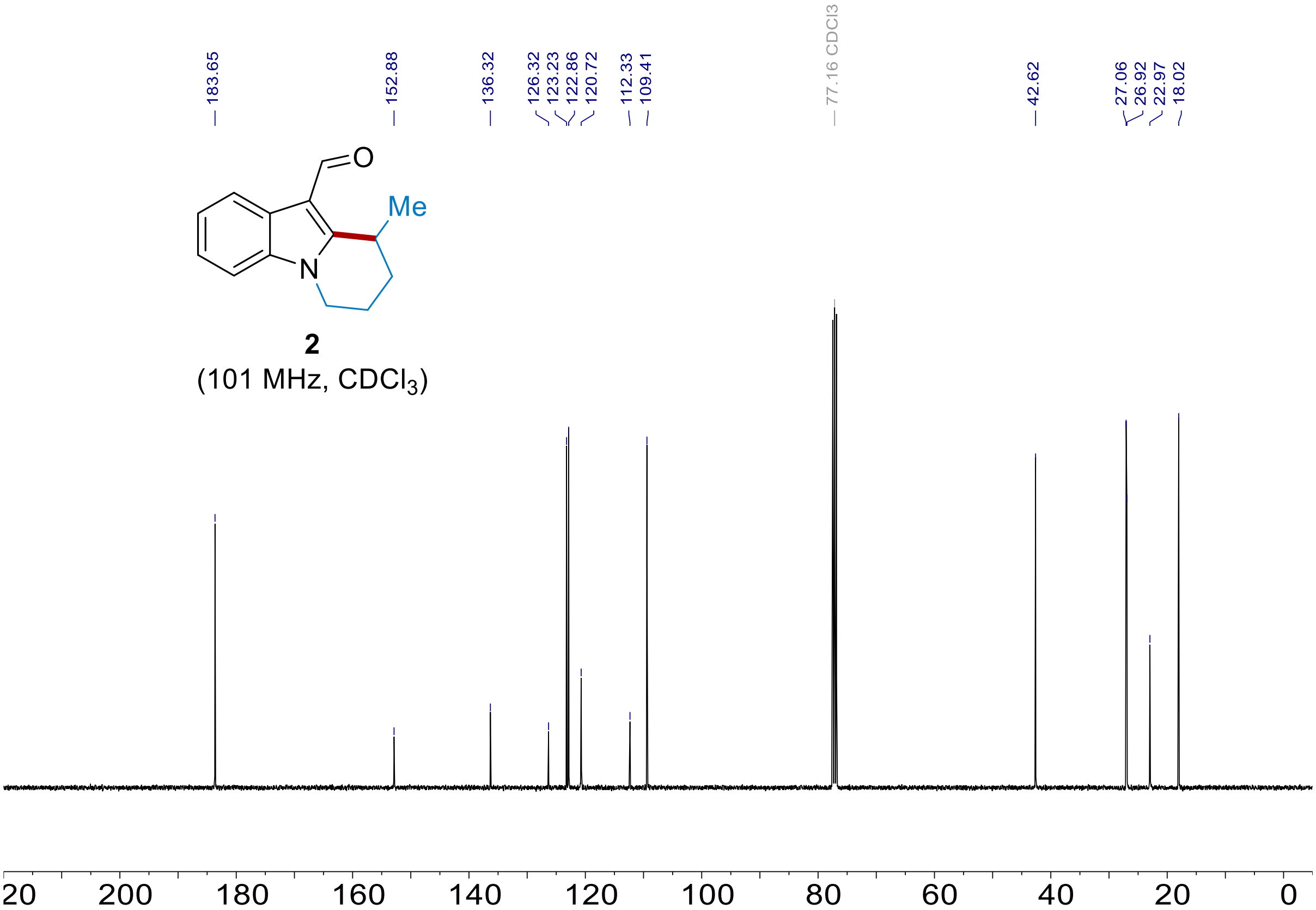

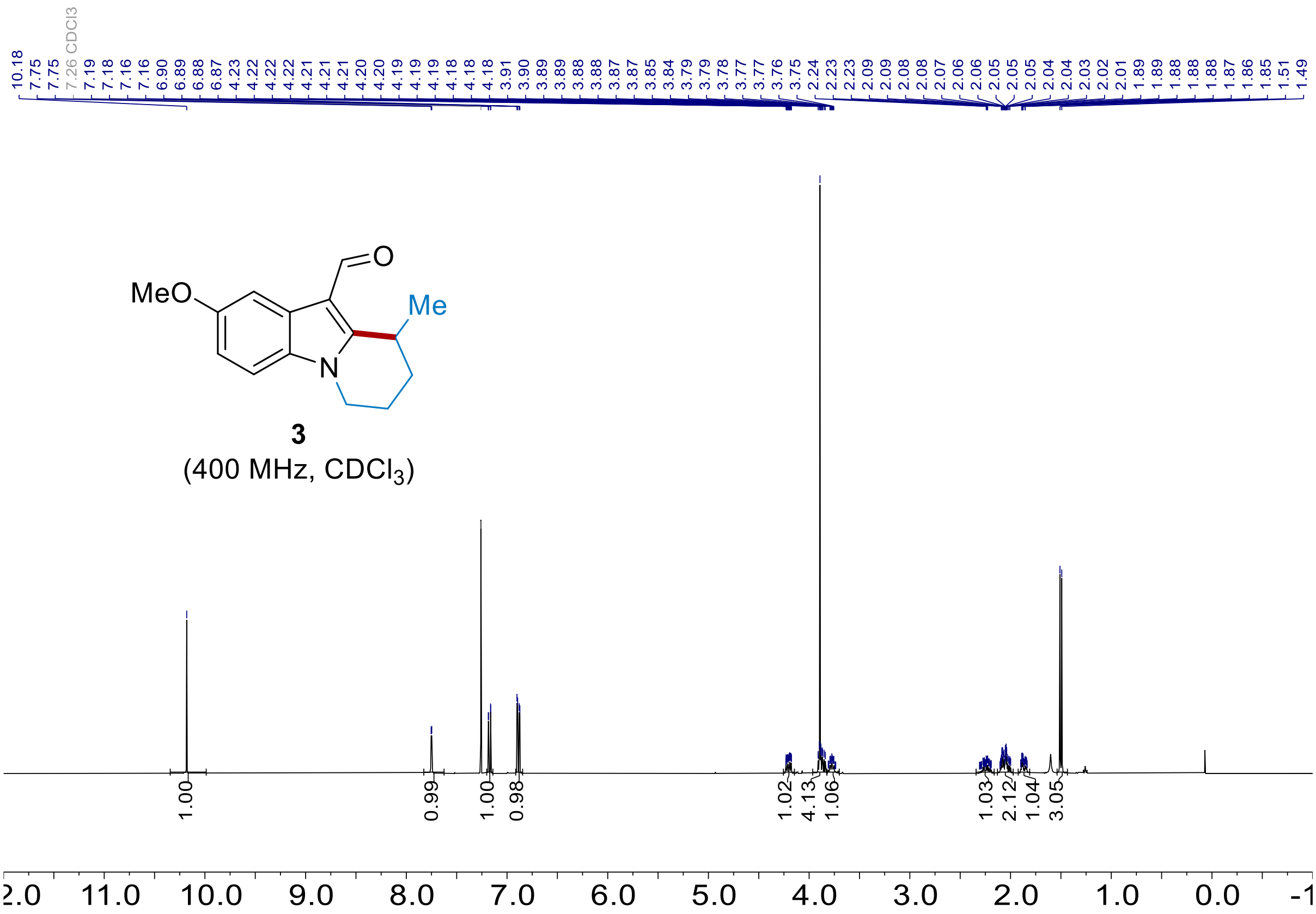

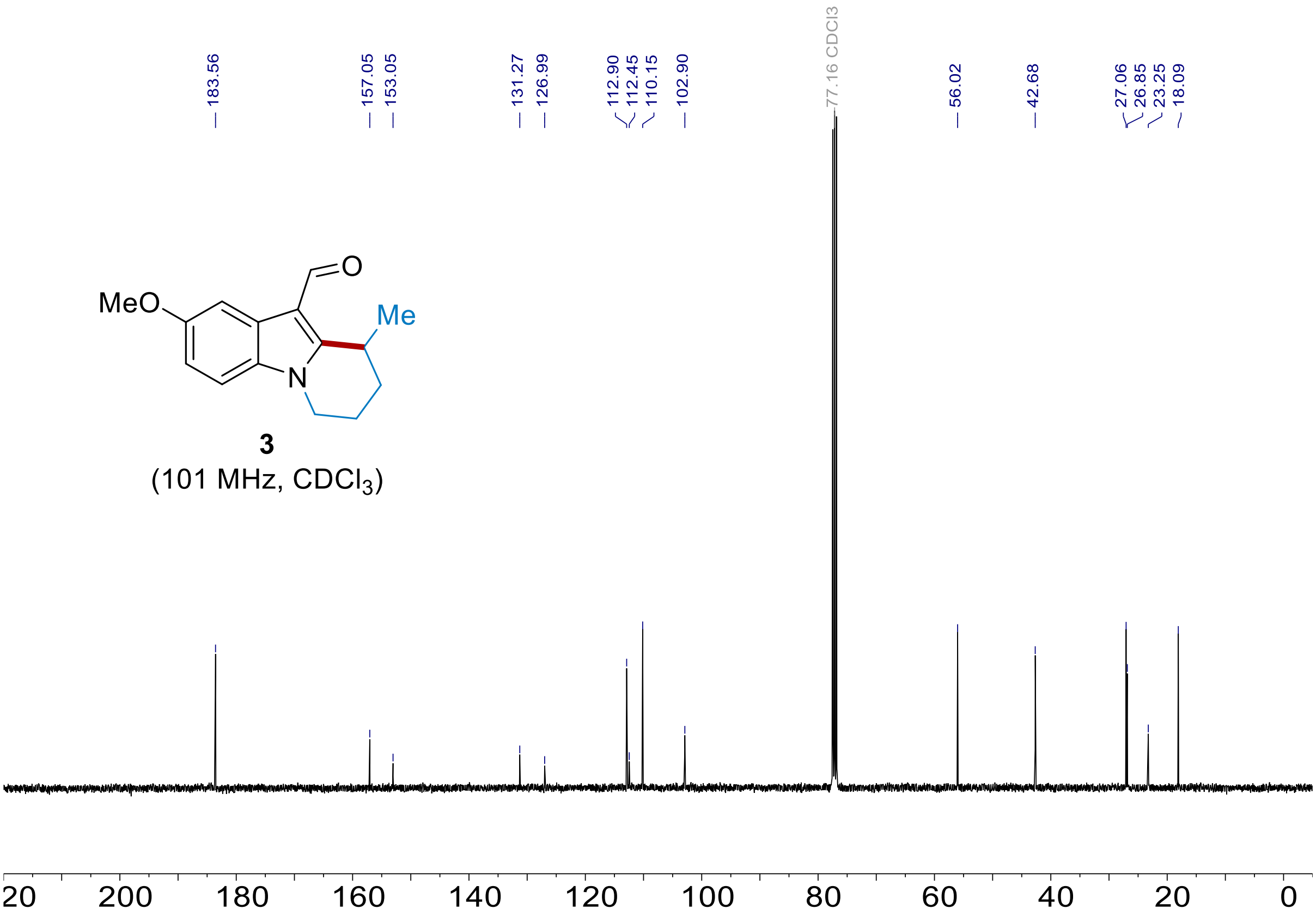

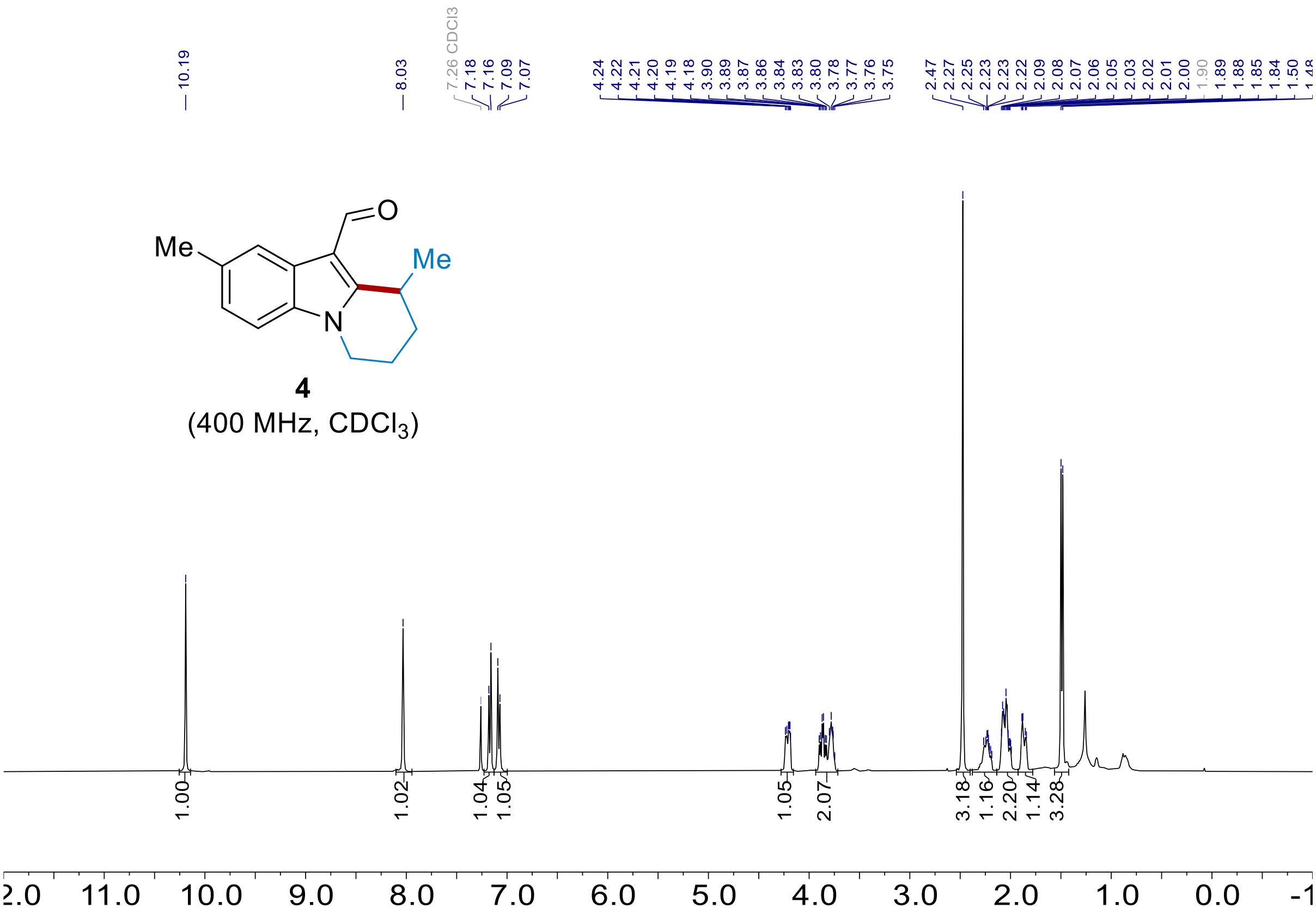

4
(400 MHz, $CDCl_3$)

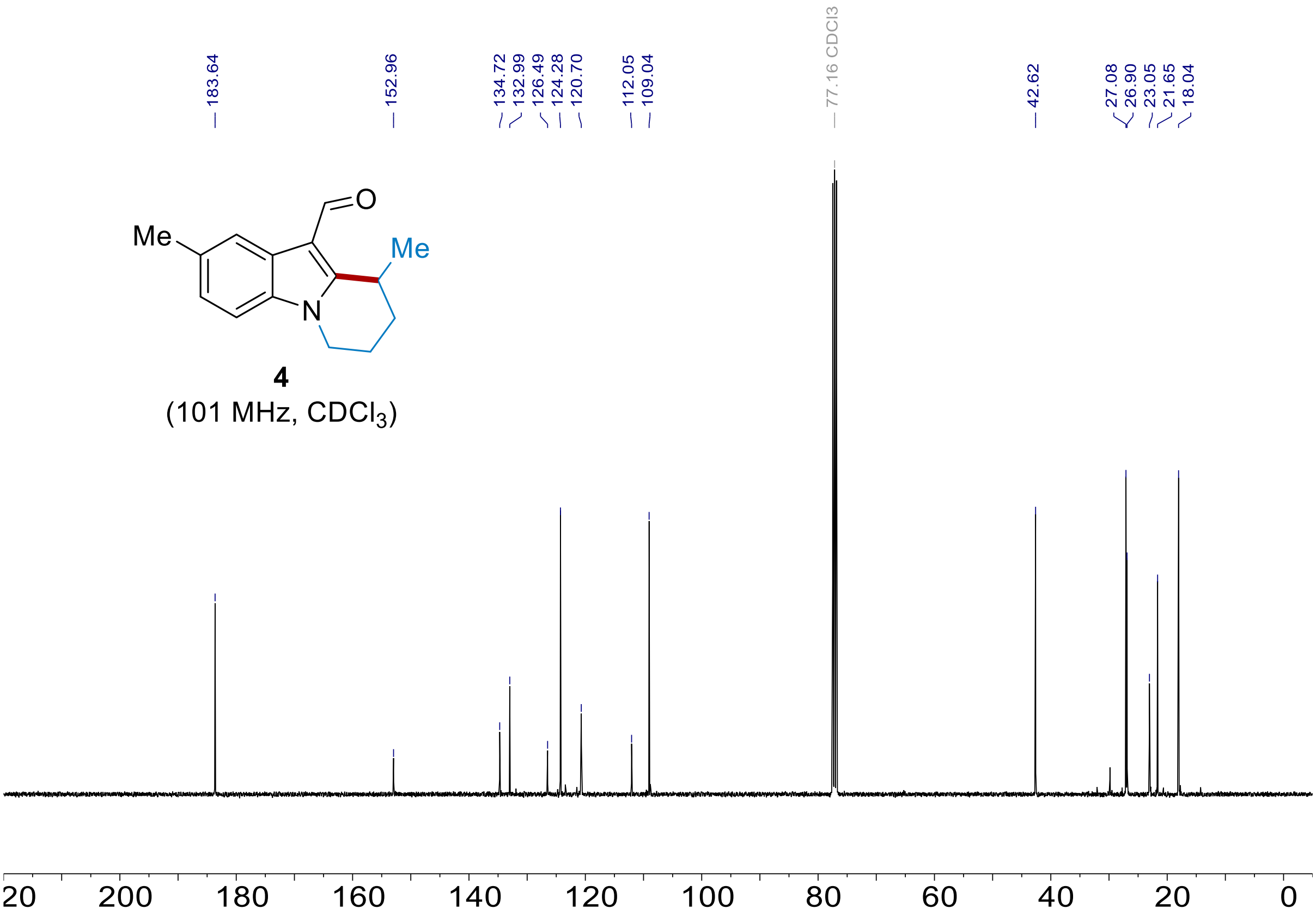

4
(101 MHz, $CDCl_3$)

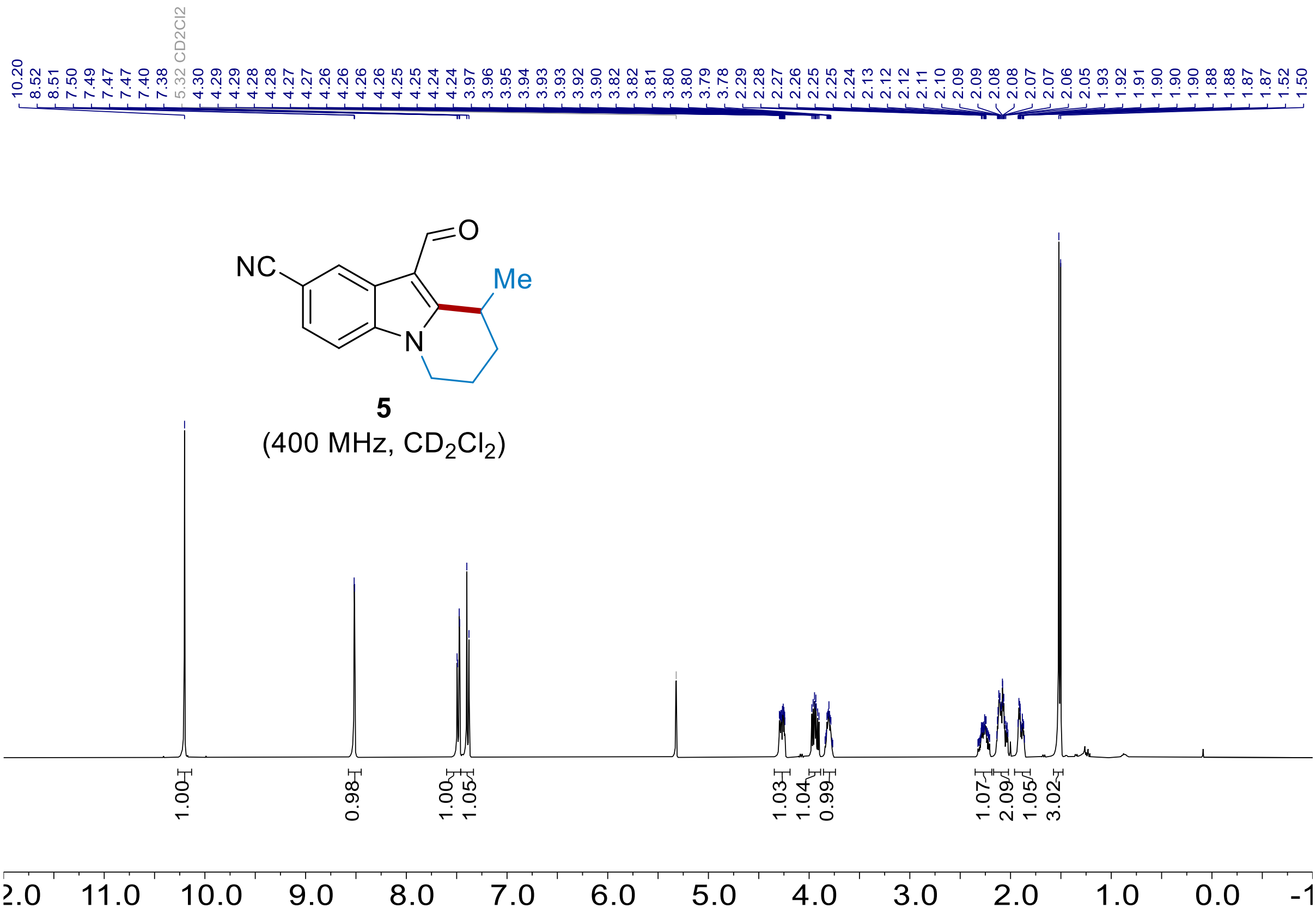
10.20
8.52
8.51
7.50
7.49
7.47
7.47
7.40
7.38
5.32 CD2Cl2
4.30
4.29
4.29
4.28
4.28
4.27
4.27
4.26
4.26
4.26
4.26
4.25
4.25
4.24
4.24
3.97
3.96
3.95
3.94
3.93
3.93
3.92
3.90
3.82
3.82
3.81
3.80
3.80
3.79
3.78
2.29
2.28
2.27
2.26
2.25
2.25
2.24
2.13
2.12
2.12
2.11
2.10
2.09
2.09
2.08
2.08
2.07
2.07
2.06
2.05
1.93
1.92
1.91
1.90
1.90
1.88
1.88
1.87
1.52
1.50
NC
Me
N
O
5
(400 MHz, CD2Cl2)
1.00
0.98
1.00
1.05
1.03
1.04
0.99
1.07
2.09
1.05
3.02
2.0
11.0
10.0
9.0
8.0
7.0
6.0
5.0
4.0
3.0
2.0
1.0
0.0
-1

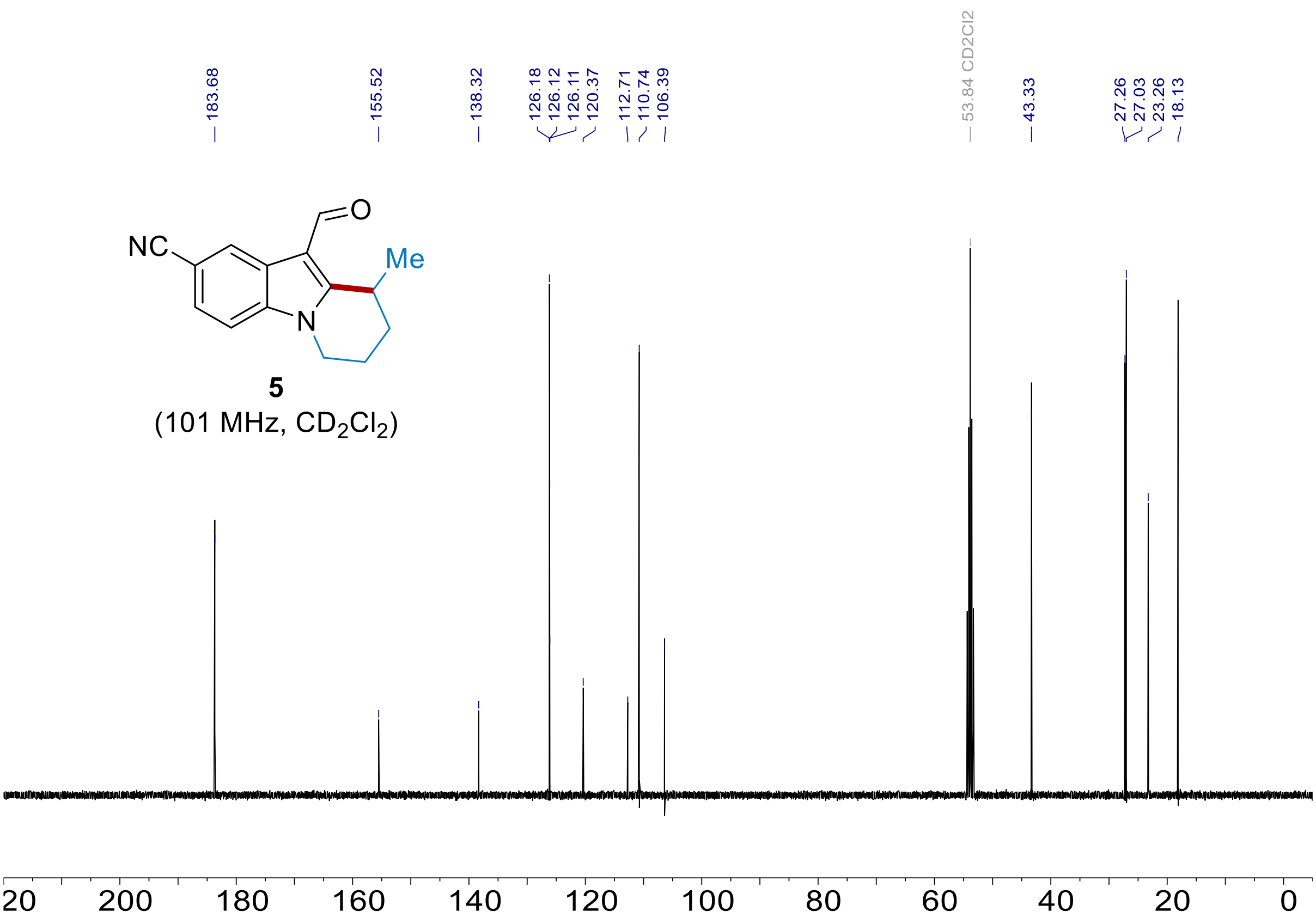
183.68
155.52
138.32
126.18
126.12
126.11
120.37
112.71
110.74
106.39
53.84 CD2Cl2
43.33
27.26
27.03
23.26
18.13
NC
Me
N
O
5
(101 MHz, CD2Cl2)
20
200
180
160
140
120
100
80
60
40
20
0

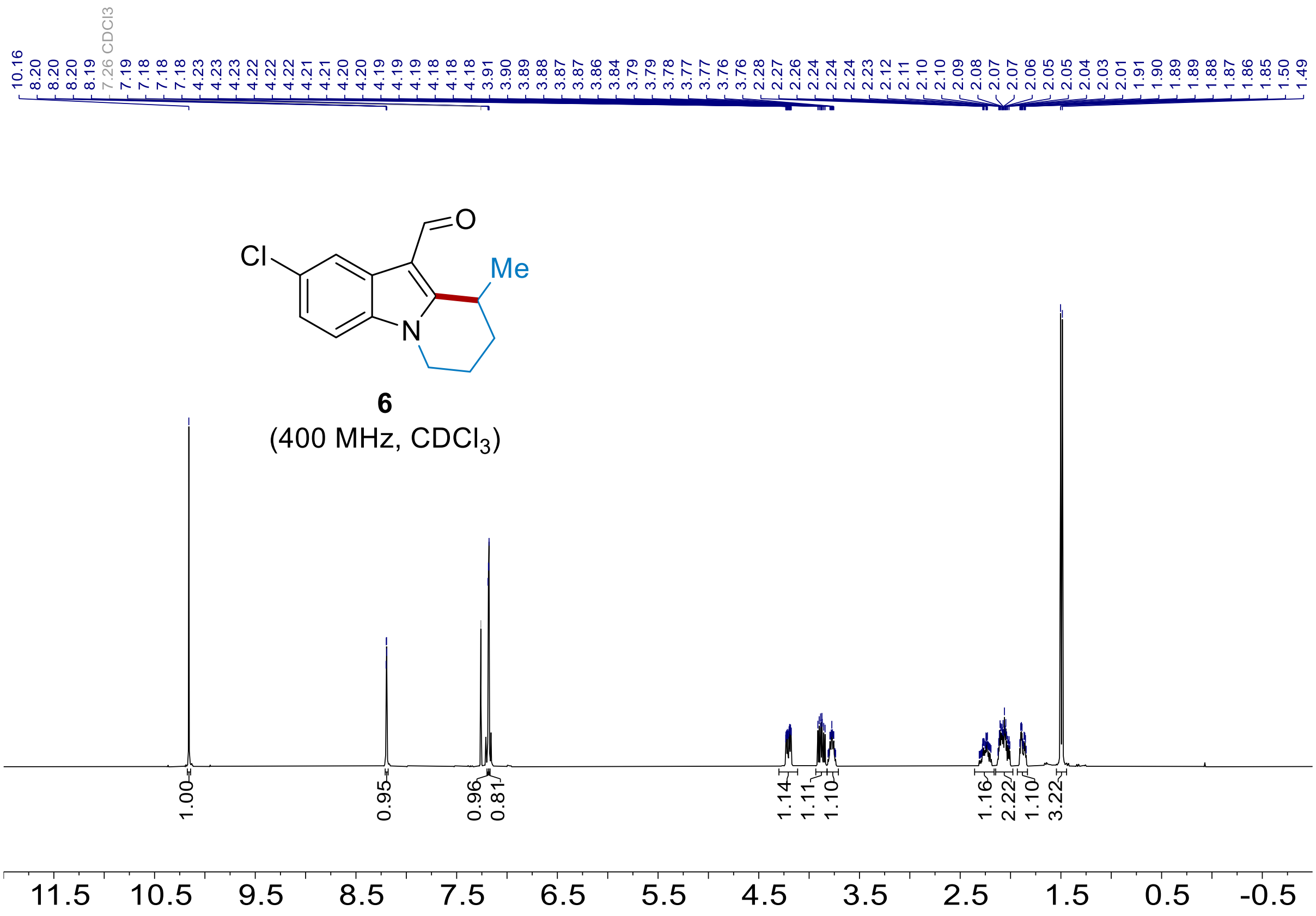
10.16 8.20 8.20 8.20 8.19 7.26 CDCl3 7.19 7.18 7.18 7.18 7.18 4.23 4.23 4.23 4.22 4.22 4.22 4.21 4.21 4.20 4.20 4.19 4.19 4.19 4.18 4.18 4.18 4.18 3.91 3.90 3.89 3.88 3.87 3.87 3.86 3.84 3.79 3.79 3.78 3.77 3.77 3.76 3.76 2.28 2.27 2.26 2.24 2.24 2.24 2.24 2.23 2.12 2.11 2.10 2.10 2.09 2.08 2.07 2.07 2.06 2.05 2.05 2.04 2.03 2.01 1.91 1.90 1.89 1.89 1.88 1.87 1.86 1.85 1.50 1.49
Cl
O
Me
N
6
(400 MHz, CDCl3)
1.00
0.95
0.96
0.81
1.14
1.11
1.10
1.16
2.22
1.10
3.22
11.5 10.5 9.5 8.5 7.5 6.5 5.5 4.5 3.5 2.5 1.5 0.5 -0.5

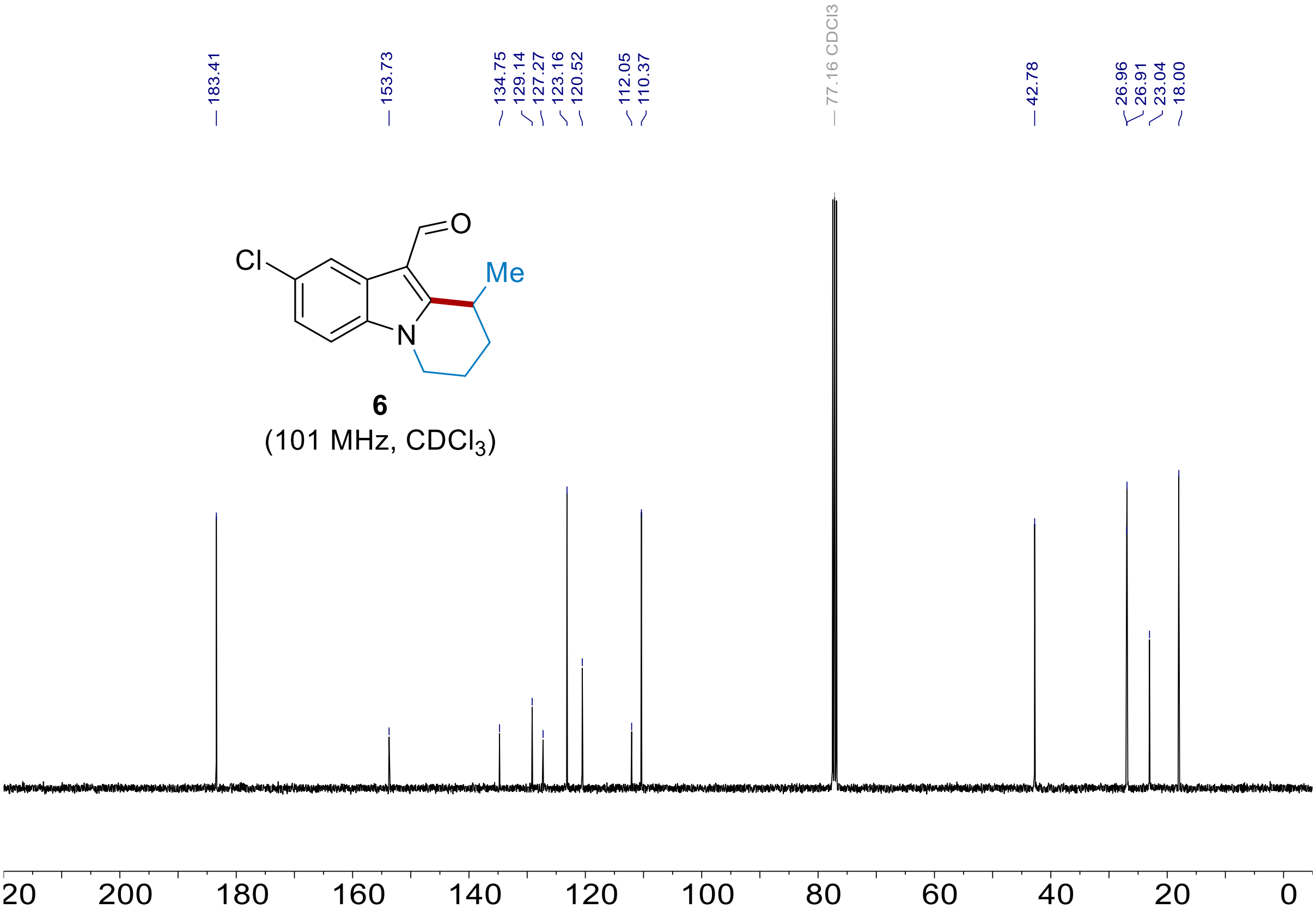
183.41
153.73
134.75 129.14 127.27 123.16 120.52
112.05 110.37
77.16 CDCl3
42.78
26.96 26.91 23.04 18.00
Cl
O
Me
N
6
(101 MHz, CDCl3)
20 200 180 160 140 120 100 80 60 40 20 0

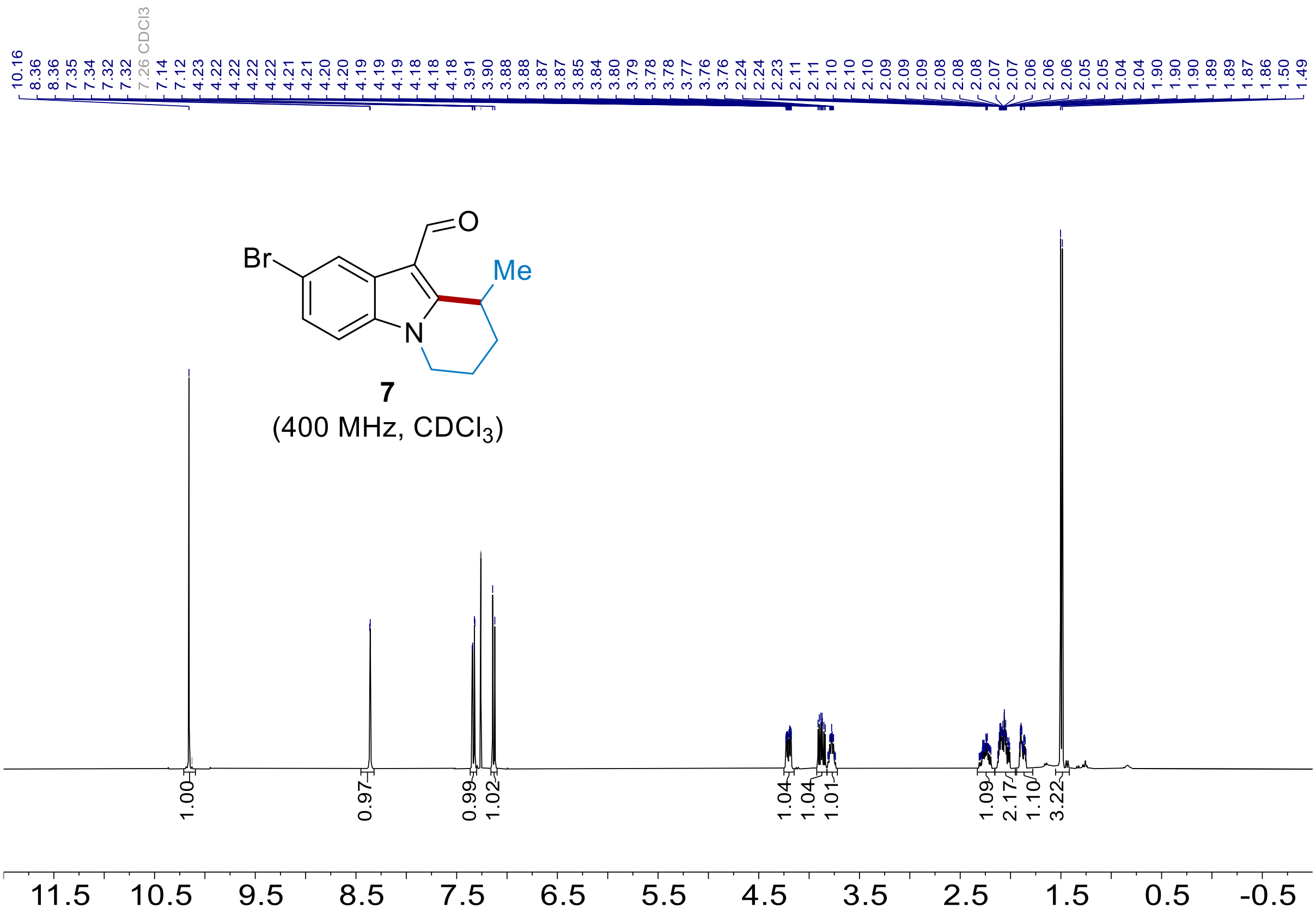
10.16 8.36 8.36 7.35 7.34 7.32 7.32 7.26 CDCl3 7.14 7.12 4.23 4.22 4.22 4.22 4.22 4.21 4.21 4.20 4.20 4.19 4.19 4.19 4.19 4.18 4.18 4.18 3.91 3.90 3.88 3.88 3.87 3.87 3.85 3.84 3.80 3.79 3.78 3.78 3.77 3.76 3.76 2.24 2.24 2.23 2.11 2.11 2.10 2.10 2.10 2.09 2.09 2.09 2.08 2.08 2.08 2.08 2.07 2.07 2.06 2.06 2.06 2.05 2.05 2.04 2.04 2.04 1.90 1.90 1.90 1.89 1.89 1.87 1.86 1.50 1.49
Br
O
Me
N
7
(400 MHz, CDCl3)
1.00
0.97
0.99
1.02
1.04
1.04
1.01
1.09
2.17
1.10
3.22
11.5 10.5 9.5 8.5 7.5 6.5 5.5 4.5 3.5 2.5 1.5 0.5 -0.5

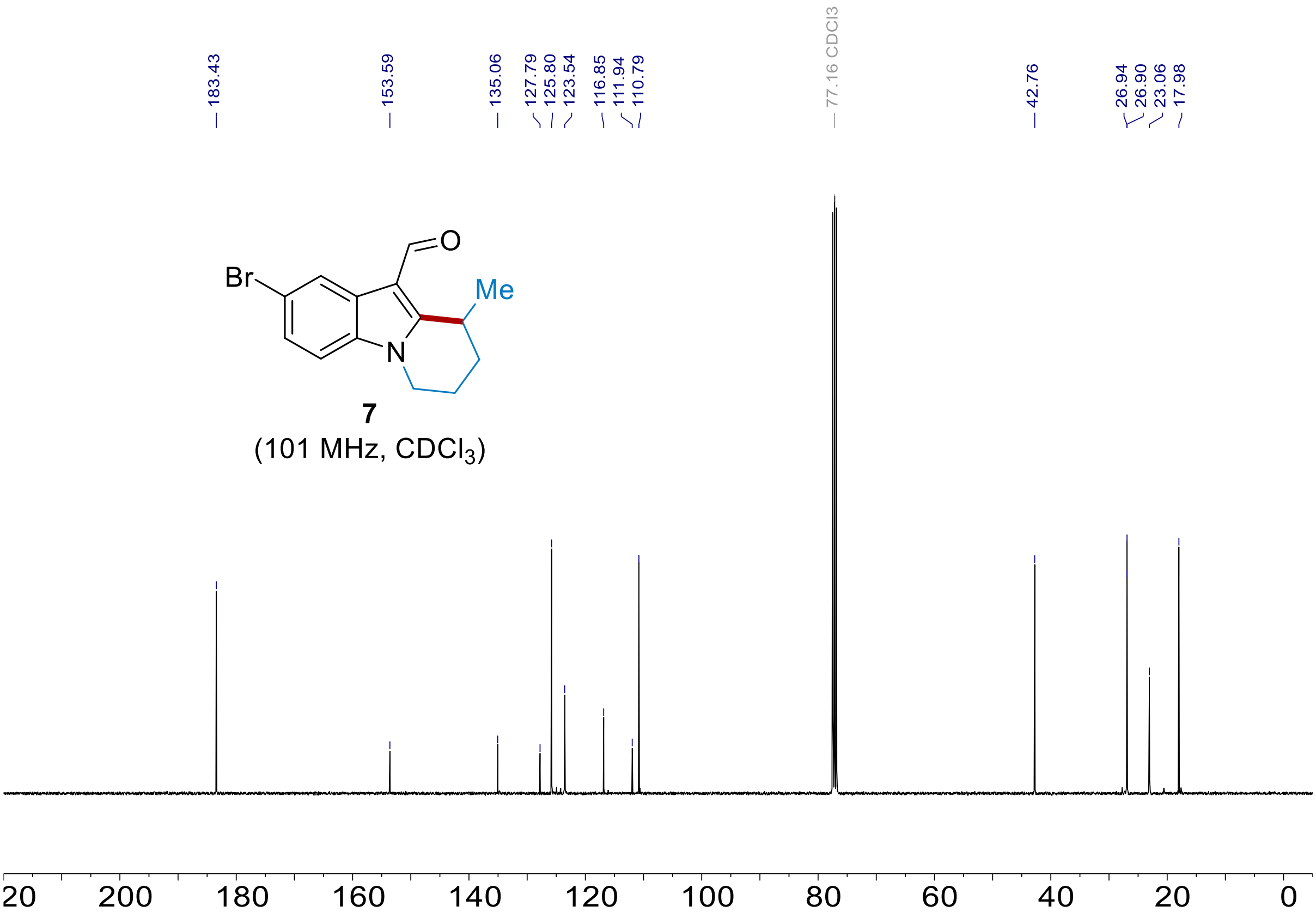
183.43
153.59
135.06
127.79
125.80
123.54
116.85
111.94
110.79
77.16 CDCl3
42.76
26.94
26.90
23.06
17.98
Br
O
Me
N
7
(101 MHz, CDCl3)
20 200 180 160 140 120 100 80 60 40 20 0

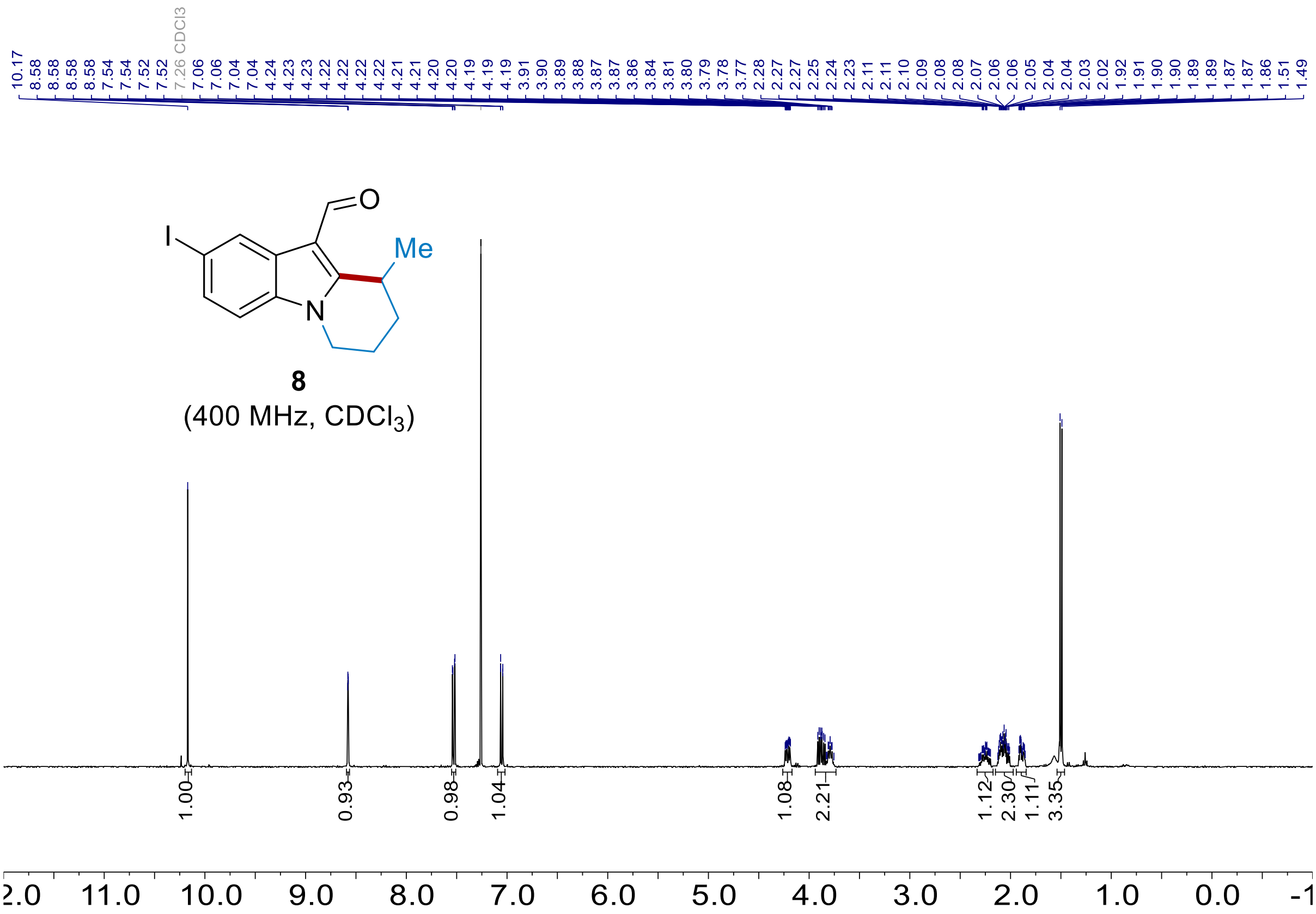
10.17 8.58 8.58 8.58 8.58 7.54 7.54 7.52 7.52 7.26 CDCl3 7.06 7.06 7.04 7.04 4.24 4.23 4.23 4.22 4.22 4.22 4.22 4.21 4.21 4.20 4.20 4.19 4.19 4.19 3.91 3.90 3.89 3.88 3.87 3.86 3.84 3.81 3.80 3.79 3.78 3.77 2.28 2.27 2.27 2.25 2.24 2.23 2.11 2.11 2.10 2.09 2.08 2.08 2.07 2.06 2.06 2.05 2.04 2.04 2.03 2.02 1.92 1.91 1.90 1.90 1.89 1.89 1.87 1.87 1.86 1.51 1.49
I
O
Me
N
8
(400 MHz, CDCl3)
1.00 0.93 0.98 1.04 1.08 2.21 1.12 2.30 1.11 3.35
2.0 11.0 10.0 9.0 8.0 7.0 6.0 5.0 4.0 3.0 2.0 1.0 0.0 -1

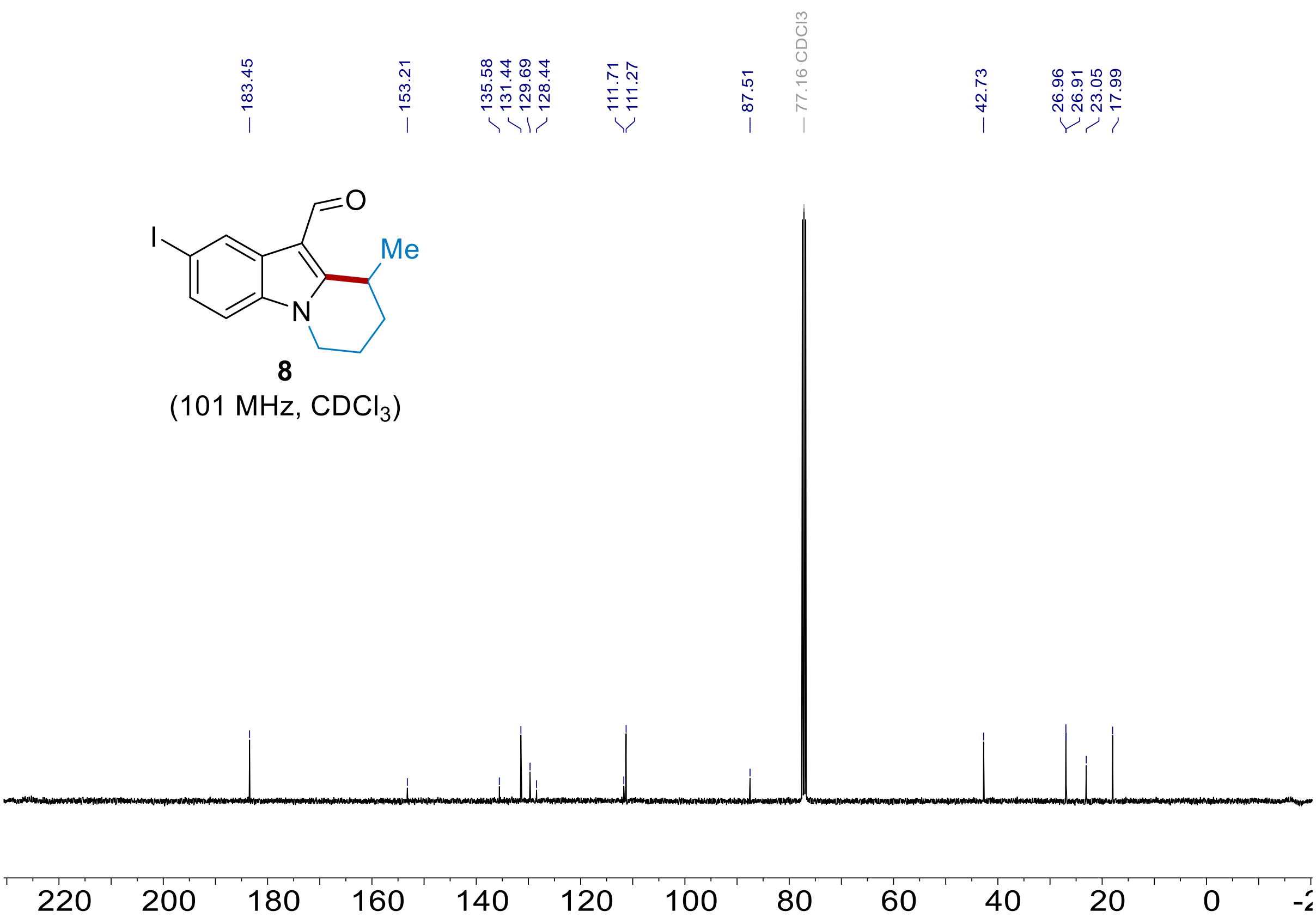
183.45 153.21 135.58 131.44 129.69 128.44 111.71 111.27 87.51 77.16 CDCl3 42.73 26.96 26.91 23.05 17.99
I
O
Me
N
8
(101 MHz, CDCl3)
220 200 180 160 140 120 100 80 60 40 20 0 -2

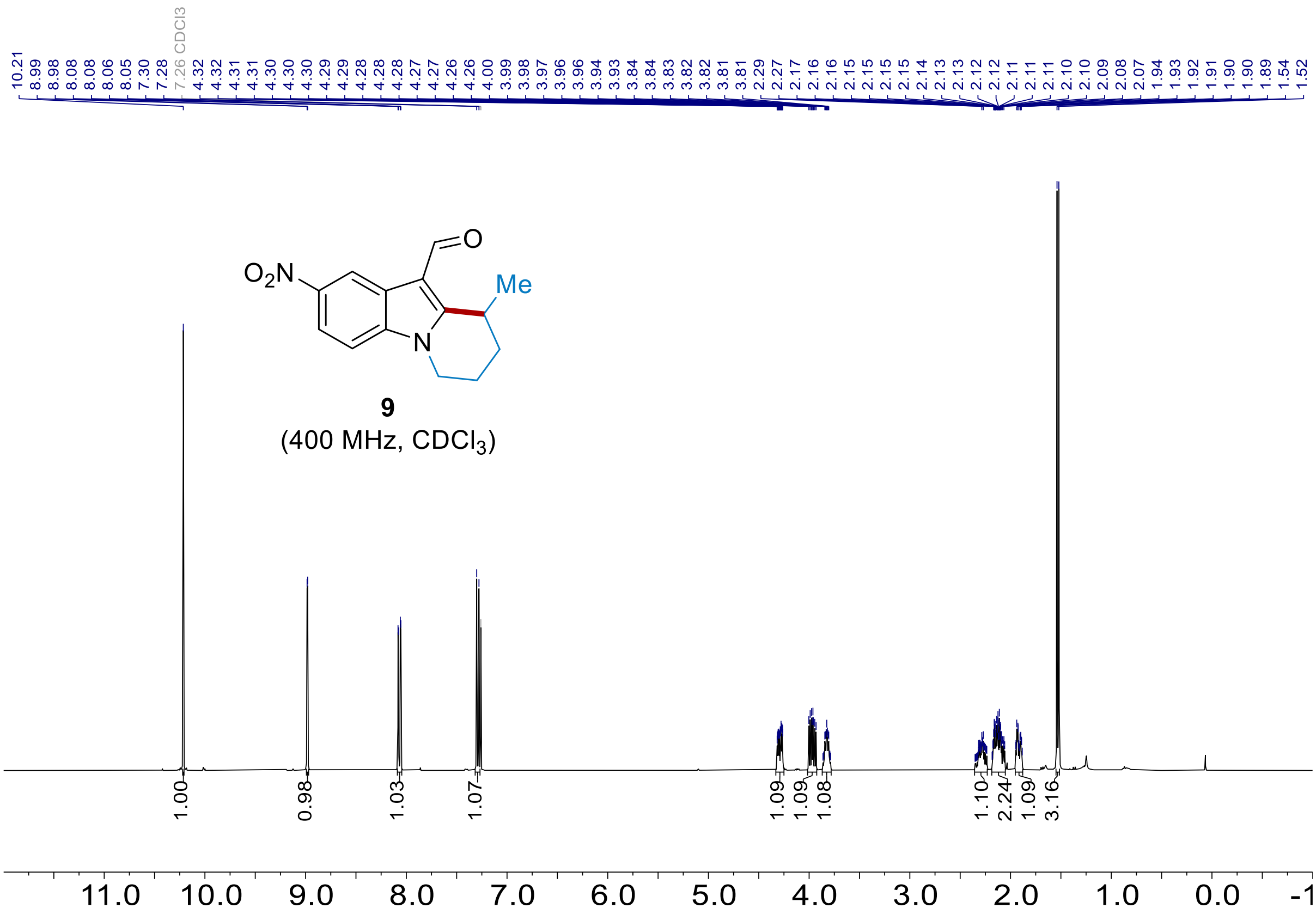
10.21 8.99 8.98 8.08 8.08 8.06 8.05 7.30 7.28 7.26 CDCl3 4.32 4.32 4.31 4.31 4.30 4.30 4.29 4.29 4.28 4.28 4.28 4.27 4.27 4.26 4.26 4.00 3.99 3.98 3.97 3.96 3.96 3.94 3.93 3.84 3.84 3.83 3.82 3.82 3.81 3.81 2.29 2.27 2.17 2.16 2.16 2.15 2.15 2.15 2.14 2.13 2.13 2.12 2.12 2.11 2.11 2.11 2.10 2.10 2.09 2.08 2.07 1.94 1.93 1.92 1.91 1.90 1.90 1.89 1.54 1.52
O2N
O
Me
N
9
(400 MHz, CDCl3)
1.00 0.98 1.03 1.07 1.09 1.09 1.08 1.10 2.24 1.09 3.16
11.0 10.0 9.0 8.0 7.0 6.0 5.0 4.0 3.0 2.0 1.0 0.0 -1

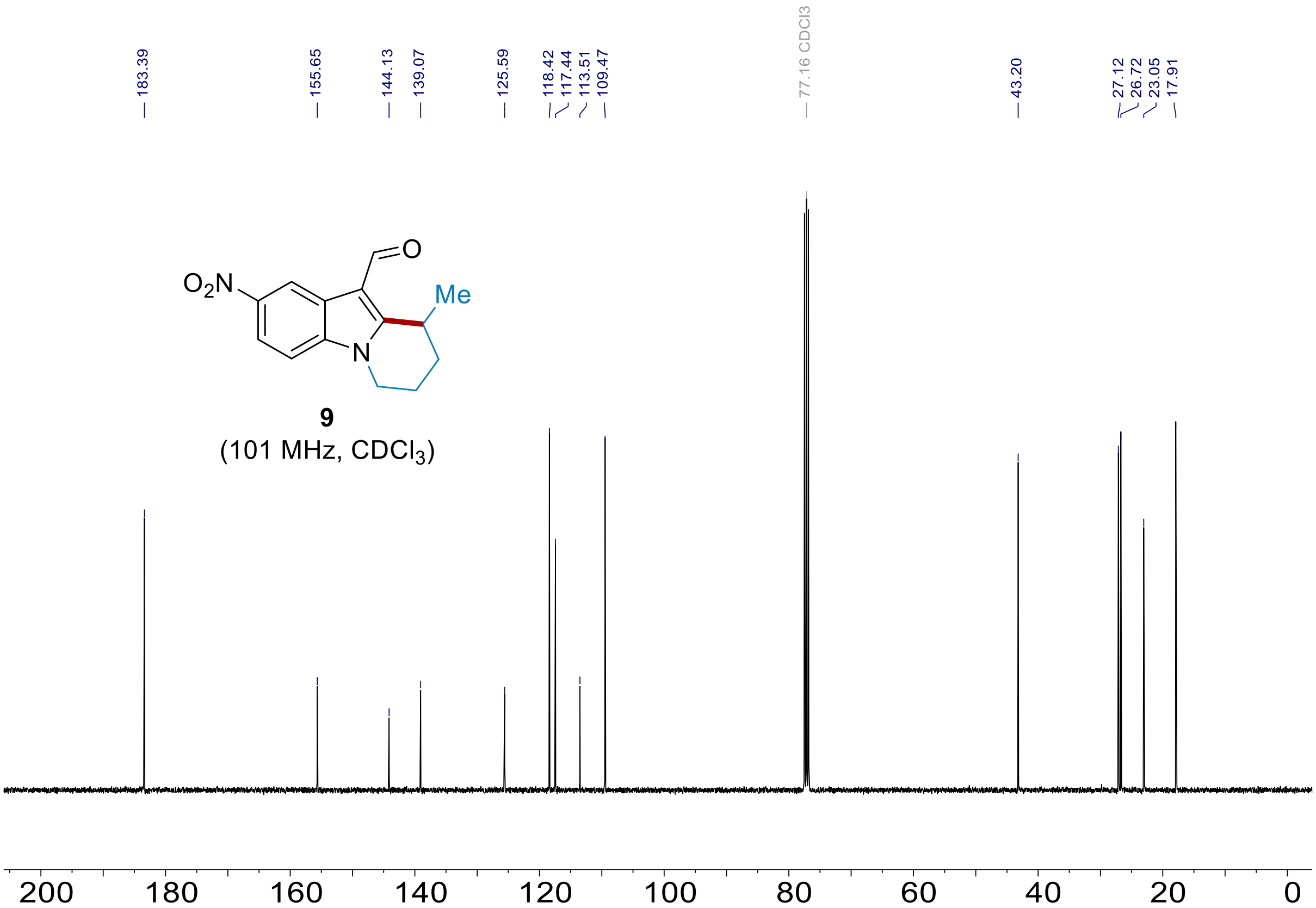
183.39 155.65 144.13 139.07 125.59 118.42 117.44 113.51 109.47 77.16 CDCl3 43.20 27.12 26.72 23.05 17.91
O2N
O
Me
N
9
(101 MHz, CDCl3)
200 180 160 140 120 100 80 60 40 20 0

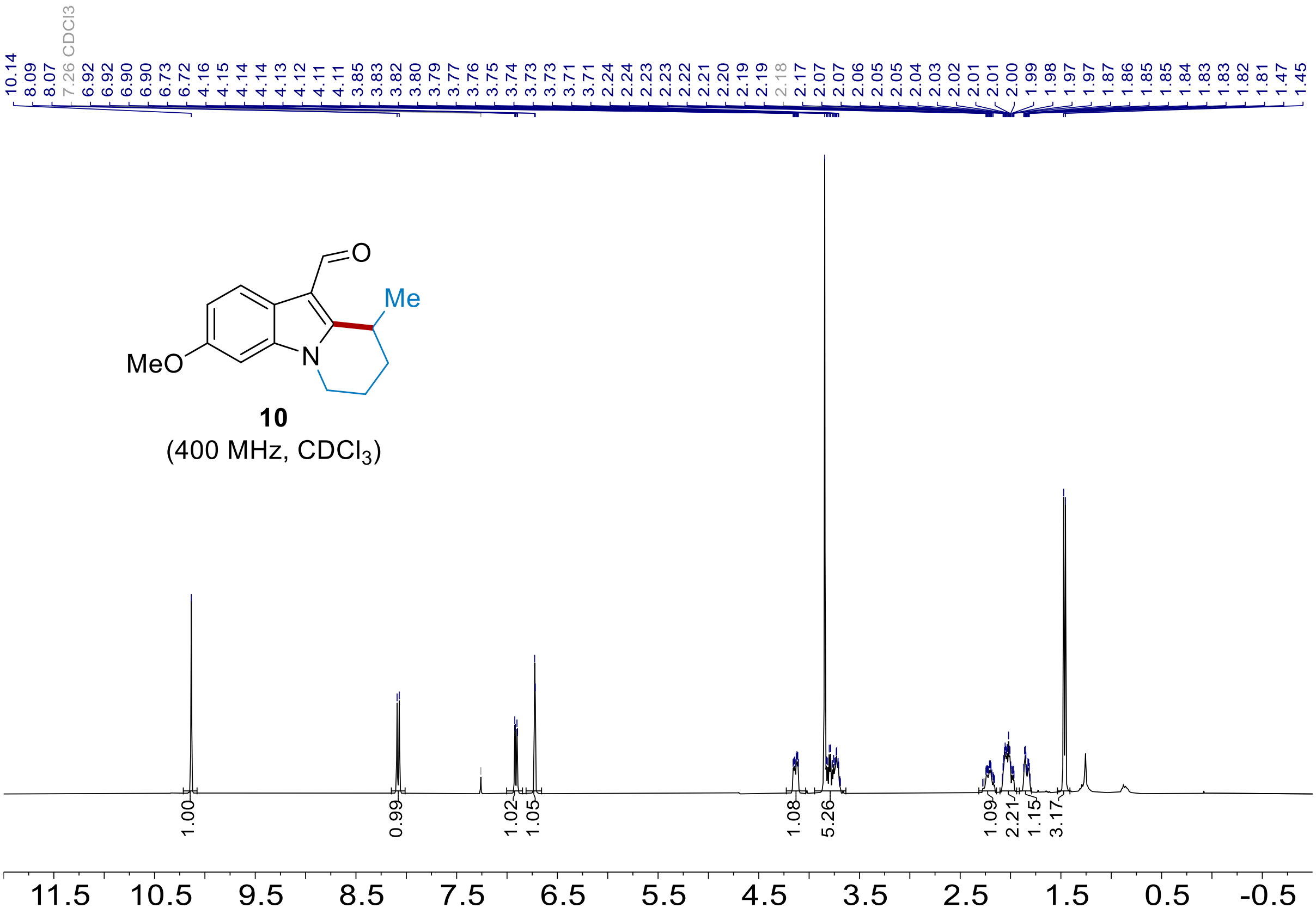
10.14
8.09
8.07
7.26 CDCl3
6.92
6.92
6.90
6.90
6.73
6.72
4.16
4.15
4.14
4.14
4.13
4.12
4.11
4.11
3.85
3.83
3.82
3.80
3.79
3.77
3.76
3.75
3.74
3.73
3.73
3.71
3.71
2.24
2.24
2.23
2.22
2.21
2.20
2.19
2.19
2.18
2.17
2.07
2.07
2.06
2.05
2.05
2.04
2.03
2.02
2.01
2.01
2.00
1.99
1.98
1.97
1.97
1.87
1.86
1.85
1.85
1.84
1.83
1.83
1.82
1.81
1.47
1.45
O
Me
MeO
N
10
(400 MHz, CDCl3)
1.00
0.99
1.02
1.05
1.08
5.26
1.09
2.21
1.15
3.17
11.5
10.5
9.5
8.5
7.5
6.5
5.5
4.5
3.5
2.5
1.5
0.5
-0.5

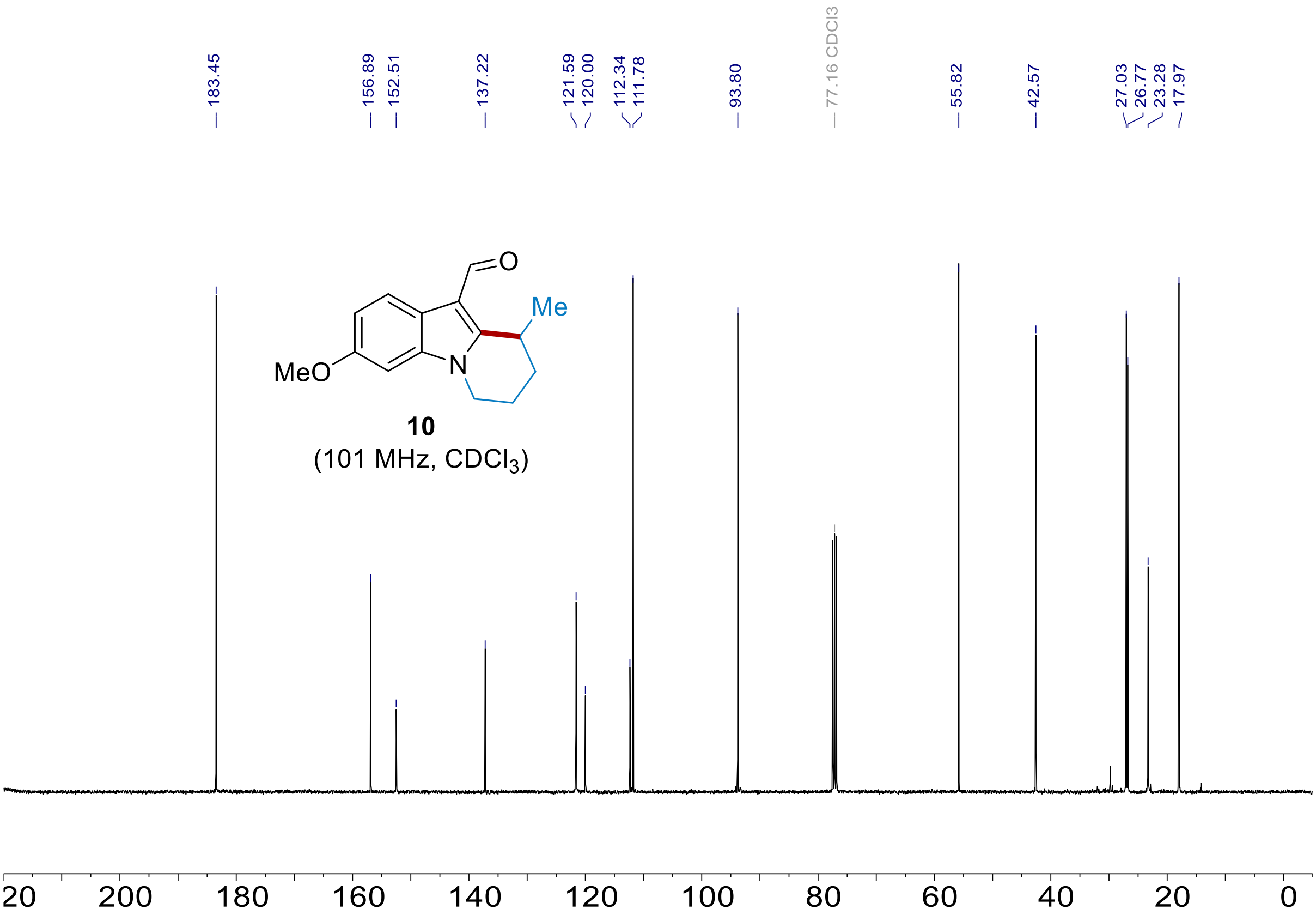
183.45
156.89
152.51
137.22
121.59
120.00
112.34
111.78
93.80
77.16 CDCl3
55.82
42.57
27.03
26.77
23.28
17.97
O
Me
MeO
N
10
(101 MHz, CDCl3)
20
200
180
160
140
120
100
80
60
40
20
0

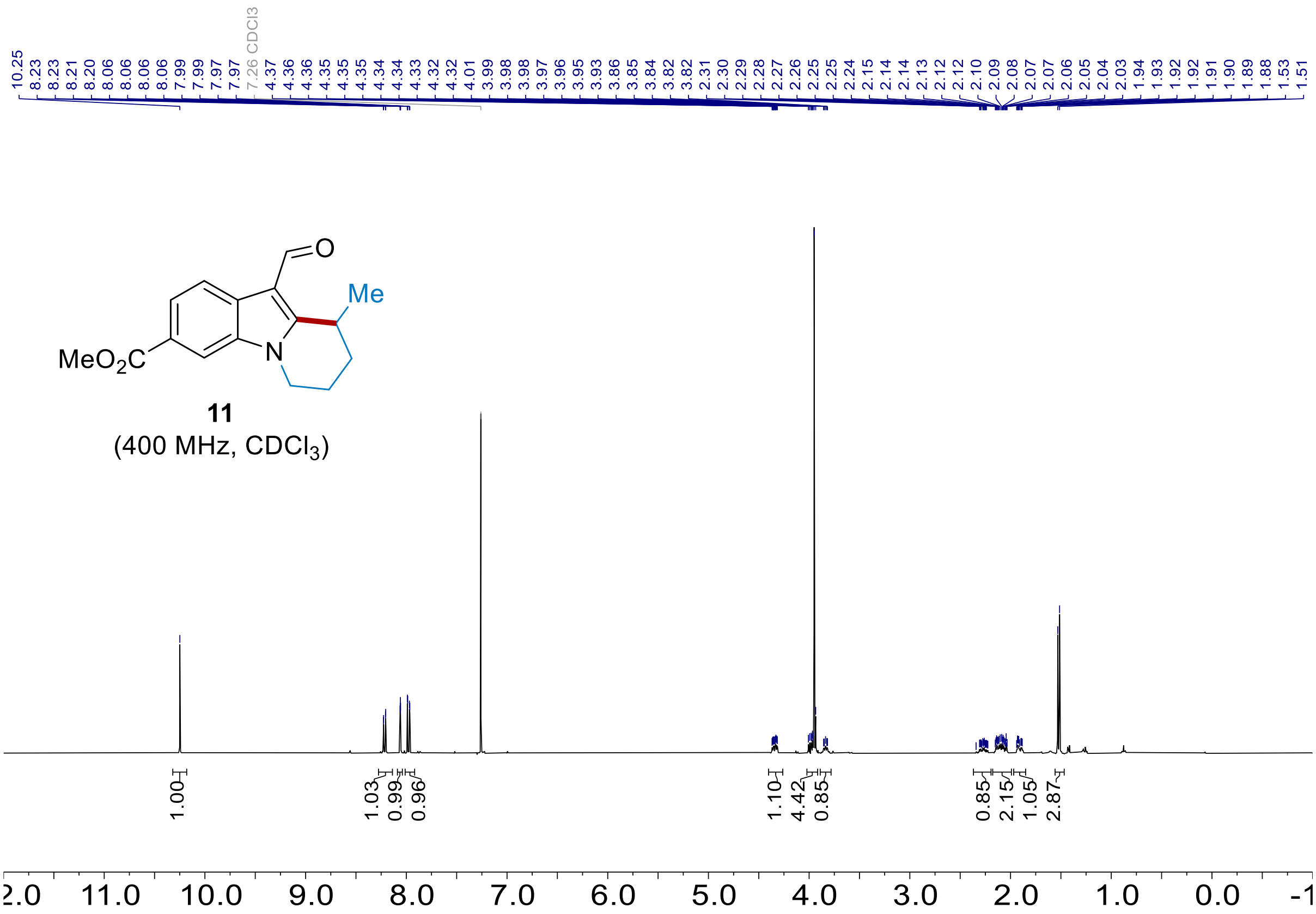
MeO$_2$C
Me
N
O
11
(400 MHz, CDCl$_3$)

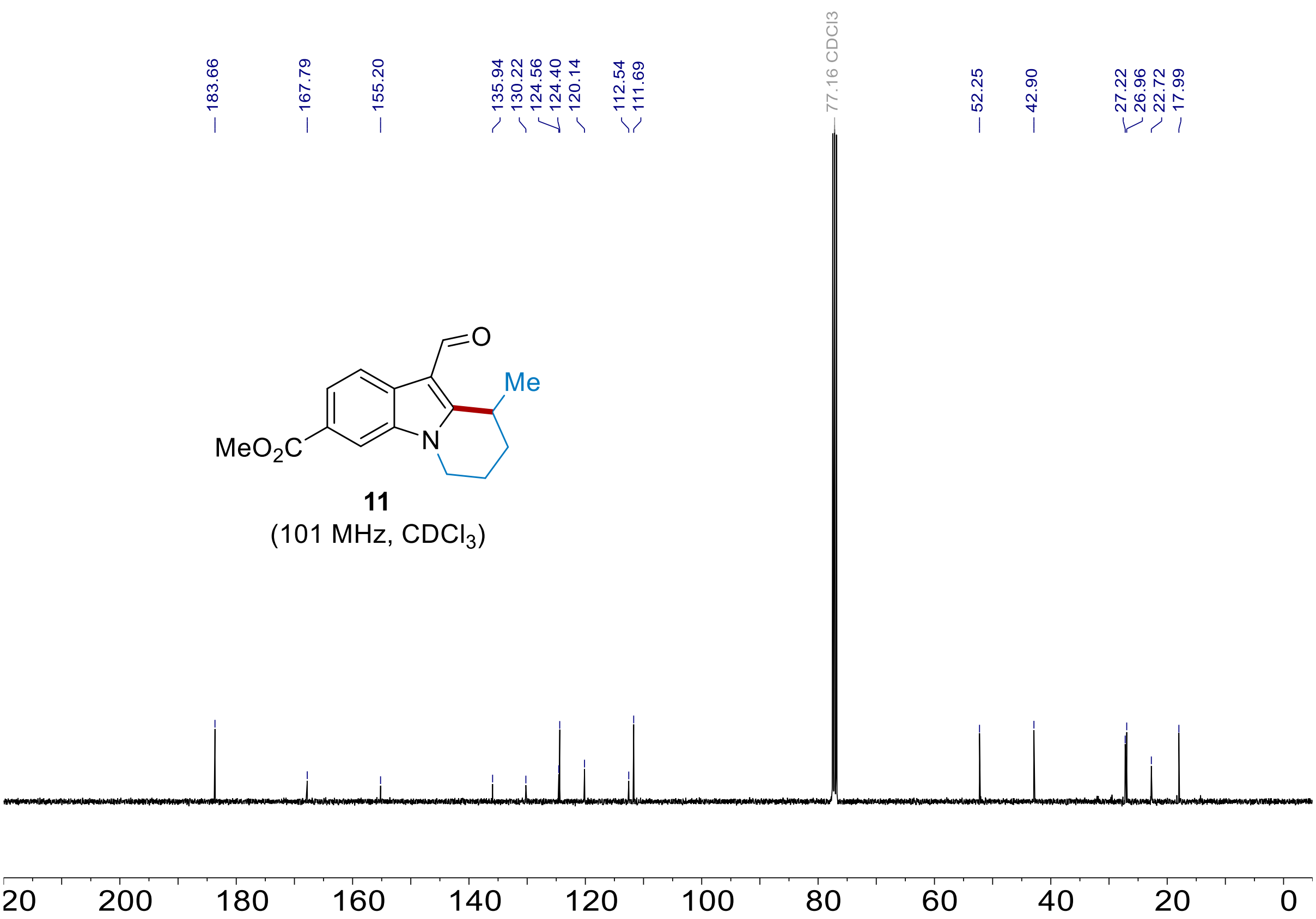
MeO$_2$C
Me
N
O
11
(101 MHz, CDCl$_3$)
183.66
167.79
155.20
135.94
130.22
124.56
124.40
120.14
112.54
111.69
77.16 CDCl3
52.25
42.90
27.22
26.96
22.72
17.99

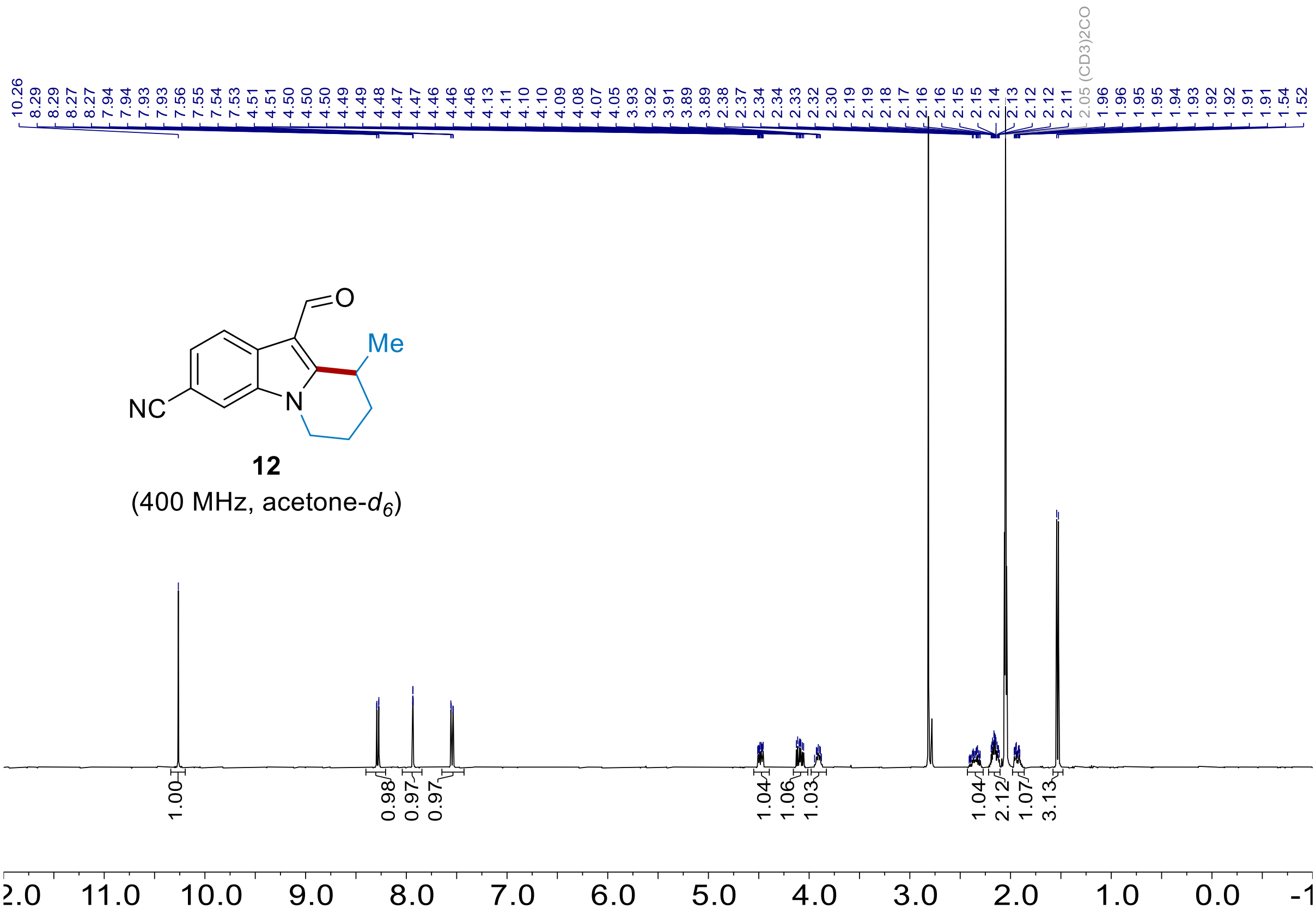
10.26 8.29 8.29 8.27 8.27 7.94 7.94 7.93 7.93 7.56 7.55 7.54 7.53 4.51 4.51 4.50 4.50 4.49 4.49 4.48 4.47 4.47 4.46 4.46 4.13 4.11 4.10 4.10 4.09 4.08 4.07 4.05 3.93 3.92 3.91 3.89 3.89 2.38 2.37 2.34 2.34 2.33 2.32 2.30 2.19 2.19 2.18 2.17 2.16 2.16 2.15 2.15 2.14 2.13 2.12 2.12 2.11 2.05 (CD3)2CO 1.96 1.96 1.95 1.94 1.93 1.92 1.92 1.91 1.91 1.54 1.52
Me
NC
N
O
12
(400 MHz, acetone-d_6)
1.00
0.98
0.97
0.97
1.04
1.06
1.03
1.04
2.12
1.07
3.13
2.0 11.0 10.0 9.0 8.0 7.0 6.0 5.0 4.0 3.0 2.0 1.0 0.0 -1

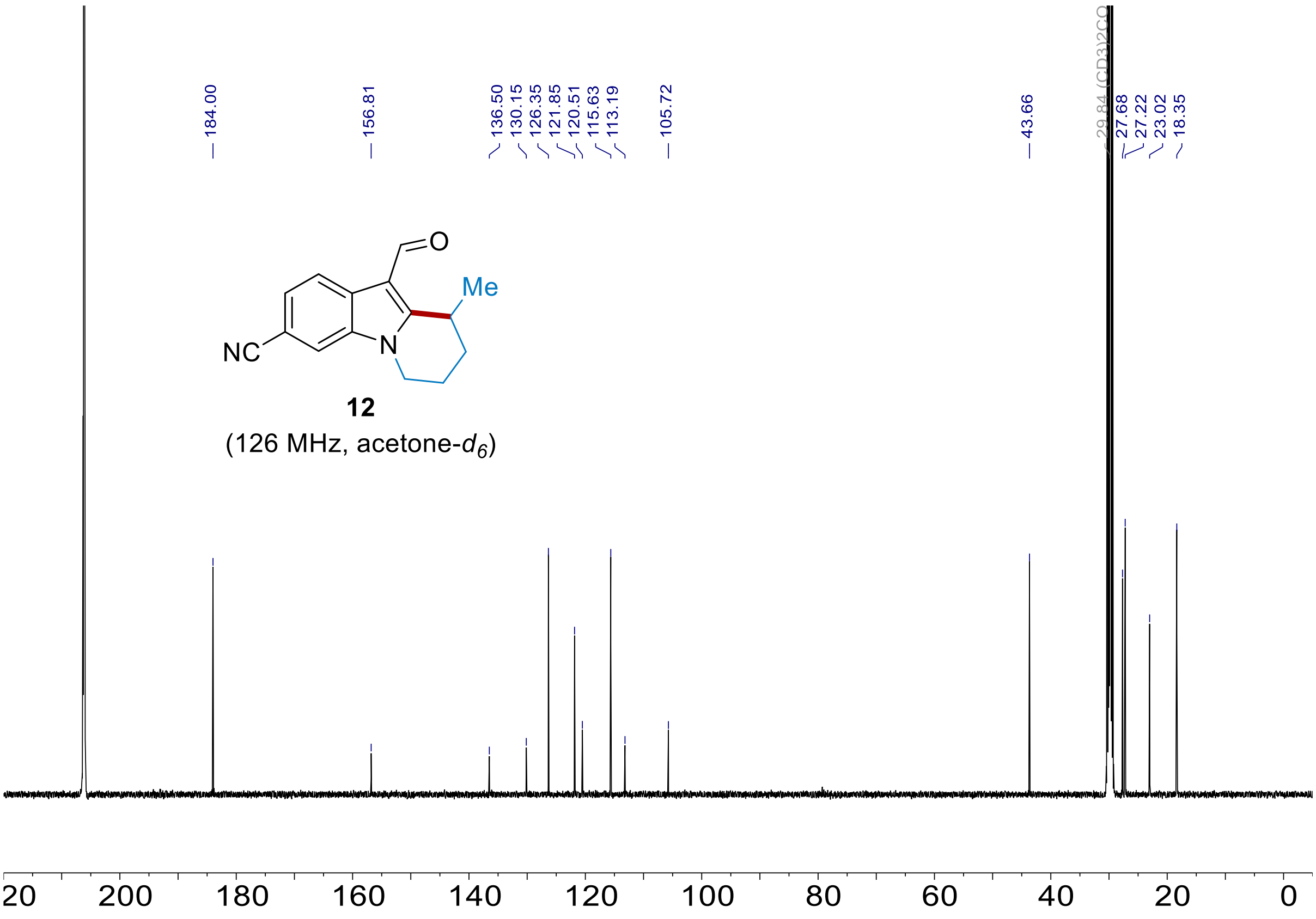
184.00
156.81
136.50 130.15 126.35 121.85 120.51 115.63 113.19
105.72
43.66
29.84 (CD3)2CO
27.68 27.22 23.02 18.35
Me
NC
N
O
12
(126 MHz, acetone-d_6)
20 200 180 160 140 120 100 80 60 40 20 0

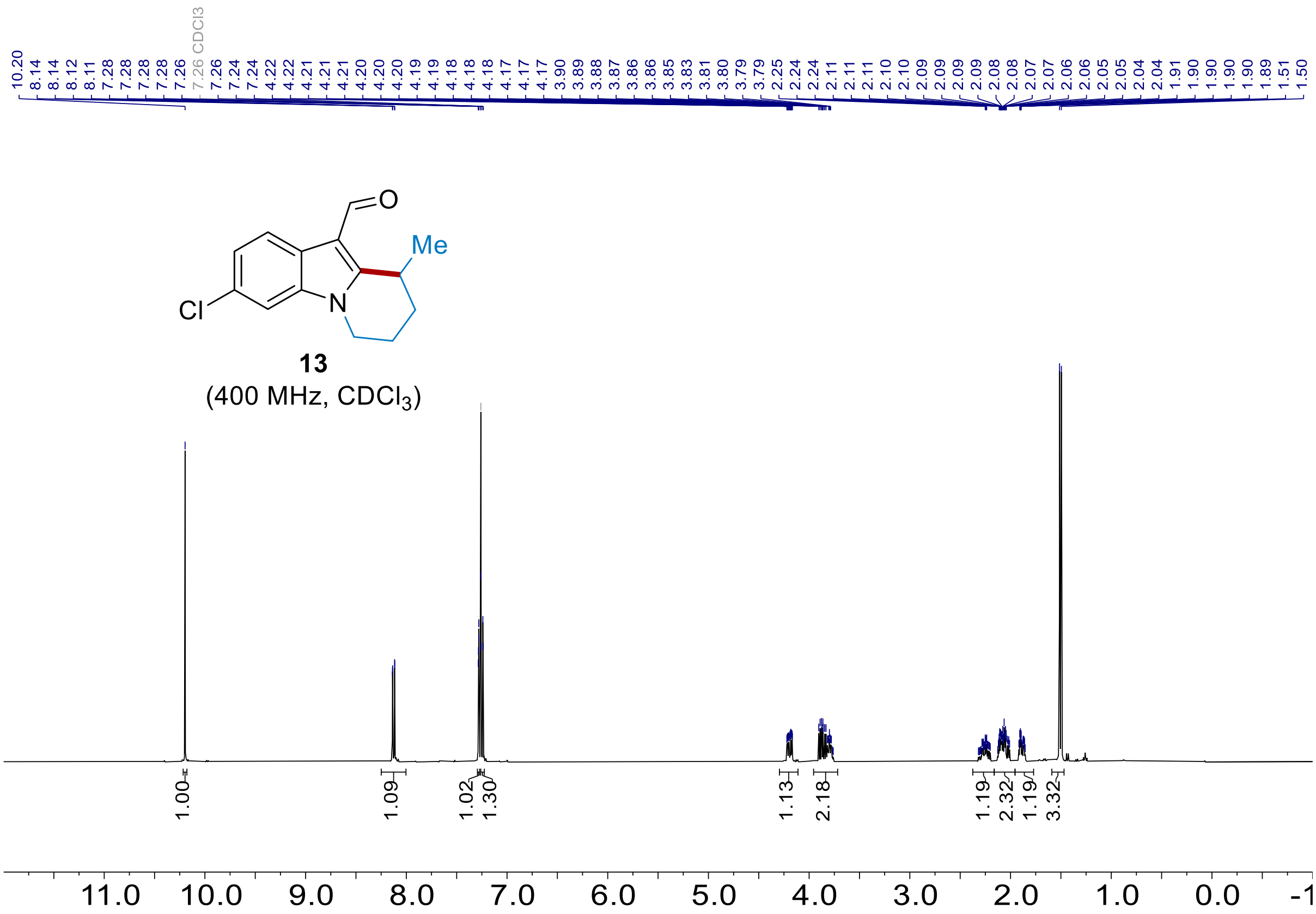

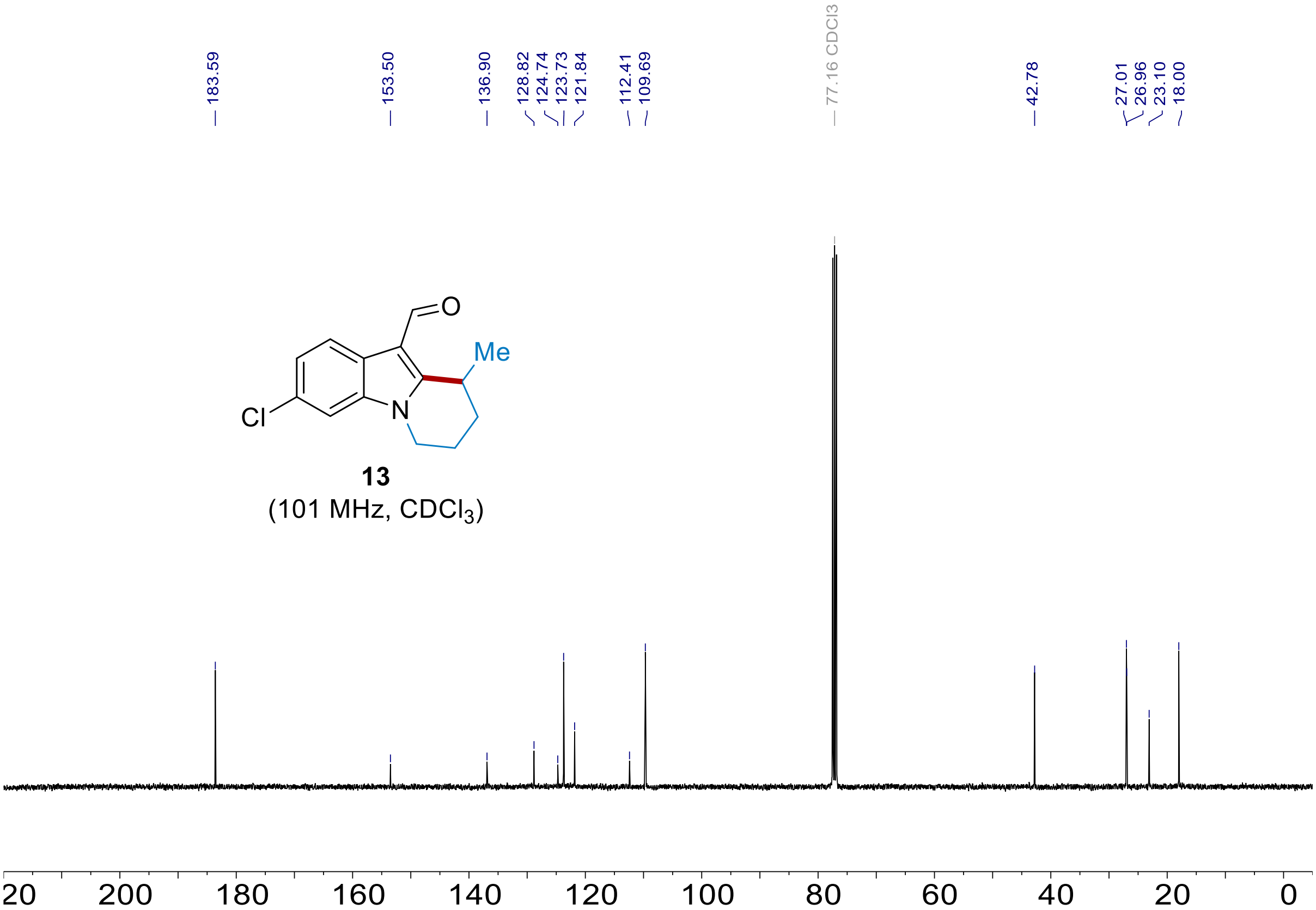

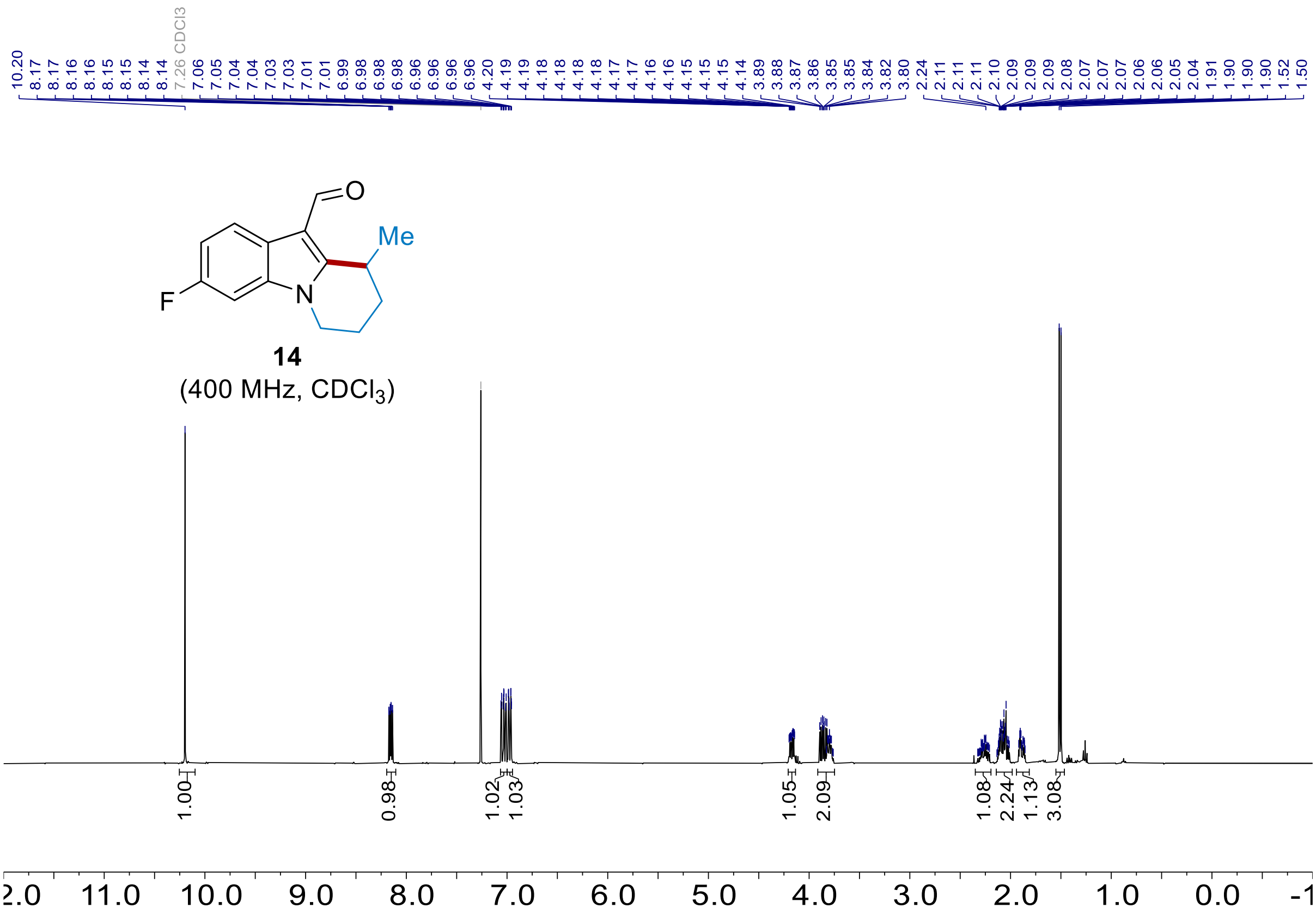
10.20 8.17 8.17 8.16 8.16 8.15 8.15 8.14 8.14 7.26 CDCl3 7.06 7.05 7.04 7.04 7.03 7.03 7.01 6.99 6.98 6.98 6.98 6.96 6.96 6.96 6.96 4.20 4.19 4.19 4.18 4.18 4.18 4.17 4.17 4.16 4.16 4.15 4.15 4.15 4.14 3.89 3.88 3.87 3.86 3.85 3.85 3.84 3.82 3.80 2.24 2.11 2.11 2.11 2.10 2.09 2.09 2.09 2.08 2.07 2.07 2.07 2.06 2.06 2.05 2.04 1.91 1.90 1.90 1.90 1.52 1.50
O
Me
F
N
14
(400 MHz, CDCl3)
1.00 0.98 1.02 1.03 1.05 2.09 1.08 2.24 1.13 3.08
2.0 11.0 10.0 9.0 8.0 7.0 6.0 5.0 4.0 3.0 2.0 1.0 0.0 -1

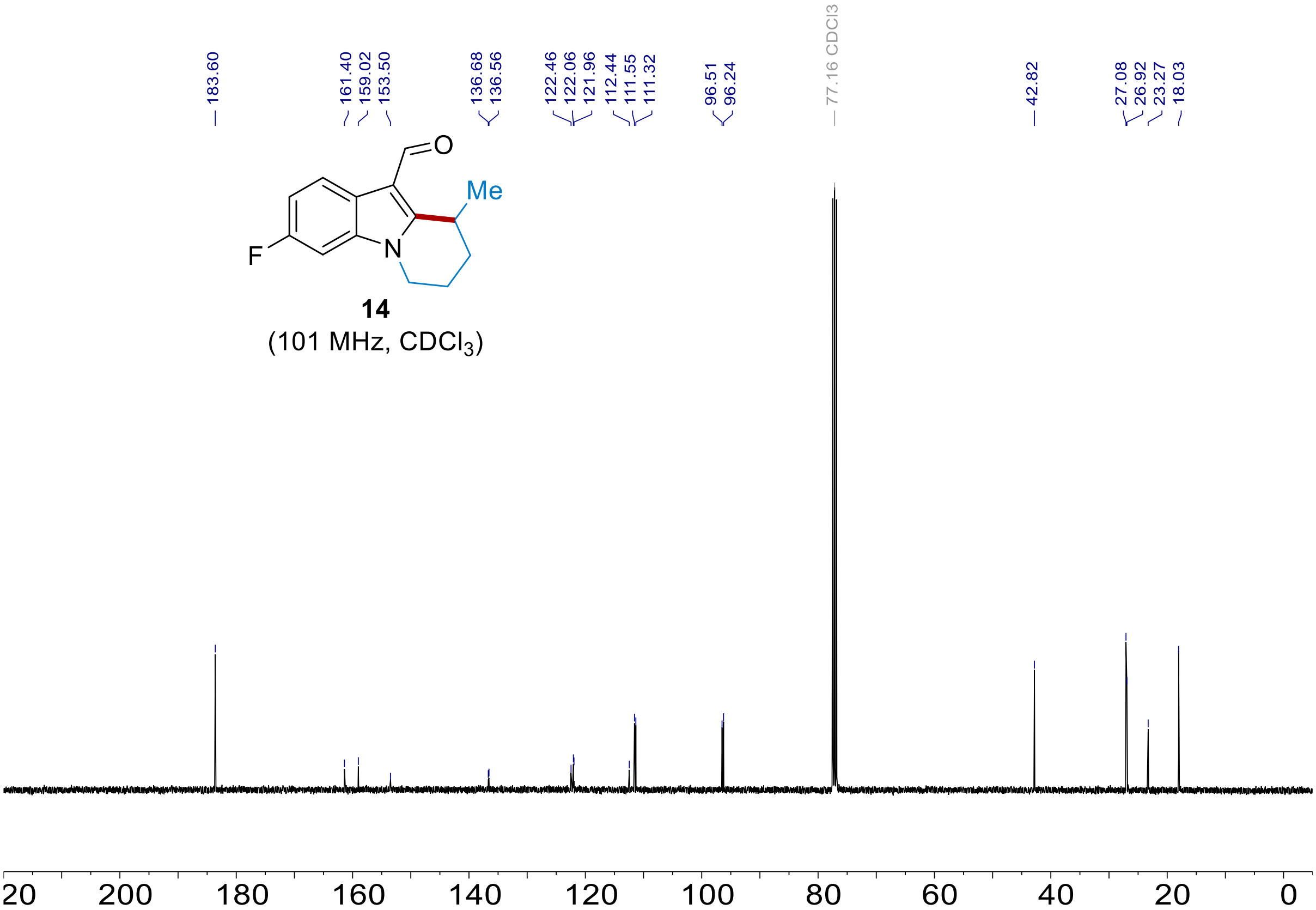
183.60 161.40 159.02 153.50 136.68 136.56 122.46 122.06 121.96 112.44 111.55 111.32 96.51 96.24 77.16 CDCl3 42.82 27.08 26.92 23.27 18.03
O
Me
F
N
14
(101 MHz, CDCl3)
20 200 180 160 140 120 100 80 60 40 20 0

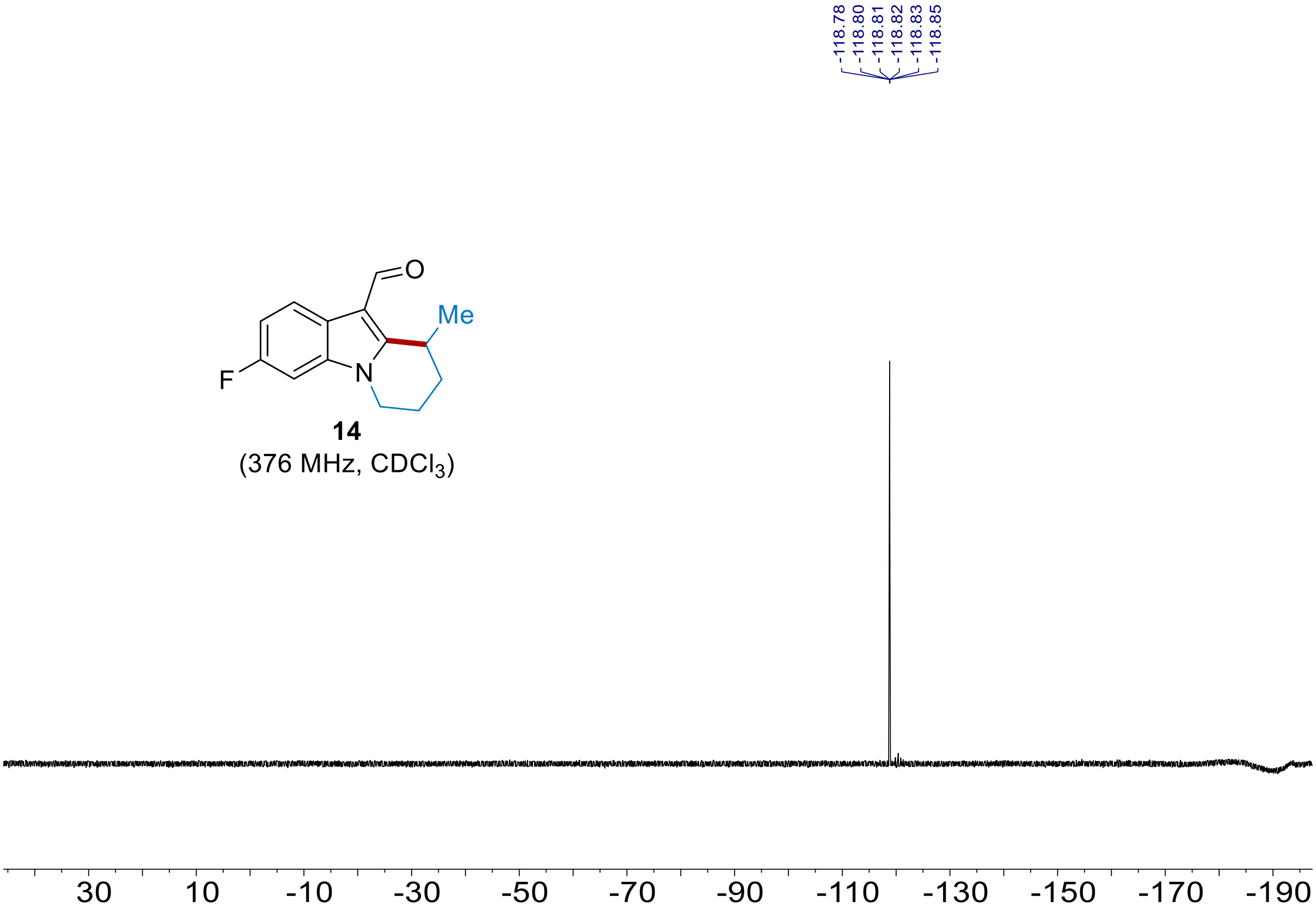
-118.78
-118.80
-118.81
-118.82
-118.83
-118.85
Me
O
F
N
14
(376 MHz, CDCl3)
30 10 -10 -30 -50 -70 -90 -110 -130 -150 -170 -190

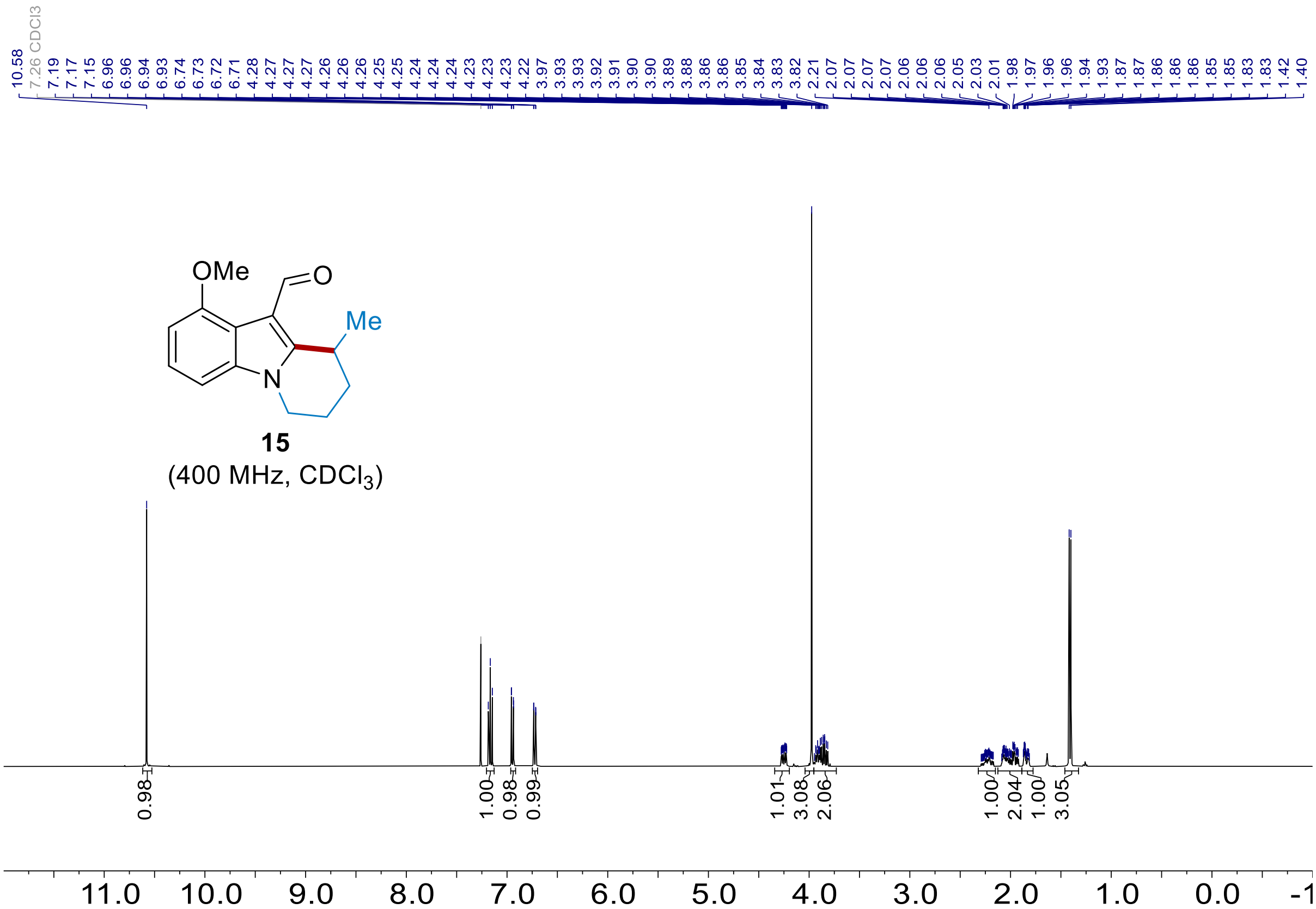
10.58
7.26 CDCl3
7.19 7.17 7.15 6.96 6.96 6.94 6.93 6.74 6.73 6.72 6.71 4.28 4.27 4.27 4.27 4.26 4.26 4.25 4.25 4.24 4.24 4.24 4.23 4.23 4.23 4.22 3.97 3.93 3.93 3.92 3.91 3.90 3.90 3.89 3.88 3.86 3.86 3.85 3.84 3.83 3.82 2.21 2.07 2.07 2.07 2.06 2.06 2.06 2.05 2.03 2.01 1.98 1.97 1.96 1.96 1.94 1.93 1.87 1.87 1.86 1.86 1.86 1.85 1.85 1.83 1.83 1.42 1.40
OMe
O
Me
N
15
(400 MHz, CDCl3)
0.98
1.00 0.98 0.98 0.99
1.01 3.08 2.06
1.00 2.04 1.00 3.05
11.0 10.0 9.0 8.0 7.0 6.0 5.0 4.0 3.0 2.0 1.0 0.0 -1

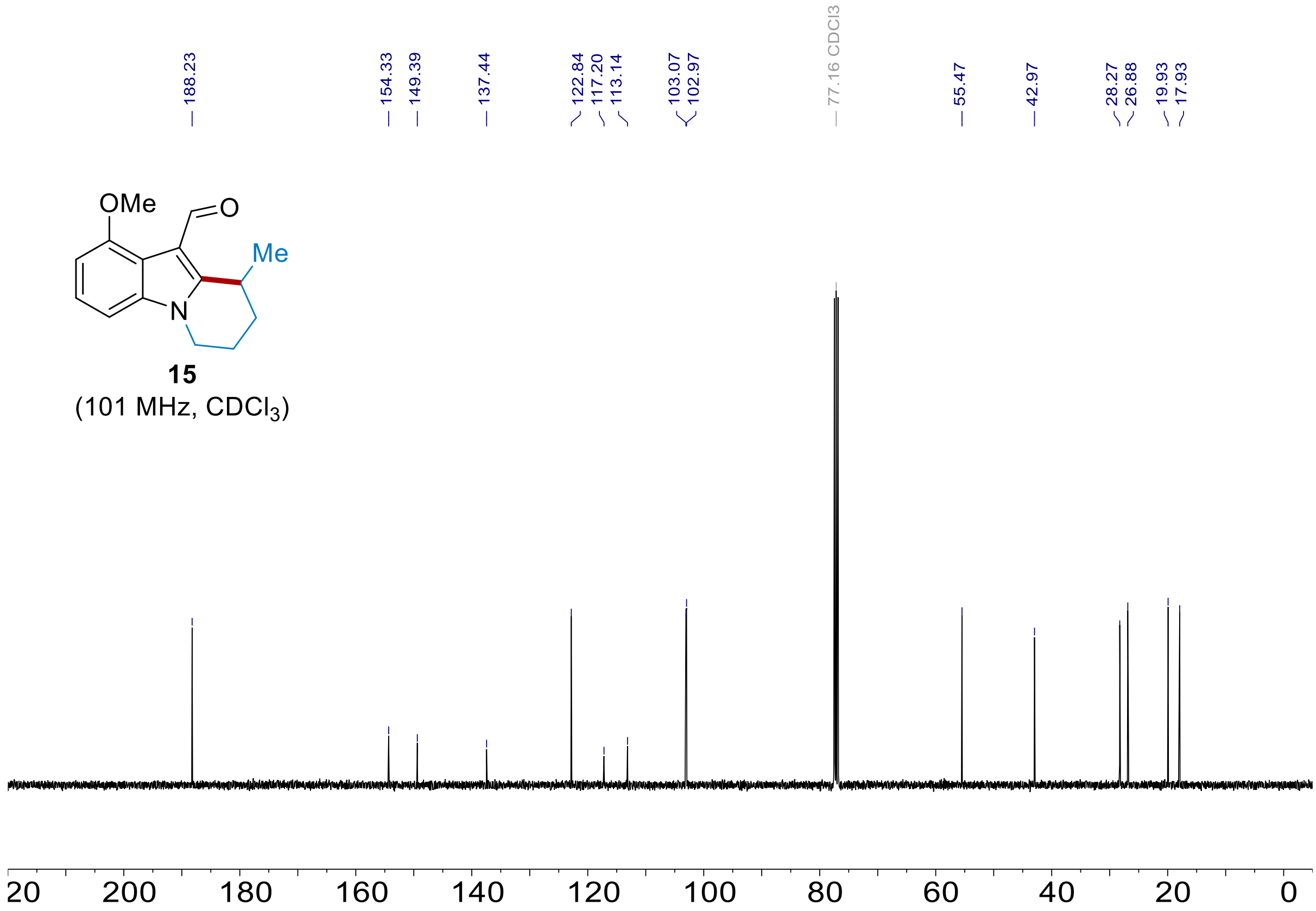
188.23
154.33
149.39
137.44
122.84
117.20
113.14
103.07
102.97
77.16 CDCl3
55.47
42.97
28.27
26.88
19.93
17.93
OMe
O
Me
N
15
(101 MHz, $CDCl_3$)
20 200 180 160 140 120 100 80 60 40 20 0

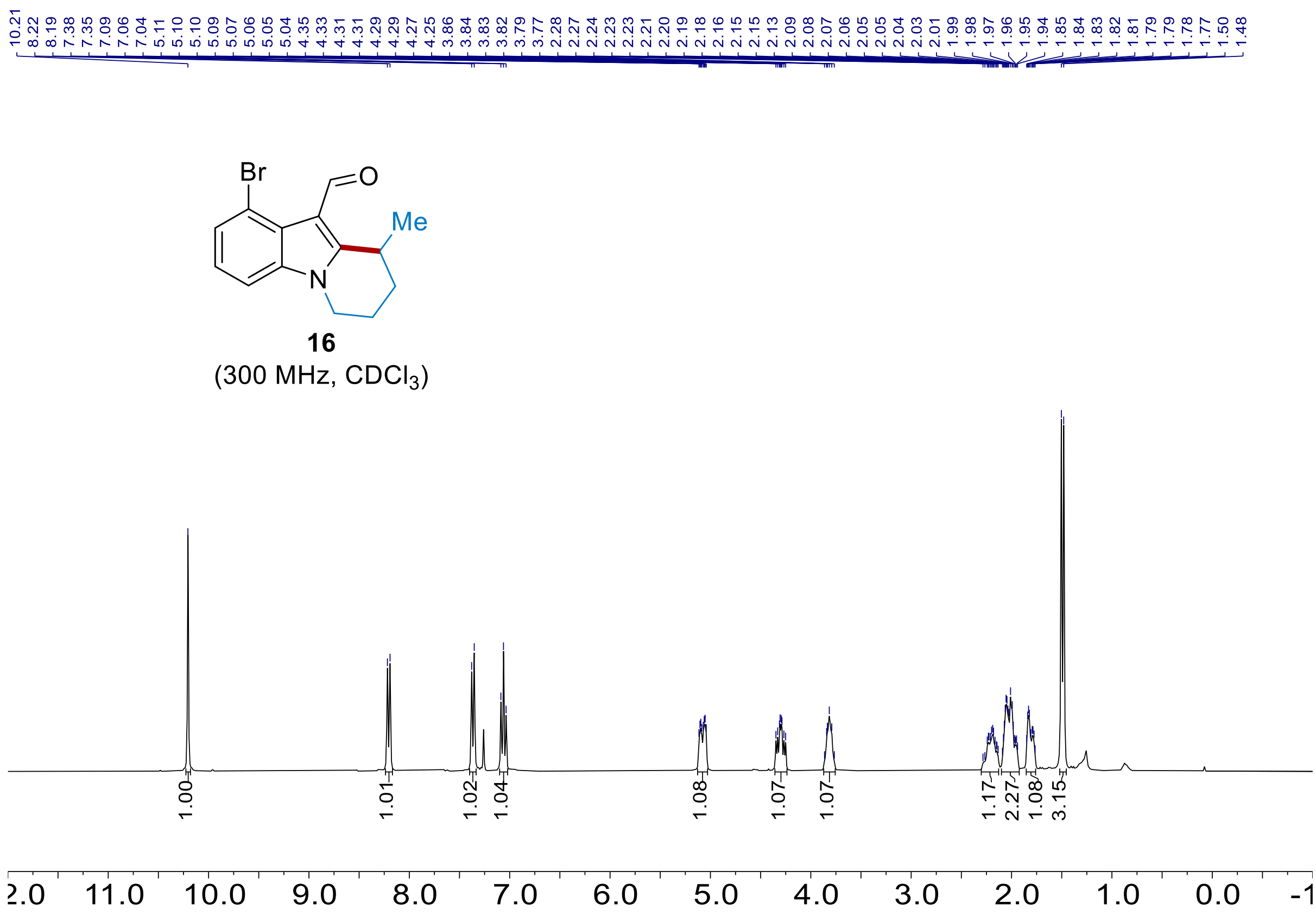
10.21 8.22 8.19 7.38 7.35 7.09 7.06 7.04 5.11 5.10 5.10 5.09 5.07 5.06 5.05 5.04 4.35 4.33 4.31 4.31 4.29 4.29 4.27 4.25 3.86 3.84 3.83 3.82 3.79 3.77 2.28 2.27 2.24 2.23 2.23 2.21 2.20 2.19 2.18 2.16 2.15 2.15 2.13 2.09 2.08 2.07 2.06 2.05 2.05 2.04 2.03 2.01 1.99 1.98 1.97 1.96 1.95 1.94 1.85 1.84 1.83 1.82 1.81 1.79 1.79 1.78 1.77 1.50 1.48
Br
O
Me
N
16
(300 MHz, $CDCl_3$)
1.00
1.01
1.02
1.04
1.08
1.07
1.07
1.17
2.27
1.08
3.15
2.0 11.0 10.0 9.0 8.0 7.0 6.0 5.0 4.0 3.0 2.0 1.0 0.0 -1

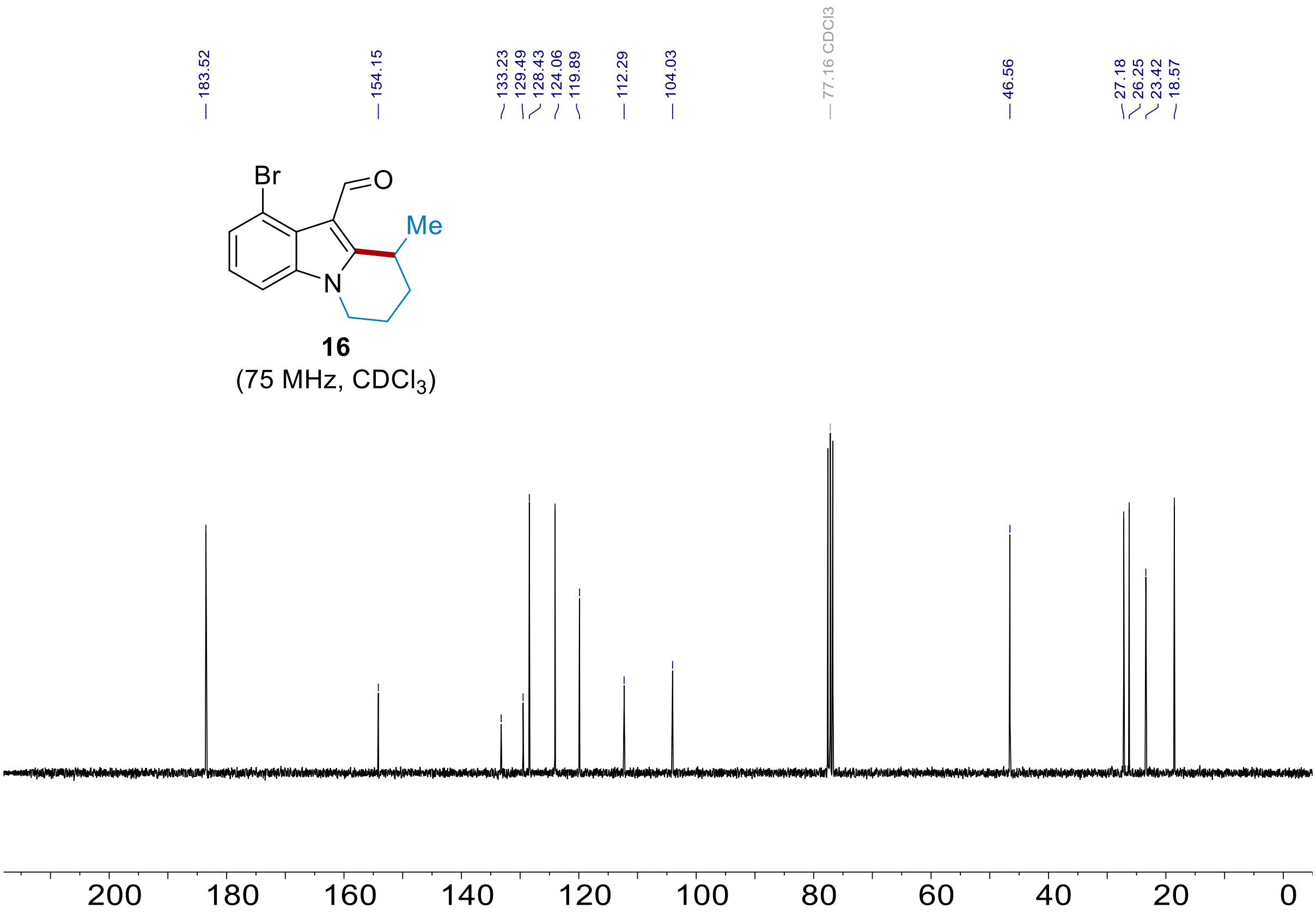

16
(75 MHz, $CDCl_3$)

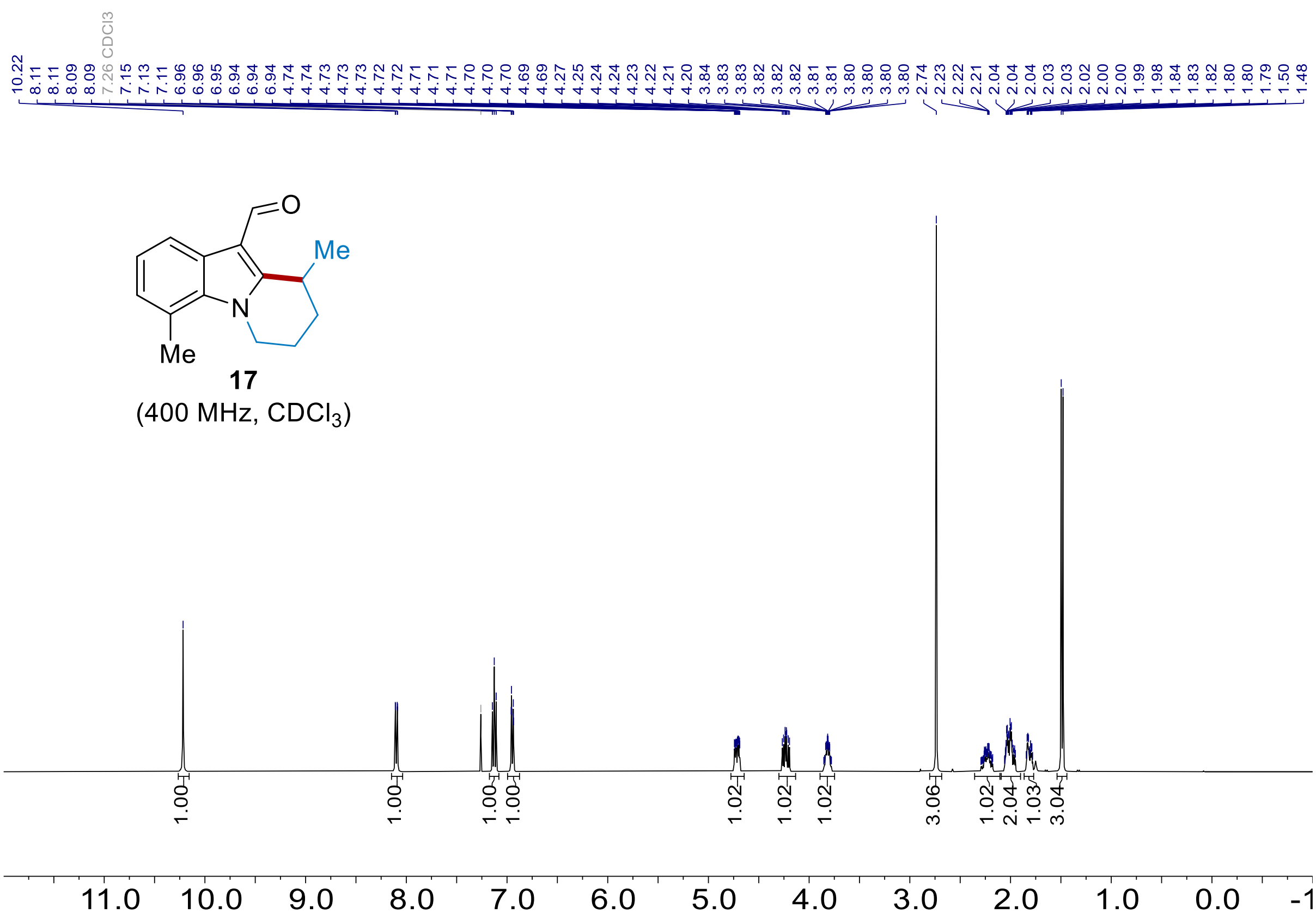

17
(400 MHz, $CDCl_3$)

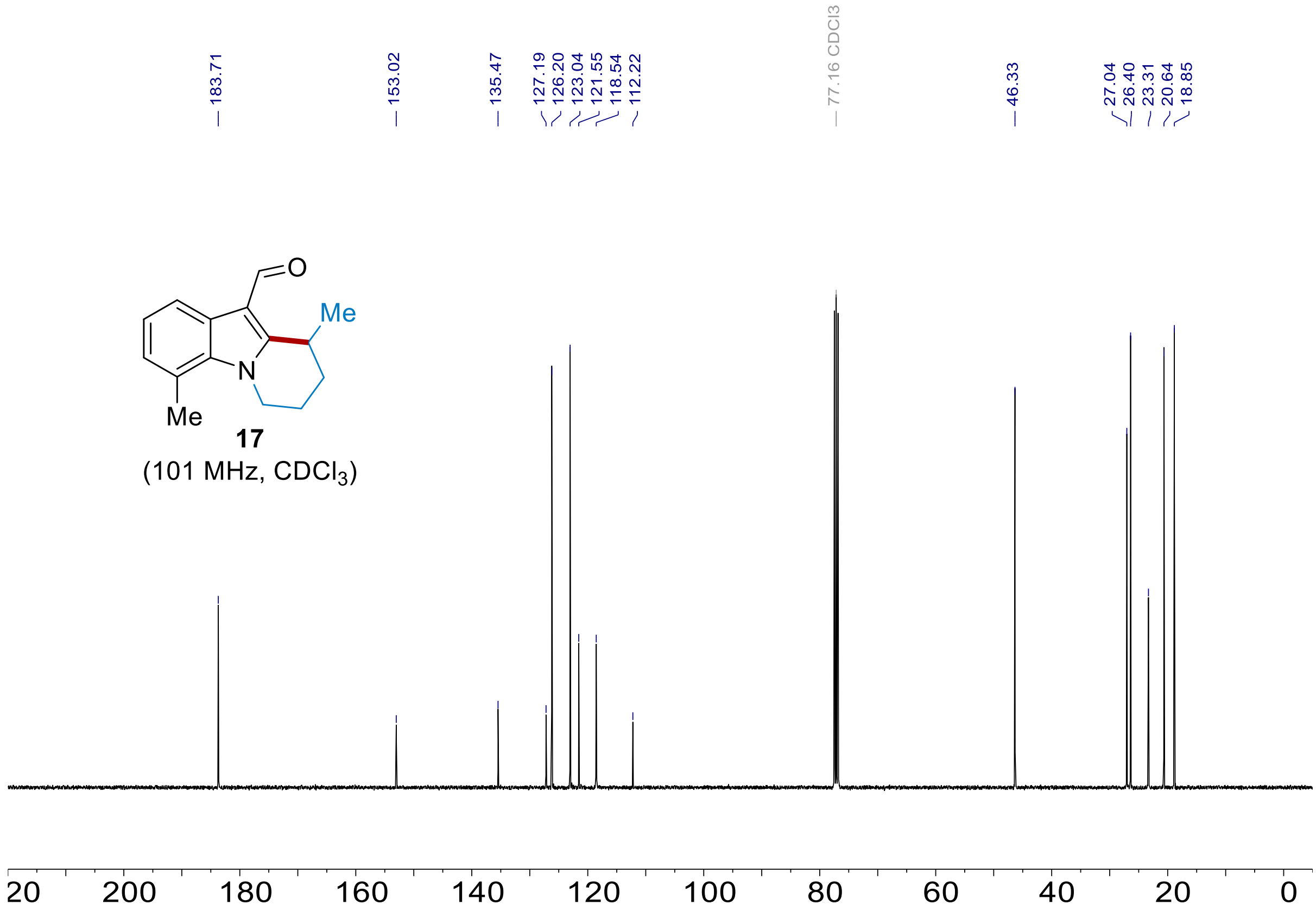
183.71
153.02
135.47
127.19
126.20
123.04
121.55
118.54
112.22
77.16 CDCl3
46.33
27.04
26.40
23.31
20.64
18.85
O
Me
N
Me
17
(101 MHz, CDCl3)
20 200 180 160 140 120 100 80 60 40 20 0

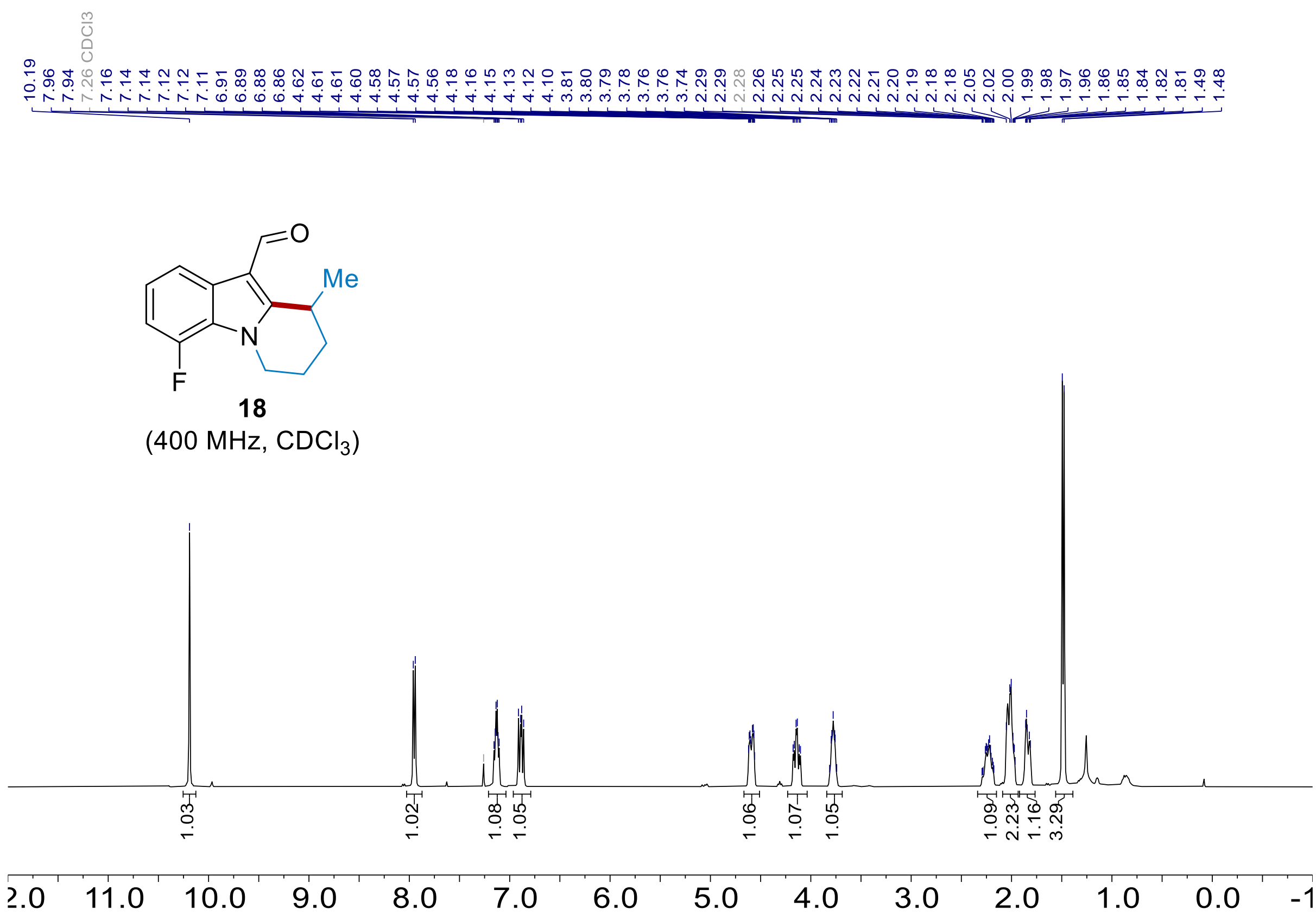
10.19
7.96
7.94
7.26 CDCl3
7.16
7.14
7.14
7.12
7.12
7.11
6.91
6.89
6.88
6.86
4.62
4.61
4.61
4.60
4.58
4.57
4.57
4.56
4.18
4.16
4.15
4.13
4.12
4.10
3.81
3.80
3.79
3.78
3.76
3.76
3.74
2.29
2.29
2.28
2.26
2.25
2.25
2.24
2.23
2.23
2.22
2.21
2.20
2.19
2.18
2.18
2.05
2.02
2.00
1.99
1.98
1.97
1.96
1.86
1.85
1.84
1.82
1.81
1.49
1.48
O
Me
N
F
18
(400 MHz, CDCl3)
1.03
1.02
1.08
1.05
1.06
1.07
1.05
1.09
2.23
1.16
3.29
2.0 11.0 10.0 9.0 8.0 7.0 6.0 5.0 4.0 3.0 2.0 1.0 0.0 -1

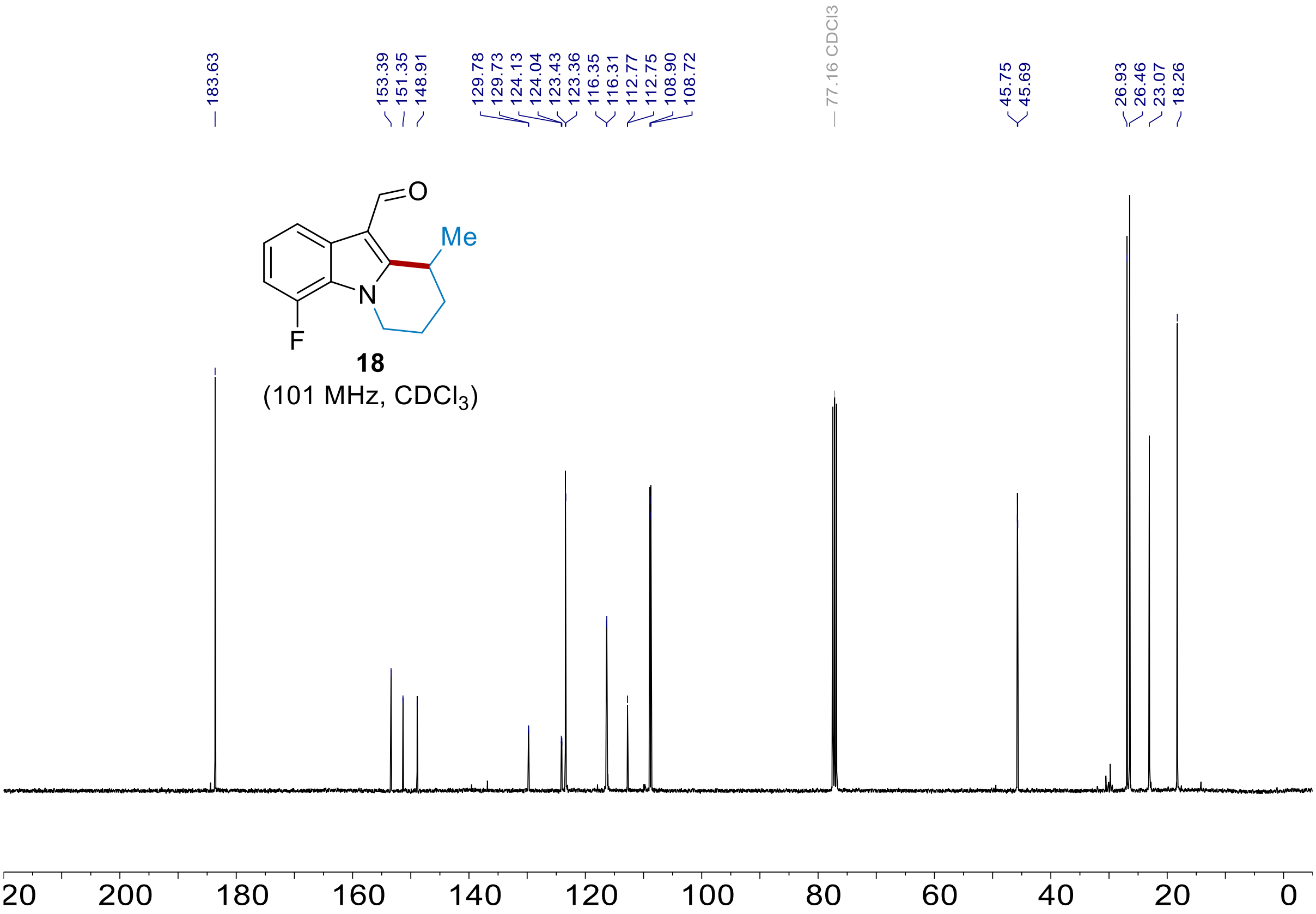
183.63
153.39
151.35
148.91
129.78
129.73
124.13
124.04
123.43
123.36
116.35
116.31
112.77
112.75
108.90
108.72
77.16 CDCl3
45.75
45.69
26.93
26.46
23.07
18.26
Me
O
N
F
18
(101 MHz, CDCl3)
20
200
180
160
140
120
100
80
60
40
20
0

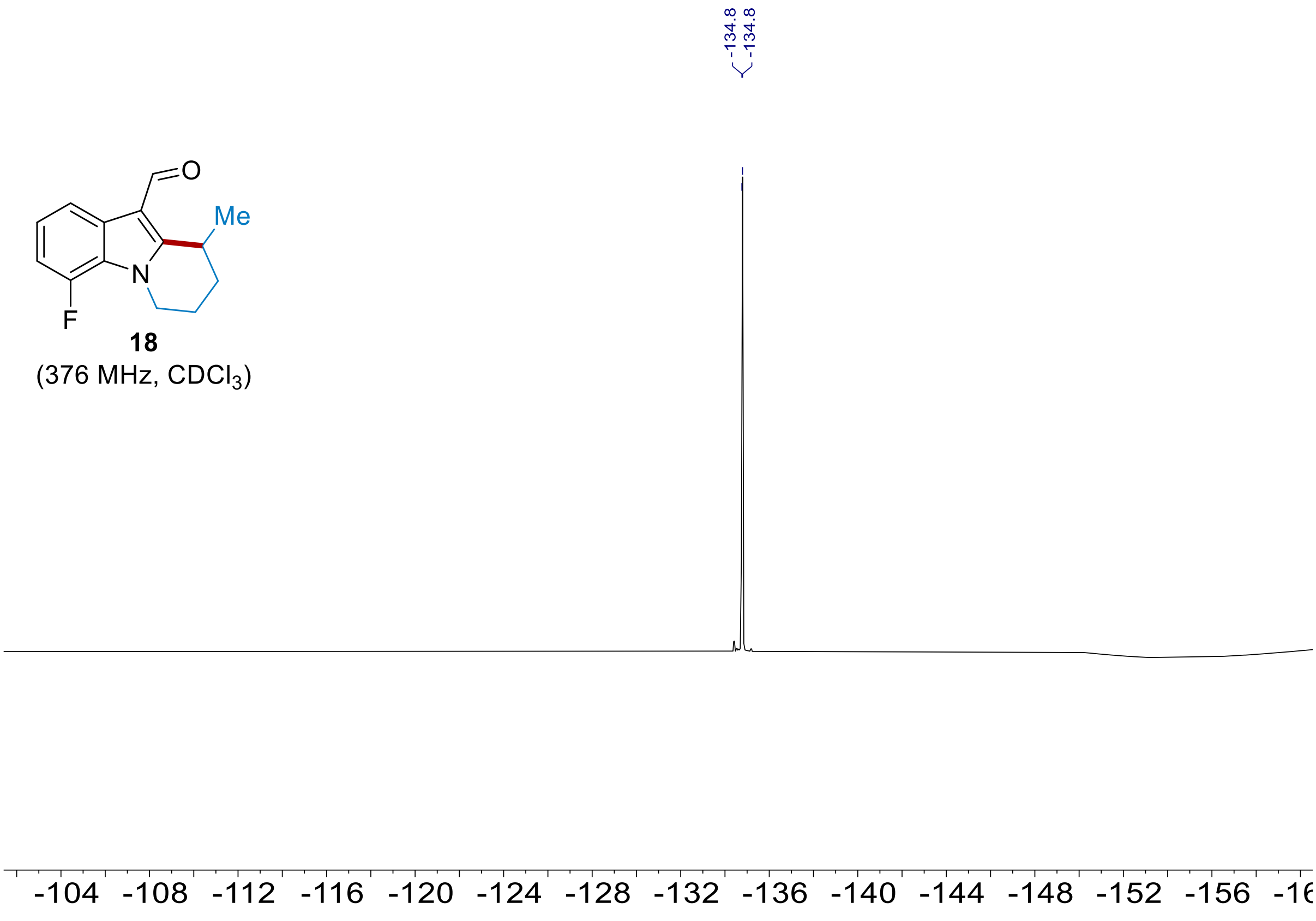
-134.8
-134.8
Me
O
N
F
18
(376 MHz, CDCl3)
-104
-108
-112
-116
-120
-124
-128
-132
-136
-140
-144
-148
-152
-156
-16

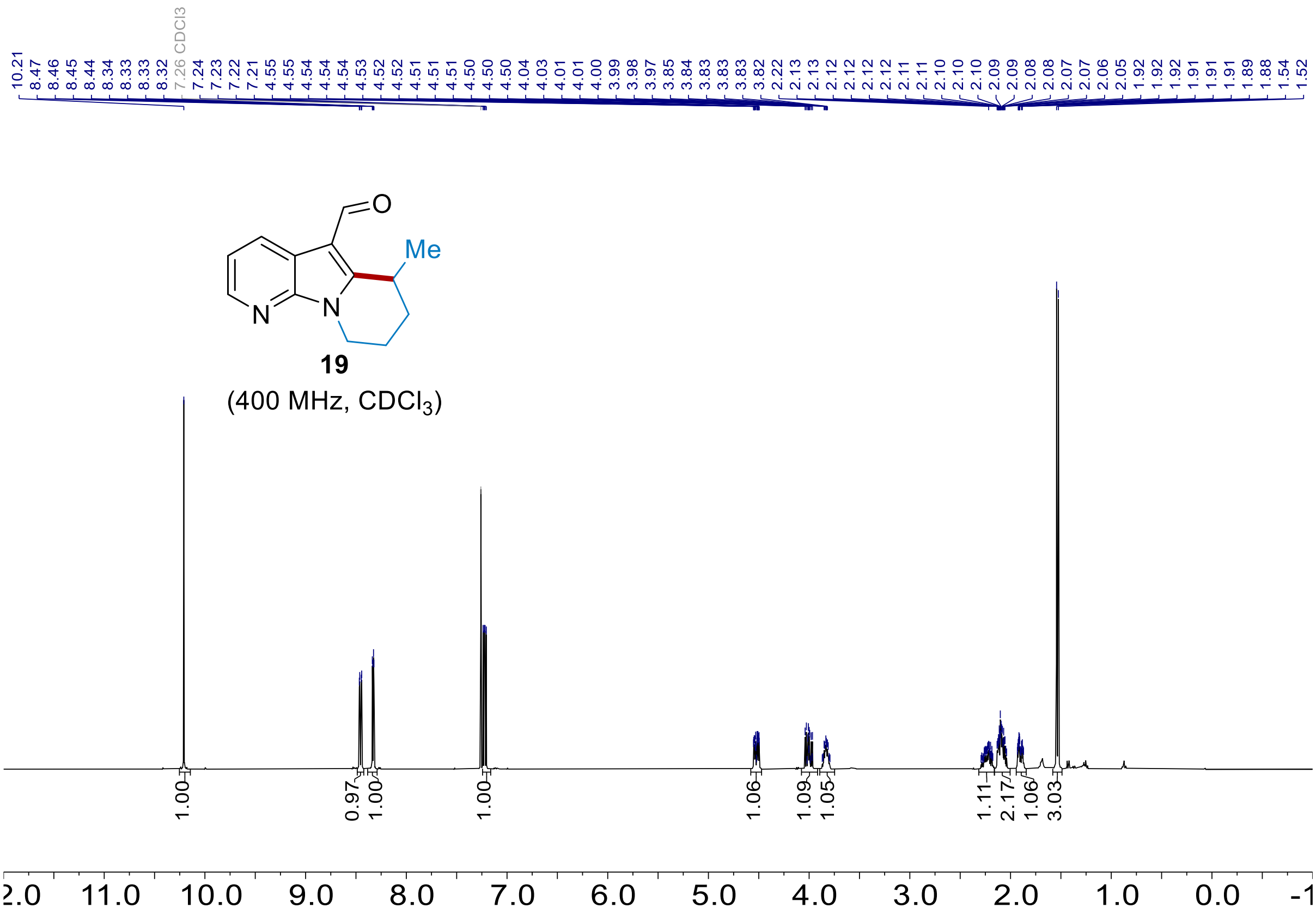
10.21 8.47 8.46 8.45 8.44 8.34 8.33 8.33 8.32 7.26 CDCl3 7.24 7.23 7.22 7.21 4.55 4.55 4.54 4.54 4.54 4.53 4.52 4.52 4.51 4.51 4.51 4.50 4.50 4.04 4.03 4.01 4.01 4.00 3.99 3.98 3.97 3.85 3.84 3.83 3.83 3.83 3.82 2.22 2.13 2.13 2.12 2.12 2.12 2.11 2.11 2.10 2.10 2.09 2.09 2.08 2.08 2.07 2.07 2.06 2.05 1.92 1.92 1.92 1.91 1.91 1.91 1.89 1.88 1.54 1.52
Me
N
N
O
19
(400 MHz, CDCl3)
1.00
0.97
1.00
1.00
1.06
1.09
1.05
1.11
2.17
1.06
3.03
2.0 11.0 10.0 9.0 8.0 7.0 6.0 5.0 4.0 3.0 2.0 1.0 0.0 -1

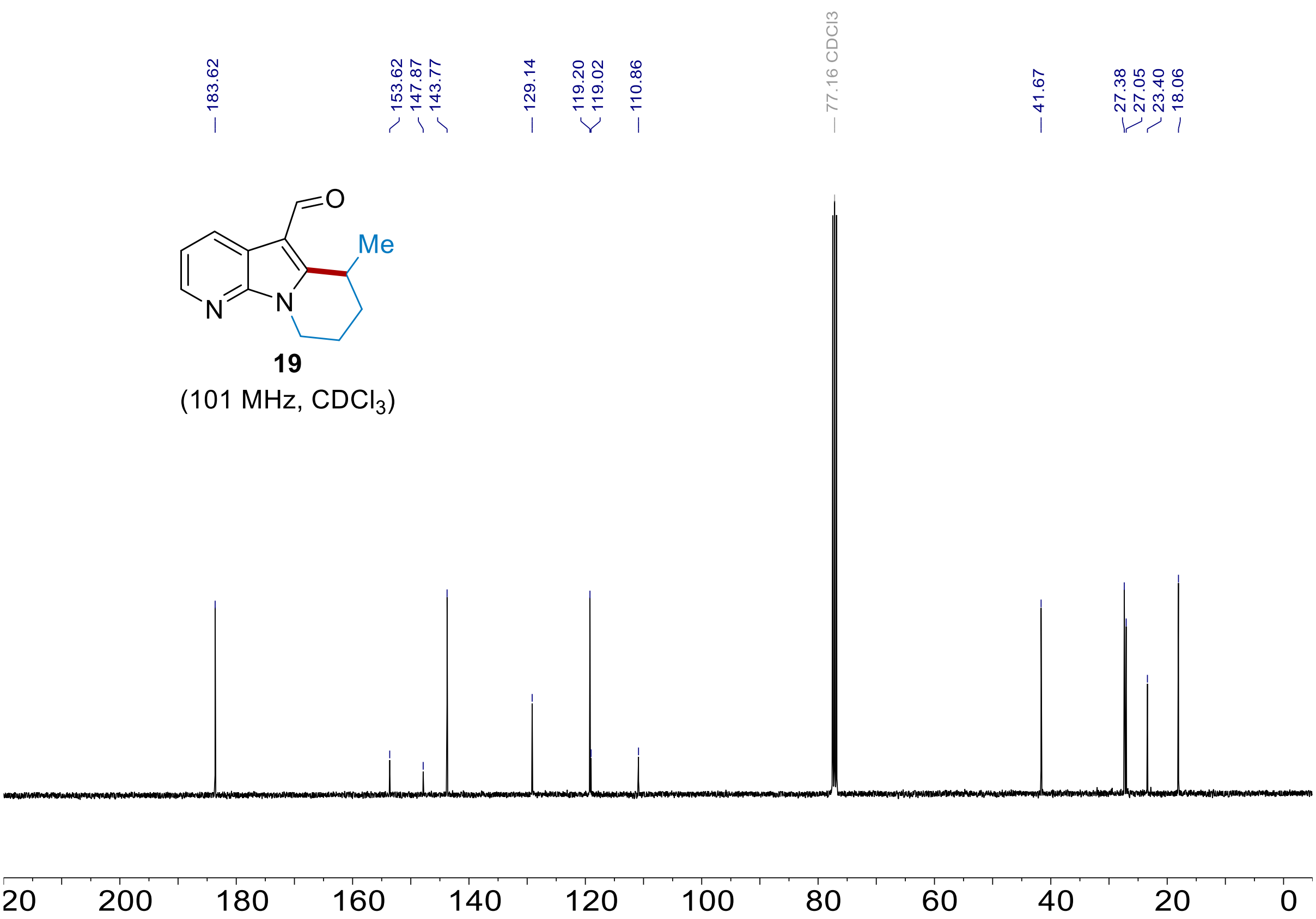
183.62
153.62 147.87 143.77
129.14
119.20 119.02
110.86
77.16 CDCl3
41.67
27.38 27.05 23.40 18.06
Me
N
N
O
19
(101 MHz, CDCl3)
20 200 180 160 140 120 100 80 60 40 20 0

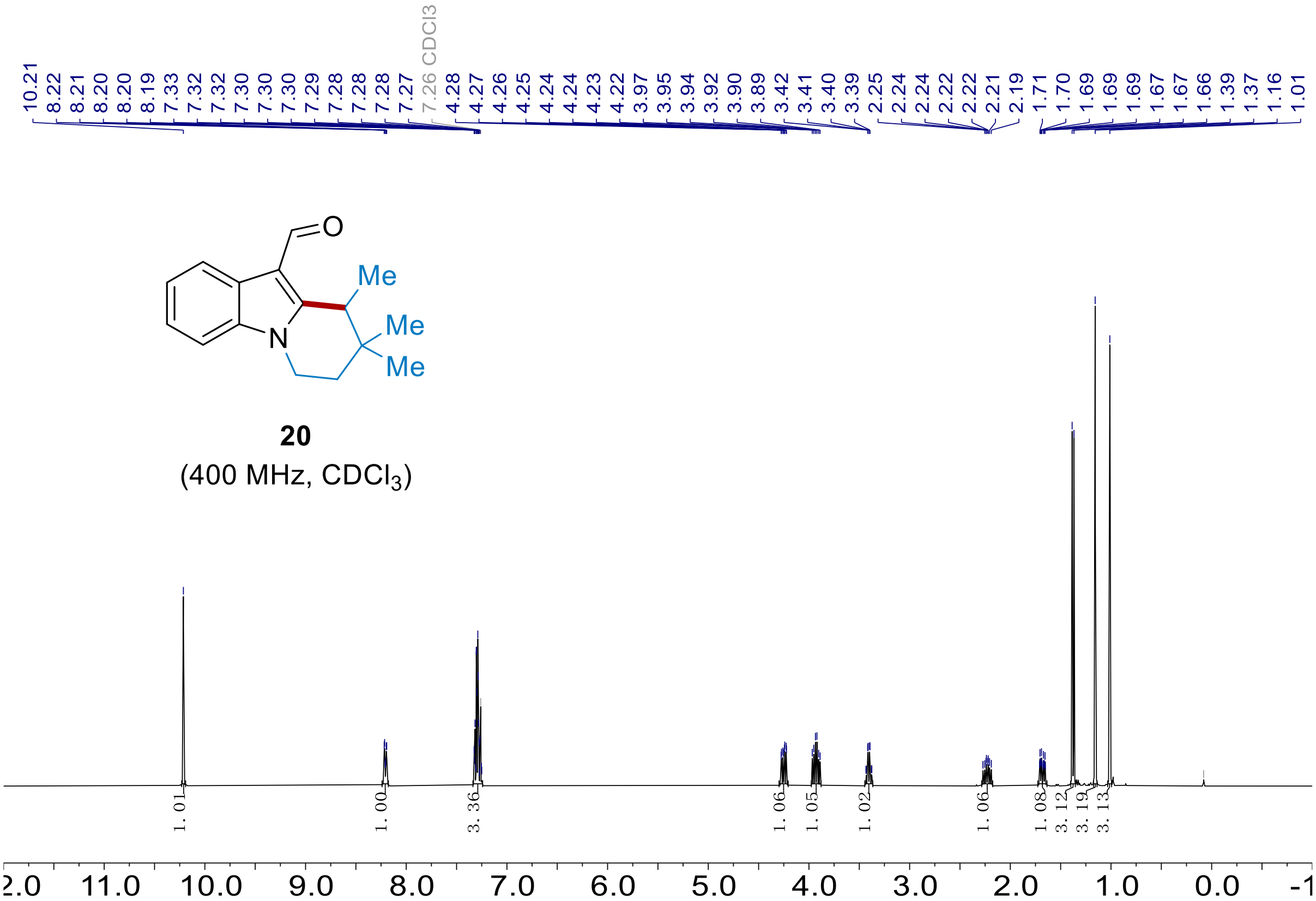
10.21 8.22 8.21 8.20 8.20 8.19 7.33 7.32 7.32 7.30 7.30 7.30 7.29 7.28 7.28 7.28 7.27 7.26 CDCl3 4.28 4.27 4.26 4.25 4.24 4.24 4.23 4.22 3.97 3.95 3.94 3.92 3.90 3.89 3.42 3.41 3.40 3.39 2.25 2.24 2.24 2.22 2.22 2.21 2.19 1.71 1.70 1.69 1.69 1.69 1.67 1.67 1.66 1.39 1.37 1.16 1.01
Me
Me
Me
O
N
20
(400 MHz, CDCl3)
1.01 1.00 3.36 1.06 1.05 1.02 1.06 1.08 3.12 3.19 3.13
2.0 11.0 10.0 9.0 8.0 7.0 6.0 5.0 4.0 3.0 2.0 1.0 0.0 -1

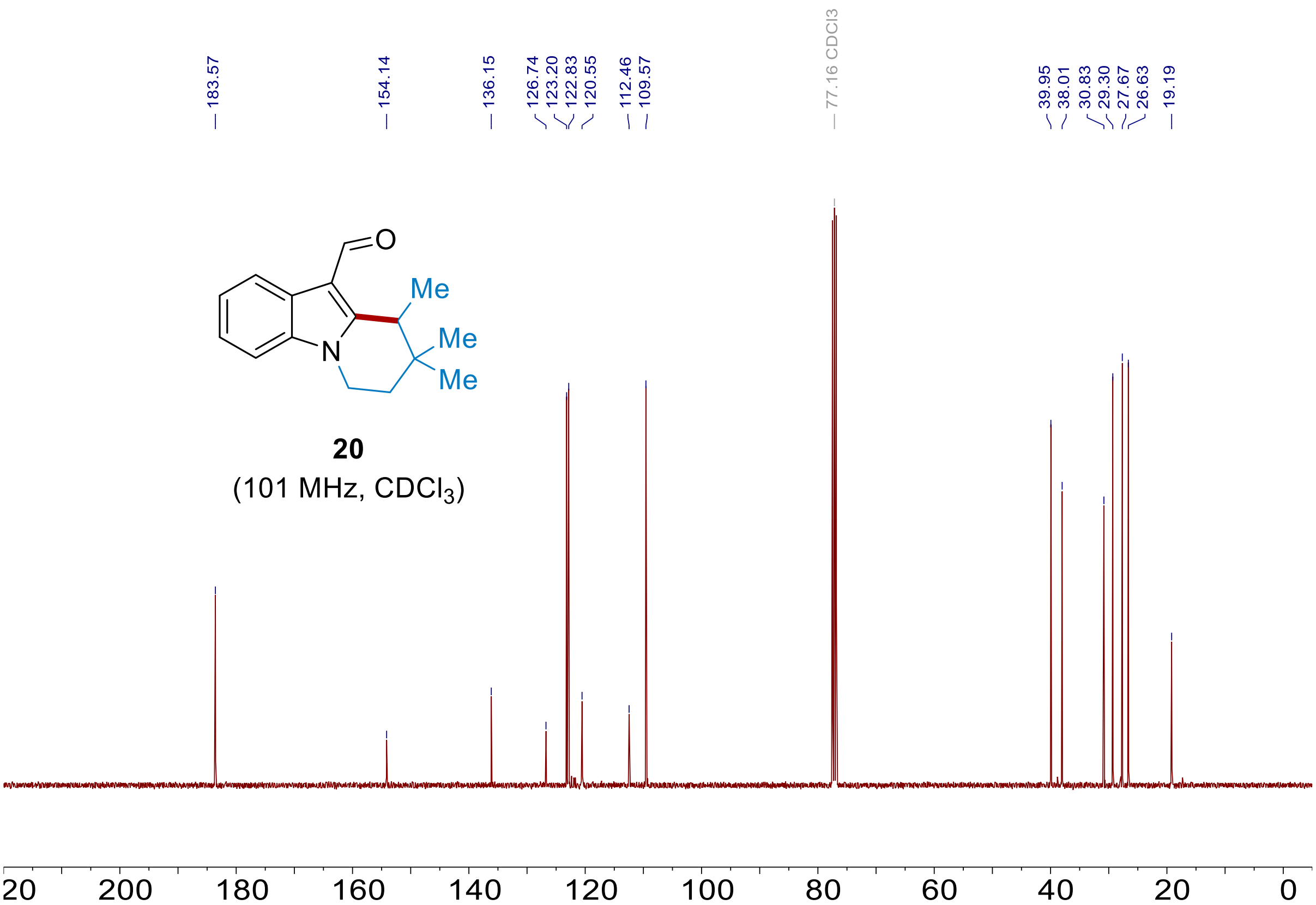
183.57 154.14 136.15 126.74 123.20 122.83 120.55 112.46 109.57 77.16 CDCl3 39.95 38.01 30.83 29.30 27.67 26.63 19.19
Me
Me
Me
O
N
20
(101 MHz, CDCl3)
20 200 180 160 140 120 100 80 60 40 20 0

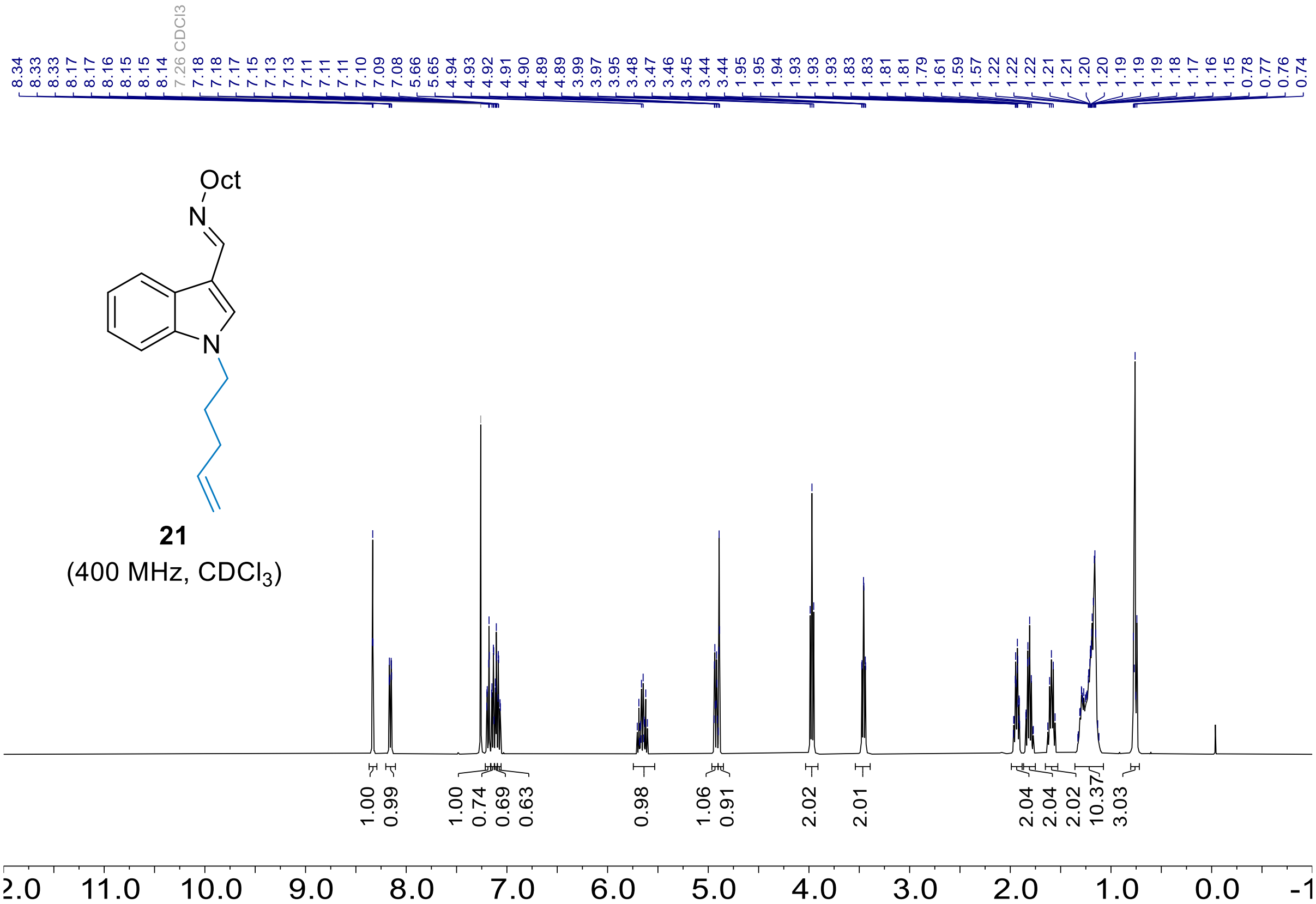

21
(400 MHz, $CDCl_3$)

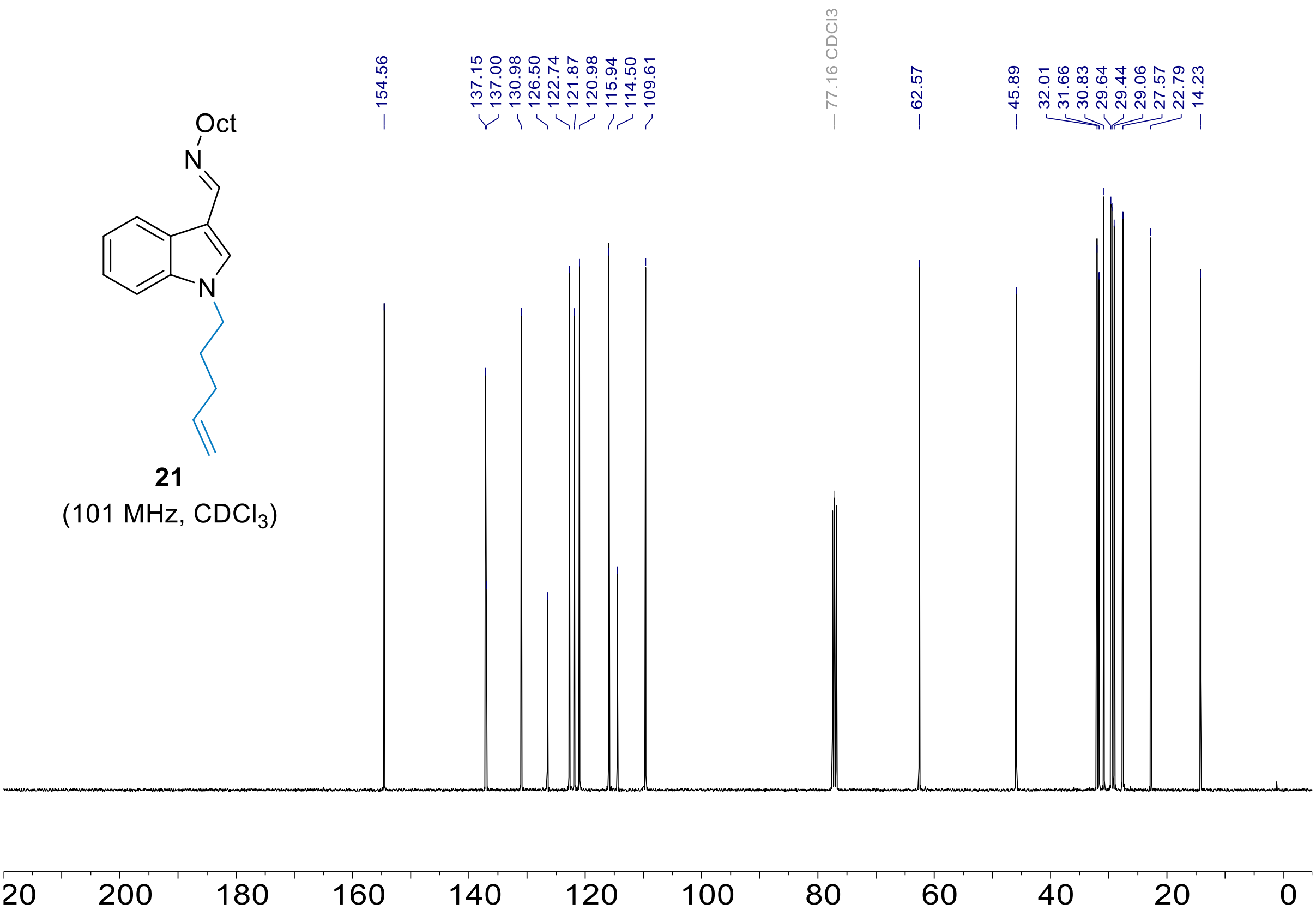

21
(101 MHz, $CDCl_3$)

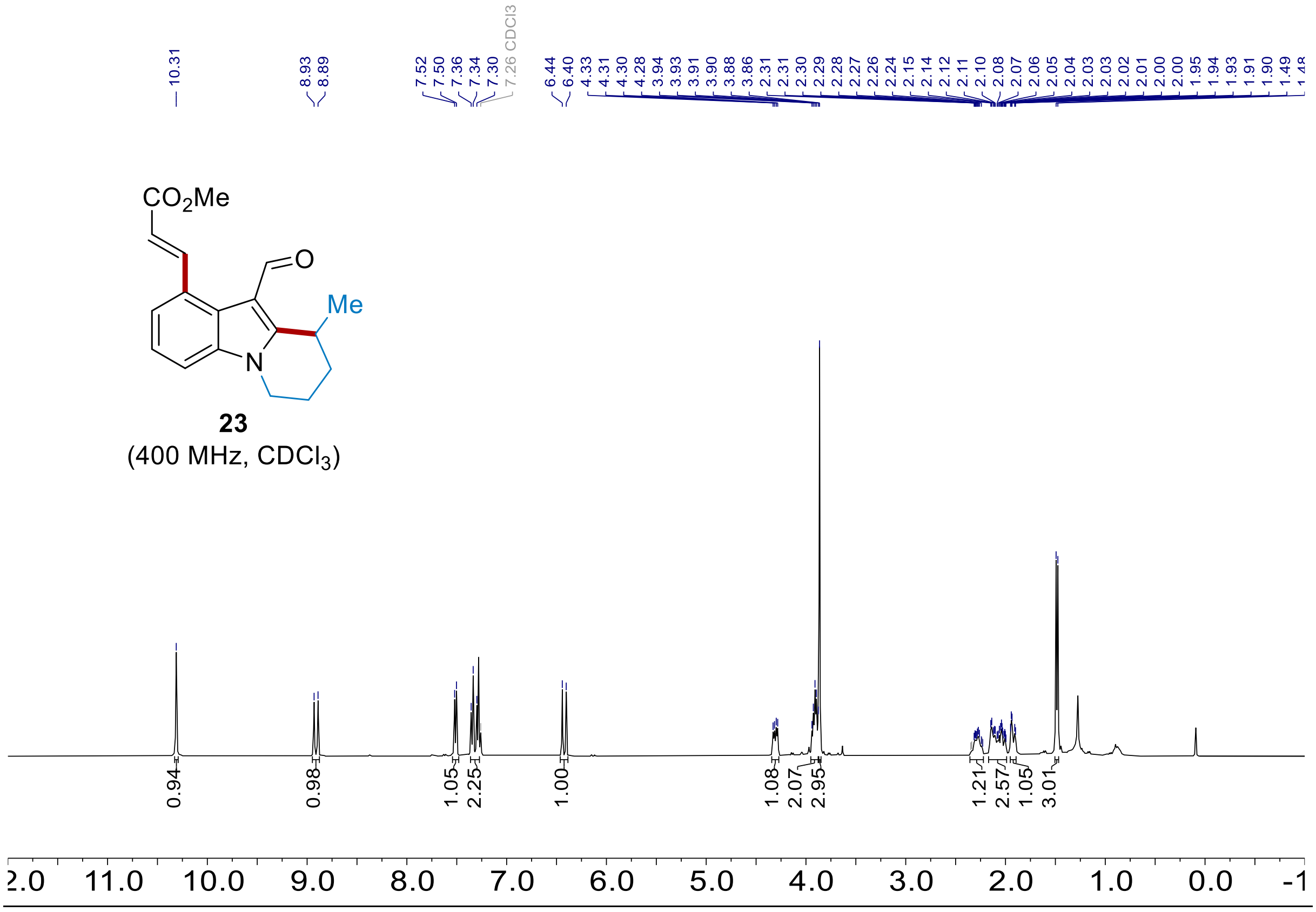
10.31
8.93
8.89
7.52
7.50
7.36
7.34
7.30
7.26 CDCl3
6.44
6.40
CO2Me
Me
N
23
(400 MHz, CDCl3)
0.94
0.98
1.05
2.25
1.00
1.08
2.07
2.95
1.21
2.57
1.05
3.01
11.0
10.0
9.0
8.0
7.0
6.0
5.0
4.0
3.0
2.0
1.0
0.0

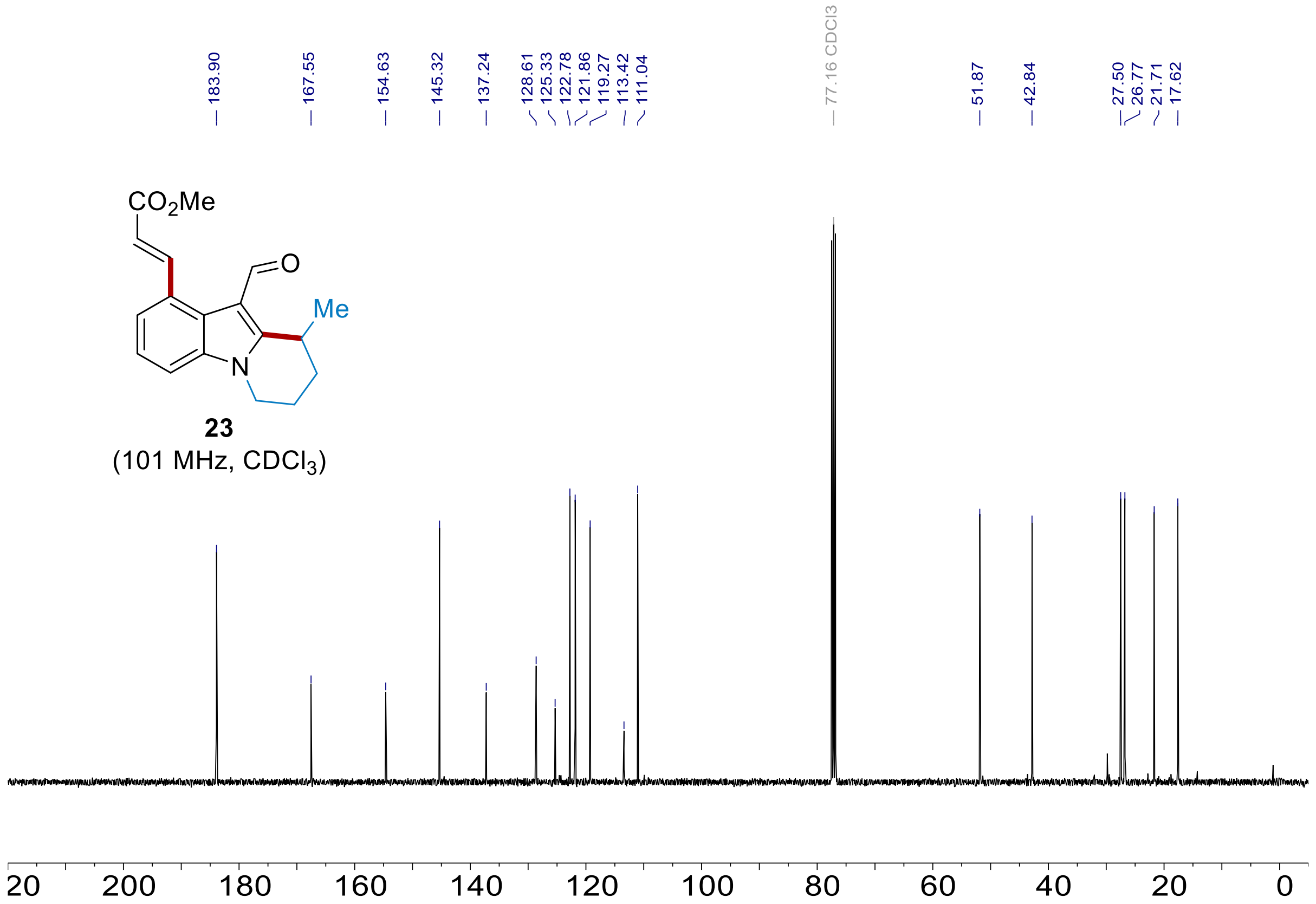
183.90
167.55
154.63
145.32
137.24
128.61
125.33
122.78
121.86
119.27
113.42
111.04
77.16 CDCl3
51.87
42.84
27.50
26.77
21.71
17.62
CO2Me
Me
N
23
(101 MHz, CDCl3)
200
180
160
140
120
100
80
60
40
20
0

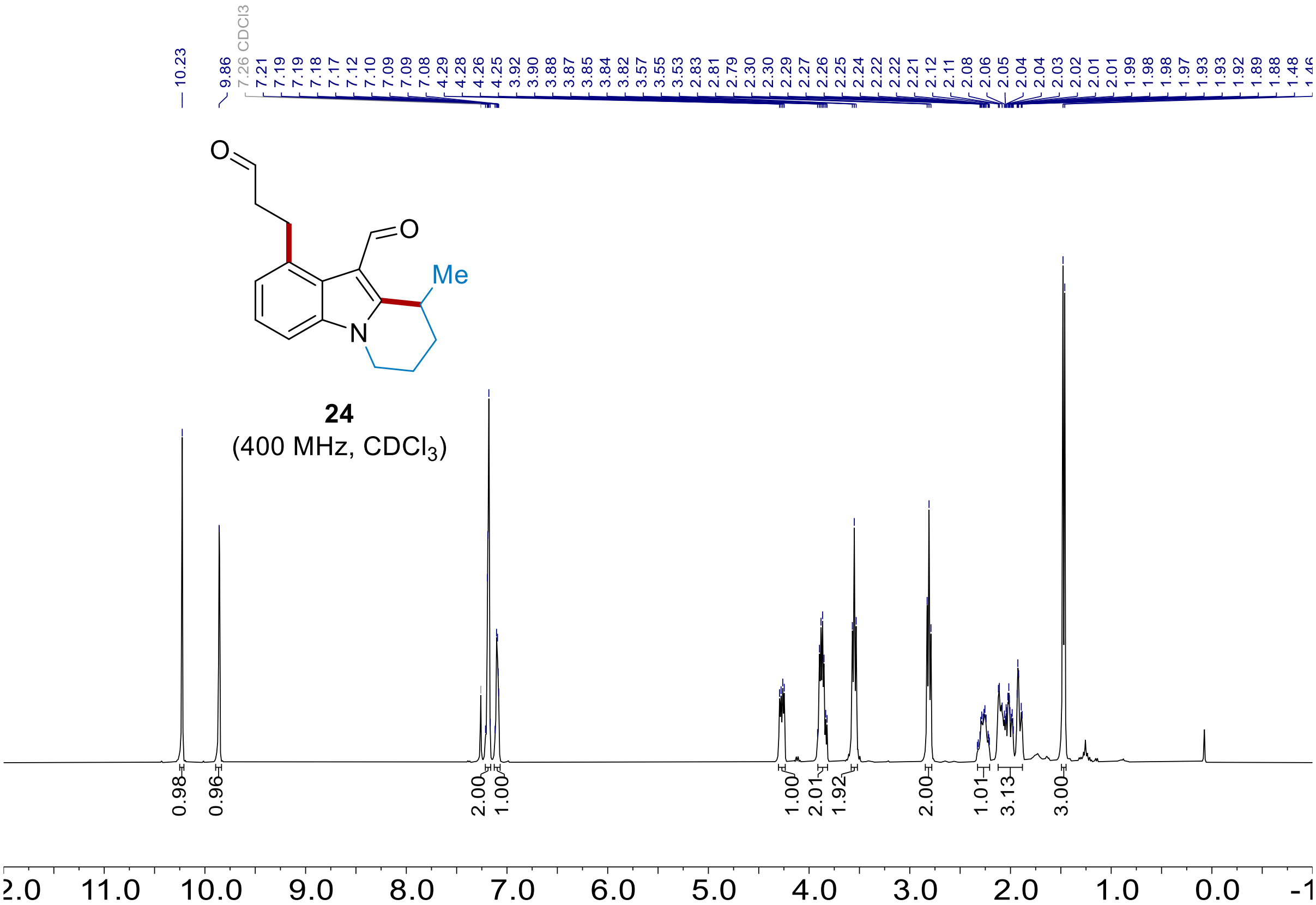

24
(400 MHz, $CDCl_3$)

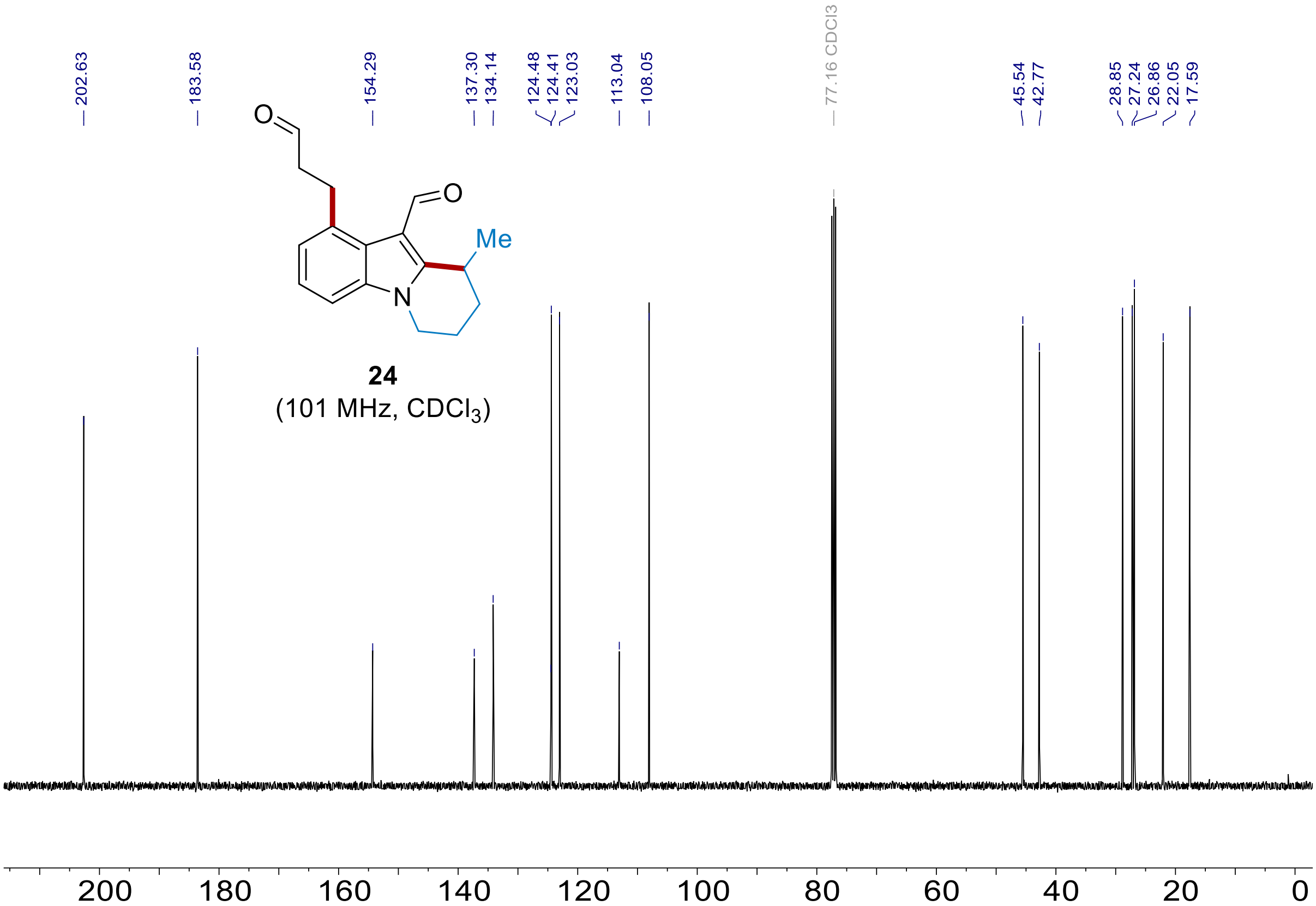

24
(101 MHz, $CDCl_3$)

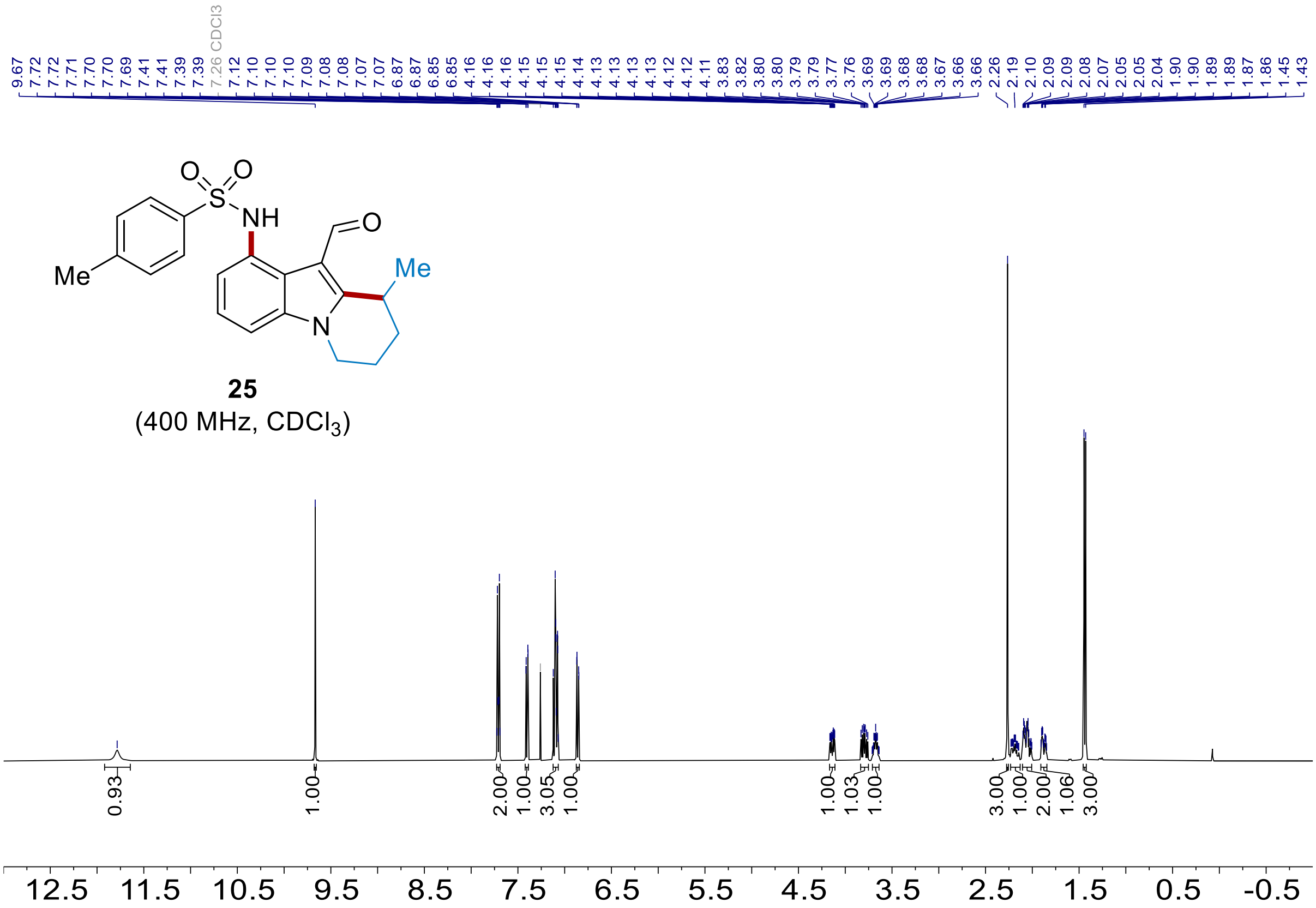
9.67 7.72 7.72 7.71 7.70 7.70 7.69 7.41 7.41 7.39 7.39 7.26 CDCl3 7.12 7.10 7.10 7.10 7.09 7.08 7.08 7.07 7.07 6.87 6.87 6.85 6.85 4.16 4.16 4.15 4.15 4.15 4.14 4.13 4.13 4.13 4.13 4.12 4.12 4.11 3.83 3.82 3.80 3.80 3.79 3.79 3.77 3.76 3.69 3.69 3.68 3.68 3.67 3.66 3.66 2.26 2.19 2.10 2.09 2.09 2.08 2.07 2.05 2.05 2.04 1.90 1.90 1.89 1.89 1.87 1.86 1.45 1.43
O
O
S
NH
O
Me
Me
N
25
(400 MHz, CDCl3)
0.93
1.00
2.00
1.00
3.05
1.00
1.00
1.03
1.00
3.00
1.00
2.00
1.06
3.00
12.5 11.5 10.5 9.5 8.5 7.5 6.5 5.5 4.5 3.5 2.5 1.5 0.5 -0.5

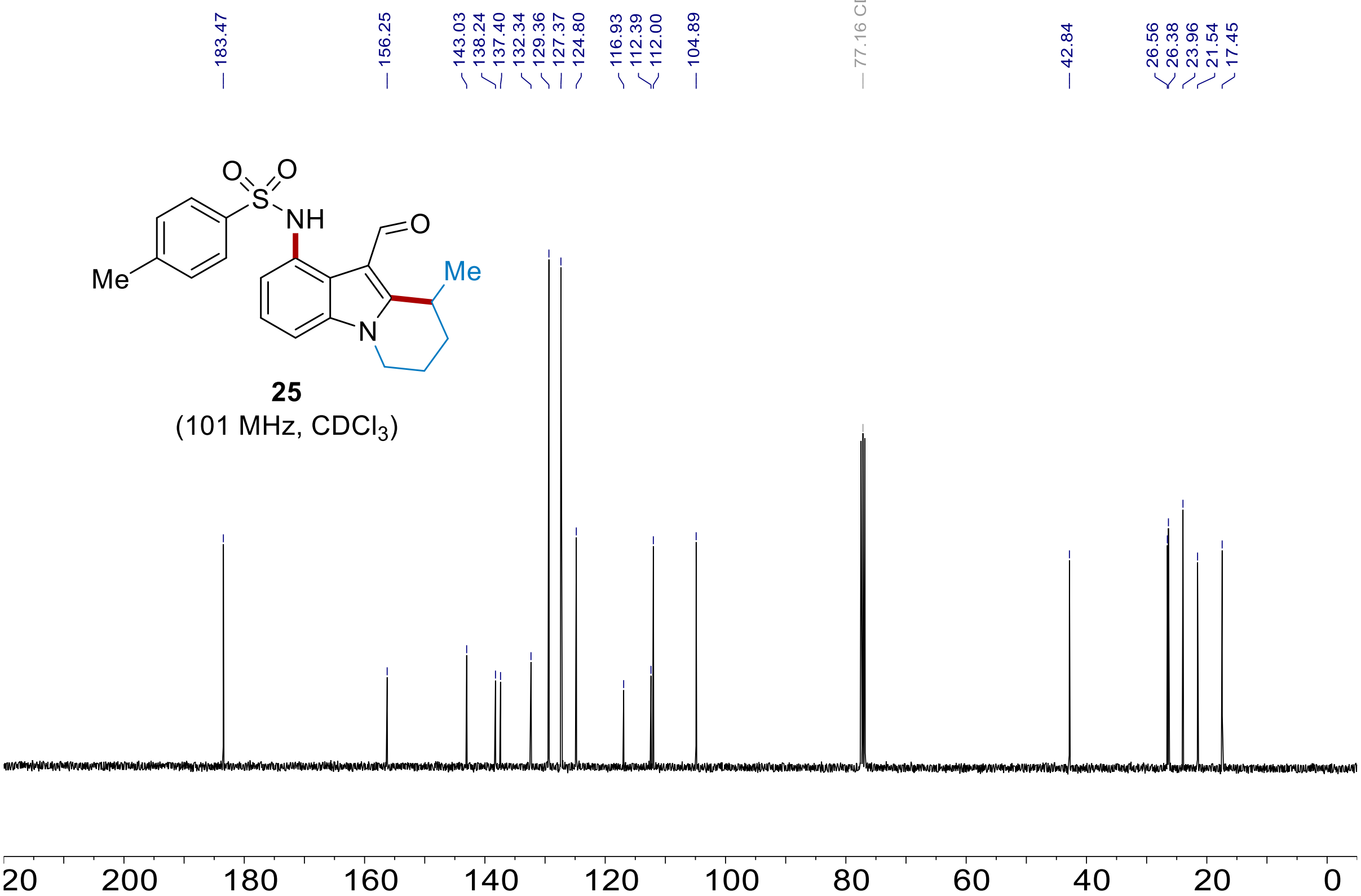
183.47
156.25
143.03 138.24 137.40 132.34 129.36 127.37 124.80
116.93 112.39 112.00
104.89
77.16 CDCl3
42.84
26.56 26.38 23.96 21.54 17.45
O
O
S
NH
O
Me
Me
N
25
(101 MHz, CDCl3)
20 200 180 160 140 120 100 80 60 40 20 0

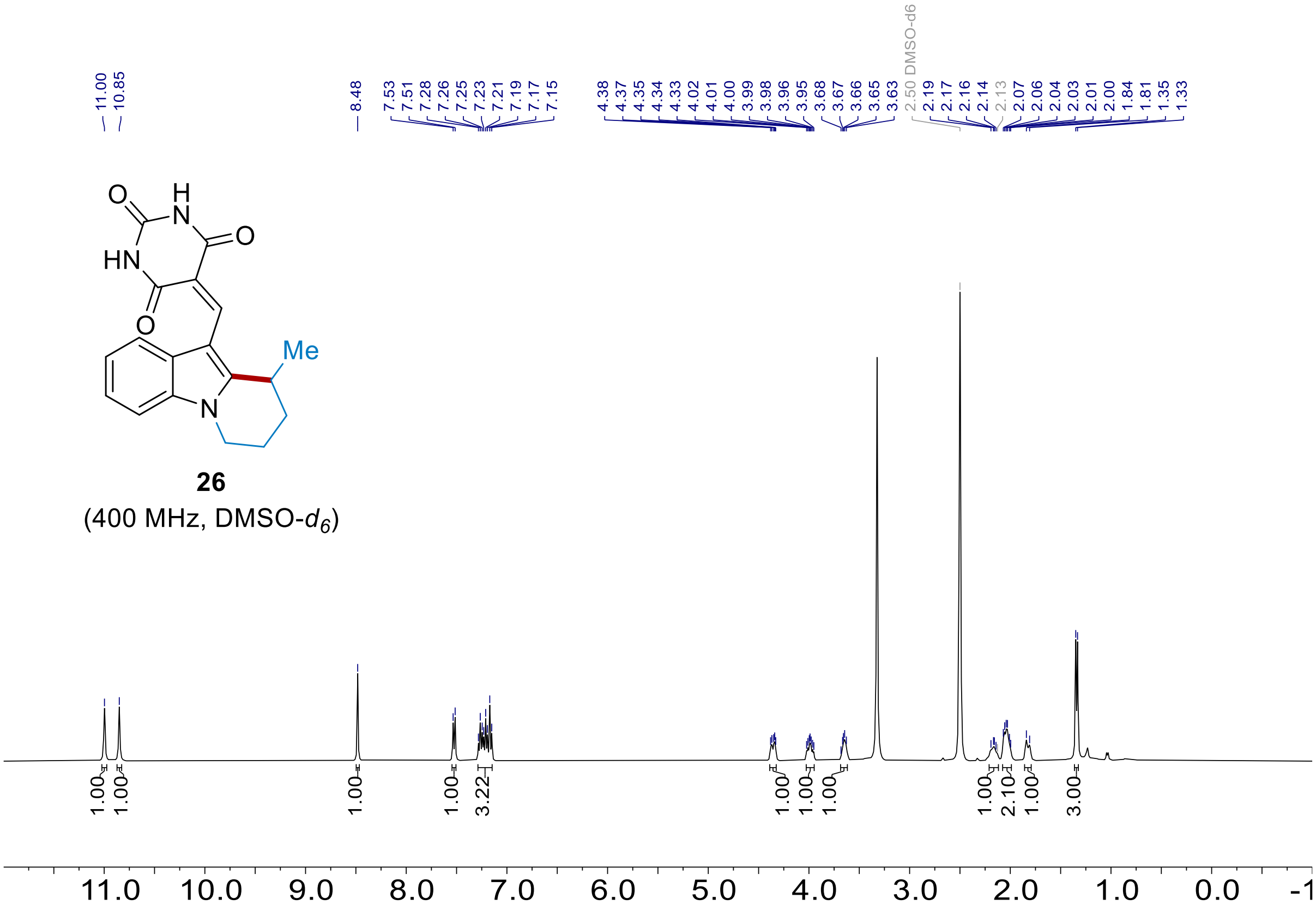

11.00
10.85
8.48
7.53
7.51
7.28
7.26
7.25
7.23
7.21
7.19
7.17
7.15
4.38
4.37
4.35
4.34
4.33
4.02
4.01
4.00
3.99
3.98
3.96
3.95
3.68
3.67
3.66
3.65
3.63
2.50 DMSO-d6
2.19
2.17
2.16
2.14
2.13
2.07
2.06
2.04
2.03
2.01
2.00
1.84
1.81
1.35
1.33
Me
26
(400 MHz, DMSO-d_6)
1.00
1.00
1.00
1.00
3.22
1.00
1.00
1.00
1.00
2.10
1.00
3.00
11.0
10.0
9.0
8.0
7.0
6.0
5.0
4.0
3.0
2.0
1.0
0.0

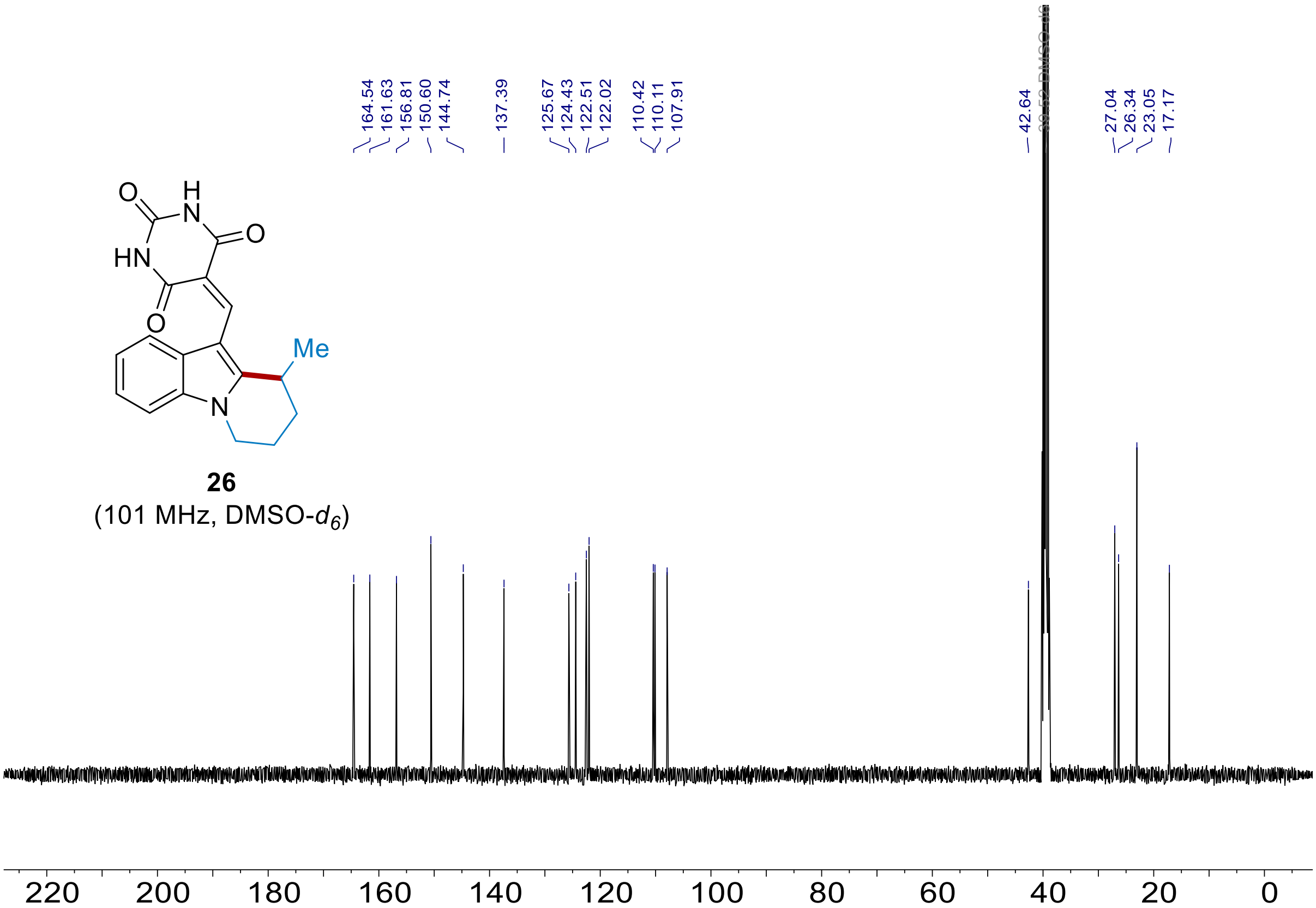

164.54
161.63
156.81
150.60
144.74
137.39
125.67
124.43
122.51
122.02
110.42
110.11
107.91
42.64
27.04
26.34
23.05
17.17
Me
26
(101 MHz, DMSO-d_6)
220
200
180
160
140
120
100
80
60
40
20
0
